# Exploring the Reactivity of Unsymmetrical Diphosphanes toward Heterocumulenes: Access to Phosphanyl and Phosphoryl Derivatives of Amides, Imines, and Iminoamides

**DOI:** 10.1021/acs.inorgchem.2c00589

**Published:** 2022-06-14

**Authors:** Natalia Szynkiewicz, Jarosław Chojnacki, Rafał Grubba

**Affiliations:** Department of Inorganic Chemistry, Faculty of Chemistry, Gdańsk University of Technology, 11/12 Gabriela Narutowicza Str., 80-233 Gdańsk, Poland

## Abstract

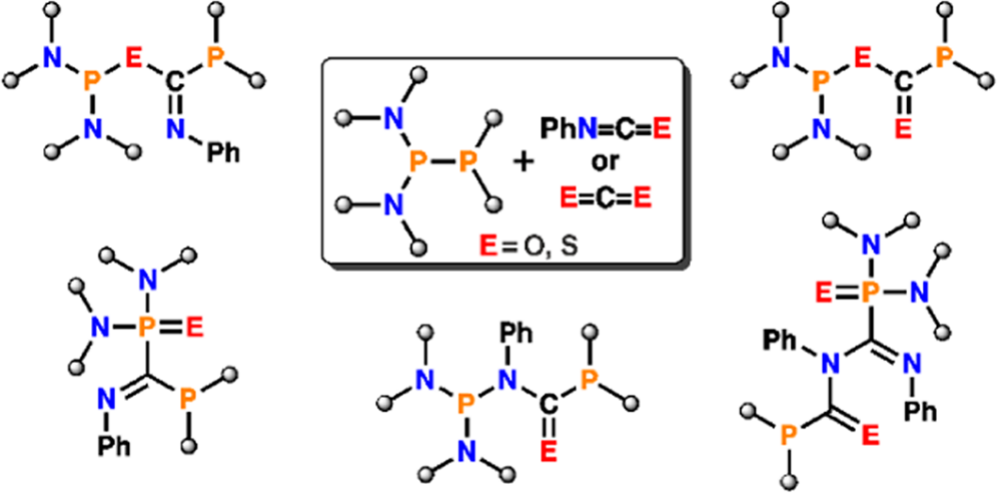

We present a comprehensive study on the diphosphanation of iso(thio)cyanates by unsymmetrical diphosphanes. The reactions involving unsymmetrical diphosphanes and phenyl isocyanate or phenyl thioisocyanate gave rise to phosphanyl, phosphoryl, and thiophosphoryl derivatives of amides, imines, and iminoamides. The structures of the diphosphanation products were confirmed through NMR spectroscopy, IR spectroscopy, and single-crystal X-ray diffraction. We showed that unsymmetrical diphosphanes could be used as building blocks to synthesize phosphorus analogues of important classes of organic molecules. The described transformations provided a new methodology for the synthesis of organophosphorus compounds bearing phosphanyl, phosphoryl, or thiophosphoryl functional groups. Moreover, theoretical studies on diphosphanation reactions explained the influence of the steric and electronic properties of the parent diphosphanes on the structures of the diphosphanation products.

## Abstract 2

We provided synthetic access to phosphanyl, phosphoryl, or thiophosphoryl derivatives of amides, imines, and iminoamides starting from simple building blocks such as unsymmetrical diphosphanes and heterocumulenes.

## 1 Introduction

**Scheme 1:**
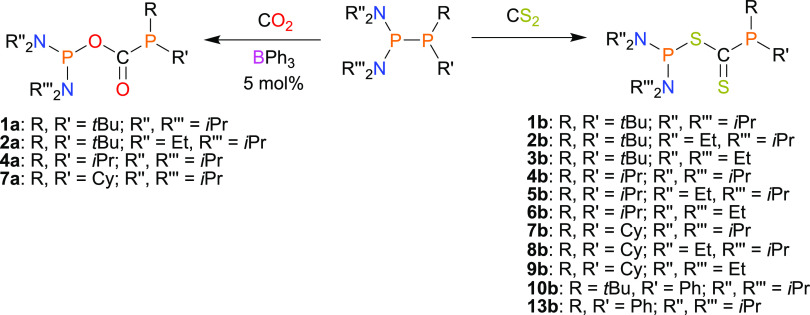
Reactions of Unsymmetrical Diphosphanes with CO_2_ and CS_2_

The development of pure, efficient, and inexpensive methods to obtain organophosphorus compounds is invariably a challenge in modern organic synthesis. Among many synthetic approaches, metal-free activation of small molecules employing low-valent phosphorus compounds has been of particular interest in recent years.^1−5^ In the vast majority of such reactions, the P center acts as a Lewis base that, either by itself^6,7^ or in cooperation with a Lewis acidic center in an ambiphilic system,^8−15^ binds to the activated molecule, leading to its functionalization. Reactions employing Lewis bases with P–P bonds are still an unexplored area of research,^16−18^ with the largest portion of reports devoted to the smallest representatives of this group—diphosphanes.^19−24^ When they react with electrophiles, the P–P bond is either retained, and thus diphosphane acts as a classical P-based nucleophile, or the initial electrophilic attack is followed by cleavage of the P–P bond, which in turn can lead to double phosphination of the activated molecule.^25−28^ To date, the vast majority of studies have focused on the diphosphanation of simple unsaturated organic compounds.^29^ Miura et al. and Sato et al. showed that two PR_2_ groups add to a double or a triple carbon–carbon bond in alkenes, alkynes, or arynes in either catalyzed or photoinitiated reactions with commercially available Ph_2_P–PPh_2_ to give 1,2-bis(diphenylphosphino) derivatives.^29−34^ By tailoring the properties of diphosphanes so that the P–P bond is more labile, it is possible to increase the diversity of the PRR′ moieties and provide a more general approach to the straightforward functionalization of the C = C or C≡C bonds. Gudat et al. described the reactivity of two types of such systems: unsymmetrical species bearing polarized P–P bonds^35−38^ and symmetrical species that undergo homolytic cleavage of the P–P bond in the solution to form two phosphinyl radicals.^23,39,40^ Both enable facile diphosphanation, however, only when reacting with electron-poor alkenes and alkynes.^27^ Indeed, the presence of the electron-withdrawing group in the vicinity of the multiple bond facilitates the diphosphanation reaction as well. Pringle et al. reported a one-step *Z*-stereospecific addition reaction of symmetrical and unsymmetrical diphosphanes to activated alkynes: acetylene mono- and dicarboxylates, giving *Z*-1,2-bis(phosphinyl)ethenes featuring diversified alkyl and aryl P-substituents.^21^ In addition to alkynes and alkenes, diphosphanes may also functionalize unsaturated compounds bearing carbon-heteroatom multiple bonds, for example, they could functionalize the C=O and C=S bonds through base-induced addition of (CF_3_)_2_P–P(CF_3_)_2_ to the carbonyl group of acetone.^41^ Masuda et al. found that persistent (H_2_C)_2_(NDipp)_2_P• radicals generated in solution from the parent symmetrical diphosphane react with heteroallenes as well: the phosphinyl fragments add across the C=O or C=S double bond of PhNCO, PhNCS, and CS_2_; however, they remain unreactive toward CO_2_.^42,43^ Moreover, the reaction of Masuda’s diphosphane [(H_2_C)_2_(NDipp)_2_P]_2_ with chalcogens (S, Se, Te) led to the insertion of one or two chalcogen atoms into P–P bond.^42^ In a prior study, we described the first example of a BPh_3_-catalyzed activation of carbon dioxide by a P–P bond system.^28^ We showed that both CO_2_ and CS_2_ might be inserted into the polarized P–P bond of unsymmetrical 1,1-diaminodiphosphanes to form phosphanyl derivatives of formic and dithioformic acid of the general formula (R_2_N)_2_P–E–C(=E)–P*t*Bu_2_ (E = O, S), also in a reversible manner. Our recent experiments involving a wide range of unsymmetrical P–P systems confirm that CO_2_ forms stable P–O–C(=O)–P products in the reaction with diphosphanes bearing highly nucleophilic **P**RR′ atom (R, R′ = *t*Bu, *i*Pr or Cy) and elongated P–P bonds in a range of 2.2278(4)–2.295(3) Å resulting from bulky substituents. Conversely, the formation of P–S–C(=S)–P systems is less demanding, and only one of these criteria has to be met (Scheme 1, see Supporting Information (SI) section Reactivity of Diphosphanes towards CO_2_ and CS_2_ for details).

Following our work, Schulz et al. reported that analogous *C*-substituted R_1_R_2_P–S–C(=S)–PR_3_R_4_ species might be obtained in the reaction of CS_2_ with sterically demanding secondary potassium phosphides followed by coupling with the respective secondary chlorophosphane.^44^ Moreover, depending on the R_1_–R_4_ substituents, these species may undergo a migration reaction to form phosphanyl thioketone of the general formula R_1_R_2_P–C(=S)–P(=S)R_3_R_4_.

Since the reactivity of diphosphanes toward heteroallenes remains a scarcely explored area, with no reports on the scope of PhNCO and PhNCS diphosphanation and determinants of efficient addition to the C=E bond, we decided to address these issues.

## 2 Results and Discussion

**Chart 1:**
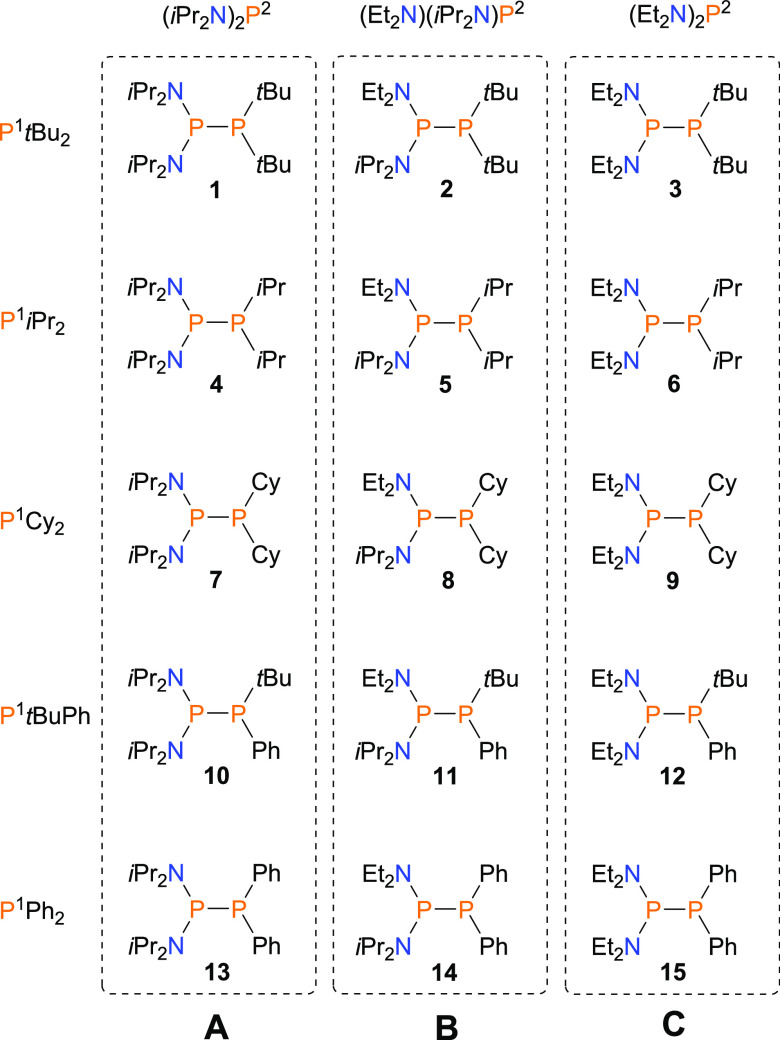
Unsymmetrical Diphosphanes Selected for Reactivity Studies

To this end, we tested the reactivity of a wide range of unsymmetrical diphosphanes **1–15** (Chart 1) toward PhNCO and PhNCS. The phosphorus reagents **1**–**15** contain PRR′ fragment varying in both nucleophilicity (see SI Table S31) and steric hindrance of the P center: P*t*Bu_2_ (**1–3**), *Pi*Pr_2_ (**4–6**), PCy_2_ (**7–9**), P*t*BuPh (**10–12**), PPh_2_ (**13–15**).^45,46^ Additionally, they can be divided into three groups, **A**, **B**, and **C**, depending on the bulkiness of the amino-substituted fragments such as (*i*Pr_2_N)_2_P, (Et_2_N)(*i*Pr_2_N)P, and (Et_2_N)_2_P, respectively (Chart 1). The syntheses of **1–3**, **10–13**, and **15** were reported previously by us.^19,28^ The synthetic procedures and X-ray and spectroscopic data of **4–9** and **13** were collected through the SI. All reactions were performed in toluene at room temperature using an excess of heterocumulene reagents in the absence of any catalyst. While the polarization of the C=O bond in CO_2_ is similar to that of PhNC=O, the C=S bond of PhNC=S is more polarized than that in CS_2_ and thus can be much more reactive (Table S32). The greater solubility of PhNCO and PhNCS in toluene compared to that of CO_2_ in this solvent facilitates the diphosphanation reaction. PhNCO and PhNCS are also more sterically crowded than CE_2_, which in turn may slow down the reaction itself but also limit the possibility of further unwanted rearrangements and product decomposition, as was the case with CS_2_.

**Chart 2:**
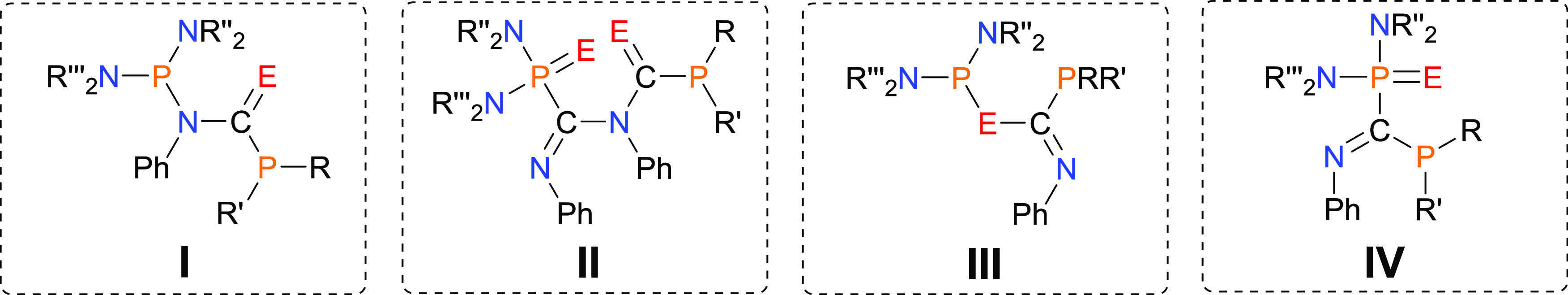
Structural Motifs of Diphosphanation Products (E = O, S)

In comparison to reactions involving CO_2_ and CS_2_, unsymmetrical diphosphanes exhibit more diversified reactivity toward phenyl isocyanate and thioisocyanate. All obtained diphosphanation products can be divided into four groups depicted in Chart 2: phosphanyl derivatives of amides (**I**), phosphanyl and (thio)phosphoryl derivatives of imino(thio)amides (**II**), phosphanyl derivatives of imino thioesteres (**III**), and phosphanyl and thiophosphoryl derivatives of imines (**IV**). Many similarities can be observed within the members of specific groups; therefore, we will use this classification to discuss the synthesis and the structural properties of diphosphanation products. The presence of the amide, imino(thio)amide, and imino-thioester functional groups was unambiguously established by the observation of the expected characteristic features in the IR, ^13^C NMR spectra, and single-crystal X-ray diffraction (see the Experimental Section in the SI). The ^31^P{^1^H} data of parent diphosphanes and the obtained diphosphanation products are listed in Tables S28 and S29, respectively.

**Scheme 2:**
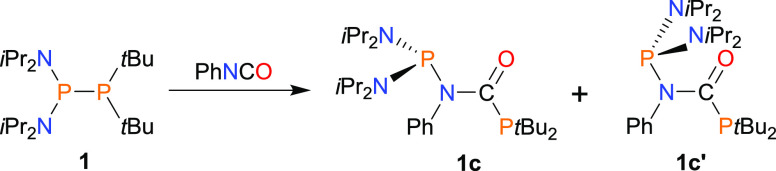
Synthesis of Phosphanyl Derivative of Amide **1c**/**1c**′ (**I**)

The compound of type **I** was obtained via the formal insertion of PhNCO into the P–P bond of diphosphane **1** (group **A**) with the formation of a P–C(=O)–N(−Ph)–P structural motif (Scheme 2). Interestingly, the main product was isolated in high yield (96%) as a mixture of two conformers **1c** and **1c**′ in a molar ratio of 2.5:1, showing very similar NMR spectroscopic properties. According to density functional theory (DFT) calculations, these two conformers of the lowest (and similar) energy result from the two possible orientations of the (*i*Pr_2_N)_2_P moiety in the structure of the product (Scheme 2). By applying variable-temperature (VT)-NMR (298–328 K), we found that the rotation barrier about the respective P–N bond was too high, as we did not observe the conversion of one isomer to the other (see SI Figure S117), presumably due to the bulkiness of the (*i*Pr_2_N)P group. They are not in the dynamic equilibrium and, once formed, do not convert each other. Crystals of **1c**/**1c**′ are prone to hydrolysis and must be handled under an inert atmosphere.

The excess phenyl isocyanate tends to trimerize under reaction conditions, precipitating [PhNCO]_3_ from the reaction mixture as a white solid. Trimer formation was also observed during all other reactions involving unsymmetrical diphosphanes and phenyl isocyanate. The catalytic role of highly nucleophilic molecules in the formation of [RNCO]_3_ was previously reported.^47,48^ Hence, the obtained diphosphanes behave as Lewis base catalysts following the previously described mechanism of cyclotrimerization of isocyanates.^47,48^

In contrast to the reaction of persistent phosphinyl radical (H_2_C)_2_(NDipp)_2_P• with PhNCO, we did not observe the formation of a product with a P–O–C(=NPh)–P structural motif.^43^

**Figure 1:**
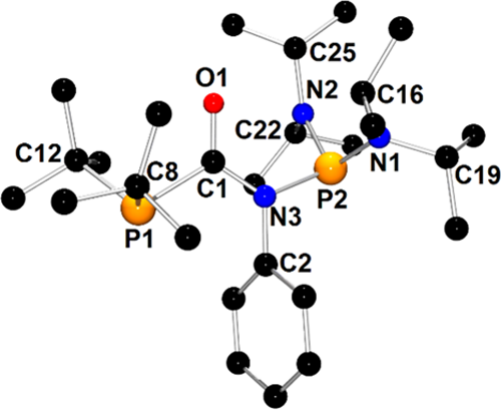
X-ray structure of **1c**′ showing the atom-numbering scheme. The H atoms are omitted for clarity.

The molecular structure of **1c**′ is depicted in Figure 1. The X-ray structure analysis confirms the formal insertion of one PhNCO molecule into the P–P bond of the parent compound **1**. The newly formed compound bears two pyramidal phosphanyl moieties that are linked to the C1(=O1)–N3–Ph amide group via P1–C1 and P2–N3 bonds. The geometry of the amide group in **1c** resembles those observed in classical organic amides.^49^

**Scheme 3:**
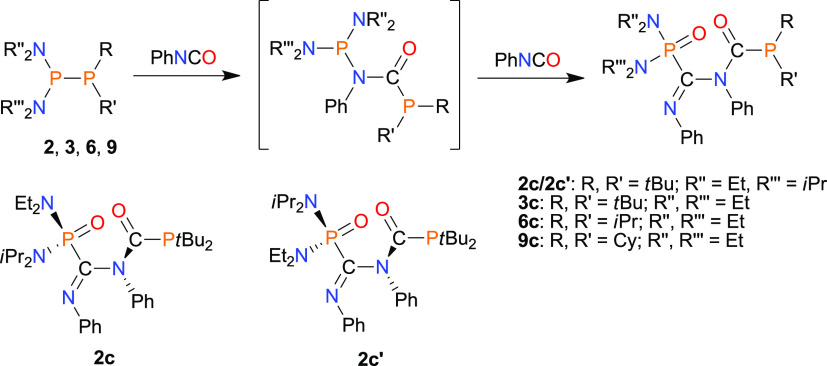
Synthesis of Phosphanyl and Phosphoryl Derivatives of Iminoamides (**II**)

To our surprise, reactions of PhNCO with diphosphanes with less crowded bisaminophosphanyl moieties such as **2**, **3**, **6**, and **9** (groups **B** and **C**) led to products of type **II** (Scheme 3). The obtained compounds **2c**/**2c**′, **3c**, **6c**, and **9c** possessed the iminoamide functional group C(=O)–N(Ph)–C(=NPh), where the first carbon atom is connected to the phosphanyl group RR′P and the second is bound to the phosphoryl moiety (R″N)(R‴N)P=O. The analytically pure products of the insertion of two PhNCO molecules into the P–P bond were isolated by evaporation of solvent as a colorless oil (**2c**/**2c**′, pair of isomers, Scheme 3) or by crystallization from concentrated petroleum ether solution as colorless crystals (**3c**, **6c**, **9c**) in high yields (73–95%). Interestingly, in contrast to **1c**/**1c**′, these products are air- and moisture-stable, both in the solid state and the solution.

**Figure 2:**
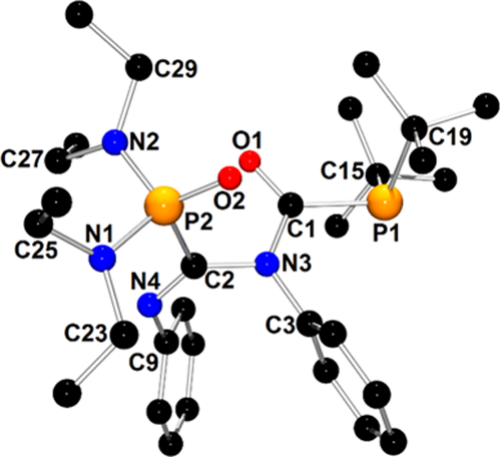
X-ray structure of **3c** showing the atom-numbering scheme. The H atoms are omitted for clarity.

The molecular structure of **3c**, which is representative of compounds of group **II**, is presented in Figure 2. Compounds **6c** and **9c** display very similar solid-state structures to **3c** (Figures S11 and S16, respectively).

In contrast to the structure of **1c**, **3c** consists of phosphanyl and phosphoryl groups linked by an iminoamide moiety C1(=O1)–N3(Ph)–C2(=N4Ph). The metric parameters of the amide C1(=O1)–N3–Ph fragment resemble those observed for **1c**. The planar geometry around the C2 atom and very short C2–N4 distance (1.273(2) Å) were expected for the imino group. The multiple bond character of the P2=O2 bond was confirmed by the respective very short bond distance with a value of 1.475(1) Å. The P1–C1 (1.891(1) Å), P2–C2 (1.854(2) Å), and N3–C2 (1.441(2) Å) distances are typical for single covalent bonds. The rotation about the N3–C2 bond, which connects planar amide and imino groups, is hindered by the spatial orientation C1=O1 and P2=O2 fragments. Interestingly, two atropisomers were found in the asymmetric unit of **6c**, which differed in the values of the C1(29)–N3(7)–C2(30)–P2(4) torsion angles (−71.7(1) vs 79.3(1)°) (Figure S11). These observations suggest that the N3–C2 bond constitutes the axis of chirality. Introducing a second element of chirality to the structure of type **II** compounds should lead to the observation of diastereoisomers in NMR spectra. Indeed, modifying the structure of **3c** by replacing one NEt_2_ group with N*i*Pr_2_ generates a chiral center at the P2 atom, and thus, the pair of diastereoisomers **2c** and **2c**′ was detected by NMR spectroscopy.

To our knowledge, the compounds bearing structures of type **I** and **II** have not been accessible in the free state. There has been only one report on the synthesis of a ligand similar to phosphanyl-substituted amide **1c**/**1c**′; this ligand had a formula of Ph_2_P–C(=O)–N(Et)–PPh_2_ and was formed in the coordination sphere of the transition metal via a reaction between phosphenium complexes [Cp(CO)(HPh_2_P)M(PPh_2_)] (M = Mo, W) and EtNCO.^50^ Furthermore, the formation of oxidized analogues of the aforementioned structures **I** and **II** was postulated based on ^31^P NMR and IR spectroscopy in the reaction of pyrophosphite R_2_POPR′_2_ (R = OBu, NEt_2_, *t*Bu; R′ = OBu, NEt_2_) with PhNCO.^51^

**Scheme 4:**
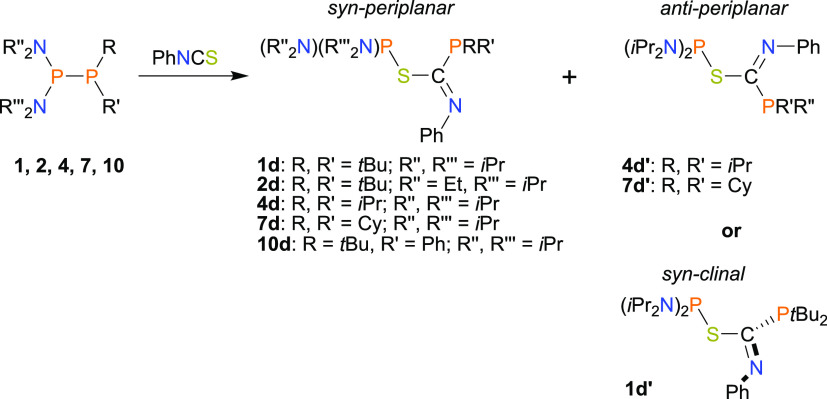
Synthesis of Phosphanyl Derivatives of Thioimino Esters (**III**)

Next, we tested the reactivity of **1–15** toward PhNCS. The analysis of the outcomes of these reactions reveals that unsymmetrical diphosphanes exhibit higher reactivity toward PhNCS than toward PhNCO. In the case of reactions involving diphosphanes **1**, **4**, **7**, **10** (group **A**), and **2** (group **B**), the PhNCS moiety is inserted into the P–P bond with the formation compounds of type **III** (Scheme 4). In contrast to reactions with PhNCO, the products containing P–N bonds are not formed at all. The obtained diphosphanation products have a common P–C(=NPh)–S–P skeleton, which is analogous to those observed for the product of the reaction of persistent phosphinyl radical (H_2_C)_2_(NDipp)_2_P• with PhNCS.^43^ However, to our knowledge, derivatives with two different phosphanyl groups bound to the C(=NPh)–S moiety remain unknown.

The products of the reactions mentioned above were isolated by evaporation of the volatiles leading to analytical pure, air-sensitive yellow oils (**1d**/**1d**′, **2d**, **4d**/**4d**′, **7d**/**7d**′, **10d**). The obtained oily products solidified at −20 °C to afford yellow X-ray quality crystals in almost quantitative yields.

**Figure 3:**
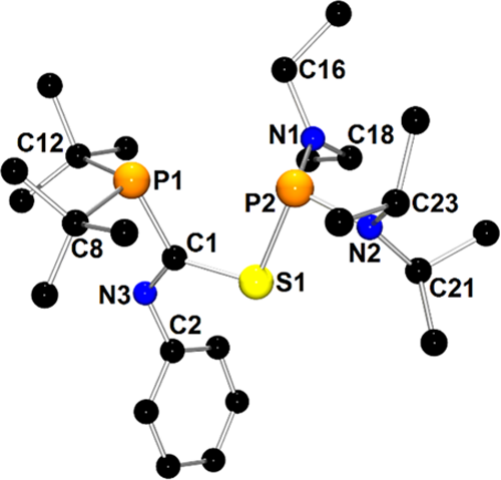
X-ray structure of **2d** showing the atom-numbering scheme. The H atoms are omitted for clarity.

The molecular structure of a group**-III-**type compound (**2d**) is presented in Figure 3. X-ray structures of other members of this group—**1d**/**1d**′, **4d**′, **7d**′, and **10d**—are depicted in Figures S3, S5, S15, and S19, respectively.

**Figure 4:**
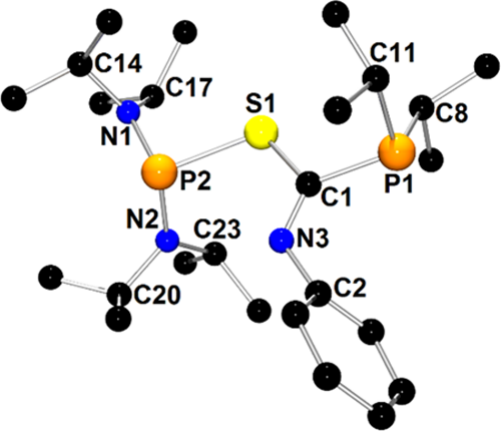
X-ray structure of **4d**′ showing the atom-numbering scheme. The H atoms are omitted for clarity.

In the case of structure **2d**, the PhNCS moiety is bound to *t*Bu_2_P1 and (Et_2_N)(*i*Pr_2_N)P2 phosphanyl groups via C1 and S1 atoms, respectively. The P1 and P2 atoms retained the pyramidal geometry observed for parent diphosphane **2**, whereas the geometry around the C1 atom of the imino group was planar. The P1–C1, S1–C1, and P2–S1 bond distances are typical for single covalent bonds (P1–C1 = 1.861(2) Å, S1–C1 = 1.790(2) Å, P2–S1 = 2.1900(7) Å; ∑*r*_cov_ (P–C) = 1.86 Å, ∑*r*_cov_ (S–C) = 1.78 Å, ∑*r*_cov_ (P–S) = 2.14 Å).^52^ Similar to the bonds of class **II** compounds, the C1–N3 bond within the imino group has a double bond character (C1–N3 = 1.276(2) Å; ∑*r*_cov_(C=N) = 1.27 Å).^53^ Interestingly, reactions involving **1**, **4**, and **7** gave mixtures of two conformers, **1d**/**1d**′, **4d**/**4d**′, and **7d**/**7d**′, which differed in the value of the P–C–S–P torsion angle and, hence, the spatial orientation of phosphanyl groups (Scheme 4). This assumption was confirmed by DFT calculations that involved a scan of the potential energy surface along the P–S–C–P dihedral and VT-NMR (273–353 K) (see SI Figures S118 and S119; Scheme S2). Free rotation about the C–S bond is precluded due to both π-interactions within the C–S confirmed by the natural bond orbital (NBO) and, more importantly, due to the bulkiness of the (*i*Pr_2_N) and PR_2_ groups (P*t*Bu_2_, *Pi*Pr_2_, and PCy_2_, respectively). Once formed, they do not interconvert due to rotation’s high-energy barrier, even at elevated temperatures. Thus, although these two conformers are of the lowest energy, they are not in dynamic equilibrium in the solution, and the **1d/1d**′, **4d/4d**′, and **7d/7d**′ molar ratios are not under thermodynamic control. DFT calculations indicate that the syn-conformer is the lowest-energy conformer of all members of group **III**. Interestingly, the relatively short intramolecular distance P1···P2 was found for syn-periplanar conformers (for representative **2d**: 3.2897(7) Å); this distance is shorter than the sum of van der Waals radii (3.60 Å). Furthermore, second-order perturbation analysis revealed a weak attractive interaction between the P1 and P2 centers, which additionally stabilized the syn-periplanar conformation resulting from the overlapping of the lone electron pair of the P1 atom and the antibonding σ*(P2–N2) orbital with an electron stabilization energy *E*(2) ascribed to this interaction of 3.44 kcal mol^–1^ (**2d**). Moreover, DFT calculations indicated that for compounds of group **III**, except **1d**′, the second-lowest-energy conformer exhibited an antiperiplanar conformation of the phosphanyl groups. An example of such a conformer is **4d**′, which is presented in Figure 4 (P1–C1–S1–P2 torsion angle = −165.5(2)°). The syn- and anti-conformers can be easily distinguished by comparing absolute values of ^3^*J*_P–P_. The syn-conformer display large ^3^*J*_P–P_ values ranging from 109.0 to 130.8 Hz, whereas the anticonformer exhibits relatively small ^3^*J*_P–P_ values (8.0–14.5 Hz), or such coupling is not visible due to the broadness of the signals (see SI Table S29). With decreasing bulkiness of either the (RR′N)_2_P moiety in **2d** or PRR′ moiety in **10d**, the rotation energy barrier decreased as well, and thus, we observed the presence of only one, the lowest-energy conformer.

Interestingly, both conformers **1d** and **1d**′ were found in the asymmetric unit (see SI Figure S3), where the lowest-energy conformer **1d** exhibits a syn-periplanar conformation and the second-lowest-energy conformer **1d**′ displays a syn-clinal conformation with P1–C1–S1–P2 torsion angles of −25.8(3) and −62.6(2)°, respectively. In this case, the antiperiplanar conformation is less energetically favored than the syn-clinal conformation observed in the crystal.

**Scheme 5:**
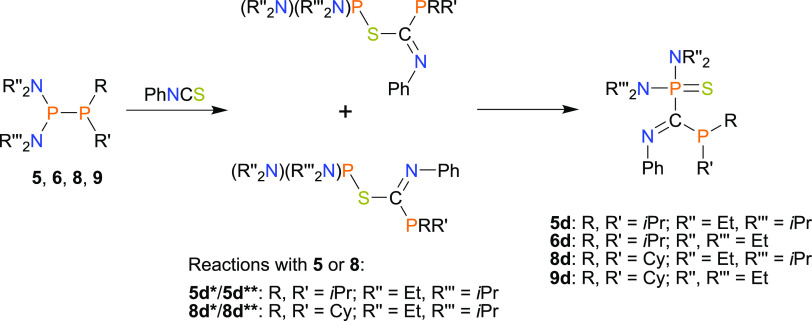
Synthesis of Phosphanyl and Thiophosphoryl Derivatives of Imines (**IV**)

The reactions of diphosphanes attributed to groups **B** and **C** with an excess of PhNCS also yielded diphosphanyl derivatives of imino thioethers (**III**); however, except for **2d**, these compounds tend to rearrange or react with the second equivalent of PhNCS. In particular, in the case of experiments employing diphosphanes bearing *Pi*Pr_2_ (**5**, **6**) or PCy_2_ groups (**8**, **9**), the intermediate imino thioether rearranges to compounds of type **IV**: **5d**, **6d**, and **8d**, **9d**, respectively (Scheme 5, see SI Figures S120–S123).

Interestingly, the reaction of **5** with an excess of PhNCS initially yields a mixture of two conformers of imino thioether 5d*/5d** and imine **5d**, which further slowly transforms to pure **5d** (see SI Figure S120). However, the removal of an excess of heterocumulene leads to the formation of an equilibrium mixture of **5**, 5d*/5d**, and **5d**. An analogous reaction pattern is observed for an experiment involving **8** (see SI Figure S121). Because of the reversible nature of the reactions of **5** and **8** with PhNCS, we were not able to cleanly isolate the products of these reactions, and they were only characterized by NMR spectroscopy. The calculated free energy values of the respective transformations corroborate the presence of equilibrium mixtures of the products for **5d** and **8d** and the formation of stable S-migration products in the case of **6d** and **9d** (see SI Schemes S3–S8).

**Figure 5:**
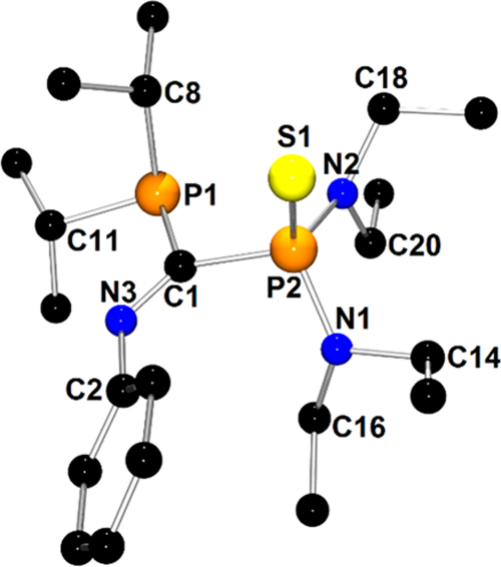
X-ray structure of **6d** showing the atom-numbering scheme. The H atoms are omitted for clarity.

From derivatives of type **IV**, the structures of **6d** and **9d** were confirmed by X-ray diffraction. Their molecular structures are presented in Figures 5 and S17, respectively. In the case of these species, phosphanyl and thiophosphoryl groups are linked by imino groups. The P1, P2, C1, C2, and N3 atoms lie almost at the same plane, which is in accordance with the expected sp^2^ hybridization of both the C1 and N3 atoms. The phosphorus–carbon bond lengths within the P1–C1–P2 fragment fall in the range of single covalent bonds, whereas C1–N3 and P2–S1 distances are typical for double bonds (for representative **6d**: P1–C1 = 1.864(2) Å, P2–C1 = 1.875(2) Å, C1–N3 = 1.279(3) Å, P2–S2 = 1.9573(9) Å). The presented phosphanyl- and thiophosphoryl-substituted imines do not have counterparts in the literature. There is only one example of a compound with a P(III)–C(=NR)–P(V) structural motif; this compound was reported by the Gessner group, and it formed when decomposition of the parent carbenoid compound yielded Ph_2_P–C(=NAd)–P(=NAd)Ph_2_.^54^

**Figure 6:**
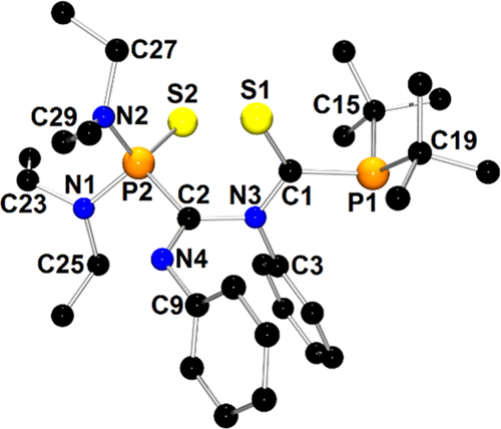
X-ray structure of **3d** showing the atom-numbering scheme. The H atoms are omitted for clarity. One molecule of the two present in the asymmetric unit was selected.

**Scheme 6:**
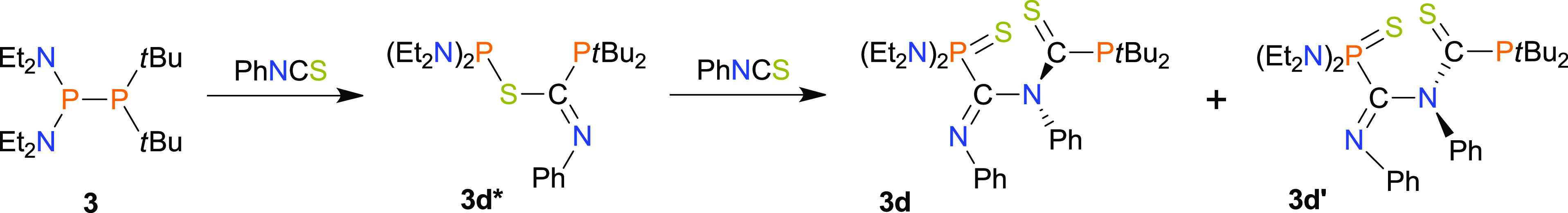
Reactions of **3** with One and Two Equivalents of PhNCS

In contrast to other diphosphanes of group **C**, compound **3** reacts with an excess of thioisocyanate with the formal insertion of two PhNCS molecules into P–P (Scheme 6). The obtained two conformers have a structure analogous to **3c** (type **II**), where sulfur atoms replaced oxygen atoms. Although **3c** and **3d** have analogous structures, elongation of the P–S bond compared to P–O as well as the increased bulkiness of the S-containing derivate results in the presence of two conformers: **3d** and **3d**′. A relaxed scan of potential energy surfaces confirmed that rotation about (E)P–C(=N−Ph) is of the lower-energy barrier for **3c** than for **3d**, which may explain why the latter compound has two rotamers. The structure of **3d** was confirmed by an X-ray single diffraction study (Figure 6). The intermediate imino thioether 3d* can be obtained and isolated via reaction of **3** with one equivalent of PhNCS.

### Mechanistic Study

**Figure 7:**
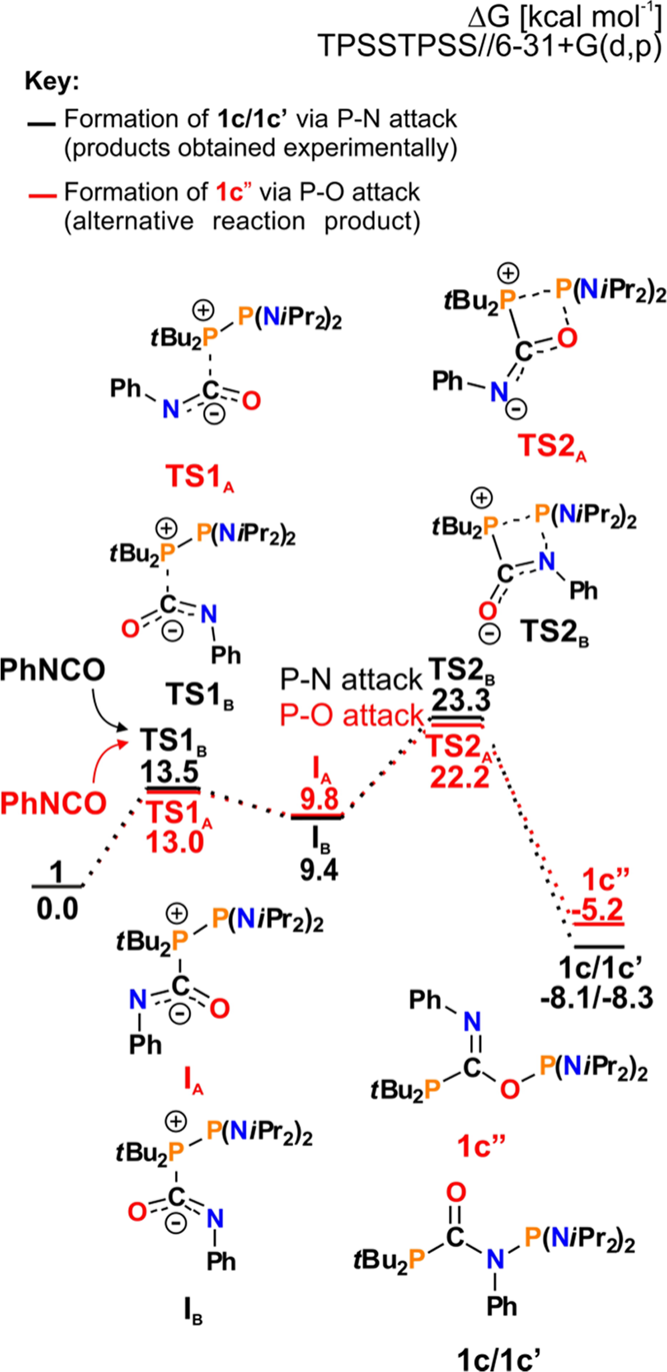
Mechanism of reaction of **1** with PhNCO. The red path represents the alternative product formation (**1c**″), which was not observed experimentally.

Given the presence of two Lewis basic centers, O and N atoms, the formal insertion of a single PhNCO molecule into the P–P bond may lead to the formation of either the (RR′N)_2_P–O–C(=N–Ph)–PR″_2_ or (RR′N)_2_P–N(Ph)–C(=O)–PR″_2_ product. According to the Gibbs free energy profiles, in each case, the reaction of **1** with PhNCO proceeds via a simple two-step mechanism (Figure 7).

**Figure 8:**
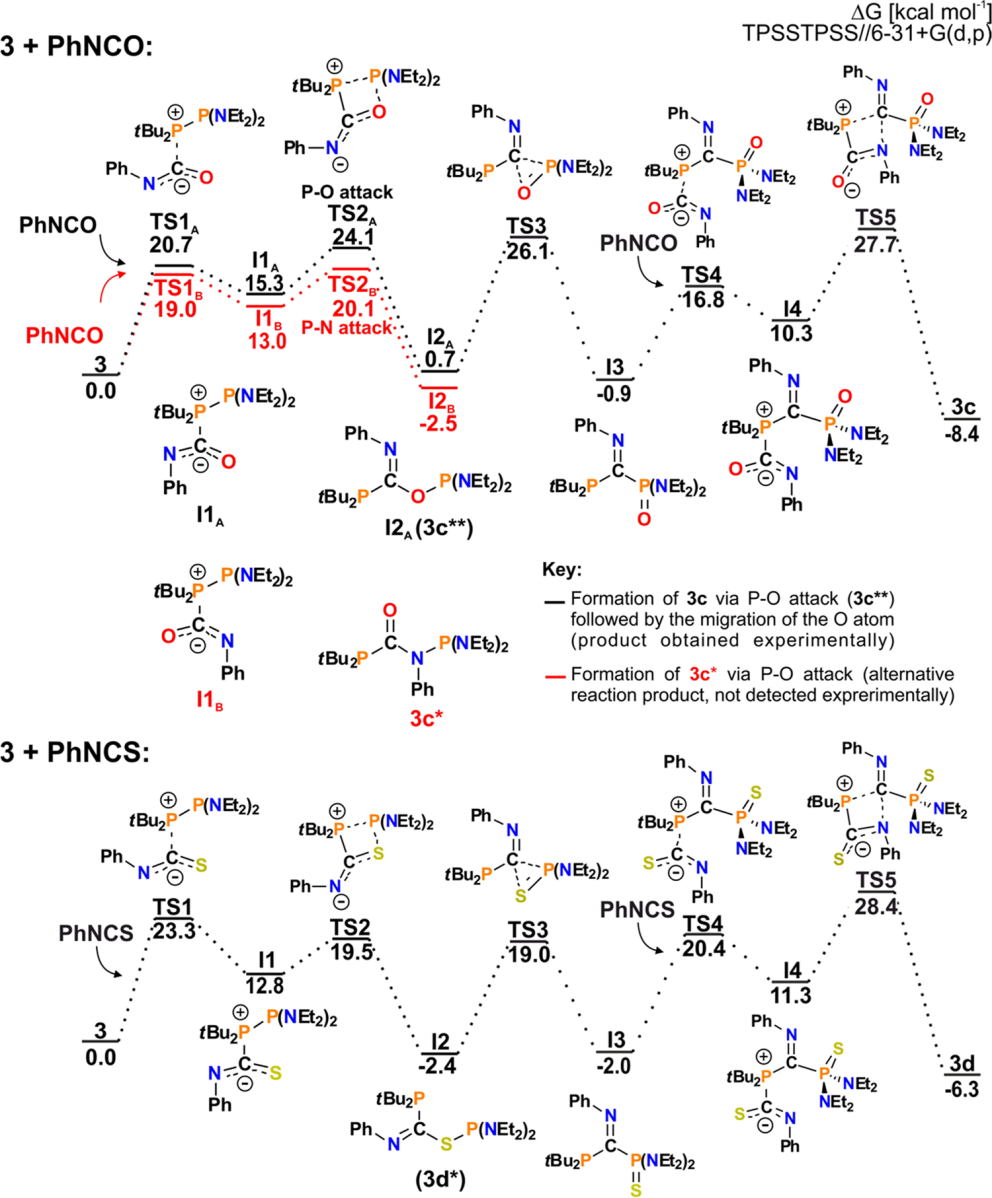
Mechanism of formation of **3c** and **3d**. The red path represents the alternative product formation (3c*), which was not observed experimentally.

The initial step involves the nucleophilic attack of the **P***t*Bu_2_ atom on the PhN**C**O atom to give intermediate **I1**. It is followed by the rate-determining step, going through four-membered PCNP (black path) or PCOP (red path) transition state **TS2**, which finally leads to **1c/1c**′ and **1c**″, respectively. Although the formation of **1c**″ is slightly faster (with an energy barrier of 22.2 kcal mol^–1^ compared to 23.3 kcal mol^–1^ for **1c/1c**′), we did not observe its generation experimentally. The only isolated products were thermodynamically favored conformers **1c** and **1c**′. Decreasing the bulkiness of the (RR′N)_2_P moiety in **2**, **3**, **6**, and **9** enables rearrangement and subsequent binding of the second PhNCO molecule, as presented for **3** (Figure 8). Unlike the reaction of **1**, the formation of (RR′N)_2_P–N(Ph)–C(=O)–PR″_2_ (3c*, red path) in the first step, though it was both thermodynamically and kinetically privileged, was not observed. The (RR′N)_2_P–O–C(=N–Ph)–PR″_2_ derivative (**I2**_A_) in turn leads to even more stable double PhNCO insertion product **3c** (black path). It proceeds via migration of the O atom to the **P**(Et_2_N)_2_ atom with simultaneous formation of a P–C bond in a three-membered POC transition state **TS3** to give **I3**. Subsequent fixation of PhNCO also starts with *t*Bu_2_**P**–**C** nucleophilic attack but is now followed by the formation of the C–N bond in the rate-determining step of the reaction, *via***TS5**, to give **3c** as the final product.

Interestingly, the PhNCS molecule(s) binding by diphosphanes follows an analogous reaction mechanism (Figure 8). Herein, depending on both steric congestion of the (RR′N)_2_P group and nucleophilicity of the **P**R″_2_ counterparts, the reaction leads to the single-PhNCS insertion derivative (RR′N)_2_P–S–C(=N–Ph)–PR″_2_, the S-migration product (RR′N)_2_P(=S)–C(=N–Ph)–PR″_2_ or double PhNCS insertion (RR′N)_2_P(=S)–C(=N–Ph)–N(Ph)–C(=S)–PR″_2_ as the final products of the reaction. Comparing the formation of **3c** and **3d**, migration of the S atom is more kinetically accessible than the migration of the O atom (19.0 and 26.1 kcal mol^–1^, respectively), whereas fixation of the second PhNCE molecule has a slightly greater energy barrier for **3d** (28.4 compared to 27.7 kcal mol^–1^). These trends are reflected in the scope of the obtained products. The formation of (RR′N)_2_P–S–C(=N–Ph)-PR″_2_ and (RR′N)_2_P(=S)–C(=N–Ph)–PR″_2_ species (once thermodynamically accessible) can occur due to the low-energy barrier, resulting in a variety of products. At the same time, only the one bearing small Et_2_N groups and the highly nucleophilic **P***t*Bu_2_ is able to attach a second PhNCS in a kinetically demanding step. Conversely, in the reaction of PhNCO, all steps are of similar, high-energy barrier, and only diphosphanes of the highest nucleophilicity of the **P**R_2_ atom form stable products, differing depending on the steric congestion of the (RR′N)_2_P counterpart.

## 3 Conclusions

**Figure 9:**
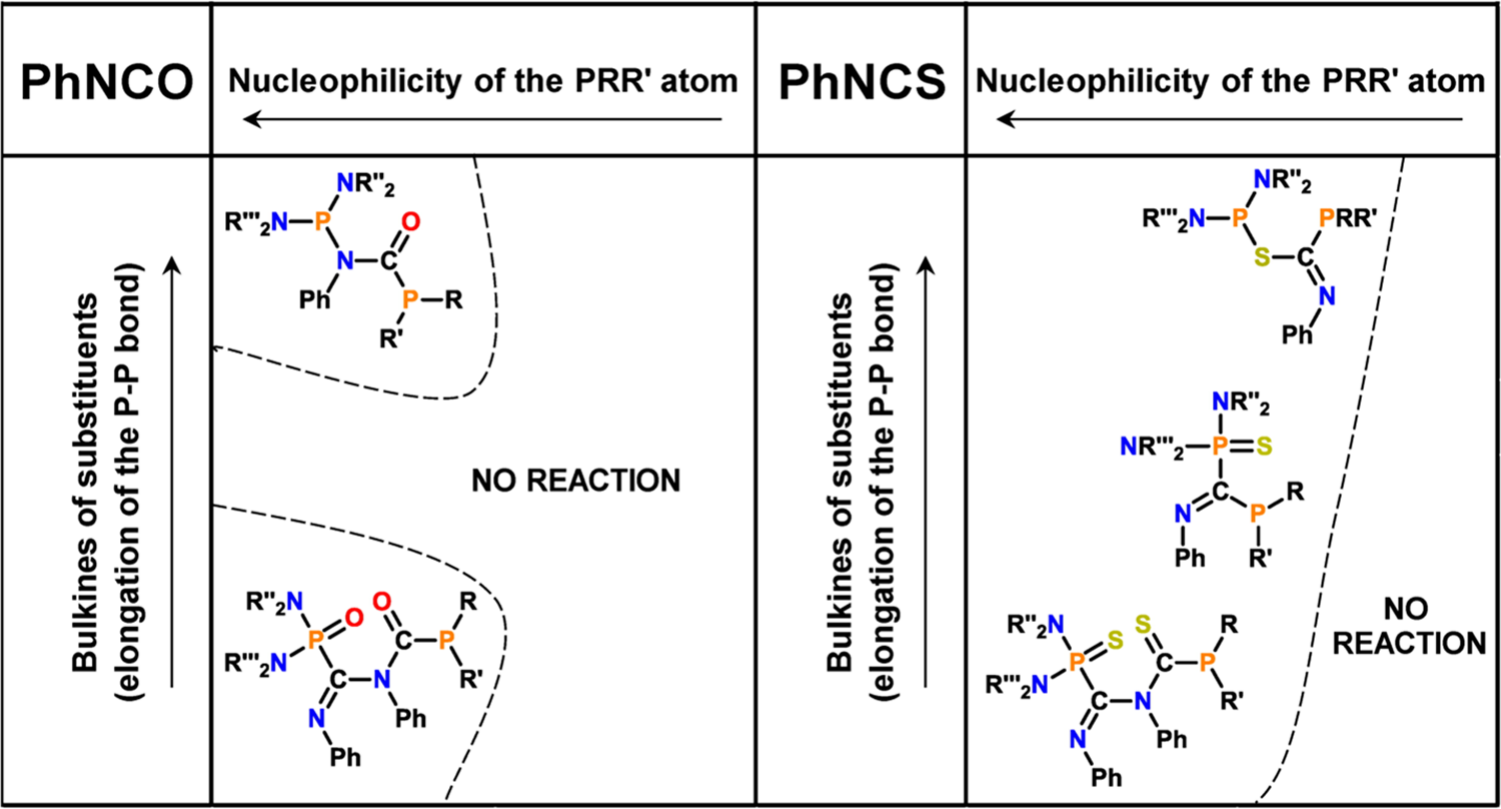
Factors guiding reactivity of 1,1-diaminodiphosphanes toward PhNCO and PhNCS.

Amides and imines have countless applications in organic synthesis, material science, and medicine. We provided synthetic access to their phosphanyl, phosphoryl, or thiophosphoryl derivatives starting from simple building blocks such as unsymmetrical diphosphanes and heterocumulenes. Experimental and theoretical studies have revealed that the outcomes of diphosphanation reactions depend on both the steric and nucleophilic properties of the parent diphosphanes, which in turn affect the kinetic and thermodynamic accessibility of the reactions leading to C=E bond functionalization (Figure 9). Among the group of diphosphanes bearing the bulkiest substituents (*i*Pr_2_N)_2_P, only the most nucleophilic (*i*Pr_2_N)_2_P–P*t*Bu_2_ reacted with phenyl isocyanate yielding the phosphanyl derivatives of amide (**I**). The reactions of PhNCO and diphosphanes with less crowded bisaminophosphanyl moieties (Et_2_N)(*i*Pr_2_N)P and (Et_2_N)_2_P led to double-insertion products classified as phosphanyl and phosphoryl derivatives of iminoamides (**II**). Unsymmetrical diphosphanes substituted with electron-withdrawing phenyl groups did not react with PhNCO. Compared to PhNCO, the unsymmetrical diphosphanes exhibit higher reactivity toward PhNCS. The reactions of diphosphanes with PhNCS yielded phosphanyl derivatives of imino thioesteres (**III**), which, in the case of reactions involving diphosphanes with less crowded phosphanyl groups, tend to rearrange to phosphanyl and thiophosphoryl derivatives of imines (**IV**) or react with the second equivalent of PhNCS to give phosphanyl and thiophosphoryl derivatives of iminothioamides (**II**). Within the group of diphosphanes possessing (*i*Pr_2_N)_2_P moiety, only the least nucleophilic (*i*Pr_2_N)_2_P–PPh_2_ was unreactive toward PhNCS. Similarly, in the case of diphosphanes bearing (Et_2_N)(*i*Pr_2_N)P and (Et_2_N)_2_P fragments, the reactions did not occur only for the diphosphanes containing weakly nucleophilic P*t*BuPPh and PPh_2_ phosphanyl groups. The chemistry of the resulting phosphanyl, phosphoryl, or thiophosphoryl derivatives of amides and imines is unexplored, and their unique structural features, such as the presence of P(III) and P(V) phosphorous centers, open new and exciting possibilities for future applications.

## Supporting Information Available

Crystallographic details, NMR spectroscopic details, and computational details (PDF)

## Accession Codes

CCDC 2068168–2068185 contain the supplementary crystallographic data for this paper. These data can be obtained free of charge via www.ccdc.cam.ac.uk/data_request/cif, or by emailing data_request@ccdc.cam.ac.uk, or by contacting The Cambridge Crystallographic Data Centre, 12 Union Road, Cambridge CB2 1EZ, UK; fax: +44 1223 336033.
