# Supplementary material for: Exploring the Reactivity of Unsymmetrical Diphosphanes toward Heterocumulenes: Access to Phosphanyl and Phosphoryl Derivatives of Amides, Imines, and Iminoamides
Source: Inorg Chem. 2022 Jun 14;61(25):9523–32. doi: 10.1021/acs.inorgchem.2c00589 (PMC9490836; doi:10.1021/acs.inorgchem.2c00589)
Supplement: Supplementary file 1 — ic2c00589_si_001.pdf [file ic2c00589_si_001.pdf]

Supplementary Material for

Exploring the reactivity of unsymmetrical diphosphanes towards heterocumulenes: access to phosphanyl and phosphoryl derivatives of amides, imines, and iminoamides

Natalia Szynkiewicz, Jarosław Chojnacki**

*and Rafał Grubba*ǂ*

ǂ*Corresponding author*

*Department of Inorganic Chemistry, Faculty of Chemistry,

Gdansk University of Technology, G. Narutowicza St. 11/12. PL-80-233, Gdansk, Poland.

CONTENTS:

Experimental section 5

Preparation of 4 5

Preparation of 5 6

Preparation of 6 7

Preparation of 7 8

Preparation of 8 9

Preparation of 9 10

Preparation of 14 11

Formation of 4a 12

Formation of 7a 12

Preparation of $2a_{Pt}$ 13

Preparation of 4b 14

Preparation of 7b 15

Preparation of 10b 16

Formation of 13b 17

Preparation of 1c 18

Preparation of 2c 19

Preparation of 3c 20

Preparation of 6c 21

Preparation of 9c 22

Preparation of 1d 23

Preparation of 2d 24

Preparation of 3d 25

Preparation of 3d* 26

Preparation of 4d 27

Formation of 5d 28

Preparation of 6d 29

Preparation of 7d 30

Formation of 8d 31

Preparation of 9d 32

Preparation of 10d 33

Reactivity of diphosphanes towards CO_2 and CS_2 34

X-ray structures analysis 36

General methods 36

Specific details for individual structures 36
Single crystal X-ray structure analysis of 1c 42
Single crystal X-ray structure analysis of 1d and 1d' 43
Single crystal X-ray structure analysis of $2a_{Pt}$ 44
Single crystal X-ray structure analysis of 2d 45
Single crystal X-ray structure analysis of 3c 46
Single crystal X-ray structure analysis of 3d 47
Single crystal X-ray structure analysis of 4 48
Single crystal X-ray structure analysis of 4b 49
Single crystal X-ray structure analysis of 4d 50
Single crystal X-ray structure analysis of 6c 51
Single crystal X-ray structure analysis of 6d 52
Single crystal X-ray structure analysis of 7 53
Single crystal X-ray structure analysis of 7b 54
Single crystal X-ray structure analysis of 7d 55
Single crystal X-ray structure analysis of 9c 56
Single crystal X-ray structure analysis of 9d 57
Single crystal X-ray structure analysis of 10b 58
Single crystal X-ray structure analysis of 10d 59
Spectroscopic data 60
NMR spectra of isolated compounds 60
NMR spectra of 4 60
NMR spectra of 5 62
NMR spectra of 6 63
NMR spectra of 7 65
NMR spectra of 8 66
NMR spectra of 9 68
NMR spectra of 14 69
NMR spectra of $2a_{Pt}$ 71
NMR spectra of 4b 72
NMR spectra of 7b 74
NMR spectra of 10b 75
NMR spectra of 1c and 1c' 77
NMR spectra of 2c 78
NMR spectra of 3c 80
NMR spectra of 6c 81

NMR spectra of 9c 83
NMR spectra of 1d and 1d' 84
NMR spectra of 2d 86
NMR spectra of 3d and 3d' 87
NMR spectra of 3d* 89
NMR spectra of 4d and 4d' 90
NMR spectra of 5d 92
NMR spectra of 6d 93
NMR spectra of 7d and 7d' 95
NMR spectra of 8d 96
NMR spectra of 9d 98
NMR spectra of 10d 99
IR spectra of isolated compounds 101
NMR studies of selected reactions 109
Variable temperature NMR study of selected compounds 109
Monitoring of reaction progress by $^{31}P\{^{1}H\}$ NMR of selected reactions 111
DFT calculations 115
General methods 115
Philicity of reactive centres 119
Values of free energy of formation of considered products 120
NBO analysis 123
Optimized structures and Cartesian coordinates 125
References 126

Experimental section

All manipulations were carried out under a dry argon atmosphere using flame-dried Schlenk-type glassware on a vacuum line or in a glove-box. In the reactions with gaseous reagents, the toluene solution of the substrate was slowly frozen in a liquid nitrogen bath, evacuated to 0.01 Torr, and backfilled with CO_2 (1 atm). Then, the cooling bath was removed, and the reaction mixture was allowed to warm to room temperature. During thawing of the reaction mixture, the Schlenk flask was opened to a line connected with a pressure-relief system to avoid overpressure in the vessel. Solvents were dried by standard procedures over Na(K)/K/Na/benzophenone and distilled under argon. 1D (^{31}P, ^{13}C, ^{11}B and ^{1}H) and 2D NMR spectra in C_6D_6 or toluene-d_8 solution were recorded on a Bruker AV400 MHz spectrometer (external standard TMS for ^{1}H and ^{13}C; 85% H_3PO_4 for ^{31}P) at an ambient or lower temperature. Reaction progress was monitored by $^{31}P\{^{1}H\}$ and ^{31}P spectra of reaction mixtures. The FTIR spectra of crystalline products were recorded using a Nicolet iS50 FT-IR spectrometer equipped with the Specac Quest single-reflection diamond attenuated total reflectance (ATR) accessory. Spectral analysis was carried out by using the OMNIC software package. Elemental analyses were performed using a Elementar's Vario El Cube CHNS micro elemental analyzer. The basic principle of quantitative CHNS analysis is the high-temperature, oxidative combustion of samples. The gaseous products of combustion are purified, separated in absorption columns into individual components (nitrogen, carbon dioxide, sulphur dioxide, water vapour) and detected in the measuring cell of a TCD detector (thermal conductivity detector). Both elemental analyses and IR spectra were not listed either for oil products (**5**, **6**, **9**, **2c**/**2c'**, **3d***, **6d** – only IR spectrum; we do not have access to equipment allowing elemental analysis of oils) or products that were not isolated as pure compounds: intermediates **5d*/5d**** and **8d*/8d**** or thermodynamically unstable **4a**, **7a**, **13b**, **5d**, and **8d**. Diphosphanes **1**, **3**, **10**, **11**, **12**, **13** and **15** were synthesized *via* the procedure described in [1] and [2] for **2**.

Preparation of 4

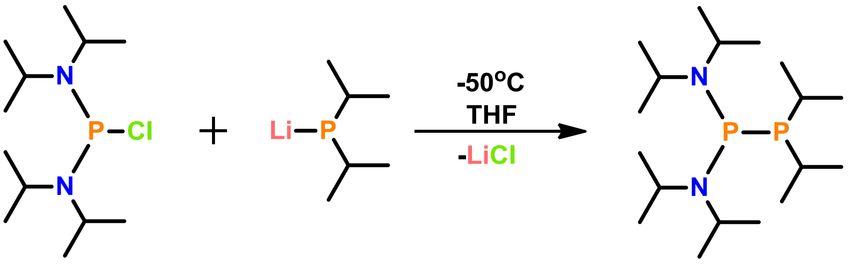

4

To a solution of iPr$_2$PLi (0.620 g, 5.00 mmol) in 40 cm^3 of THF cooled to -50°C, a solution of (iPr$_2$N)$_2$PCl (1.334 g, 5.00 mmol) in 5 cm^3 of THF was added dropwise. The reaction mixture was stirred at -50°C for 30 minutes and then allowed to warm to room temperature for further 30 minutes. The solvent was evaporated, and the residue was dried under a vacuum (0.01 mmHg) for 30 minutes at 50°C to remove all volatiles. The crude product was dissolved in 10 cm^3 of petroleum ether and filtered. The solution was concentrated and left at -30°C to afford X-ray quality crystals of **4**. The crystalline product was separated and dried under vacuum (0.01 Torr), giving **4** as analytically pure white solid. Yield 82% (1.430 g, 4.103 mmol).

NMR:

$^{31}P\{^1H\}$ NMR (C_6D_6, 298K): δ 63.9 (d, $^1J_{PP}$ =146.7 Hz, P(iPr$_2$N)$_2$), -14.0 (d, $^1J_{PP}$ =146.7 Hz, PiPr$_2$).

^1H NMR (C_6D_6, 298K): δ 3.69 (m, 4H, NC*H*CH$_3$), 2.13 (m, 2H, C*H*CH$_3$), 1.48 (d, 3H, $^3J_{HH}$ = 6.6 Hz, CHC*H$_3$*), 1.46 (d, 3H, $^3J_{HH}$ = 6.5 Hz, CHC*H$_3$*), 1.44 (dd, 3H, $^3J_{HH}$ = 7.3 Hz, $^4J_{HH}$ = 1.5 Hz, CHC*H$_3$*), 1.40 (dd, 3H, $^3J_{PH}$ = 7.6 Hz, $^4J_{HH}$ = 1.5 Hz, CHC*H$_3$*), 1.23 (d, 12H, $^3J_{HH}$ = 6.7 Hz, NCHC*H$_3$*), 1.15 (d, 12H, $^3J_{HH}$ = 6.7 Hz, NCHC*H$_3$*).

$^{13}C\{^1H\}$ NMR (C_6D_6, 298K): δ 48.7 (d, $^2J_{PC}$ = 8.1 Hz, N*C*HCH$_3$), 48.6 (d, $^2J_{PC}$ = 8.1 Hz, N*C*HCH$_3$), 24.8 (d, $^3J_{PC}$ = 6.6 Hz, NCH*CH$_3$*), 24.6 (d, $^3J_{PC}$ = 6.6 Hz, NCH*CH$_3$*), 24.0 (s, CH*CH$_3$*), 23.9 (d, $^1J_{PC}$ = 15.3 Hz, *C*HCH$_3$), 23.7 (s, CH*CH$_3$*), 23.6 (d, $^1J_{PC}$ = 18.3 Hz, *C*HCH$_3$), 22.0 (s, CH*CH$_3$*), 21.9 (s, CH*CH$_3$*).

Elemental analysis: calcd. for $C_{18}H_{42}N_2P_2$: C, 62.04; H, 12.15; N, 8.04 Found: C, 61.99; H, 12.031; N, 8.06.

Preparation of 5

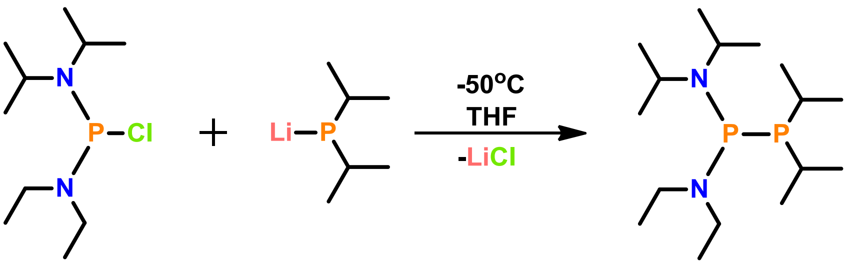

5

To a solution of iPr$_2$PLi (0.797 g, 6.421 mmol) in 40 cm^3 of THF cooled to -50°C, (Et$_2$N)(iPr$_2$N)PCl (1.533 g, 6.421 mmol) was added dropwise. The reaction mixture was stirred at -50°C for 30 minutes and then allowed to warm to room temperature for further 30 minutes. The solvent was evaporated, and the residue was dried under a vacuum (0.01 mmHg) for 30 minutes at 50°C to remove all volatiles. The crude product was dissolved in 10 cm^3 of petroleum ether and filtered. Removal of the solvent under vacuum afforded 1.945 g (6.070 mmol) of **5** as a yellowish oil in 95% yield.

NMR:

$^{31}P\{^1H\}$ NMR (C_6D_6, 298K): δ 83.1 (d, $^1J_{PP}$ =145.3 Hz, P(Et$_2$N)(iPr$_2$N)), -19.0 (d, $^1J_{PP}$ =145.3 Hz, PiPr$_2$).

^1H NMR (C_6D_6, 298K): δ 3.55 (broad m, 2H, NC*H*CH$_3$), 3.31 (m, 2H, C*H$_2$*CH$_3$), 3.12 (m, 2H, C*H$_2$*CH$_3$), 2.06 (m, 2H, C*H*CH$_3$), 1.38-1.30 (overlapped dublets, 12H, CHC*H$_3$*), 1.16 (d, 6H, $^3J_{HH}$ = 6.0 Hz, NCHC*H$_3$*), 1.08 (d, 6H, $^3J_{HH}$ = 6.6 Hz, NCHC*H$_3$*), 1.03 (t, 6H, $^3J_{HH}$ = 7.1 Hz, CH$_2$C*H$_3$*).

$^{13}C\{^1H\}$ NMR (C_6D_6, 298K): δ 47.5 (broad, N*C*HCH$_3$), 44.9 (d, $^2J_{PC}$ = 8.8 Hz, *CH$_2$*CH$_3$), 44.7 (d, $^2J_{PC}$ = 8.8 Hz, *CH$_2$*CH$_3$), 24.2 (broad s, NCH*CH$_3$*), 23.6 (dd, $^2J_{PC}$ = 23.5 Hz, $^3J_{PC}$ = 5.1 Hz, CH*CH$_3$*), 23.0 (dd, $^1J_{PC}$ = 19.8 Hz, $^2J_{PC}$ = 16.1 Hz, *C*HCH$_3$), 22.9 (dd, $^2J_{PC}$ = 13.2 Hz, $^3J_{PC}$ = 8.1 Hz, CH*CH$_3$*), 22.2 (dd, $^2J_{PC}$ = 13.2 Hz, $^3J_{PC}$ = 5.9 Hz, CH*CH$_3$*), 22.1 (dd, $^1J_{PC}$ = 21.3 Hz, $^2J_{PC}$ = 17.6 Hz, *C*HCH$_3$), 20.2 (d, $^3J_{PC}$ = 9.5 Hz, CH*CH$_3$*) 14.6 (d, $^3J_{PC}$ = 3.7 Hz, *CH$_2$*CH$_3$).

Preparation of 6

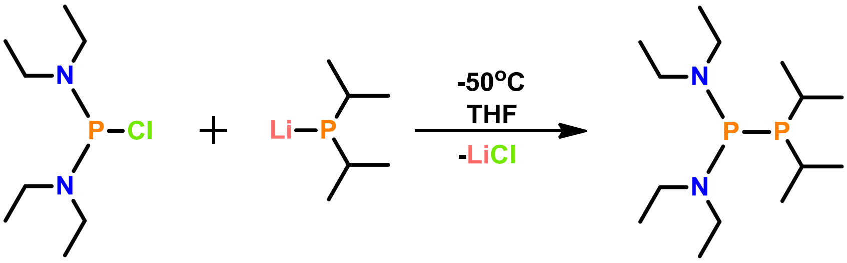

6

To a solution of *i*Pr_2PLi (0.671 g, 5.411 mmol) in 40 cm^3 of THF cooled to -50°C, $(Et_2N)_2PCl$ (1.140 g, 5.411 mmol) was added dropwise. The reaction mixture was stirred at -50°C for 30 minutes and then allowed to warm to room temperature for a further 30 minutes. The solvent was evaporated, and the residue was dried under vacuum (0.01 mmHg) for 30 minutes at 50°C to remove all volatiles. The crude product was dissolved in 10 cm^3 of petroleum ether and filtered. Removal of the solvent under vacuum afforded 1.391 g (4.757 mmol) of **6** as a yellowish oil in 88% yield.

NMR:

$^{31}P\{^1H\}$ NMR (C_6D_6, 298K): δ 106.6 (d, $^1J_{PP}$ =152.6 Hz, $P(Et_2N)_2$), -19.0 (d, $^1J_{PP}$ =152.6 Hz, P*i*Pr_2).

^1H NMR (C_6D_6, 298K): δ 2.98 (m, 4H, C$H_2$$CH_3$), 2.87 (m, 4H, C$H_2$$CH_3$), 1.78 (m, 2H, C*H*$CH_3$), 1.06 (d, 3H, $^3J_{HH}$ = 7.2 Hz, CHCH_3), 1.05 (d, 3H, $^3J_{HH}$ = 6.8 Hz, CHCH_3), 1.03 (d, 3H, $^3J_{HH}$ = 7.1 Hz, CHCH_3), 1.02 (d, 3H, $^3J_{HH}$ = 7.3 Hz, CHCH_3), 0.75 (t, 12H, $^3J_{HH}$ = 7.1 Hz, CH_2CH_3).

$^{13}C\{^1H\}$ NMR (C_6D_6, 298K): δ 44.8 (d, $^2J_{PC}$ = 8.2 Hz, $CH_2$$CH_3$), 44.7 (d, $^2J_{PC}$ = 8.2 Hz, $CH_2$$CH_3$), 22.6 (d, $^1J_{PC}$ = 16.3 Hz, *CH*CH_3), 22.4 (d, $^1J_{PC}$ = 16.3 Hz, *CH*CH_3), 22.4 (d, $^2J_{PC}$ = 6.4 Hz, CHCH_3), 22.2 (d, $^2J_{PC}$ = 6.4 Hz, CHCH_3), 21.4 (d, $^2J_{PC}$ = 8.2 Hz, CHCH_3), 21.3 (d, $^2J_{PC}$ = 8.2 Hz, CHCH_3), 14.4 (d, $^3J_{PC}$ = 3.6 Hz, $CH_2$$CH_3$).

Preparation of 7

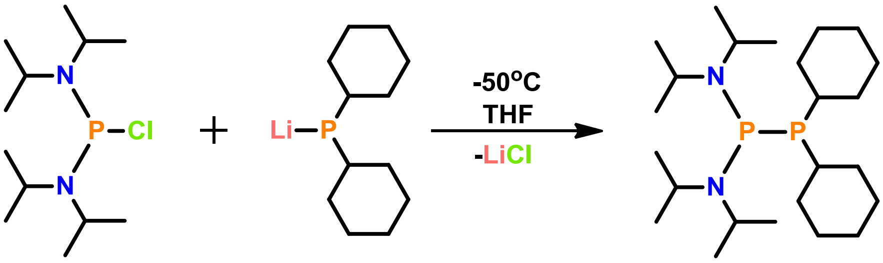

7

To a solution of Cy_2PLi (1.021 g, 5.00 mmol) in 40 cm^3 of THF cooled to -50°C, a solution of $(iPr_2N)_2PCl$ (1.334 g, 5.00 mmol) in 5 cm^3 of THF was added dropwise. The reaction mixture was stirred at -50°C for 30 minutes and then allowed to warm to room temperature for further 30 minutes. The solvent was evaporated, and the residue was dried under a vacuum (0.01 mmHg) for 30 minutes at 50°C to remove all volatiles. The crude product was dissolved in 10 cm^3 of petroleum ether and filtered. The solution was concentrated and left at -30°C to afford X-ray quality crystals of **7**. The crystalline product was separated and dried under vacuum (0.01 Torr), giving **7** as analytically pure white solid. Yield 87% (1.875 g, 4.375 mmol).

NMR:

$^{31}P\{^1H\}$ NMR (C_6D_6, 298K): δ 60.3 (d, $^1J_{PP}$ =138.9 Hz, $P(iPr_2N)_2$), -18.7 (d, $^1J_{PP}$ =138.9 Hz, PCy_2).

^1H NMR (C_6D_6, 298K): δ 3.74 (m, 4H, NC*H*CH_3), 2.43 (m, 2H, CH_2), 2.18 (m, 2H, C*H*), 1.91-1.59 (overlapped m, 12H, CH_2), 1.36-1.16 (overlapped m, 8H, CH_2), 1.25 (d, 6H, $^3J_{HH}$ = 6.7 Hz, CH_3), 1.20 (d, 6H, $^3J_{HH}$ = 6.6 Hz, CH_3).

$^{13}C\{^1H\}$ NMR (C_6D_6, 298K): δ 48.6 (d, $^2J_{PC}$ = 8.8 Hz, N*CH*CH_3), 48.5 (d, $^2J_{PC}$ = 8.8 Hz, N*CH*CH_3), 35.0 (d, $^1J_{PC}$ = 15.4 Hz, *C*H), 34.8 (d, $^2J_{PC}$ = 4.4 Hz, *C*H_2), 34.7 (d, $^1J_{PC}$ = 15.4 Hz, *C*H), 34.6 (d, $^2J_{PC}$ = 4.4 Hz, *C*H_2), 32.73 (d, $^2J_{PC}$ = 8.8 Hz, *C*H_2), 32.72 (d, $^2J_{PC}$ = 8.8 Hz, *C*H_2), 29.0 (d, $^3J_{PC}$ = 14.7 Hz, *C*H_2), 28.9 (d, $^3J_{PC}$ = 14.7 Hz, *C*H_2), 28.5 (s, *C*H_2), 28.4 (s, *C*H_2), 26.7 (s, *C*H_2), 24.8 (d, $^3J_{PC}$ = 6.6 Hz, *C*H_3), 24.7 (d, $^3J_{PC}$ = 6.6 Hz, *C*H_3).

Elemental analysis: calcd. for $C_{24}H_{50}N_2P_2$: C, 62.04; H, 12.15; N, 8.04 Found: C, 61.99; H, 12.031; N, 8.06.

Preparation of 8

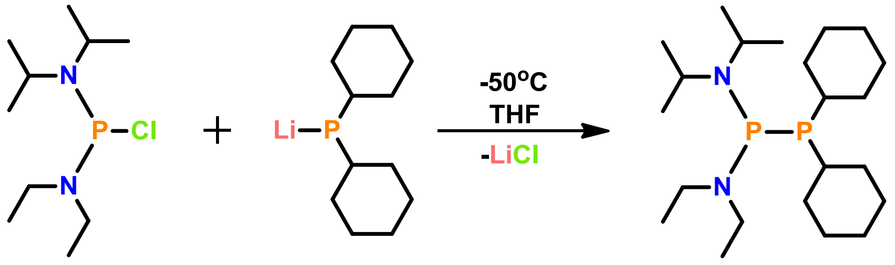

8

To a solution of Cy_2PLi (0.900 g, 4.406 mmol) in 40 cm^3 of THF cooled to -50°C, a solution of $(Et_2N)(iPr_2N)_2PCl$ (1.052 g, 4.406 mmol, 1 cm^3) in 5 cm^3 of THF was added dropwise. The reaction mixture was stirred at -50°C for 30 minutes and then allowed to warm to room temperature for further 30 minutes. The solvent was evaporated, and the residue was dried under a vacuum (0.01 mmHg) for 30 minutes at 50°C to remove all volatiles. The crude product was dissolved in 10 cm^3 of petroleum ether and filtered. The solution was concentrated and left at -80°C to afford white crystals of **8**. The crystalline product was separated and dried under vacuum (0.01 Torr), giving **8** as analytically pure white solid. Yield 89% (1.564 g, 3.905 mmol).

NMR:

$^{31}P\{^1H\}$ NMR (C_6D_6, 298K): δ 79.8 (d, $^1J_{PP}$ =138.1 Hz, $P(Et_2N)(iPr_2N)$), -25.0 (d, $^1J_{PP}$ =138.1 Hz, PCy_2).

^1H NMR (C_6D_6, 298K): δ 3.57 (m, 2H, $NCHCH_3$), 3.34 (m, 2H, NCH_2CH_3), 3.13 (m, 2H, NCH_2CH_3), 2.36-2.21 (m, 2H, CH_2), 2.21-213 (m, 2H, CH), 1.94-1.52 (overlapped m, 12H, CH_2), 1.37-1.21 (overlapped m, 6H, CH_2), 1.18-1.13 (overlapped m, 12H, $CHCH_3$), 1.07 (d, 6H, $^3J_{HH}$ = 7.1 Hz, CH_2CH_3).

$^{13}C\{^1H\}$ NMR (C_6D_6, 298K): δ 47.5 (broad m, $NCHCH_3$), 44.8 (d, $^2J_{PC}$ = 9.1 Hz, NCH_2CH_3), 44.7 (d, $^2J_{PC}$ = 9.1 Hz, NCH_2CH_3), 34.3 (dd, $^2J_{PC}$ = 22.7 Hz, $^3J_{PC}$ = 5.4 Hz, CH_2), 34.0 (dd, $^1J_{PC}$ = 20.0 Hz, $^2J_{PC}$ = 15.4 Hz, CH), 33.4 (dd, $^1J_{PC}$ = 21.8 Hz, $^2J_{PC}$ = 15.4 Hz, CH), 33.3 (dd, $^2J_{PC}$ = 12.7 Hz, $^3J_{PC}$ = 8.2 Hz, CH_2), 32.4 (dd, $^2J_{PC}$ = 10.9 Hz, $^3J_{PC}$ = 6.4 Hz, CH_2), 31.2 (d, $^3J_{PC}$ = 8.2 Hz, CH_2), 28.7 (s, CH_2), 28.6 (s, CH_2), 28.2 (dd, $^2J_{PC}$ = 15.4 Hz, $^3J_{PC}$ = 9.1 Hz, CH_2), 28.1 (s, CH_2), 26.7 (s, CH_2), 26.5 (s, CH_2), 24.2 (broad s, $CHCH_3$), 14.7 (d, $^3J_{PC}$ = 2.7 Hz, CH_2CH_3).

Elemental analysis: calcd. for $C_{22}H_{46}N_2P_2$: C, 65.97; H, 11.58; N, 6.99. Found: C, 65.81; H, 11.551; N, 7.07.

Preparation of 9

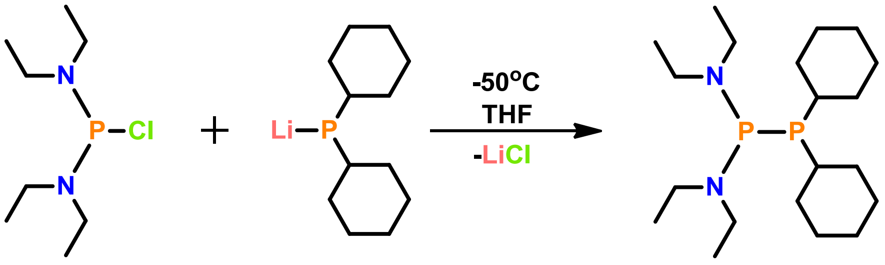

9

To a solution of Cy_2PLi (1.063 g, 5.207 mmol) in 40 cm^3 of THF cooled to -50°C, $(Et_2N)_2PCl$ (1.097 g, 5.207 mmol) was added dropwise. The reaction mixture was stirred at -50°C for 30 minutes and then allowed to warm to room temperature for further 30 minutes. The solvent was evaporated, and the residue was dried under a vacuum (0.01 mmHg) for 30 minutes at 50°C to remove all volatiles. The crude product was dissolved in 10 cm^3 of petroleum ether and filtered. Removal of the solvent under vacuum afforded 1.750 g (4.698 mmol) of **9** as an orange oil in 90% yield.

NMR:

$^{31}P\{^1H\}$ NMR (C_6D_6, 298K): δ 104.8 (d, $^1J_{PP}$ =152.6 Hz, $P(Et_2N)_2$), -25.9 (d, $^1J_{PP}$ =152.6 Hz, PCy_2).

^1H NMR (C_6D_6, 298K): δ 3.27 (m, 4H, $NC\mathit{H_2}CH_3$), 3.16 (m, 4H, $NC\mathit{H_2}CH_3$), 2.21 (m, 2H, $C\mathit{H_2}$), 2.14 (m, 2H, $C\mathit{H_2}$), 1.90 (m, 2H, $C\mathit{H}$), 1.78 (m, 4H, $C\mathit{H_2}$), 1.65 (m, 2H, $C\mathit{H_2}$), 1.54 (m, 4H, $C\mathit{H_2}$), 1.36-1.16 (overlapped m, 6H, $C\mathit{H_2}$), 1.04 (d, 12H, $^3J_{HH}$ = 7.0 Hz, $CH_2C\mathit{H_3}$).

$^{13}C\{^1H\}$ NMR (C_6D_6, 298K): δ 44.8 (d, $^2J_{PC}$ = 8.2 Hz, $N\mathit{CH_2}CH_3$), 44.7 (d, $^2J_{PC}$ = 8.2 Hz, $N\mathit{CH_2}CH_3$), 33.4 (dd, $^1J_{PC}$ = 20.0 Hz, $^2J_{PC}$ = 15.4 Hz, $\mathit{C}H$), 32.8 (dd, $^2J_{PC}$ = 14.5 Hz, $^3J_{PC}$ = 6.4 Hz, $\mathit{C}H_2$), 32.1 (dd, $^2J_{PC}$ = 8.2 Hz, $^3J_{PC}$ = 8.2 Hz, $\mathit{C}H_2$), 28.3 (d, $^3J_{PC}$ = 10.9 Hz, $\mathit{C}H_2$), 28.0 (d, $^3J_{PC}$ = 7.3 Hz, $\mathit{C}H_2$), 26.6 (s, $\mathit{C}H_2$), 14.6 (s, $NCH_2\mathit{CH_3}$), 14.5 (s, $NCH_2\mathit{CH_3}$).

Preparation of 14

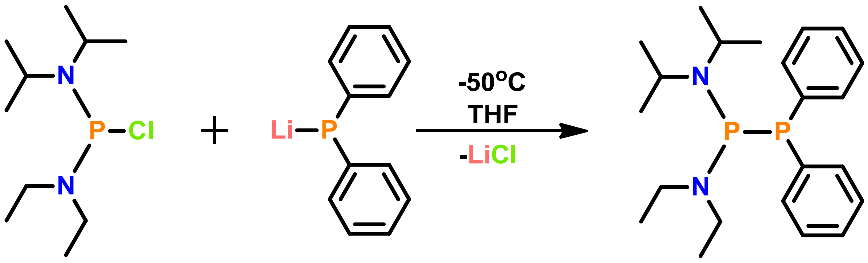

14

To a solution of Ph_2PLi (0.843 g, 4.390 mmol) in 40 cm^3 of THF cooled to -50°C, a solution of $(Et_2N)(iPr_2N)_2PCl$ (1.048 g, 4.390 mmol) in 5 cm^3 of THF was added dropwise. The reaction mixture was stirred at -50°C for 30 minutes and then allowed to warm to room temperature for further 30 minutes. The solvent was evaporated, and the residue was dried under a vacuum (0.01 mmHg) for 30 minutes at 50°C to remove all volatiles. The crude product was dissolved in 10 cm^3 of petroleum ether and filtered. Removal of the solvent under vacuum afforded 1.545 g (3.977 mmol) of **14** as a white solid in 91% yield.

NMR:

$^{31}P\{^1H\}$ NMR (C_6D_6): δ 88.5 (d, $^1J_{PP}$ = 123.5 Hz, $P(Et_2N)(iPr_2N)$), -40.4 (d, $^1J_{PP}$ = 123.5 Hz, PPh_2).

^1H NMR (C_6D_6): δ 7.9 (m, 4H, o-CH), 7.12-7.01 (m, 6H, m,p-CH), 3.63 (broad, 2H, NCH), 3.27 (m, 2H, NCH_2), 3.10 (m, 2H, NCH_2), 1.15 (d, $^3J_{HH}$ = 6.6 Hz, 6H, $CHCH_3$), 0.83 (d, $^3J_{HH}$ = 6.6 Hz, 6H, $CHCH_3$), 0.82 (t, $^3J_{HH}$ = 6.7 Hz, 6H, CH_2CH_3).

$^{13}C\{^1H\}$ NMR (C_6D_6): δ 138.4 (dd, $^1J_{PC}$ = 17.6 Hz, $^2J_{PC}$ = 16.9 Hz, *ipso*-C), 137.4 (dd, $^1J_{PC}$ = 16.9 Hz, $^2J_{PC}$ = 16.9 Hz, *ipso*-C), 136.2 (dd, $^2J_{PC}$ = 19.8 Hz, $^3J_{PC}$ = 8.1 Hz, *ortho*-CH), 135.0 (dd, $^2J_{PC}$ = 19.1 Hz, $^3J_{PC}$ = 8.1 Hz, *ortho*-CH), 128.6 (s, *para*-CH), 128.1 (s, *para*-CH), 127.9 (d, $^3J_{PC}$ = 4.9 Hz, *meta*-CH), 127.8 (d, $^3J_{PC}$ = 5.9 Hz, *meta*-CH), 48.0 (broad m, N*C*H), 44.5 (dd, $^2J_{PC}$ = 15.4 Hz, $^3J_{PC}$ = 8.8 Hz, NCH_2), 24.0 (broad d, $^3J_{PC}$ = 5.1 Hz, $CHCH_3$), 23.7 (broad d, $^3J_{PC}$ = 4.4 Hz, $CHCH_3$) 14.3 (d, $^3J_{PC}$ = 3.7 Hz, CH_2CH_3).

Elemental analysis: calcd. for $C_{22}H_{34}N_2P_2$: C, 68.02; H, 8.82; N, 7.21. Found: C, 67.74; H, 8.700; N, 7.04.

Formation of 4a

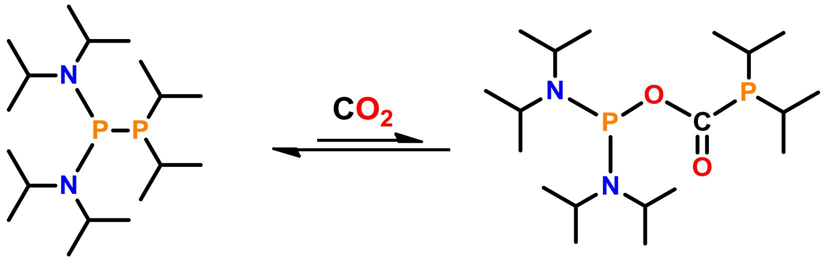

4 **4a**

A solution of **4** (87 mg, 0.250 mmol) in toluene (4 mL) was slowly frozen in a liquid nitrogen bath, evacuated to 0.01 Torr and backfilled with CO_2 (1 atm). The solution was allowed to warm to room temperature and stirred for 24 hours. $^{31}P\{^1H\}$ of the colourless reaction mixture revealed establishing of equilibrium between **4** and **4a** (in 4:1 molar ratio). Product was not isolated as the formation of **4a** is reversible in the absence of CO_2 – under argon atmosphere present in the reaction mixture **4a** regenerates to the parent diphosphane **4**.

NMR :

$^{31}P\{^1H\}$ NMR (C_6D_6, 298K): δ 114.4 (d, $^3J_{PP}$ =14.5 Hz, P(*i*Pr$_2$N)$_2$), 20.9 (d, $^3J_{PP}$ =14.5 Hz, P*i*Pr$_2$).

Formation of 7a

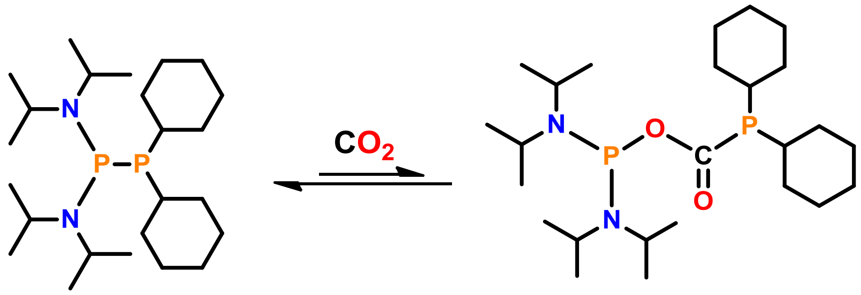

7 **7a**

A solution of **7** (107 mg, 0.250 mmol) in toluene (4 mL) was slowly frozen in a liquid nitrogen bath, evacuated to 0.01 Torr and backfilled with CO_2 (1 atm). The solution was allowed to warm to room temperature and stirred for 24 hours. $^{31}P\{^1H\}$ of the colourless reaction mixture revealed establishing of equilibrium between **7** and **7a** (in 4:1 molar ratio). Product was not isolated as the formation of **7a** is reversible in the absence of CO_2 – under argon atmosphere present in the reaction mixture **7a** regenerates to the parent diphosphane **7**.

NMR :

$^{31}P\{^1H\}$ NMR (C_6D_6, 298K): δ 114.3 (d, $^3J_{PP}$ =14.5 Hz, P(*i*Pr$_2$N)$_2$), 12.6 (d, $^3J_{PP}$ =14.5 Hz, P*i*Pr$_2$).

Preparation of $2a_{Pt}$

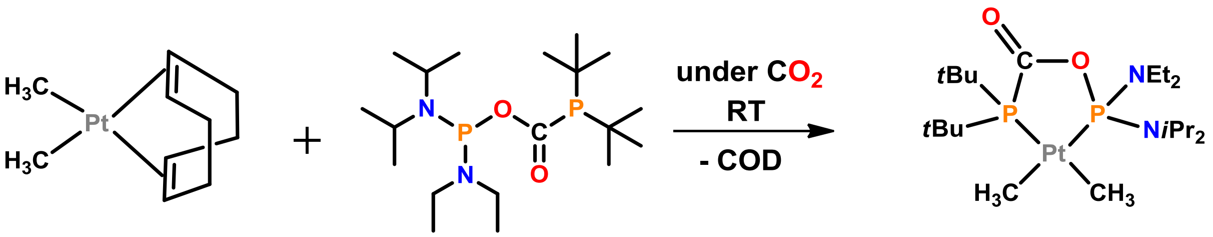

To a solution of **2a** (151 mg, 0.385 mmol) in 4 cm^3 of toluene at room temperature and under CO_2 atmosphere, a solution of (1,5-COD)$PtMe_2$ (128 mg, 0.385 mmol) in 1 cm^3 of toluene was added. The reaction mixture was kept stirring under these conditions, and the $^{31}P\{1H\}$ NMR spectra recorder after two days revealed the complete conversion of substrates into $\mathbf{2a_{Pt}}$. The solvent was evaporated, and the residue was dried under a vacuum (0.01 mmHg) at 50°C to remove all volatiles. The crude oily product was dissolved in 5 cm^3 of petroleum ether and left at -20^{0}C to afford colourless X-ray quality crystals of $\mathbf{2a_{Pt}}$. Yield 72% (172 mg, 0.278 mmol).

NMR:

$^{31}P\{^1H\}$ NMR (C_6D_6): δ = 132.1 (d, $^2J_{PP}$ = 21.8 Hz, $^1J_{PPt}$ = 2659.4 Hz, P(Et_2N)($i$$Pr_2$N)) , 86.4 (d, $^2J_{PP}$ = 21.8 Hz, $^1J_{PPt}$ = 1707.5 Hz, P$t$$Bu_2$).

^1H NMR (C_6D_6): δ 4.03 (sept, $^3J_{HH}$ = 6.8 Hz, 2H, *CH*CH_3), 3.32 (m, 2H, CH_2CH_3), 2.90 (m, 2H, CH_2CH_3), 1.38-1.24 (two overlapped m, 6H, PtCH_3), 1.36 (d, $^3J_{PH}$ = 13.8 Hz, 9H, C(CH_3)$_3$), 1.31 (d, $^3J_{PH}$ = 14.0 Hz, 9H, C(CH_3)$_3$), 1.16 (d, $^3J_{HH}$ = 6.8 Hz, 6H, CHCH_3), 1.13 (d, $^3J_{HH}$ = 6.8 Hz, 6H, CHCH_3), 0.93 (d, $^3J_{HH}$ = 7.0 Hz, 12H, CHCH_3).

$^{13}C\{^1H\}$ NMR (C_6D_6): δ 175.8 (dd, $^1J_{PC}$ = 33.7 Hz, $^2J_{PC}$ = 24.2 Hz, *C*=O), 47.4 (d, $^2J_{PC}$ = 11.0 Hz, $^3J_{CPt}$ = 13.2 Hz, *CH*CH_3), 39.2 (d, $^2J_{PC}$ = 9.5 Hz, $^3J_{CPt}$ = 13.2 Hz, CH_2CH_3), 37.2 (dd, $^1J_{PC}$ = 8.8 Hz, $^3J_{PC}$ = 2.9 Hz, $^2J_{CPt}$ = 24.9 Hz, *C*(CH_3)$_3$), 37.0 (dd, $^1J_{PC}$ = 10.3 Hz, $^3J_{PC}$ = 2.2 Hz, $^2J_{CPt}$ = 26.8 Hz, *C*(CH_3)$_3$), 29.6 (d, $^2J_{PC}$ = 5.1 Hz, $^3J_{CPt}$ = 10.3 Hz C(CH_3)$_3$), 29.4 (d, $^2J_{PC}$ = 5.9 Hz, $^3J_{CPt}$ = 11.0 Hz C(CH_3)$_3$), 24.1 (d, $^3J_{PC}$ = 3.7 Hz, CHCH_3), 23.7 (d, $^3J_{PC}$ = 2.9 Hz, CHCH_3), 13.4 (d, $^3J_{PC}$ = 2.2 Hz, CH_2CH_3), 2.3 (dd, $^2J_{PC}$ = 95.0 Hz, $^2J_{PC}$ = 7.3 Hz, $^1J_{CPt}$ = 605.6 Hz, PtCH_3), -0.3 (dd, $^2J_{PC}$ = 133.9 Hz, $^1J_{PC}$ = 7.3 Hz, $^2J_{CPt}$ = 577.7 Hz, PtCH_3).

Elemental analysis: calcd. for $C_{21}H_{48}N_2O_2P_2Pt$: C, 40.84; H, 7.83; N, 4.54. Found: C, 40.70; H, 7.642; N, 4.51.

IR (solid): $\tilde{\upsilon}$ = 2969, 2932, 2872, 1715 (**C=O**), 1464, 1408, 1366, 1196, 1176, 1126, 1083, 1014, 1003, 987, 934, 916, 883, 852, 811, 795, 776, 672, 651, 612, 594, 561, 534, 505, 465, 418.

Preparation of 4b

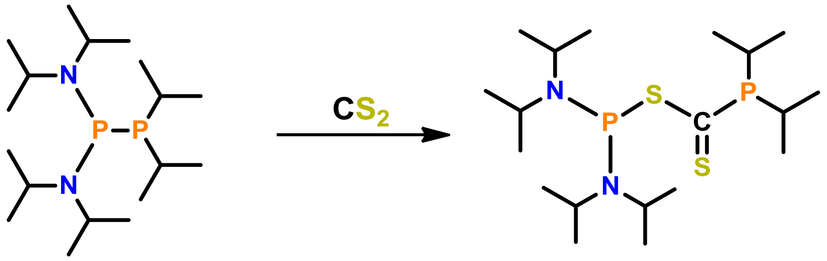

4 4b

To a solution of **4** (87 mg, 0.250 mmol) in toluene (4 mL) an excess of CS_2 (0.1 mL, 126 mg, 1.655 mmol) was added dropwise at room temperature. The solution was stirred for 24 hours. $^{31}P\{^1H\}$ NMR of the deep red reaction mixture revealed the complete conversion of **4** into **4b**. The solvent was evaporated, and the residue was dried under vacuum (0.01 Torr) at 50°C giving **4d** as a red solid. Yield 98% (104 mg, 0.245 mmol). X-ray quality crystals (deep red blocks) were grown from a petroleum solution at -20°C.

NMR :

$^{31}P\{^1H\}$ NMR (C_6D_6, 298K): δ 108.5 (d, $^3J_{PP}$ =14.5 Hz, P(*i*Pr$_2$N)$_2$), 63.2 (d, $^3J_{PP}$ =14.5 Hz, P*i*Pr$_2$).

^1H NMR (C_6D_6, 298K): δ 3.41 (m, 4H, NC*H*CH$_3$), 2.41 (m, 2H, C*H*CH$_3$), 1.25-1.18 (overlapped m, 12H, CH*CH$_3$*), 1.24 (d, 12H, $^3J_{HH}$ = 6.6 Hz, NCHC*H$_3$*), 1.08 (d, 12H, $^3J_{HH}$ = 6.7 Hz, NCHC*H$_3$*).

$^{13}C\{^1H\}$ NMR (C_6D_6, 298K): δ 249.1 (dd, $^1J_{PC}$ = 59.0 Hz, $^2J_{PC}$ = 18.2 Hz, *C*=S), 49.0 (d, $^2J_{PC}$ = 12.7 Hz, N*CH*CH$_3$), 26.72 (d, $^1J_{PC}$ = 19.1 Hz, *CH*CH$_3$), 26.70 (d, $^1J_{PC}$ = 19.1 Hz, *CH*CH$_3$), 23.8 (d, $^3J_{PC}$ = 4.5 Hz, NCH*CH$_3$*), 23.4 (d, $^3J_{PC}$ = 9.1 Hz, NCH*CH$_3$*), 19.7 (d, $^2J_{PC}$ = 13.6 Hz, CH*CH$_3$*), 19.2 (d, $^2J_{PC}$ = 13.6 Hz, CH*CH$_3$*).

Elemental analysis: calcd. for $C_{19}H_{42}N_2P_2S_2$: C, 53.74; H, 9.97; N, 6.60; S, 15.10. Found: C, 53.62; H, 9.801; N, 6.60; S, 15.154.

IR (solid): $\tilde{\upsilon}$ = 2965, 2926, 2866, 1456 (**C=S**), 1380, 1363, 1195, 1175, 1154, 1115, 1084, 1047, 1019, 949, 867, 816, 660, 531, 511, 436.

Preparation of 7b

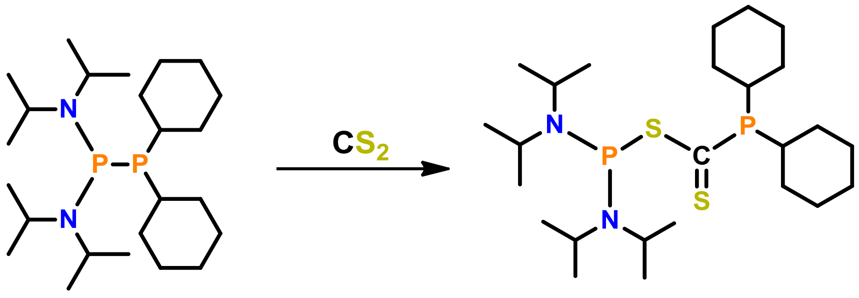

7 **7b**

To a solution of **7** (107 mg, 0.250 mmol) in toluene (4 mL) an excess of CS_2 (0.1 mL, 126 mg, 1.655 mmol) was added dropwise at room temperature. The solution was stirred for 24 hours. $^{31}P\{^1H\}$ NMR of the deep red reaction mixture revealed the complete conversion of **7** into **7b**. The solvent was evaporated, and the residue was dried under vacuum (0.01 Torr) at 50°C to remove all volatiles. The oily red product was dissolved in 3 cm^3 of petroleum ether and left at -20°C to afford deep red X-ray quality crystals of **7b**, which were dried in a vacuum. Yield 87% (110 mg, 0.218 mmol).

NMR :

$^{31}P\{^1H\}$ NMR (C_6D_6, 298K): δ 108.3 (d, $^3J_{PP}$ =14.5 Hz, P(iPr$_2$N)$_2$), 56.0 (d, $^3J_{PP}$ =14.5 Hz, PCy_2).

^1H NMR (C_6D_6, 298K): δ 3.43 (m, 4H, NCHCH$_3$), 2.38 (m, 2H, CH), 2.11 (m, 2H, CH_2), 2.02 (m, 2H, CH_2), 1.71-1.43 (m, 12H, CH_2), 1.32-1.13 (overlapped m, 4H, CH), 1.27 (d, 12H, $^3J_{HH}$ = 6.7 Hz, CHCH_3), 1.08 (d, 12H, $^3J_{HH}$ = 6.7 Hz, CHCH_3).

$^{13}C\{^1H\}$ NMR (C_6D_6, 298K): δ 248.9 (dd, $^1J_{PC}$ = 59.0 Hz, $^2J_{PC}$ = 18.2 Hz, C=S), 49.0 (d, $^2J_{PC}$ = 12.7 Hz, NCHCH$_3$), 36.7 (d, $^1J_{PC}$ = 20.0, $^3J_{PC}$ = 1.8, Hz, CH), 30.2 (d, $^2J_{PC}$ = 11.8 Hz, CH$_2$), 29.4 (d, $^2J_{PC}$ = 11.8 Hz, CH$_2$), 27.5 (d, $^3J_{PC}$ = 10.0 Hz, CH$_2$), 27.2 (d, $^3J_{PC}$ = 10.0 Hz, CH$_2$), 26.5 (s, CH$_2$), 23.8 (d, $^3J_{PC}$ = 5.5 Hz, CH$_3$), 23.4 (d, $^3J_{PC}$ = 9.1 Hz, CH$_3$).

Elemental analysis: calcd. for $C_{25}H_{50}N_2P_2S_2$: C, 59.49; H, 9.98; N, 5.55; S, 12.71. Found: C, 59.40; H, 10.004; N, 5.53; S, 12.648.

IR (solid): $\tilde{\upsilon}$ = 2966, 2922, 2847, 1447 (**C=S**), 1382, 1361, 1195, 1174, 1155, 1116, 1043, 1030, 998, 962, 949, 865, 840, 532, 509.

Preparation of 10b

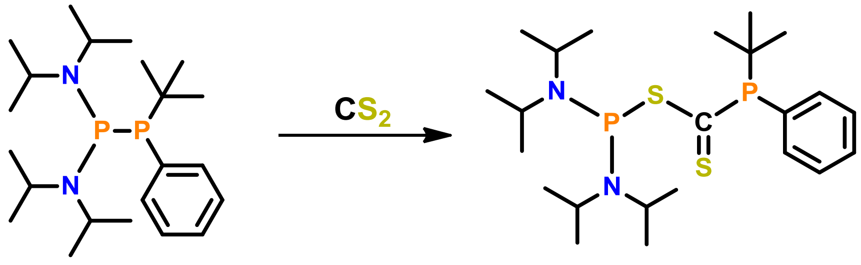

10 **10b**

To a solution of **10** (99 mg, 0.250 mmol) in toluene (4 mL) an excess of CS_2 (0.1 mL, 126 mg, 1.655 mmol) was added dropwise at room temperature. The solution was stirred for 24 hours. $^{31}P\{^1H\}$ NMR of the deep-red reaction mixture revealed the complete conversion of **10** into **10b**. The solvent was evaporated, and the residue was dried under a vacuum (0.01 Torr) at 50°C to remove all volatiles. The oily red product slowly solidifies at -20°C to afford deep red X-ray quality crystals of **10b**. Yield 95% (112 mg, 0.237 mmol).

NMR:

$^{31}P\{^1H\}$ NMR (C_6D_6): δ 109.7 (d, $^3J_{PP}$ = 14.5 Hz, P(*i*Pr$_2$N)$_2$), 67.3 (d, $^3J_{PP}$ = 14.5 Hz, P*t*BuPh).

^1H NMR (C_6D_6): δ 7.78 (m, 2H, o-C*H*), 7.14-7.09 (m, 3H, m,p-C*H*), 3.32 (m, 4H, C*H*), 1.34 (d, $^3J_{PH}$ = 13.0, 9H, C(C*H$_3$*)$_3$), 1.18 (d, $^3J_{HH}$ = 6.6 Hz, 6H, CHC*H$_3$*), 1.16 (d, $^3J_{HH}$ = 6.6 Hz, 6H, CHC*H$_3$*), 1.04 (d, $^3J_{HH}$ = 6.8 Hz, 6H, CHC*H$_3$*), 1.02 (d, $^3J_{HH}$ = 6.8 Hz, 6H, CHC*H$_3$*).

$^{13}C\{^1H\}$ NMR (C_6D_6): δ 248.3 (dd, $^1J_{PC}$ = 58.1 Hz, $^2J_{PC}$ = 19.1 Hz, *C*=S), 137.0 (d, $^1J_{PC}$ = 19.1 Hz, *ipso*-C), 136.7 (d, $^2J_{PC}$ = 20.9 Hz, *ortho*-CH), 129.5 (d, $^3J_{PC}$ = 1.8 Hz, *meta*-CH), 127.8 (s, *para*-CH), 49.0 (d, $^2J_{PC}$ = 12.7 Hz, CH), 48.9 (d, $^2J_{PC}$ = 12.7 Hz, CH), 34.9 (d, $^1J_{PC}$ = 19.1 Hz, *C*(CH$_3$)$_3$), 27.9 (d, $^2J_{PC}$ = 13.6 Hz, C(*CH$_3$*)$_3$), 23.8 (d, $^3J_{PC}$ = 4.5 Hz, CH*CH$_3$*), 23.7 (d, $^3J_{PC}$ = 4.5 Hz, CH*CH$_3$*), 23.3 (d, $^3J_{PC}$ = 3.6 Hz, CH*CH$_3$*), 23.2 (d, $^3J_{PC}$ = 3.6 Hz, CH*CH$_3$*).

Elemental analysis: calcd. for $C_{23}H_{42}N_2P_2S_2$: C, 58.44; H, 8.96; N, 5.93; S, 13.57. Found: C, 58.48; H, 8.895; N, 5.90; S, 12.786.

IR (solid): $\tilde{\upsilon}$ = 2965, 2925, 2864, 1456 (**C=S**), 1434, 1380, 1362, 1344, 1309, 1195, 1173, 1151, 1116, 1096, 1035, 1018, 951, 867, 818, 742, 696, 511, 484, 430.

Formation of 13b

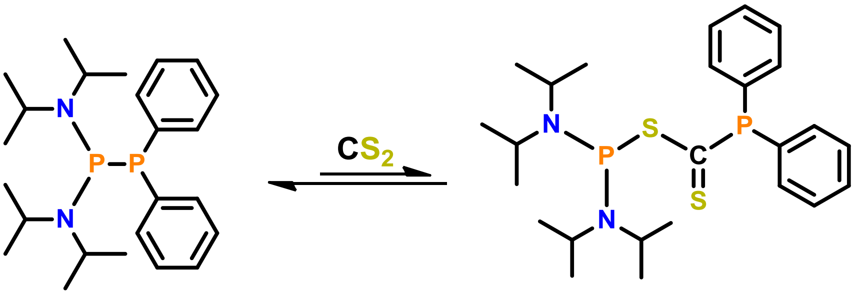

13 **13b**

To a solution of **13** (104 mg, 0.250 mmol) in toluene (4 mL) an excess of CS_2 (0.1 mL, 126 mg, 1.655 mmol) was added dropwise at room temperature. The solution was stirred for 24 hours. $^{31}P\{^1H\}$ NMR of the deep red reaction mixture revealed establishing of equilibrium between **13** and **13b** (in 4:1 molar ratio). Product was not isolated as the formation of **13b** is reversible in the absence of CS_2 – after evaporation of CS_2 **13b** present in the reaction mixture regenerates to parent diphosphane **13**.

NMR:

$^{31}P\{^1H\}$ NMR (C_6D_6): δ 111.8 (d, $^3J_{PP}$ = 14.5 Hz, P(*i*Pr$_2$N)$_2$), 42.4 (d, $^3J_{PP}$ = 14.5 Hz, PPh$_2$).

Preparation of 1c

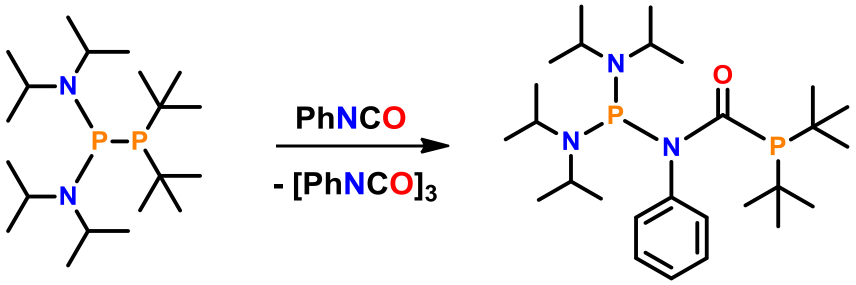

1 **1c/1c'**

To a solution of **1** (94 mg, 0.250 mmol) in THF (4 mL), an excess of PhNCO (0.5 mL, 548 mg, 4.600 mmol) was added at room temperature. The solution was stirred for 26 days yielding white precipitate. $^{31}P\{^{1}H\}$ NMR of the yellowish reaction mixture (suspension) revealed the complete conversion of **1** into a mixture of two isomers **1c** and **1c'** with simultaneous formation of **[PhNCO]$_3$**. The solvent was evaporated, and the residue was dried under a vacuum (0.01 Torr) at 50°C to remove all volatiles. The mixture of **1c**, **1c'** and **[PhNCO]$_3$** was dissolved in 10 cm^3 of pentane and filtered to separate products: **[PhNCO]$_3$** as white solid, which was dried in vacuum (492 mg, 1.377 mmol) and solution of **1c** and **1c'**. The solvent was evaporated, giving **1c/1c'** as a colourless oil which slowly solidifies at -20°C to afford colourless X-ray quality crystals. Yield 96% (119 mg, 0.240 mmol). The isolated product contains conformational isomers **1c** and **1c'** in a molar ratio of 2.5:1

NMR:

$^{31}P\{^{1}H\}$ NMR (C_6D_6): **1c**: δ 115.5 (broad s, P(*i*Pr$_2$N)$_2$), 29.1 (broad s, P*t*Bu$_2$). **1c'**: δ 103.3 (d, $^{3}J_{PP}$ = 14.5 Hz, P(*i*Pr$_2$N)$_2$), 37.3 (d, $^{3}J_{PP}$ = 14.5 Hz, P*t*Bu$_2$).

^{1}H NMR (C_6D_6): **1c**: δ 7.17 (m, 2H, *o*-CH), 7.01-6.97 (overlapped m, 2H, *m*-CH), 6.88 (m, 1H, *p*-CH), 3.55 (broad m, 4H, C*H*), 1.34 (d, $^{3}J_{PH}$ = 11.7, 18H, C(C*H$_3$*)$_3$), 1.22 (d, $^{3}J_{HH}$ = 6.7 Hz, 12H, CHC*H$_3$*), 1.21 (d, $^{3}J_{HH}$ = 6.7 Hz, 12H, CHC*H$_3$*). **1c'**: δ 7.45 (m, 2H, *o*-CH), 7.12 (m, 2H, *m*-CH), 6.99 (overlapped m, 1H, *p*-CH), 3.79 (sept, $^{3}J_{HH}$ = 6.7 Hz, 4H, C*H*), 1.33 (d, $^{3}J_{PH}$ = 11.5, 18H, C(C*H$_3$*)$_3$), 1.22 (d, $^{3}J_{HH}$ = 6.7 Hz, 12H, CHC*H$_3$*), 1.21 (d, $^{3}J_{HH}$ = 6.7 Hz, 12H, CHC*H$_3$*).

$^{13}C\{^{1}H\}$ NMR (C_6D_6): 1c: δ* 148.9 (d, $^{2}J_{PC}$ = 10.9 Hz, *ipso*-C), 128.3 (s, *ortho*-C), 122.3 (s, *para*-CH), 121.4 (s, *meta*-CH), 46.0 (d, $^{2}J_{PC}$ = 15.4 Hz, CH), 32.5 (d, $^{1}J_{PC}$ = 26.3 Hz, *C*(CH$_3$)$_3$), 30.3 (d, $^{2}J_{PC}$ = 14.5 Hz, C(*CH$_3$*)$_3$), 24.6 (d, $^{3}J_{PC}$ = 6.4 Hz, CH*CH$_3$*), 23.6 (d, $^{3}J_{PC}$ = 7.3 Hz, CH*CH$_3$*). *The tertiary carbon atom C=O of isomer **1c** was not detected in the $^{13}C\{1H\}$ NMR. **1c':** δ 180.1 (d, $^{1}J_{PC}$ = 41.8 Hz, *C*=O), 143.7 (dd, $^{2}J_{PC}$ = 15.4 Hz, $^{3}J_{PC}$ = 6.4 Hz, *ipso*-C), 132.4 (dd, $^{3}J_{PC}$ = 6.4 Hz, $^{4}J_{PC}$ = 4.5 Hz, *ortho*-C), 127.8 (s, *meta*-CH), 126.3 (s, *para*-CH), 48.3 (d, $^{2}J_{PC}$ = 16.3 Hz, CH), 33.7 (d, $^{1}J_{PC}$ = 26.3 Hz, *C*(CH$_3$)$_3$), 30.6 (d, $^{2}J_{PC}$ = 15.4 Hz, C(*CH$_3$*)$_3$), 24.2 (d, $^{3}J_{PC}$ = 8.2 Hz, CH*CH$_3$*), 24.0 (d, $^{3}J_{PC}$ = 8.2 Hz, CH*CH$_3$*).

Elemental analysis: calcd. for $C_{27}H_{51}N_3OP_2$: C, 65.43; H, 10.37; N, 8.48; S, 13.57. Found: C, 65.38; H, 10.353; N, 8.50.

IR (solid): $\tilde{\upsilon}$ = 2965, 2940, 2894, 2860, 1608 (**C=O**), 1589 (**C=O**), 1489, 1456, 1391, 1375, 1360, 1262, 1196, 1171, 1150, 1117, 1074, 1022, 952, 926, 869, 812, 771, 692, 644, 533, 512, 492.

Preparation of 2c

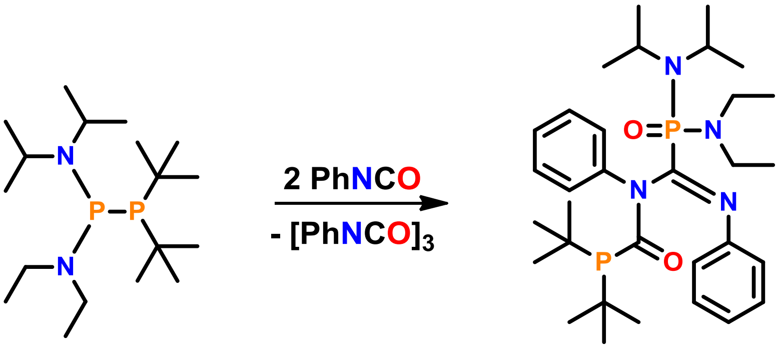

2 **2c/2c'**

To a solution of **2** (87 mg, 0.250 mmol) in THF (4 mL), an excess of PhNCO (0.5 mL, 548 mg, 4.600 mmol) was added at room temperature. The solution was stirred for 9 days yielding white precipitate. ^{31}P{^{1}H} NMR of the yellowish reaction mixture (suspension) revealed the complete conversion of **2** into **2c** and **2c'** with simultaneous formation of **[PhNCO]$_3$**. The solvent was evaporated, and the residue was dried under a vacuum (0.01 Torr) at 50°C to remove all volatiles. The mixture of **2c** and **[PhNCO]$_3$** was dissolved in 10 cm^3 of pentane and filtered to separate products: **[PhNCO]$_3$** as white solid, which was dried in vacuum (470 mg, 1.315 mmol) and solution of **2c**. The solvent was evaporated, giving a mixture of isomers **2c** and **2c'** as colourless oil. Yield 95% (140 mg, 0.239 mmol). The isolated product contains conformational isomers **2c** and **2c'** in a molar ratio of 1:0.75

NMR:

^{31}P{^{1}H} NMR (C_6D_6): δ 30.6 (s, P*t*Bu$_2$, **2c'**), 29.8 (s, P*t*Bu$_2$, **2c**), 14.7 (s, P(Et$_2$N)(*i*Pr$_2$N), **2c'**), 14.5 (s, P(Et$_2$N)(*i*Pr$_2$N), **2c**).

^{1}H NMR (C_6D_6): 7.24-6.87 (overlapped m, 10H, *o,m,p*-CH of **2c** and **2c'**), 3.91 (m, 2H, NC*H* of **2c**), 3.61 (m, 4H, NC*H$_2$* of **2c'**), 3.40 (m, 2H, NC*H* of **2c'**), 2.97 (m, 4H, NC*H$_2$* of **2c**), 1.60 (d, $^{3}J_{HH}$ = 6.6 Hz, 6H, CHC*H$_3$* of **2c**), 1.51 (d, $^{3}J_{PH}$ = 12.1 Hz, 18H, C(C*H$_3$*)$_3$ of **2c**), 1.31 (d, $^{3}J_{HH}$ = 6.8 Hz, 6H, CHC*H$_3$* of **2c**), 1.28 (d, $^{3}J_{PH}$ = 11.5 Hz, 9H, C(C*H$_3$*)$_3$ of **2c'**), 1.27 (d, $^{3}J_{HH}$ = 6.7 Hz, 6H, CHC*H$_3$* of **2c'**), 1.23 (t, $^{3}J_{HH}$ = 7.1 Hz, 6H, CH$_2$C*H$_3$* of **2c'**), 1.21 (d, $^{3}J_{HH}$ = 6.7 Hz, 6H, CHC*H$_3$* of **2c'**), 1.16 (d, $^{3}J_{PH}$ = 11.5 Hz, 9H, C(C*H$_3$*)$_3$ of **2c'**), 0.91 (t, $^{3}J_{HH}$ = 7.0 Hz, 6H, CH$_2$C*H$_3$* of **2c**).

^{13}C{^{1}H} NMR (C_6D_6): δ 181.6 (d, $^{1}J_{PC}$ = 41.8 Hz, C=O of **2c'**), 180.1 (d, $^{1}J_{PC}$ = 41.8 Hz, C=O of **2c**), 159.9 (d, $^{1}J_{PC}$ = 199.8 Hz, C=N of **2c'**), 158.6 (d, $^{1}J_{PC}$ = 200.7 Hz, C=N of **2c**), 148.5 (d, $^{3}J_{PC}$ = 18.2 Hz, *ipso*-CH of **2c**), 148.3 (d, $^{3}J_{PC}$ = 18.2 Hz, *ipso*-CH of **2c'**), 139.2 (d, $^{3}J_{PC}$ = 4.5 Hz, *ipso*-CH of **2c'**), 138.9 (d, $^{3}J_{PC}$ = 4.5 Hz, *ipso*-CH of **2c'**), 131.9 (d, *ortho*-CH of **2c**), 131.8 (d, *ortho*-CH of **2c'**), 128.9 (s, *ortho*-CH of **2c'**), 128.8 (s, *ortho*-CH of **2c**), 128.3 (s, *meta*-CH of **2c'**), 128.2 (s, *meta*-CH of **2c**), 128.0 (s, *para*-CH of **2c'**), 127.9 (s, *para*-CH of **2c**), 124.9 (s, *para*-CH of **2c'**), 124.7 (s, *para*-CH of **2c**), 118.8 (s, *meta*-CH of **2c'**), 118.6 (s, *meta*-CH of **2c**), 46.9 (d, $^{2}J_{PC}$ = 5.4 Hz, N*C*H of **2c**), 46.4 (d, $^{2}J_{PC}$ = 5.4 Hz, N*C*H of **2c'**), 38.2 (d, $^{2}J_{PC}$ = 4.5 Hz, N*C*H$_2$ of **2c'**), 37.1 (d, $^{2}J_{PC}$ = 4.5 Hz, N*C*H$_2$ of **2c**), 34.5 (d, $^{1}J_{PC}$ = 24.5 Hz, *C*(CH$_3$)$_3$ of **2c'**), 34.3 (d, $^{1}J_{PC}$ = 24.5 Hz, *C*(CH$_3$)$_3$ of **2c**), 33.0 (d, $^{1}J_{PC}$ = 24.5 Hz, *C*(CH$_3$)$_3$ of **2c**), 32.8 (d, $^{1}J_{PC}$ = 24.5 Hz, *C*(CH$_3$)$_3$ of **2c'**), 30.3 (d, $^{2}J_{PC}$ = 14.5 Hz, C(*CH$_3$*)$_3$ of **2c'**), 30.2 (d, $^{2}J_{PC}$ = 14.5 Hz, C(*CH$_3$*)$_3$ of **2c'**), 30.1 (d, $^{2}J_{PC}$ = 14.5 Hz, C(*CH$_3$*)$_3$ of **2c**), 23.1 (s, CH*C*H$_3$ of **2c'**), 22.8 (s, CH*C*H$_3$ of **2c**), 13.1 (d, $^{3}J_{PC}$ = 2.7 Hz, CH$_2$*C*H$_3$ of **2c'**), 12.9 (d, $^{3}J_{PC}$ = 2.7 Hz, CH$_2$*C*H$_3$ of **2c**).

Preparation of 3c

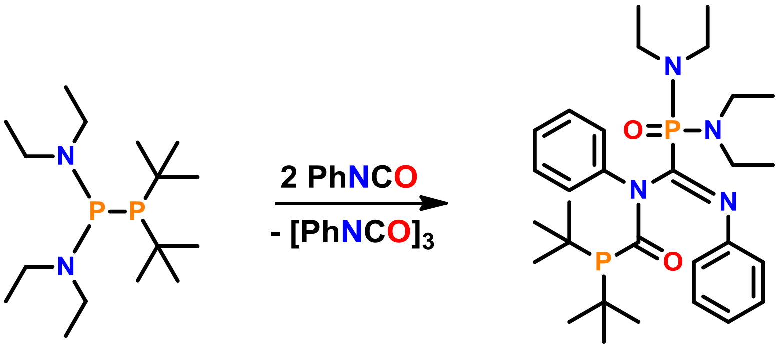

3 **3c**

To a solution of **3** (80 mg, 0.250 mmol) in THF (4 mL), an excess of PhNCO (0.5 mL, 548 mg, 4.600 mmol) was added at room temperature. The solution was stirred for 7 days yielding white precipitate. $^{31}P\{^{1}H\}$ NMR of the yellowish reaction mixture (suspension) revealed the complete conversion of **3** into **3c** and simultaneous formation of **[PhNCO]$_3$**. The solvent was evaporated, and the residue was dried under a vacuum (0.01 Torr) at 50°C to remove all volatiles. The mixture of **3c** and **[PhNCO]$_3$** was dissolved in 10 cm^3 of pentane and filtered to separate products: **[PhNCO]$_3$** as white solid, which was dried in vacuum (481 mg, 1.346 mmol) and solution of **3c**. The solution was concentrated and left at -20°C to afford colourless X-ray quality crystals of **3c**. Yield 82% (115 mg, 0.206 mmol). **3c** is air- and moisture-stable, both in the solid-state and solution.

NMR:

$^{31}P\{^{1}H\}$ NMR (C_6D_6): δ 31.4 (s, P(Et_2N)$_2$), 16.8 (s, P*t*Bu_2).

^{1}H NMR (C_6D_6): 7.26 (m, 2H, o-C*H*), 7.12 (m, 2H, o-C*H*), 6.98 (m, 2H, m-C*H*), 6.94 (m, 1H, p-C*H*), 6.91 (m, 2H, m-C*H*), 6.86 (m, 1H, p-C*H*), 3.43 (broad m, 2H, CH_2), 3.32 (broad m, 2H, CH_2), 3.23 (broad m, 2H, CH_2), 2.89 (broad m, 2H, CH_2), 1.50 (d, $^{3}J_{PH}$ = 11.7, 9H, C(CH_3)$_3$), 1.27 (d, $^{3}J_{PH}$ = 11.5, 9H, C(CH_3)$_3$), 1.14 (t, $^{3}J_{HH}$ = 6.6 Hz, 6H, CH_3), 0.91 (t, $^{3}J_{HH}$ = 6.5 Hz, 6H, CH_3).

$^{13}C\{^{1}H\}$ NMR (C_6D_6): δ 181.6 (d, $^{1}J_{PC}$ = 42.7 Hz, C=O), 159.7 (d, $^{1}J_{PC}$ = 204.4 Hz, C=N), 148.3 (d, $^{3}J_{PC}$ = 18.2 Hz, *ipso*-CH), 139.4 (d, $^{3}J_{PC}$ = 4.5 Hz, *ipso*-CH), 131.5 (d, $^{4}J_{PC}$ = 3.6 Hz, *ortho*-CH), 128.8 (s, *ortho*-CH), 128.0 (s, *meta*-CH), 127.9 (s, *para*-CH), 124.9 (s, *meta*-CH), 118.8 (s, *para*-CH), 37.9 (s, CH_2), 37.4 (s, CH_2), 34.6 (d, $^{1}J_{PC}$ = 23.6 Hz, *C*(CH_3)$_3$), 32.8 (d, $^{1}J_{PC}$ = 24.5 Hz, *C*(CH_3)$_3$), 30.3 (d, $^{2}J_{PC}$ = 14.5 Hz, C(*CH_3*)$_3$), 30.2 (d, $^{2}J_{PC}$ = 14.5 Hz, C(*CH_3*)$_3$), 13.4 (s, CH_3), 13.0 (s, CH_3).

Elemental analysis: calcd. for $C_{30}H_{48}N_4O_2P_2$: C, 64.50; H, 8.66; N, 10.03. Found: C, 64.45; H, 8.790; N, 10.04.

IR (solid): $\tilde{\upsilon}$ = 2969, 2937, 2892, 2866, 1631 (**C=O**), 1610 (**C=N**), 1590 (**C=N**), 1489, 1477, 1458, 1383, 1369, 1363, 1354, 1278, 1228, 1206, 1191, 1169, 1109, 1073, 1059, 1018, 944, 930, 903, 813, 799, 789, 764, 738, 713, 696, 672, 648, 585, 536, 519, 506.

Preparation of 6c

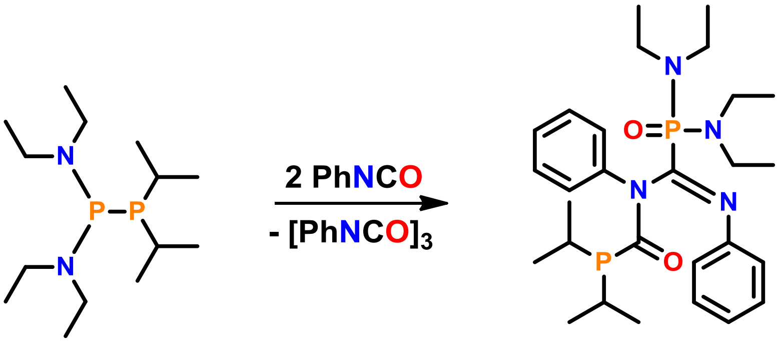

6 **6c**

To a solution of **6** (73 mg, 0.250 mmol) in THF (4 mL), an excess of PhNCO (0.5 mL, 548 mg, 4.600 mmol) was added at room temperature. The solution was stirred for 20 days yielding white precipitate. $^{31}P\{^1H\}$ NMR of the yellowish reaction mixture (suspension) revealed the complete conversion of **6** into **6c** and simultaneous formation of **[PhNCO]$_3$**. The solvent was evaporated, and the residue was dried under a vacuum (0.01 Torr) at 50°C to remove all volatiles. The mixture of **6c** and **[PhNCO]$_3$** was dissolved in 10 cm^3 of pentane and filtered to separate products: **[PhNCO]$_3$** as white solid, which was dried in vacuum (450 mg, 1.259 mmol) and solution of **6c**. The solution was concentrated and left at -30°C to afford colourless X-ray quality crystals of **6c**. Yield 73% (97 mg, 0.183 mmol). **6c** is air- and moisture-stable, both in the solid-state and solution.

NMR:

$^{31}P\{^1H\}$ NMR (C_6D_6): δ 16.6 (s, P(Et$_2$N)$_2$), 12.0 (s, P*i*Pr$_2$).

^1H NMR (C_6D_6): 7.28 (m, 2H, o-C*H*), 7.12 (m, 2H, o-C*H*), 7.02 (m, 2H, m-C*H*), 6.96 (m, 1H, p-C*H*), 6.92 (m, 2H, m-C*H*), 6.86 (m, 1H, p-C*H*), 3.41 (broad m, 2H, C*H*$_2$), 3.31 (broad m, 2H, C*H*$_2$), 3.22 (broad m, 2H, C*H*$_2$), 2.90 (broad m, 2H, C*H*$_2$), 2.29 (broad s, 2H, CH), 1.44 (broad m, 6H, CHC*H*$_3$), 1.24 (broad overlapped m, 6H, CHC*H*$_3$), 1.14 (broad m, 6H, CH$_2$C*H*$_3$), 0.92 (broad m, 6H, CH$_2$C*H*$_3$).

$^{13}C\{^1H\}$ NMR (C_6D_6): δ 181.7 (d, $^1J_{PC}$ = 35.4 Hz, C=O), 159.4 (d, $^1J_{PC}$ = 204.4 Hz, C=N), 148.3 (d, $^3J_{PC}$ = 18.2 Hz, *ipso*-CH), 138.7 (d, $^3J_{PC}$ = 3.6 Hz, *ipso*-CH), 131.4 (d, $^4J_{PC}$ = 3.6 Hz, *ortho*-CH), 128.8 (s, *ortho*-CH), 128.2 (s, *meta*-CH), 128.1 (s, *para*-CH), 125.1 (s, *para*-CH), 119.0 (s, *meta*-CH), 38.0 (s, CH$_2$), 37.4 (s, CH$_2$), 24.4 (d, $^1J_{PC}$ = 13.6 Hz, CH), 22.6 (d, $^1J_{PC}$ = 14.5 Hz, CH), 20.4 (s, CH*CH*$_3$), 20.2 (s, CH*CH*$_3$), 19.0 (s, CH*CH*$_3$), 18.9 (s, CH*CH*$_3$), 13.4 (s, CH$_2$*CH*$_3$), 13.1 (s, CH$_2$*CH*$_3$).

Elemental analysis: calcd. for $C_{28}H_{44}N_4O_2P_2$: C, 63.38; H, 8.36; N, 10.56. Found: C, 63.39; H, 8.290; N, 10.59.

IR (solid): $\tilde{\upsilon}$ = 2973, 2952, 2933, 2866, 1633 (**C=O**), 1609 (**C=N**), 1589 (**C=N**), 1485, 1462, 1379, 1289, 1228, 1206, 1190, 1162, 1101, 1064, 1017, 949, 931, 918, 788, 766, 744, 714, 694, 672, 638, 586, 540, 513.

Preparation of 9c

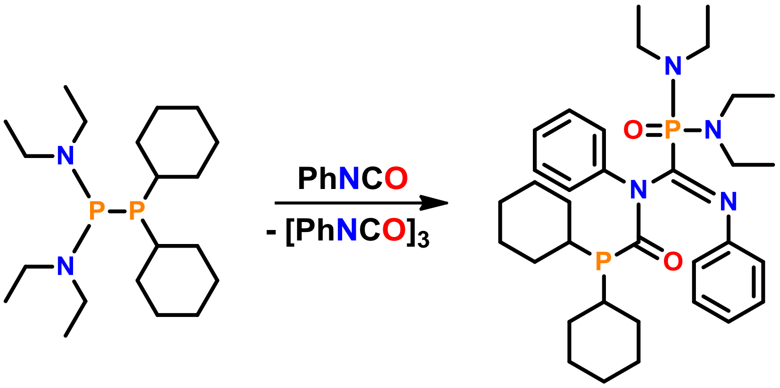

9 9c

To a solution of **9** (93 mg, 0.250 mmol) in THF (4 mL), an excess of PhNCO (0.5 mL, 548 mg, 4.600 mmol) was added at room temperature. The solution was stirred for 27 days yielding white precipitate. $^{31}P\{^1H\}$ NMR of the yellowish reaction mixture (suspension) revealed the complete conversion of **9** into **9c** and simultaneous formation of **[PhNCO]$_3$**. The solvent was evaporated, and the residue was dried under a vacuum (0.01 Torr) at 50°C to remove all volatiles. The mixture of **9c** and **[PhNCO]$_3$** was dissolved in 10 cm^3 of pentane and filtered to separate products: **[PhNCO]$_3$** as white solid, which was dried in vacuum (469 mg, 1.312 mmol) and solution of **9c**. The solution was concentrated and left at -20°C to afford colourless X-ray quality crystals of **9c**. Yield 85% (130 mg, 0.213 mmol). **9c** is air- and moisture-stable, both in the solid-state and solution.

NMR:

$^{31}P\{^1H\}$ NMR (C_6D_6): δ 16.7 (s, P($Et_2N)_2$), 4.0 (s, PCy_2).

^1H NMR (C_6D_6): 7.32 (m, 2H, o-C*H*), 7.18 (m, 2H, o-C*H*), 7.04 (m, 2H, m-C*H*), 6.99 (m, 1H, p-C*H*), 6.95 (m, 2H, m-C*H*), 6.88 (m, 1H, p-C*H*), 3.39 (broad m, 2H, CH_2), 3.30 (broad m, 2H, CH_2), 3.21 (broad m, 2H, CH_2), 2.88 (broad m, 2H, CH_2), 2.32-1.32 (broad overlapped m, 22H, CH and CH_2 of Cy groups) 1.15 (broad m, 6H, CH_3), 0.91 (broad m, 6H, CH_3).

$^{13}C\{^1H\}$ NMR (C_6D_6): δ 181.7 (d, $^1J_{PC}$ = 35.4 Hz, C=O), 159.3 (d, $^1J_{PC}$ = 203.5 Hz, C=N), 148.4 (d, $^3J_{PC}$ = 18.2 Hz, *ipso*-CH), 138.9 (d, $^3J_{PC}$ = 3.6 Hz, *ipso*-CH), 131.4 (d, $^4J_{PC}$ = 4.5 Hz, *ortho*-CH), 128.8 (s, *ortho*-CH), 128.2 (s, *meta*-CH), 128.1 (s, *para*-CH), 125.0 (s, *para*-CH), 119.0 (s, *meta*-CH), 38.1 (s, N*C*H_2), 37.4 (s, N*C*H_2), 34.0 (d, $^1J_{PC}$ = 16.3 Hz, CH), 32.6 (d, $^1J_{PC}$ = 14.5 Hz, CH), 30.4 (d, $^2J_{PC}$ = 11.8 Hz, CH_2), 29.4 (s, CH_2), 29.3 (s, CH_2), 27.6 (broad m, CH_2), 27.1 (broad m, CH_2), 26.5 (s, CH_2), 13.5 (s, CH_3), 13.1 (s, CH_3).

Elemental analysis: calcd. for $C_{34}H_{54}N_4O_2P_2$: C, 66.86; H, 8.58; N, 9.17. Found: C, 66.92; H, 8.566; N, 9.22.

IR (solid): $\tilde{\upsilon}$ = 2968, 2922, 2849, 1636 (**C=O**), 1608 (**C=N**), 1589 (**C=N**), 1493, 1445, 1388, 1376, 1355, 1285, 1229, 1207, 1196, 1162, 1096, 1070, 1030, 1015, 954, 933, 798, 784, 769, 742, 712, 698, 665, 589, 539, 511, 478.

Preparation of 1d

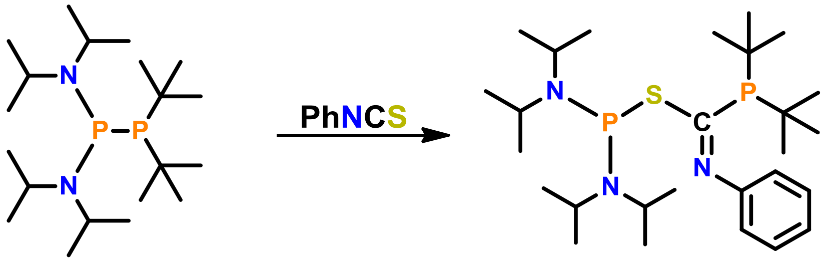

1 **1d/1d'**

To a solution of **1** (188 mg, 0.500 mmol) in toluene (5 mL) an excess of PhNCS (0.07 mL, 81 mg, 0.600 mmol) was added dropwise at room temperature. The solution was stirred for 13 days. $^{31}P\{^1H\}$ NMR of the orange reaction mixture revealed the complete conversion of **1** into **1d** and **1d'**. The solvent was evaporated, and the residue was dried under a vacuum (0.01 Torr) at 50°C to remove all volatiles. The oily product slowly solidifies at -20°C to afford yellow X-ray quality crystals of **1d/1d'**. Yield 98% (250 mg, 0.489 mmol). The isolated product contains conformational isomers **1d** and **1d'** in a molar ratio of 1.35:1.

NMR:

$^{31}P\{^1H\}$ NMR (C_6D_6): **1d**: δ 101.2 (d, $^3J_{PP}$ = 109.0 Hz, P(*i*Pr$_2$N)$_2$), 45.1 (d, $^3J_{PP}$ = 109.0 Hz, P*t*Bu$_2$). **1d'**: δ 97.7 (d, $^3J_{PP}$ = 8.0 Hz, P(*i*Pr$_2$N)$_2$), 39.0 (d, $^3J_{PP}$ = 8.0 Hz, P*t*Bu$_2$).

^1H NMR (C_6D_6): 1d: δ 7.20 (m, 2H, *o*-CH), 6.99-6.83 (m, 3H, p,*m*-CH, overlapped with **1d'**), 3.34 (sept, $^3J_{HH}$ = 6.6 Hz, 4H, C*H*), 1.51 (d, $^3J_{PH}$ = 11.1, 18H, C(C*H$_3$*)$_3$), 1.20 (d, $^3J_{HH}$ = 6.6 Hz, 12H, CHC*H$_3$*), 1.06 (d, $^3J_{HH}$ = 6.6 Hz, 12H, CHC*H$_3$*). **1d'**: δ 7.16 (m, 2H, *o*-CH), 6.99-6.83 (m, 3H, p,*m*-CH, overlapped with **1d**), 3.57 (sept, $^3J_{HH}$ = 6.1 Hz, 4H, C*H*), 1.38 (d, $^3J_{PH}$ = 11.9, 18H, C(C*H$_3$*)$_3$), 1.33 (d, $^3J_{HH}$ = 6.1 Hz, 12H, CHC*H$_3$*), 1.15 (d, $^3J_{HH}$ = 6.1 Hz, 12H, CHC*H$_3$*).

$^{13}C\{^1H\}$ NMR (C_6D_6): 1d: δ 170.8 (dd, $^1J_{PC}$ = 56.3 Hz, $^2J_{PC}$ = 10.9 Hz, *C*=N), 151.5 (s, *ipso*-C), 128.4 (s, *ortho*-C), 123.6 (s, *para*-CH), 120.1 (s, *meta*-CH), 48.0 (d, $^2J_{PC}$ = 12.7 Hz, N*C*H), 34.3 (dd, $^1J_{PC}$ = 28.2 Hz, $^4J_{PC}$ = 3.6 Hz *C*(CH$_3$)$_3$), 30.6 (s, C(*CH$_3$*)$_3$), 23.5 (d, $^3J_{PC}$ = 9.1 Hz, CH*CH$_3$*), 23.2 (d, $^3J_{PC}$ = 9.1 Hz, CH*CH$_3$*). **1d':** δ 172.7 (dd, $^1J_{PC}$ = 75.4 Hz, $^2J_{PC}$ = 5.4 Hz, *C*=N), 152.7 (d, $^3J_{PC}$ = 15.4 Hz, *ipso*-C), 128.2 (s, *ortho*-C), 128.0 (s, *para*-CH), 122.2 (s, *meta*-CH), 48.3 (d, $^2J_{PC}$ = 12.7 Hz, N*C*H), 32.9 (d, $^1J_{PC}$ = 29.1 Hz, *C*(CH$_3$)$_3$), 30.8 (s, C(*CH$_3$*)$_3$), 23.9 (d, $^3J_{PC}$ = 5.4 Hz, CH*CH$_3$*), 23.8 (d, $^3J_{PC}$ = 5.4 Hz, CH*CH$_3$*).

Elemental analysis: calcd. for $C_{27}H_{51}N_3P_2S$: C, 63.37; H, 10.05; N, 8.21; S, 6.27. Found: C, 63.26; H, 10.071; N, 8.29; S, 6.313.

IR (solid): $\tilde{\nu}$ = 2962, 2931, 2861, 1557 (**C=N**), 1483, 1453, 1384, 1362, 1195, 1175, 1157, 1114, 1071, 1019, 948, 906, 863, 810, 799, 756, 691, 621, 592, 578, 515, 448, 432.

Preparation of 2d

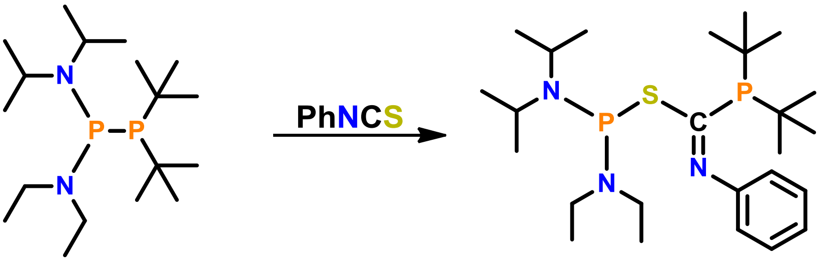

2 **2d**

To a solution of **2** (174 mg, 0.500 mmol) in THF (5 mL) an excess of PhNCS (0.07 mL, 81 mg, 0.600 mmol) was added dropwise at room temperature. The solution was stirred for 24 hours. ^{31}P{^{1}H} NMR of the orange reaction mixture revealed the complete conversion of **2** into **2d**. The solvent was evaporated, and the residue was dried under a vacuum (0.01 Torr) at 50°C to remove all volatiles. The oily product slowly solidifies at -20°C to afford yellow X-ray quality crystals of **2d**. Yield 97% (234 mg, 0.484 mmol).

NMR:

^{31}P{^{1}H} NMR (C_6D_6): δ 106.6 (d, $^{3}J_{PP}$ = 130.8 Hz, P(Et_2N)($i$$Pr_2$N)), 43.0 (d, $^{3}J_{PP}$ = 130.8 Hz, P$t$$Bu_2$).

^{1}H NMR (C_6D_6): δ 7.21 (m, 2H, *o*-CH), 6.98 (m, 2H, *m*-CH), 6.93 (m, 1H, *p*-CH), 3.28 (m, 2H, CH_2N), 3.19 (m, 2H, C*H*N), 3.01 (m, 2H, CH_2N), 1.53 (d, $^{3}J_{PH}$ = 10.9 Hz, 9H, C(CH_3)$_3$), 1.48 (d, $^{3}J_{PH}$ = 11.4 Hz, 9H, C(CH_3)$_3$), 1.06 (d, $^{3}J_{HH}$ = 6.7 Hz, 6H, CHCH_3), 1.02 (t, $^{3}J_{HH}$ = 7.3 Hz, 6H, CH_2CH_3), 1.01 (d, $^{3}J_{HH}$ = 6.7 Hz, 6H, CHCH_3).

^{13}C{^{1}H} NMR (C_6D_6): δ 171.4 (dd, $^{1}J_{PC}$ = 54.5 Hz, $^{2}J_{PC}$ = 7.3 Hz, *C*=N), 152.0 (s, *ipso*-C), 128.7 (s, *ortho*-C), 123.5 (s, *para*-CH), 119.4 (s, *meta*-CH), 47.5 (d, $^{2}J_{PC}$ = 14.5 Hz, N*C*H), 43.1 (d, $^{2}J_{PC}$ = 19.1 Hz, NCH_2) 43.0 (d, $^{2}J_{PC}$ = 19.1 Hz, NCH_2), 34.3 (dd, $^{1}J_{PC}$ = 27.2 Hz, $^{4}J_{PC}$ = 2.7 Hz, *C*(CH_3)$_3$), 34.0 (dd, $^{1}J_{PC}$ = 28.2 Hz, $^{4}J_{PC}$ = 6.4 Hz, *C*(CH_3)$_3$), 30.6 (d, $^{2}J_{PC}$ = 13.6 Hz, C(CH_3)$_3$), 30.2 (d, $^{2}J_{PC}$ = 14.5 Hz, C(CH_3)$_3$), 23.5 (d, $^{3}J_{PC}$ = 7.3 Hz, CHCH_3), 23.3 (d, $^{3}J_{PC}$ = 9.1 Hz, CHCH_3), 14.7 (d, $^{3}J_{PC}$ = 3.6 Hz, $CH_2$$CH_3$).

Elemental analysis: calcd. for $C_{25}H_{47}N_3P_2S$: C, 62.08; H, 9.79; N, 8.69; S, 6.63. Found: C, 61.89; H, 9.724; N, 8.69; S, 6.471.

IR (solid): $\tilde{\upsilon}$ = 2964, 2930, 2891, 2859, 1592 **(C=N)**, 1566 **(C=N)**, 1483, 1474, 1450, 1385, 1375, 1362, 1197, 1178, 1121, 1071, 1017, 955, 926, 911, 869, 807, 799, 787, 755, 693, 659, 624, 595, 577, 508, 464.

Preparation of 3d

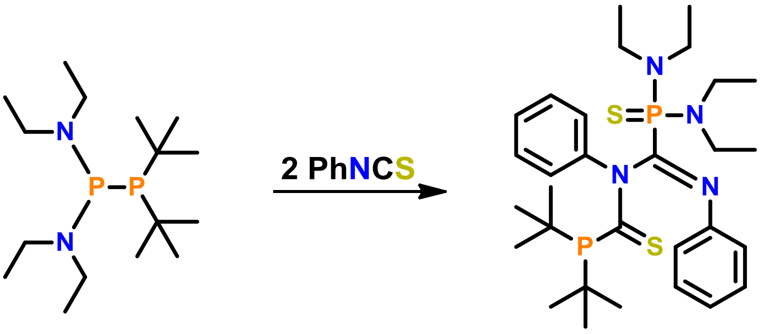

3 3d/3d'

To a solution of **3** (160 mg, 0.500 mmol) in toluene (5 mL) an excess of PhNCS (0.13 mL, 149 mg, 1.100 mmol) was added dropwise at room temperature. The solution was stirred for 3 days. $^{31}P\{^{1}H\}$ NMR of the orange reaction mixture revealed the complete conversion of **3** into **3d** and **3d'**. The solvent was evaporated, and the residue was dried under a vacuum (0.01 Torr) at 50°C to remove all volatiles. The red oily residue was dissolved in 5 mL of pentane and left at -20ºC to afford red X-ray quality crystals of **3d/3d'**. Yield 67% (197 mg, 0.333 mmol). The isolated product contains conformational isomers **3d** and **3d'** in a molar ratio of 2:1.

NMR:

$^{31}P\{^{1}H\}$ NMR (C_6D_6): **3d**: δ 71.5 (s, $P(Et_2N)_2$), 39.8 (s, P*t*Bu_2). **3d'**: δ 68.2 (s, $P(Et_2N)_2$), 43.6 (s, P*t*Bu_2).

^{1}H NMR (C_6D_6): δ 7.19-6.74 (overlapped m, 20H, *o,m,p*-CH of **3d** and **3d'**), 3.75 (m, 2H, CH_2 of **3d'**), 3.25 (m, 8H, CH_2 of **3d**), 3.12 (m, 4H, CH_2 of **3d'**), 2.89 (m, 2H, CH_2 of **3d'**), 1.47 (d, $^{3}J_{PH}$ = 12.4 Hz, 9H, C(CH_3)$_3$ of **3d'**), 1.32 (d, $^{3}J_{PH}$ = 12.1 Hz, 9H, C(CH_3)$_3$ of **3d'**), 1.17 (d, $^{3}J_{PH}$ = 12.0 Hz, 18H, C(CH_3)$_3$ of **3d**), 1.12 (t, $^{3}J_{HH}$ = 7.1 Hz, 6H, CH_2CH_3 of **3d'**), 1.04 (t, $^{3}J_{HH}$ = 7.0 Hz, 12H, CH_2CH_3 of **3d**), 0.97 (t, $^{3}J_{HH}$ = 7.0 Hz, 6H, CH_2CH_3 of **3d'**).

$^{13}C\{^{1}H\}$ NMR (C_6D_6): δ 218.9 (d, $^{1}J_{PC}$ = 59.9 Hz, *C*=S of **3d**), 159.4 (d, $^{1}J_{PC}$ = 158.9 Hz, *C*=N of **3d**), 159.3 (d, $^{1}J_{PC}$ = 157.1 Hz, $^{3}J_{PC}$ = 5.4 Hz, *C*=N of **3d'**), 151.9 (d, $^{3}J_{PC}$ = 15.4 Hz, *ipso*-C of **3d'**), 150.6 (d, $^{3}J_{PC}$ = 23.6 Hz, *ipso*-C of **3d'**), 147.8 (d, $^{3}J_{PC}$ = 17.2 Hz, *ipso*-C of **3d**), 142.1 (d, $^{3}J_{PC}$ = 5.4 Hz, *ipso*-C of **3d**), 128.9-118.4 (s, Ar-C of **3d** and **3d'**), 39.4 (d, $^{2}J_{PC}$ = 4.5 Hz, N*C*H_2 of **3d**), 37.9 (s, N*C*H_2 of **3d'**), 37.8 (s, N*C*H_2 of **3d'**), 35.9 (d, $^{1}J_{PC}$ = 26.3 Hz, *C*$(CH_3)_3$ of **3d'**), 33.9 (d, $^{1}J_{PC}$ = 27.2 Hz, *C*$(CH_3)_3$ of **3d'**), 33.0 (d, $^{1}J_{PC}$ = 30.0 Hz, *C*$(CH_3)_3$ of **3d**), 30.6 (d, $^{2}J_{PC}$ = 16.4 Hz, C(*C*H_3)$_3$ of **3d**), 30.5 (d, $^{2}J_{PC}$ = 15.4 Hz, C(*C*H_3)$_3$ of **3d'**), 30.0 (d, $^{2}J_{PC}$ = 16.4 Hz, C(*C*H_3)$_3$ of **3d'**), 13.6 (broad s, CH_2*C*H_3 of **3d**), 12.6 (s, CH_2*C*H_3 of **3d'**), 12.5 (s, of **3d'**).

Elemental analysis: calcd. for $C_{30}H_{48}N_4P_2S_2$: C, 60.99; H, 8.19; N, 9.48; S, 10.85. Found: C, 60.94; H, 8.298; N, 9.60; S, 10.644.

IR (solid): $\tilde{\upsilon}$ = 2971, 2930, 2892, 2861, 1614 (**C=N**), 1591 (**C=N**), 1484, 1472, 1455, 1377, 1354, 1304, 1197, 1172, 1103, 1072, 1058, 1014, 945, 934, 926, 912, 872, 789, 759, 720, 692, 626, 618, 604, 535, 510, 493.

Preparation of 3d*

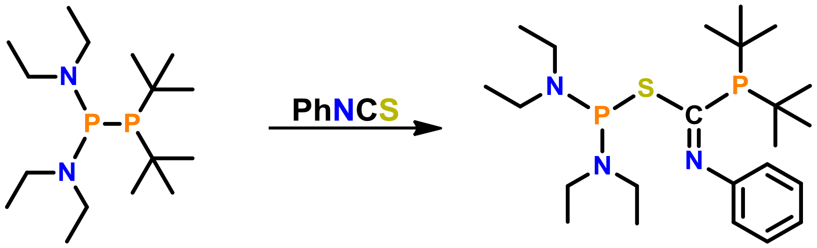

3 **3d***

To a solution of **3** (160 mg, 0.500 mmol) in toluene (5 mL) an equimolar amount of PhNCS (0.06 mL, 68 mg, 0.500 mmol) was added dropwise at room temperature. The solution was stirred for 12 hours. $^{31}P\{^1H\}$ NMR of the yellow reaction mixture revealed the complete conversion of **3** into **3d***. The solvent was evaporated, and the residue was dried under vacuum (0.01 Torr) at 50°C to remove all volatiles giving **3d*** as an orange oil (225 mg, 0.494 mmol).

NMR:

^{31}P{^{1}H} NMR (C_6D_6): δ 126.4 (d, $^3J_{PP}$ = 138.1 Hz, P(Et_2N)$_2$), 44.2 (d, $^3J_{PP}$ = 138.1 Hz, P*t*Bu_2).

^{1}H NMR (C_6D_6): δ 7.21 (m, 2H, *o*-CH), 6.99 (m, 2H, *m*-CH), 6.94 (m, 1H, *p*-CH), 3.05 (m, 4H, C*H_2*), 2.90 (m, 4H, C*H_2*), 1.50 (d, $^3J_{PH}$ = 11.1 Hz, 18H, C(C*H_3*)$_3$), 0.93 (t, $^3J_{HH}$ = 7.1 Hz, 12H, C*H_3*).

^{13}C{^{1}H} NMR (C_6D_6): δ 171.4 (dd, $^1J_{PC}$ = 52.7 Hz, $^2J_{PC}$ = 7.3 Hz, *C*=N), 152.1 (d, $^3J_{PC}$ = 1.8 Hz, *ipso*-C), 128.7 (s, *ortho*-C), 123.5 (s, *para*-CH), 119.4 (s, *meta*-CH), 43.0 (d, $^2J_{PC}$ = 18.2 Hz, *C*H_2), 42.9 (d, $^2J_{PC}$ = 18.2 Hz, *C*H_2), 34.3 (dd, $^1J_{PC}$ = 27.2 Hz, $^4J_{PC}$ = 4.5 Hz, *C*(CH_3)$_3$), 30.3 (d, $^2J_{PC}$ = 12.7 Hz, C(*CH_3*)$_3$), 14.6 (d, $^3J_{PC}$ = 3.6 Hz, *C*H_3).

Preparation of 4d

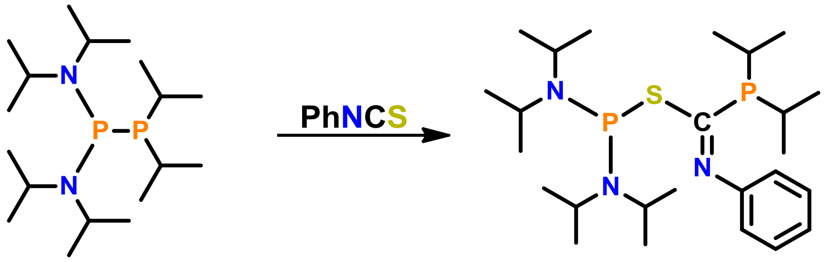

4 **4d/4d'**

To a solution of **4** (174 mg, 0.500 mmol) in THF (5 mL) an excess of PhNCS (0.07 mL, 81 mg, 0.600 mmol) was added dropwise at room temperature. The solution was stirred for 24 h. $^{31}P\{^{1}H\}$ NMR of the orange reaction mixture revealed the complete conversion of **4** into **4d** and **4d'**. The solvent was evaporated, and the residue was dried under a vacuum (0.01 Torr) at 50°C to remove all volatiles. The oily product slowly solidifies at -20°C to afford yellow X-ray quality crystals of **4d/4d'**. Yield 98% (238 mg, 0.492 mmol). The isolated product contains conformational isomers **4d** and **4d'** in a molar ratio of 2:1.

NMR:

$^{31}P\{^{1}H\}$ NMR (toluene-d_8, 273K): **4d**: δ 97.9 (d, $^3J_{PP}$ = 130.8 Hz, P(*i*Pr$_2$N)$_2$), 24.6 (d, $^3J_{PP}$ = 130.8 Hz, P*i*Pr$_2$). **4d'**: δ 98.7 (d, $^3J_{PP}$ = 14.5 Hz, P(*i*Pr$_2$N)$_2$), 13.9 (d, $^3J_{PP}$ = 14.5 Hz, P*i*Pr$_2$).

^1H NMR (toluene-d_8, 273K): 4d: δ 7.23 (m, 2H, *o*-CH), 6.98 (m, 2H, *m*-CH), 6.88 (m, 1H, *p*-CH), 3.36 (m, 4H, NC*H*), 2.61 (m, 2H, C*H*), 1.44 (dd, $^3J_{PH}$ = 14.5 Hz, $^3J_{HH}$ = 7.1 Hz, 6H, CHC*H$_3$*), 1.30 (dd, $^3J_{PH}$ = 11.1 Hz, $^3J_{HH}$ = 7.3 Hz, 6H, CHC*H$_3$*), 1.24 (d, $^3J_{HH}$ = 6.6 Hz, 12H, NCHC*H$_3$*), 1.10 (d, $^3J_{HH}$ = 6.7 Hz, 12H, NCHC*H$_3$*). **4d'**: δ 7.18 (m, 2H, *o*-CH), 6.95 (m, 2H, *m*-CH, overlapped by **4d**), 6.89 (m, 1H, *p*-CH, overlapped by **4d**), 3.60 (m, 4H, NC*H*), 2.33 (m, 2H, C*H*), 1.37-1.27 (overlapped m, 6H, CHC*H$_3$*), 1.36 (d, $^3J_{HH}$ = 6.7 Hz, 12H, NCHC*H$_3$*), 1.21 (d, $^3J_{HH}$ = 6.6 Hz, 12H, NCHC*H$_3$*), 1.03 (d, $^3J_{PH}$ = 14.7 Hz, $^3J_{HH}$ = 7.1 Hz, 6H, CHC*H$_3$*).

$^{13}C\{^{1}H\}$ NMR (toluene-d_8, 273K): 4d: δ 171.4 (dd, $^1J_{PC}$ = 44.5 Hz, $^2J_{PC}$ = 10.0 Hz, *C*=N), 152.1 (d, $^3J_{PC}$ = 2.7 Hz, *ipso*-C), 128.5 (s, *ortho*-C), 123.5 (s, *para*-CH), 120.2 (s, *meta*-CH), 47.8 (d, $^2J_{PC}$ = 13.6 Hz, N*C*H), 24.2 (dd, $^1J_{PC}$ = 17.3 Hz, $^4J_{PC}$ = 5.4 Hz, *C*HCH$_3$), 23.7 (d, $^3J_{PC}$ = 5.4 Hz, NCH*CH$_3$*), 23.4 (d, $^3J_{PC}$ = 10.9 Hz, NCH*CH$_3$*), 21.1 (s, CH*CH$_3$*), 20.9 (s, CH*CH$_3$*). **4d':** δ 175.2 (dd, $^1J_{PC}$ = 59.9 Hz, $^2J_{PC}$ = 8.2 Hz, *C*=N), 152.6 (d, $^3J_{PC}$ = 14.5 Hz, *ipso*-C), 128.4 (s, *ortho*-C), 122.6 (s, *para*-CH), 120.4 (s, *meta*-CH), 48.1 (d, $^2J_{PC}$ = 12.7 Hz, N*C*H), 26.2 (d, $^1J_{PC}$ = 18.2 Hz, *C*HCH$_3$), 24.0 (d, $^3J_{PC}$ = 4.5 Hz, NCH*CH$_3$*), 23.2 (d, $^3J_{PC}$ = 9.1 Hz, NCH*CH$_3$*), 18.6 (s, CH*CH$_3$*), 18.5 (s, CH*CH$_3$*).

Elemental analysis: calcd. for $C_{25}H_{47}N_3P_2S$: C, 62.08; H, 9.79; N, 8.69; S, 6.63. Found: C, 61.98; H, 9.767; N, 8.77; S, 6.693.

IR (solid): $\tilde{\nu}$ = 2970, 2957, 2923, 2864, 1594 (**C=N** of **4d'**), 1560 (**C=N** of **4d**), 1481, 1463, 1452, 1388, 1379, 1363, 1194, 1176, 1156, 1115, 1021, 949, 912, 881, 864, 758, 692, 527, 513, 479, 451.

Formation of 5d

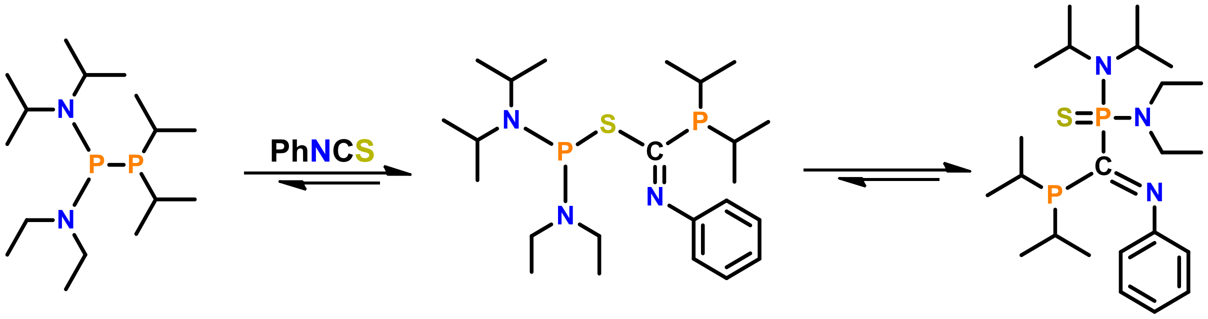

5 **5d*/5d**** **5d**

To a solution of **5** (160 mg, 0.500 mmol) in C_6D_6 (4 mL) an excess of PhNCS (0.12 mL, 135 mg, 1.000 mmol) was added at room temperature. The reaction progress was monitored by $^{31}P\{^1H\}$ and ^1H NMR, and after stirring the solution for 9 days it revealed the complete conversion of **5** into the mixture of **5d*, 5d**** and **5d** (see Figure S118). Complete rearrangement of **5*** and **5d**** into the final product **5d** was observed after 53 days. Product was not isolated as the formation of **5d** is reversible in the absence of PhNCS – after evaporation of PhNCS, there is established an equilibrium between **5**, **5d***, **5d**** and **5d**.

NMR:

$^{31}P\{^1H\}$ NMR (C_6D_6): δ 65.7 (d, $^2J_{PP}$ = 43.6 Hz, S=P(Et$_2$N)(*i*Pr$_2$N)), 26.5 (d, $^2J_{PP}$ = 43.6 Hz, P*i*Pr$_2$).

^1H NMR (C_6D_6): δ 7.15 (m, 2H, *o*-CH, overlapped with C_6D_6 residual peak), 6.90 (m, 1H, *p*-CH), 6.82 (m, 2H, *m*-CH), 3.83 (sept, $^3J_{HH}$ = 7.2 Hz, 2H, NC*H*), 3.50 (m, 2H, NC*H$_2$*), 3.07 (m, 2H, NC*H$_2$*), 3.01 (overlapped m, 2H, C*H*CH$_3$), 1.38 (d, $^3J_{HH}$ = 7.2 Hz, 6H, NCHC*H$_3$*), 1.36 (d, $^3J_{HH}$ = 7.2 Hz, 6H, NCHC*H$_3$*), 1.17 (dd, $^3J_{PH}$ = 15.7 Hz, $^3J_{HH}$ = 7.1 Hz, 6H, CHC*H$_3$*), 1.04 (dd, $^3J_{PH}$ = 14.2 Hz, $^3J_{HH}$ = 7.1 Hz, 6H, CHC*H$_3$*), 1.03 (t, $^3J_{HH}$ = 7.0 Hz, 12H, CH$_2$C*H$_3$*).

$^{13}C\{^1H\}$ NMR (C_6D_6): δ 183.9 (dd, $^1J_{PC}$ = 102.6 Hz, $^1J_{PC}$ = 80.8 Hz, *C*=N), 151.4 (dd, $^3J_{PC}$ = 30.0 Hz, $^3J_{PC}$ = 10.9 Hz, *ipso*-C), 128.4 (s, *ortho*-C), 123.6 (s, *para*-CH), 118.0 (s, *meta*-CH), 47.5 (d, $^2J_{PC}$ = 4.5 Hz, N*C*H), 39.4 (d, $^2J_{PC}$ = 3.6 Hz, N*C*H$_2$), 25.5 (dd, $^1J_{PC}$ = 19.1 Hz, $^3J_{PC}$ = 1.8 Hz, *C*H), 24.5 (dd, $^1J_{PC}$ = 18.2 Hz, $^3J_{PC}$ = 2.7 Hz, *C*H), 24.1 (d, $^3J_{PC}$ = 3.6 Hz, NCH*CH$_3$*), 23.6 (d, $^3J_{PC}$ = 3.6 Hz, NCH*CH$_3$*), 23.0 (d, $^2J_{PC}$ = 16.3 Hz, CH*CH$_3$*), 22.8 (d, $^2J_{PC}$ = 17.3 Hz, CH*CH$_3$*), 20.9 (d, $^2J_{PC}$ = 18.2 Hz, CH*CH$_3$*), 20.8 (d, $^2J_{PC}$ = 18.2 Hz, CH*CH$_3$*), 13.7 (d, $^3J_{PC}$ = 2.7 Hz, CH$_2$*CH$_3$*).

Preparation of 6d

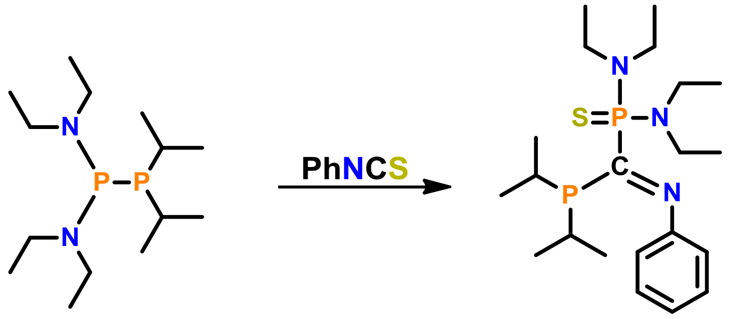

6 **6d**

To a solution of **6** (146 mg, 0.500 mmol) in toluene (5 mL) an excess of PhNCS (0.07 mL, 81 mg, 0.600 mmol) was added dropwise at room temperature. The solution was stirred for 24 hours. $^{31}P\{^1H\}$ NMR of the orange reaction mixture revealed the complete conversion of **6** into **6d**. The solvent was evaporated, and the residue was dried under a vacuum (0.01 Torr) at 50°C to remove all volatiles. The oily product slowly solidifies at -20°C to afford orange X-ray quality crystals of **6d,** which melt at room temperature. Yield 95% (204 mg, 0.477 mmol).

NMR:

$^{31}P\{^1H\}$ NMR (C_6D_6): δ 65.1 (broad s, S=P($Et_2N)_2$), 23.4 (broad s, P*i*Pr_2).

^1H NMR (C_6D_6): δ 7.15 (m, 2H, *o*-CH, overlapped with C_6D_6 residual peak), 6.90 (m, 2H, *m*-CH), 6.89 (m, 1H, *p*-CH), 3.27 (m, 4H, CH_2), 3.16 (m, 2H, C*H*), 2.98 (m, 4H, CH_2), 1.20 (dd, $^3J_{PH}$ = 15.1 Hz, $^3J_{HH}$ = 7.0 Hz, 6H, CHCH_3), 1.08 (dd, $^3J_{PH}$ = 14.0 Hz, $^3J_{HH}$ = 7.2 Hz, 6H, CHCH_3), 1.02 (t, $^3J_{HH}$ = 7.0 Hz, 12H, CH_2CH_3).

$^{13}C\{^1H\}$ NMR (C_6D_6): δ* 151.7 (dd, $^3J_{PC}$ = 27.2 Hz, $^3J_{PC}$ = 11.8 Hz, *ipso*-C), 128.4 (s, *ortho*-C), 123.7 (s, *meta*-CH), 118.4 (s, *para*-CH), 39.4 (broad s, *C*H_2), 24.9 (d, $^1J_{PC}$ = 15.4 Hz, *C*H), 22.4 (d, $^2J_{PC}$ = 19.1 Hz, CHCH_3), 20.6 (d, $^2J_{PC}$ = 19.1 Hz, CHCH_3), 13.7 (d, $^3J_{PC}$ = 2.7 Hz, CH_2CH_3). *The tertiary carbon atom C=N was not detected in the $^{13}C\{1H\}$ NMR.

IR (solid): $\tilde{\upsilon}$ = 2972, 2947, 2927, 2865, 1575 (**C=N**), 1563 (**C=N**), 1478, 1457, 1446, 1375, 1362, 1345, 1295, 1198, 1168, 1099, 1065, 1014, 946, 933, 919, 878, 803, 776, 764, 705, 695, 686, 657, 642, 569, 558, 506, 463.

Preparation of 7d

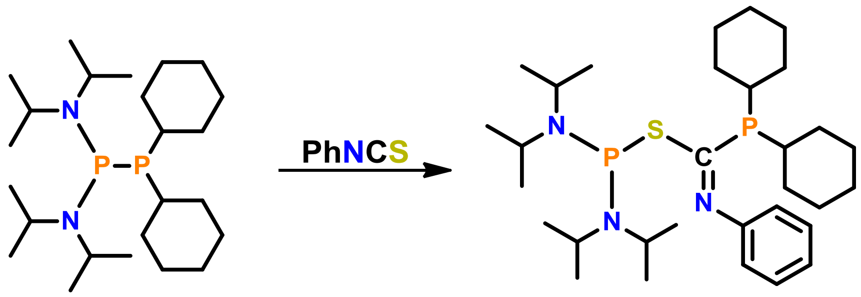

7 **7d/7d'**

To a solution of **7** (214 mg, 0.500 mmol) in toluene (5 mL) an excess of PhNCS (0.07 mL, 81 mg, 0.600 mmol) was added dropwise at room temperature. The solution was stirred for 3 days. $^{31}P\{^1H\}$ NMR of the orange reaction mixture revealed the complete conversion of **7** into **7d** and **7d'**. The solvent was evaporated, and the residue was dried under a vacuum (0.01 Torr) at 50°C to remove all volatiles. The oily product slowly solidifies at -20°C to afford yellow X-ray quality crystals of **7d/7d'**. Yield 99% (279 mg, 0.495 mmol). The isolated product contains conformational isomers **7d** and **7d'** in a molar ratio of 1.55:1.

NMR:

$^{31}P\{^1H\}$ NMR (toluene-d$_8$, 273K): **7d**: δ 97.7 (d, $^3J_{PP}$ = 130.8 Hz, P(*i*Pr$_2$N)$_2$), 16.8 (d, $^3J_{PP}$ = 130.8 Hz, PCy$_2$). **7d'**: δ 98.9 (broad s, P(*i*Pr$_2$N)$_2$), 3.8 (broad s, PCy$_2$).

^1H NMR (toluene-d$_8$, 273K): δ 7.22-7.18 (overlapped m, 4H, *o*-C*H* of **7d** and **7d'**), 7.00-6.90 (overlapped m, 6H, *m,p*-C*H* of **7d** and **7d'**), 3.61 (m, 4H, NC*H* of **7d'**), 3.39 (m, 4H, NC*H* of **7d**), 2.60 (m, 2H, C*H* of **7d**), 2.42 (m, 2H, C*H$_2$* of **7d**), 2.30 (m, 2H, C*H* of **7d**), 2.27-2.16 (m, 2H, CH$_2$ of **7d**), 1.90-1.33 (overlapped m, 36H, C*H*$_2$ of **7d** and **7d'**), 1.38 (d, $^3J_{HH}$ = 6.4 Hz, 12H, C*H$_3$* of **7d'**), 1.28 (d, $^3J_{HH}$ = 6.6 Hz, 12H, C*H$_3$* of **7d**), 1.21 (d, $^3J_{HH}$ = 6.5 Hz, 12H, C*H$_3$* of **7d'**), 1.12 (d, $^3J_{HH}$ = 6.7 Hz, 12H, C*H$_3$* of **7d**).

$^{13}C\{^1H\}$ NMR (toluene-d$_8$, 273K): 7d: δ 171.2 (dd, $^1J_{PC}$ = 42.7 Hz, $^2J_{PC}$ = 8.2 Hz, *C*=N), 152.3 (d, $^3J_{PC}$ = 2.7 Hz, *ipso*-C), 128.5 (s, *ortho*-C), 123.4 (s, *para*-CH), 120.1 (s, *meta*-CH), 47.7 (d, $^2J_{PC}$ = 12.7 Hz, N*C*H), 34.4 (dd, $^1J_{PC}$ = 17.3 Hz, $^4J_{PC}$ = 6.4 Hz, *C*H), 31.4 (d, $^2J_{PC}$ = 17.3 Hz, *C*H$_2$), 28.7 (d, $^3J_{PC}$ = 8.2 Hz, *C*H$_2$), 27.7 (d, $^3J_{PC}$ = 6.4 Hz, *C*H$_2$), 27.6 (d, $^2J_{PC}$ = 13.6 Hz, *C*H$_2$), 26.8 (s, *C*H$_2$), 23.7 (d, $^3J_{PC}$ = 5.5 Hz, *C*H$_3$), 23.5 (d, $^3J_{PC}$ = 9.1 Hz, *C*H$_3$). **7d':** δ 174.9 (dd, $^1J_{PC}$ = 59.0 Hz, $^2J_{PC}$ = 6.4 Hz, *C*=N), 152.8 (d, $^3J_{PC}$ = 14.5 Hz, *ipso*-C), 128.3 (s, *ortho*-C), 122.6 (s, *para*-CH), 120.4 (s, *meta*-CH), 48.2 (d, $^2J_{PC}$ = 12.7 Hz, N*C*H), 36.3 (d, $^1J_{PC}$ = 17.3 Hz, *C*H), 30.9 (d, $^2J_{PC}$ = 18.2 Hz, *C*H$_2$), 30.5 (d, $^2J_{PC}$ = 11.8 Hz, *C*H$_2$), 27.2 (d, $^3J_{PC}$ = 10.0 Hz, *C*H$_2$), 26.8 (d, $^3J_{PC}$ = 13.6 Hz, *C*H$_2$), 26.4 (s, *C*H$_2$), 23.9 (d, $^3J_{PC}$ = 5.4 Hz, *C*H$_3$), 23.2 (d, $^3J_{PC}$ = 9.1 Hz, *C*H$_3$).

Elemental analysis: calcd. for $C_{31}H_{55}N_3P_2S$: C, 66.04; H, 9.83; N, 7.45; S, 5.69. Found: C, 65.90; H, 9.847; N, 7.42; S, 5.691.

IR (solid): $\tilde{\upsilon}$ = 2965, 2922, 2848, 1560 (**C=N**), 1483, 1444, 1381, 1363, 1195, 1175, 1156, 1114, 1019, 996, 948, 912, 879, 863, 851, 753, 688, 529, 512.

Formation of 8d

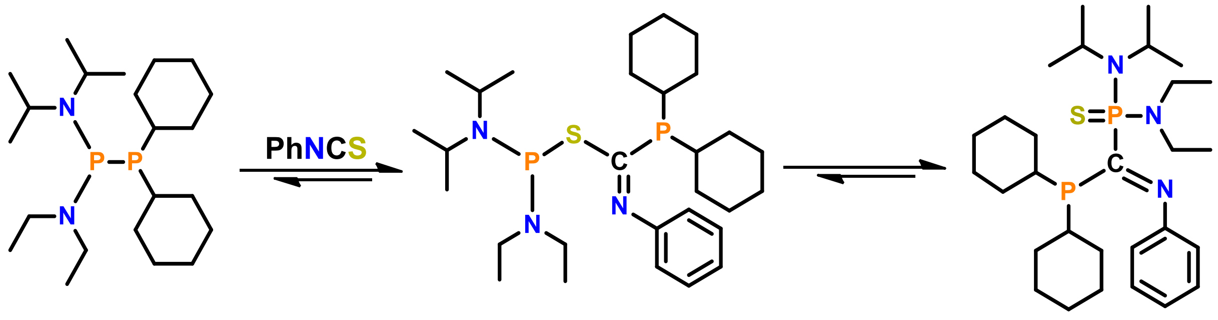

To a solution of **8** (200 mg, 0.500 mmol) in C_6D_6 (4 mL) an excess of PhNCS (0.12 mL, 135 mg, 1.000 mmol) was added at room temperature. The reaction progress was monitored by $^{31}P\{^1H\}$ and ^1H NMR, and after stirring the solution for 7 days it revealed the complete conversion of **8** into the mixture of **8d*, 8d**** and **8d** (see Figure 119). Complete rearrangement of **8*** and **8d**** into the final product **8d** was observed after 45 days. Product was not isolated as the formation of **8d** is reversible in the absence of PhNCS – after evaporation of PhNCS, there is established an equilibrium between **8**, **8d***, **8d**** and **8d**.

NMR:

$^{31}P\{^1H\}$ NMR (C_6D_6): δ 65.6 (d, $^2J_{PP}$ = 43.6 Hz, S=P(Et_2N)(*i*Pr_2N)), 17.2 (d, $^2J_{PP}$ = 43.6 Hz, PCy_2).

^1H NMR (C_6D_6): δ 7.18 (m, 2H, *o*-CH), 6.91 (m, 1H, *p*-CH), 6.86 (m, 2H, *m*-CH), 3.84 (sept, $^3J_{HH}$ = 7.1 Hz, 2H, NC*H*), 3.58 (m, 2H, C*H*$_2$), 3.10 (m, 2H, C*H*$_2$), 2.85 (m, 2H, C*H*), 1.94 (m, 4H, CH_2), 1.70-1.54 (overlapped m, 6H, CH_2), 1.44-1.11 (overlapped m, 10H, CH_2), 1.40 (d, $^3J_{HH}$ = 7.1 Hz, 6H, CHC*H*$_3$), 1.38 (d, $^3J_{HH}$ = 7.1 Hz, 6H, CHC*H*$_3$), 1.06 (t, $^3J_{HH}$ = 7.0 Hz, 6H, CH_2C*H*$_3$).

$^{13}C\{^1H\}$ NMR (C_6D_6): δ 184.2 (dd, $^1J_{PC}$ = 102.6 Hz, $^1J_{PC}$ = 81.7 Hz, *C*=N), 151.6 (dd, $^3J_{PC}$ = 30.0 Hz, $^3J_{PC}$ = 10.0 Hz, *ipso*-C), 128.5 (s, *ortho*-C), 123.6 (s, *para*-CH), 117.9 (s, *meta*-CH), 47.5 (d, $^2J_{PC}$ = 5.4 Hz, N*C*H), 39.3 (d, $^2J_{PC}$ = 2.7 Hz, N*C*H2), 36.0 (dd, $^1J_{PC}$ = 19.1 Hz, $^3J_{PC}$ = 1.8 Hz, *C*H), 34.8 (dd, $^1J_{PC}$ = 18.2 Hz, $^3J_{PC}$ = 2.7 Hz, *C*H), 32.8 (d, $^2J_{PC}$ = 13.6 Hz, *C*H_2), 32.6 (d, $^2J_{PC}$ = 13.6 Hz, *C*H_2), 31.3 (d, $^2J_{PC}$ = 7.3 Hz, *C*H_2), 31.1 (d, $^2J_{PC}$ = 7.3 Hz, *C*H_2), 27.3 (d, $^3J_{PC}$ = 3.6 Hz, *C*H_2), 27.2 (d, $^3J_{PC}$ = 3.6 Hz, *C*H_2), 27.1 (d, $^3J_{PC}$ = 10.0 Hz, *C*H_2), 27.0 (d, $^3J_{PC}$ = 10.0 Hz, *C*H_2), 26.3 (s, *C*H_2), 26.2 (s, *C*H_2), 24.1 (d, $^3J_{PC}$ = 4.5 Hz, CH*C*H_3), 23.6 (d, $^3J_{PC}$ = 4.5 Hz, CH*C*H_3), 13.6 (d, $^3J_{PC}$ = 2.7 Hz,CH_2*C*H_3).

Preparation of 9d

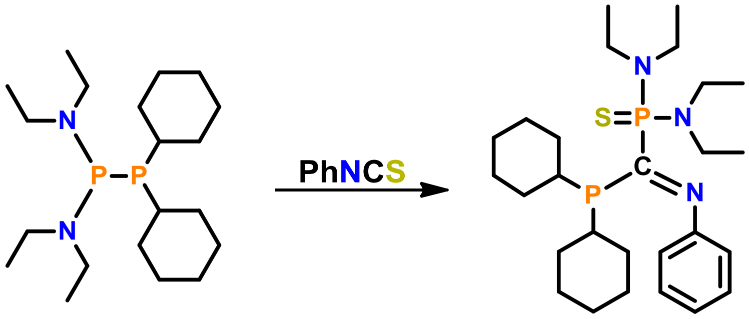

9 **9d**

To a solution of **9** (186 mg, 0.500 mmol) in toluene (5 mL) an excess of PhNCS (0.07 mL, 81 mg, 0.600 mmol) was added dropwise at room temperature. The solution was stirred for 24 h. $^{31}P\{^{1}H\}$ NMR of the orange reaction mixture revealed the complete conversion of **9** into **9d**. The solvent was evaporated, and the residue was dried under a vacuum (0.01 Torr) at 50°C to remove all volatiles. The oily product slowly solidifies at -20°C to afford yellow X-ray quality crystals of **9d**. Yield 97% (247 mg, 0.487 mmol).

NMR:

$^{31}P\{^{1}H\}$ NMR (C_6D_6): δ 65.8 (broad s, S=P($Et_2N)_2$), 13.6 (broad s, PCy_2).

^{1}H NMR (C_6D_6): δ 7.18 (m, 2H, *o*-CH), 6.91 (m, 2H, *m*-CH), 6.90 (m, 1H, *p*-CH), 3.36 (m, 4H, C*H₂*), 3.01 (m, 4H, C*H₂*), 3.01 (m, 2H, C*H*, overlapped by NCH_2) 1.99 (m, 4H, CH_2), 1.73-1.58 (overlapped m, 6H, CH_2), 1.42-1.12 (overlapped m, 10H, CH_2), 1.05 (t, $^{3}J_{HH}$ = 7.0 Hz, 12H, CH_2CH_3).

$^{13}C\{^{1}H\}$ NMR (C_6D_6): δ* 151.9 (dd, $^{3}J_{PC}$ = 28.2 Hz, $^{3}J_{PC}$ = 11.8 Hz, *ipso*-C), 128.4 (s, *ortho*-C), 123.7 (s, *meta*-CH), 118.3 (s, *para*-CH), 39.2 (broad s, N*C*H₂), 35.1 (d, $^{1}J_{PC}$ = 19.1 Hz, *C*H), 32.5 (d, $^{2}J_{PC}$ = 16.4 Hz, *C*H₂), 31.1 (d, $^{2}J_{PC}$ = 18.2 Hz, *C*H₂), 27.2 (d, $^{3}J_{PC}$ = 11.8 Hz, *C*H₂), 27.0 (d, $^{3}J_{PC}$ = 10.9 Hz, *C*H₂), 26.3 (s, *C*H₂), 13.6 (d, $^{3}J_{PC}$ = 2.7 Hz, *C*H₃). *The tertiary carbon atom C=N was not detected in the ^{13}C\{1H\} NMR.

Elemental analysis: calcd. for $C_{27}H_{47}N_3P_2S$: C, 63.87; H, 9.33; N, 8.28; S, 6.32. Found: C, 63.62; H, 9.140; N, 8.37; S, 6.525.

IR (solid): $\tilde{\nu}$ = 2972, 2918, 2847, 1588 (**C=N**), 1477, 1446, 1378, 1292, 1196, 1171, 1071, 1023, 1012, 940, 906, 882, 851, 793, 775, 761, 707, 697, 637, 498.

Preparation of 10d

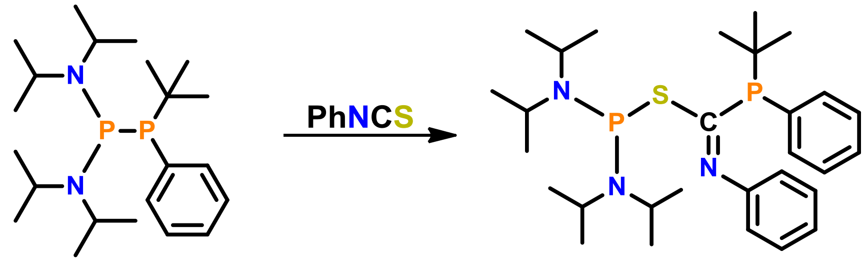

10 **10d**

To a solution of **10** (198 mg, 0.500 mmol) in toluene (5 mL) an excess of PhNCS (0.07 mL, 81 mg, 0.600 mmol) was added dropwise at room temperature. The solution was stirred for 24 hours. $^{31}P\{^{1}H\}$ NMR of the orange reaction mixture revealed the complete conversion of **10** into **10d**. The solvent was evaporated, and the residue was dried under a vacuum (0.01 Torr) at 50°C to remove all volatiles. The oily product slowly solidifies at -20°C to afford yellow X-ray quality crystals of **10d**. Yield 97% (259 mg, 0.487 mmol).

NMR:

$^{31}P\{^{1}H\}$ NMR (C_6D_6): δ 101.0 (d, $^{3}J_{PP}$ = 130.8 Hz, P(*i*Pr$_2$N)$_2$), 25.0 (d, $^{3}J_{PP}$ = 130.8 Hz, P*t*BuPh).

^{1}H NMR (C_6D_6): δ 7.88 (m, 2H, *o*-CH of P*t*BuPh), 7.24 (m, 2H, *o*-CH of N-Ph), 7.23 (m, 2H, *m*-CH of P*t*BuPh), 7.17 (m, 1H, *p*-CH of P*t*BuPh), 7.10 (m, 2H, *m*-CH of N-Ph), 6.95 (m, 1H, *p*-CH of N-Ph), 3.54 (sept, $^{3}J_{HH}$ = 6.6 Hz, 2H, C*H*), 3.06 (broad m, 2H, C*H*), 1.36 (d, $^{3}J_{PH}$ = 12.6 Hz, 9H, C(C*H$_3$*)$_3$), 1.33 (d, $^{3}J_{HH}$ = 6.6 Hz, 6H, CHC*H$_3$*), 1.06 (d, $^{3}J_{HH}$ = 6.6 Hz, 6H, CHC*H$_3$*), 0.94 (d, $^{3}J_{HH}$ = 6.7 Hz, 6H, CHC*H$_3$*), 0.76 (d, $^{3}J_{HH}$ = 6.7 Hz, 6H, CHC*H$_3$*).

$^{13}C\{^{1}H\}$ NMR (C_6D_6): δ 170.2 (dd, $^{1}J_{PC}$ = 39.0 Hz, $^{2}J_{PC}$ = 6.3 Hz, *C*=N), 151.5 (d, $^{3}J_{PC}$ = 4.5 Hz, *ipso*-C of N-Ph), 136.6 (d, $^{2}J_{PC}$ = 20.0 Hz, *ortho*-C of P*t*BuPh), 135.1 (d, $^{1}J_{PC}$ = 15.4 Hz, *ipso*-C of P*t*BuPh), 128.9 (s, *para*-*C*H of P*t*BuPh), 128.6 (s, *meta*-CH of P*t*BuPh), 127.7 (d, $^{4}J_{PC}$ = 8.2 Hz, *ortho*-C of N-Ph), 123.6 (s, *para*-CH of N-Ph), 120.4 (s, *meta*-CH of N-Ph), 47.7 (d, $^{2}J_{PC}$ = 12.7 Hz, N*C*H), 47.4 (d, $^{2}J_{PC}$ = 12.7 Hz, N*C*H), 32.8 (dd, $^{1}J_{PC}$ = 18.2 Hz, $^{4}J_{PC}$ = 9.1 Hz, *C*(CH$_3$)$_3$), 27.3 (d, $^{2}J_{PC}$ = 13.6 Hz, C(*CH$_3$*)$_3$), 23.9 (d, $^{3}J_{PC}$ = 4.5 Hz, CH*CH$_3$*), 23.4 (d, $^{3}J_{PC}$ = 5.4 Hz, CH*CH$_3$*), 23.3 (d, $^{3}J_{PC}$ = 5.4 Hz, CH*CH$_3$*), 23.0 (d, $^{3}J_{PC}$ = 9.1 Hz, CH*CH$_3$*).

Elemental analysis: calcd. for $C_{29}H_{47}N_3P_2S$: C, 65.51; H, 8.91; N, 7.90; S, 6.03. Found: C, 65.48; H, 8.809; N, 7.81; S, 5.972.

IR (solid): $\tilde{\upsilon}$ = 2963, 2925, 2865, 1577 (**C=N**), 1484, 1454, 1433, 1385, 1363, 1193, 1175, 1156, 1127, 1114, 1069, 1019, 944, 913, 880, 863, 803, 757, 738, 692, 581, 526, 509, 491, 445.

Reactivity of diphosphanes towards CO_2 and CS_2

We found that only diphosphanes of the general formula $(R_2N)_2PP$*t*Bu_2, which bear a highly nucleophilic P*t*Bu_2 fragment (**1**, **2**), insert CO_2 molecules between the P-P bonds in the quantitative reaction in the presence of the BBh_3 catalyst. However, as the bulkiness of the $(R_2N)_2P$ moiety decreases the reactivity changes. The increasing size of $(R_2N)_2P$ causes the elongation of the P-P bond, which facilitates the insertion of the CO_2 molecule. Hence, only **1** gives the stable product **1a,** while **2a** regenerates to parent **2** in the absence of a CO_2 atmosphere. Analogous diphosphination products were not obtained in the reaction involving $(Et_2N)_2PP$*t*Bu_2 (**3**) or other diphosphanes. The reversibly formed product **2a** may be stabilized by complexation with CODPt$(CH_3)_2$. It forms a five-membered metallacycle **2a$_{Pt}$**, which is both air- and moisture stable and has the same structural features as the previously described Pt complex of **1a**.[2] Interestingly, in the ^{31}P NMR spectra of reaction mixtures of **4** and **7** performed under a CO_2 atmosphere, we observed the formation of **4a** and **7a**, respectively, but only in approximately 20% yield. Hence, we assume that diphosphane has to meet both criteria to efficiently bind and functionalize CO_2 molecules: it possesses a highly nucleophilic **P**RR' atom (resulting from the electron-donating groups R and R'), which provides kinetic accessibility of the conversion and will be thermodynamically privileged only when both PR_2 fragments feature bulky substituents, which leads to the significant elongation of the P-P bond. Since species bearing both $(Et_2N)_2P$ or (Et_2N)(*i*$Pr_2N)P$ on one side and P*t*BuPh or PPh_2 on the other side of the P-P bond do not satisfy any of these conditions, they are the least reactive species; they do not react with either CO_2 or CS_2 (Scheme S1). Considering reactions with CS_2, one may note that the range of diphosphanes giving the corresponding P-S-C(=S)-P product is much broader, and their formation is much more thermodynamically privileged than that of their CO_2 analogues. Indeed, diphosphanes can be less bulky, have a less nucleophilic PRR' atom, and still, efficiently add to the C=S bond. All (*i*$Pr_2N)_2P$ (group **A**) species but one, the least nucleophilic **13**, react quantitatively with CS_2 to form stable products (**1b**, **4b**, **7b,** and **10b**). The reaction of **13** with an excess of CS_2 leads to an equilibrium mixture of **13** and **13b** and contains mostly reactants. However, complete conversion of substrates into products is relatively slow (1-3 days), with a maximum value for the most congested **1**. Moreover, we found that the sterically crowded (*i*$Pr_2N)_2P$ fragment not only accounts for elongation of the P-P bond facilitating insertion of CS_2 but also precludes rearrangement and/or decomposition of (*i*$Pr_2N)_2P$-C(=S)-S-PRR' products. Conversely, systems with decreased steric bulk of $(R_2N)_2P$: (Et_2N)(*i*$Pr_2N)P$ (group **B**) and $Et_2N)_2P$ (group **C**) retained the highly nucleophilic character of the phosphorus atom in the PRR' counterpart; P*t*Bu_2, P*i*Pr_2, PCy_2, yielded respective $(RR'N)_2P$-C(=S)-S-PRR' products within minutes; however, they are not stable and tend to rearrange to other unidentified compounds. Hence, we assume these adducts may be regarded as kinetically accessible intermediates that rearrange to other, presumably more thermodynamically privileged derivatives (Figure S1).

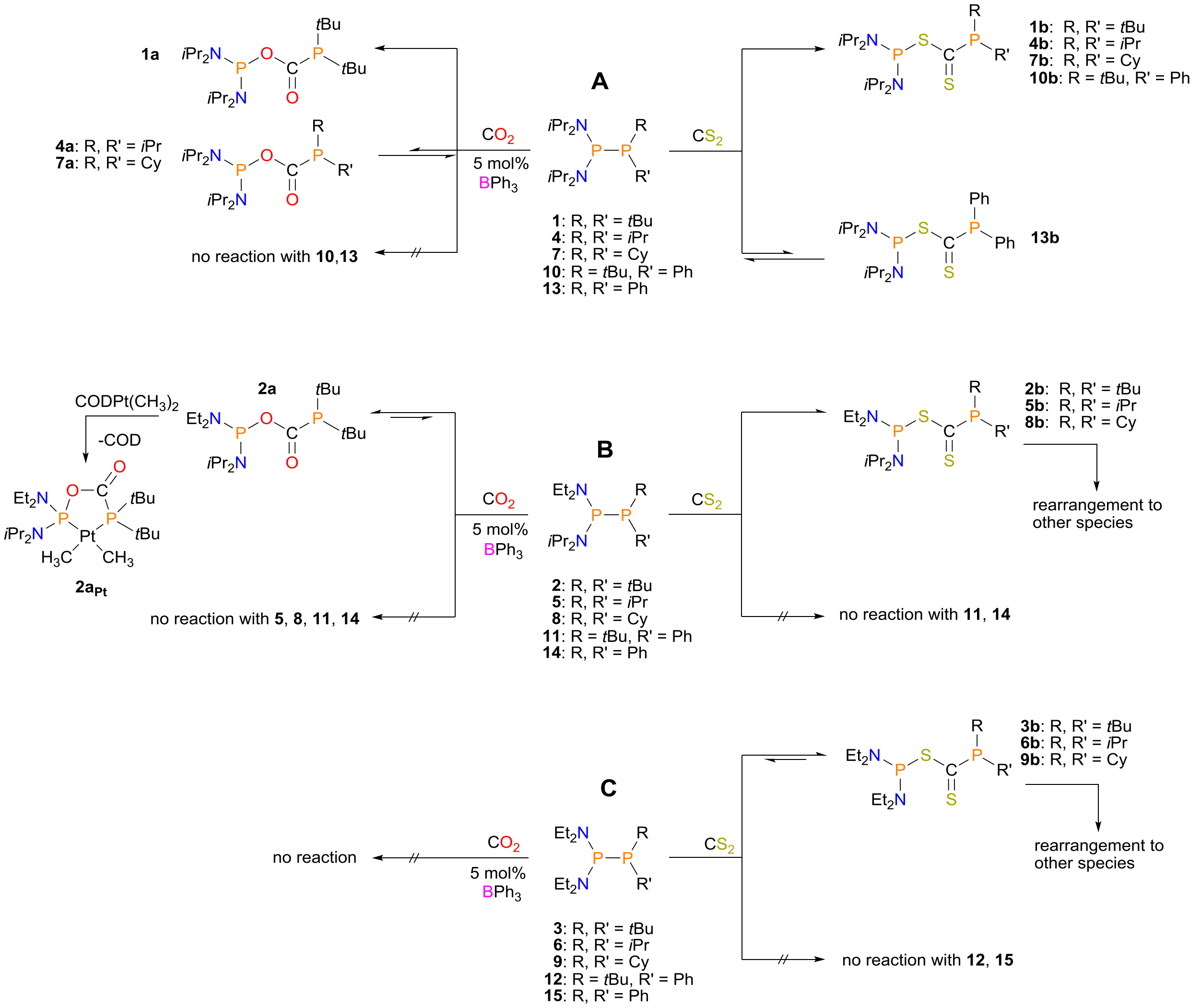

SCHEME. S1. THE REACTIONS OF UNSYMMETRICAL DIPHOSPHANES WITH CO_2 AND CS_2

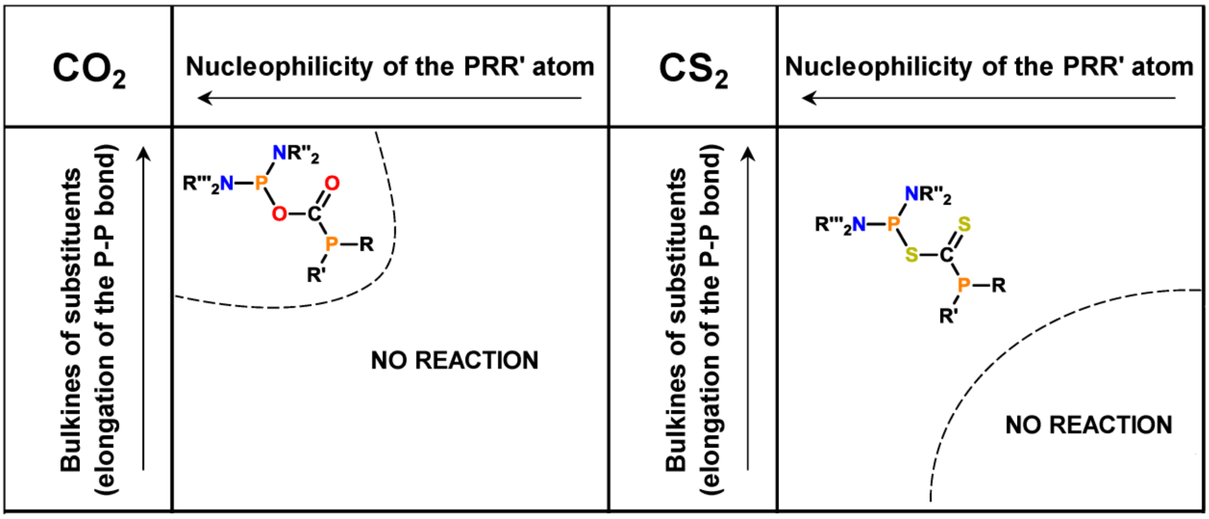

FIG. S1. FACTORS GUIDING REACTIVITY OF 1,1-DIAMINODIPHOSPHANES TOWARDS CO_2 AND CS_2

X-ray structures analysis

General methods

The X-ray diffraction data were collected on an IPDS 2T dual-beam diffractometer (STOE&Cie GmbH, Darmstadt, Germany) at 120.0(2) K with Mo-K_{α} radiation of a microfocus X-ray source (GeniX 3D Mo HighFlux, Xenocs, Sassenage, France, 50 kV, 1.0 mA, λ = 0.71069 Å)for **1d, 2d, 3c, 3d, 4, 4b, 4d, 6c, 7, 7b, 9d, 10b** and **10d**. For structures **1c, 2aPt**, **6d, 7d, 9c** we used Cu-K_{α} radiation of a microfocus X-ray source (GeniX 3D Cu HighFlux, Xenocs, 50 kV, 0.6 mA, λ = 1.54186 Å), the choice was dictated by temporary unavailability (failure) of the Mo lamp. Every crystal was thermostated in nitrogen stream at 120 K using CryoStream-800 device (Oxford CryoSystem, UK) during the entire experiment. Data collection and data reduction were controlled by X-Area 1.75 program.[3] The structure was solved by the SHELXT method[4,5] and refined using the program packages Olex2[6,7] and SHELX-2015.[4,5] All non-hydrogen atoms were modeled as anisotropic, all H-atoms were refined as isotropic. Hydrogen atoms were placed in idealized positions and refined with usual restraints of the riding model.

Crystallographic data for all structures reported in this paper have been deposited with the Cambridge Crystallographic Data Centre as supplementary publication No. CCDC 2068168-2068185. The data can be obtained free of charge from The Cambridge Crystallographic Data Centre via www.ccdc.cam.ac.uk/structures.

Specific details for individual structures

Most of the structures contain one molecule in the asymmetric unit (Z' = 1), however two independent molecules are found in structures **1d**, **3d**, and **6c**. Diffraction pattern recorded for **1d** was difficult to process. One cell axis was long (*c* > 51 Å), which caused overlap of reflections in our IPDS system, hard to resolve despite very narrow scanning angle, set to 0.4° (usually use of 1° is sufficient) and long detector distance. By turning overlap checking on, some reflections, not separable, were excluded by the data processing program, leading to lowered completeness. If we had turned off the checking, the R_{int} would have increased to unreasonable values. Structure **$2a_{Pt}$** was refined in the chiral group $P2_1$, Flack x = 0.00(3), and one tert-butyl group was found disordered over two positions with site occupation factors of 0.68(6)/0.32(6). Structure of **3d** was refined as a non-merohedral twin with the domain's mass fraction equal to 0.492(4), using HKLF 5 instruction under SHELXL. The numerical multi-scan absorption correction was performed for all structures, investigated using the copper radiation, and for some structures using Mo radiation when necessary (see Tables S1-S6). Due to the construction of IPDS 2T two-circle diffractometer (and reasonable time limits), all structures with $P\underline{1}$ space groups symmetry have lower than full data completeness for experiments with copper radiation (image plate detector theta = 30 deg). Majority of crystal structures was determined by Jarosław Chojnacki, except from **4** and **7**, which were determined by Łukasz Ponikiewski.

Structures **1d**, **3d**, **4d**, **7**, **7b**, **9c** show high values of wR^2 > 0.2. Not the best structure quality indicators probably result from some unresolved minor disorder, modulations or twinning, naturally occurring in real crystals. We have presented our interpretation of these problems in CIF files in VRF fields, but we repeat it also here. Hence, in **1d,** the source of the problem stems from extra-long unit cell parameter *c* > 50Å, causing partial reflections

overlapping as mentioned above. For such large structures, the quality indicator limits need to be slightly relaxed. Structure **3d** shows deteriorated quality despite applying twinning in HKLF 5 option, which improved the original, primary solution. Structure **4d** is just above the threshold $wR^2 > 0.2018$, so we left the data unchanged. In the case of structure **7** electron density map is probably affected by a disorder of the whole molecule (i.e. original and inverted molecule), which is hard to model. The second part gives a very low contribution to the electron density but negatively influences refinement indicators. Crystals of **7b** were small, and all specimens had very low scattering power. The frame exposition time was set to the highest practical limit (5 minutes). Structure **9c** due to low scattering power was determined using Cu radiation and long frame exposition time, but still, R_{int} indicator was high ca 7%. The results are the best we could get from the experimental data.

TABLE S1. CRYSTAL DATA AND STRUCTURE REFINEMENT FOR **1A**, **1D** AND **2A$_{Pt}$**

	**1c**	**1d**	**2a$_{Pt}$**
CCDC	2068168	2068169	2068170
Empirical formula	$C_{27}H_{51}N_3OP_2$	$C_{27}H_{51}N_3P_2S$	$C_{21}H_{48}N_2O_2P_2Pt$
M_r [g mol^{-1}]	495.64	511.70	617.64
Crystal system	Monoclinic	Monoclinic	Monoclinic
Space group	$P2_1/n$	$P2_1/n$	$P2_1$
a [Å]	9.3602(3)	10.7908(2)	8.2748(8)
b [Å]	26.8331(7)	11.1769(2)	14.9152(14)
c [Å]	12.0410(4)	51.0241(10)	10.8405(7)
α [°]	90	90	90
β [°]	105.403(3)	96.126(1)	99.900(6)
γ [°]	90	90	90
V [Å^3]	2915.63(16)	6118.8(2)	1318.0(2)
Z	4	8	2
Calculated density [Mg m^{-3}]	1.129	1.111	1.556
T [K]	120	120	120
μ [mm^{-1}]	1.52	0.23	11.23
Crystal size/mm^3	0.45 × 0.24 × 0.18	0.41 × 0.12 × 0.11	0.32 × 0.13 × 0.09
λ [Å]	1.54186 (CuKα)	0.71073 (MoKα)	1.54186 (CuKα)
F(000)	1088	2240	624
S	1.07	1.04	1.08
R_{int}	0.021	0.084	0.028
No. of measured, independent, observed [I > 2σ(I)] reflections	13049, 5080, 4809	21744, 9514, 7418	11255, 4445, 4433
$R[F^2 > 2\sigma(F^2)]$	0.040	0.096	0.051
$wR(F^2)$	0.106	0.279	0.136
Largest diff. peak/hole / e Å^{-3}	0.31/-0.34	1.04/-0.55	1.16/-1.66

TABLE S2. CRYSTAL DATA AND STRUCTURE REFINEMENT FOR **2D**, **3C** AND **3D**

	**2d**	**3c**	**3d**
CCDC	2068171	2068172	2068173
Empirical formula	$C_{25}H_{47}N_3P_2S$	$C_{30}H_{48}N_4O_2P_2$	$C_{30}H_{48}N_4P_2S_2$
M_r [g mol^{-1}]	483.65	558.66	590.78
Crystal system	Monoclinic	Triclinic	Triclinic
Space group	$P2_1/c$	P-1	P-1
a [Å]	9.6368(7)	10.0510(5)	11.7372(5)
b [Å]	31.250(3)	10.2266(5)	16.1864(7)
c [Å]	10.7380(9)	16.5703(8)	17.6860(9)
α [°]	90	95.147(4)	98.506(4)
β [°]	116.104(6)	97.578(4)	101.051(4)
γ [°]	90	109.585(4)	90.046(3)
V [Å^3]	2903.9(4)	1574.23(14)	3260.0(3)
Z	4	2	4
Calculated density [Mg m^{-3}]	1.106	1.179	1.204
T [K]	120	120	120
μ [mm^{-1}]	0.24	0.17	0.29
Crystal size/mm^3	0.12 × 0.06 × 0.05	0.46 × 0.23 × 0.19	0.33 × 0.25 × 0.18
λ [Å]	0.71073 (MoKα)	0.71073 (MoKα)	0.71073 (MoKα)
F(000)	1056	604	1272
S	1.08	1.08	1.44
R_{int}	0.03	0.04	-
No. of measured, independent, observed [I > 2σ(I)] reflections	15355, 6331, 5205	21258, 8465, 7189	11655, 11655, 8280
$R[F^2 > 2\sigma(F^2)]$	0.041	0.053	0.129
$wR(F^2)$	0.114	0.152	0.375
Largest diff. peak/hole / e Å^{-3}	0.41/-0.42	0.60/-0.44	2.81/-1.54

TABLE S3. CRYSTAL DATA AND STRUCTURE REFINEMENT FOR **4**, **4B** AND **4D**

	**4**	**4b**	**4d**
CCDC	2068174	2068175	2068176
Empirical formula	$C_{18}H_{42}N_2P_2$	$C_{19}H_{42}N_2P_2S_2$	$C_{25}H_{47}N_3P_2S$
M_r [g mol^{-1}]	348.47	424.60	483.65
Crystal system	Triclinic	Monoclinic	Monoclinic
Space group	P-1	$P2_1/n$	$P2_1/n$
a [Å]	9.3130(6)	10.6190(5)	9.1018(5)
b [Å]	10.5225 (7)	10.1850(4)	22.0959(7)
c [Å]	13.1467(9)	23.4058(10)	14.3481(7)
α [°]	93.361(6)	90	90
β [°]	108.423(5)	92.801(4)	97.444(4)
γ [°]	115.803(5)	90	90
V [Å^3]	1071.01(13)	2528.42(19)	2061.3(2)
Z	2	4	4
Calculated density [Mg m^{-3}]	1.081	1.115	1.123

T [K]	120	120	120
μ [mm^{-1}]	0.20	0.34	0.24
Crystal size/mm^3	0.57 × 0.32 × 0.28	0.16 × 0.15 × 0.05	0.24 × 0.08 × 0.06
λ [Å]	0.71073 (MoKα)	0.71073 (MoKα)	0.71073 (MoKα)
F(000)	388	928	1056
S	1.06	1.00	1.02
R_{int}	0.034	0.053	0.094
No. of measured, independent, observed [I > 2σ(I)] reflections	10994, 5720, 4730	15945, 6753, 4332	23552, 7728, 4202
$R[F^2 > 2\sigma(F^2)]$	0.036	0.047	0.076
$wR(F^2)$	0.102	0.116	0.202
Largest diff. peak/hole / e Å^{-3}	0.56/-0.27	0.39/-0.48	1.52/-0.60

TABLE S4. CRYSTAL DATA AND STRUCTURE REFINEMENT FOR **6C**, **6D** AND **7**

	**6c**	**6d**	**7**
CCDC	2068177	2068178	2068179
Empirical formula	$C_{28}H_{44}N_4O_2P_2$	$C_{21}H_{39}N_3P_2S$	$C_{24}H_{50}N_2P_2$
M_r [g mol^{-1}]	530.61	427.55	428.6
Crystal system	Monoclinic	Triclinic	Triclinic
Space group	$P2_1/c$	P-1	P-1
a [Å]	15.9319(2)	7.3770(2)	10.1568(5)
b [Å]	21.2399(3)	9.1768(3)	10.1692(5)
c [Å]	18.0048(2)	19.7041(7)	14.0942(8)
α [°]	90	79.286(3)	105.917(4)
β [°]	102.407(1)	86.835(3)	97.631(4)
γ [°]	90	66.673(2)	104.632(4)
V [Å^3]	5950.39(13)	1203.28(7)	1322.20(12)
Z	8	2	2
Calculated density [Mg m^{-3}]	1.185	1.180	1.077
T [K]	120	120	120
μ [mm^{-1}]	0.18	2.52	0.18
Crystal size/mm^3	0.52 × 0.28 × 0.26	0.34 × 0.11 × 0.09	0.47 × 0.24 × 0.21
λ [Å]	0.71073 (MoKα)	1.54186 (CuKα)	0.71073 (MoKα)
F(000)	2288	464	476
S	1.03	1.12	1.09
R_{int}	0.033	0.020	0.067
No. of measured, independent, observed [I > 2σ(I)] reflections	86395, 16088, 13435	10420, 3840, 3748	12946, 6981, 4693
$R[F^2 > 2\sigma(F^2)]$	0.035	0.042	0.068
$wR(F^2)$	0.094	0.114	0.221
Largest diff. peak/hole / e Å^{-3}	0.43/-0.40	0.31/-0.46	1.93/-0.38

TABLE S5. CRYSTAL DATA AND STRUCTURE REFINEMENT FOR **7B**, **7D** AND **9C**

	**7b**	**7d**	**9c**
CCDC	2068180	2068181	2068182
Empirical formula	$C_{25}H_{50}N_2P_2S_2$	$C_{31}H_{55}N_3P_2S$	$C_{34}H_{52}N_4O_2P_2$
M_r [g mol^{-1}]	504.73	563.78	610.73
Crystal system	Triclinic	Triclinic	Monoclinic
Space group	P-1	P-1	$P2_1/c$
a [Å]	9.8111(9)	10.2695(7)	10.0777(11)
b [Å]	11.5053(13)	10.6501(12)	13.5186(15)
c [Å]	13.7292(14)	15.8134(17)	25.847(3)
α [°]	87.016(9)	91.995(9)	90
β [°]	85.050(8)	94.153 (7)	98.004(9)
γ [°]	71.233(8)	106.537(7)	90
V [Å^3]	1461.4(3)	1650.9(3)	3487.1(7)
Z	2	2	4
Calculated density [Mg m^{-3}]	1.147	1.134	1.163
T [K]	120	120	120
μ [mm^{-1}]	0.31	1.95	1.39
Crystal size/mm^3	0.32 × 0.25 × 0.07	0.18 × 0.11 × 0.03	0.36 × 0.25 × 0.21
λ [Å]	0.71073 (MoKα)	1.54186 (CuKα)	1.54186 (CuKα)
F(000)	552	616	1320
S	0.97	1.05	1.25
R_{int}	0.152	0.027	0.075
No. of measured, independent, observed [I > 2σ(I)] reflections	16071, 7762, 2987	12595, 5389, 4403	18971, 5773, 5148
$R[F^2 > 2\sigma(F^2)]$	0.092	0.036	0.099
$wR(F^2)$	0.280	0.092	0.272
Largest diff. peak/hole / e Å^{-3}	0.43/-0.55	0.23/-0.26	0.78/-0.91

TABLE S6. CRYSTAL DATA AND STRUCTURE REFINEMENT FOR **9D**, **10B** AND **10D**

	**9d**	**10b**	**10d**
CCDC	2068183	2068184	2068185
Empirical formula	$C_{27}H_{47}N_3P_2S$	$C_{23}H_{42}N_2P_2S$	$C_{29}H_{47}N_3P_3S$
M_r [g mol^{-1}]	507.67	472.64	531.69
Crystal system	Monoclinic	Monoclinic	Triclinic
Space group	$P2_1/n$	$P2_1/n$	P-1
a [Å]	10.6476(5)	7.5525(7)	9.8758(7)
b [Å]	25.0357(15)	20.2528(13)	11.3563(8)
c [Å]	11.6567(5)	17.9006(14)	15.5737(11)
α [°]	90	90	99.447(6)
β [°]	112.079(3)	94.149(7)	95.411(5)
γ [°]	90	90	113.786(5)
V [Å^3]	2879.5(3)	2730.9(4)	1551.5(2)
Z	4	4	2
Calculated density [Mg m^{-3}]	1.171	1.150	1.138
T [K]	120	120	120
μ [mm^{-1}]	0.24	0.32	0.23
Crystal size/mm^3	0.26 × 0.19 × 0.03	0.41 × 0.23 × 0.19	0.28 × 0.11 × 0.04
λ [Å]	0.71073 (MoKα)	0.71073 (MoKα)	0.71073 (MoKα)
F(000)	1104	1024	576
S	1.04	1.00	1.04
R_{int}	0.031	0.042	0.054
No. of measured, independent, observed [I > 2σ(I)] reflections	19017, 7701, 5915	16038, 7319, 4852	23634, 8365, 6110
$R[F^2 > 2\sigma(F^2)]$	0.064	0.042	0.044
$wR(F^2)$	0.182	0.104	0.118
Largest diff. peak/hole / e Å^{-3}	1.18/-0.66	0.33/-0.30	0.39/-0.33

Single crystal X-ray structure analysis of 1c

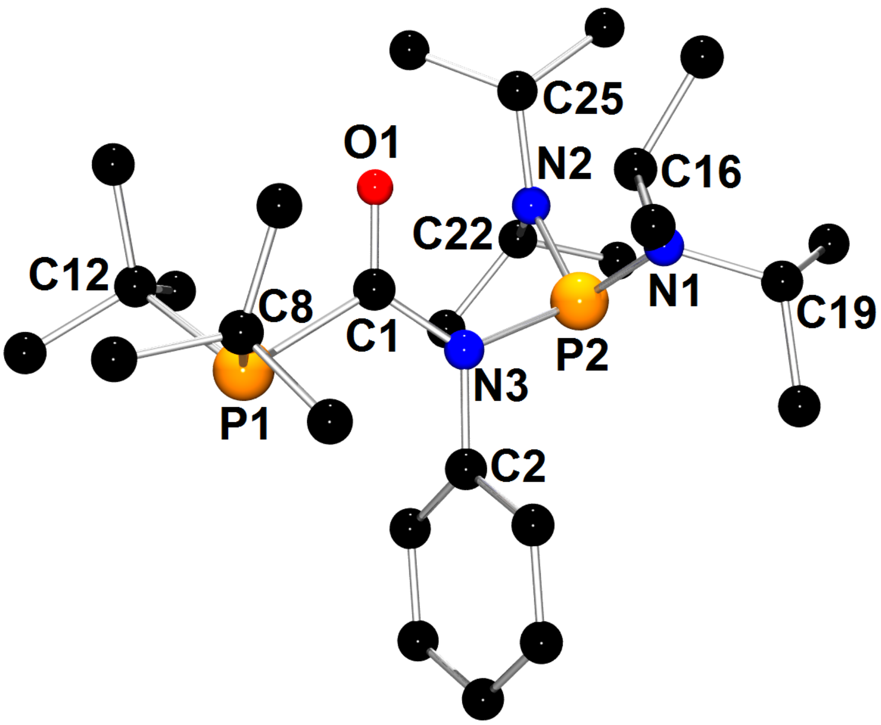

FIG. S2. MOLECULAR STRUCTURE OF **1C**

TABLE S7. SELECTED STRUCTURAL PARAMETERS OF **1C**

**Bond lengths [Å]**		**Bond angles [°]**		**Dihedrals [°]**	
P1-C8	1.895(2)	P1-C1-O1	121.2(1)	P1-C1-N3-P2	166.73(8)
P1-C12	1.895(2)	N3-C1-O1	121.0(1)	O1-C1-N3-C2	178.2(1)
P2-N1	1.680(1)	P1-C1-N3	117.7(1)		
P2-N2	1.683(2)	P2-N3-C2	110.2(1)		
P1-C1	1.909(2)	P2-N3-C1	127.6(1)		
C1-N3	1.374(2)	C1-N3-C2	121.6(1)		
C1-O1	1.228(2)	P2-N1-C19	115.4(1)		
P2-N3	1.818(1)	P2-N1-C16	128.1(1)		
N3-C2	1.446(2)	C16-N1-C19	116.4(1)		
N1-C16	1.481(2)	P2-N2-C22	114.5(1)		
N1-C19	1.493(2)	P2-N2-C25	128.4(1)		
N2-C22	1.493(2)	C22-N2-C25	116.4(1)		
N2-C25	1.480(2)				

Single crystal X-ray structure analysis of 1d and 1d’

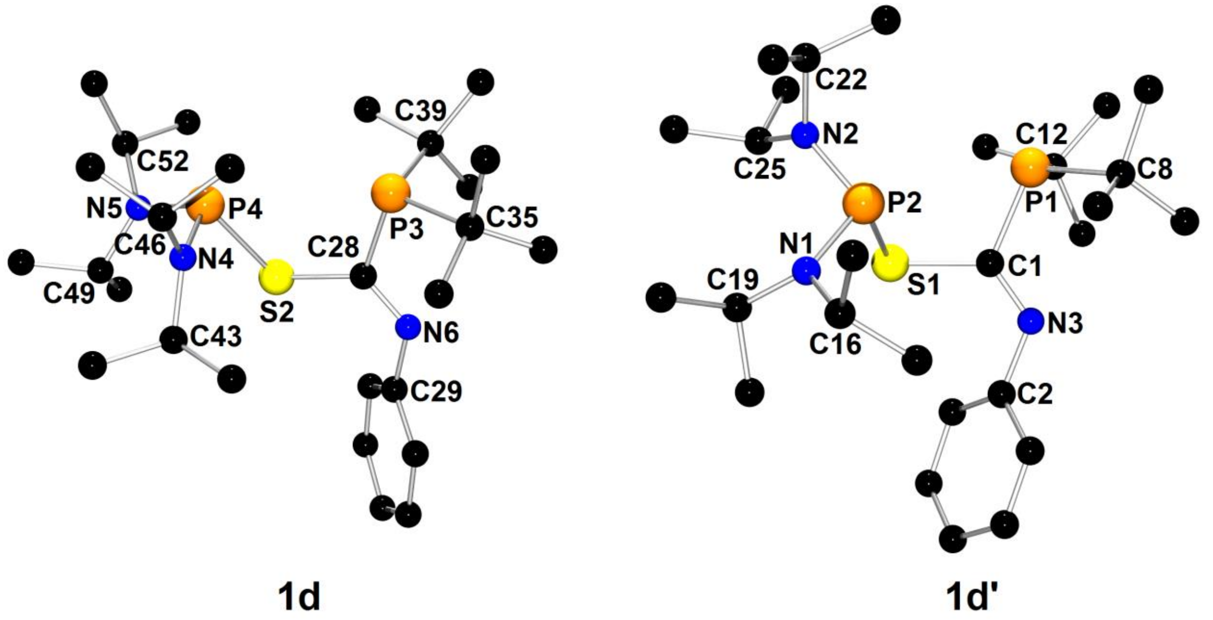

FIG. S3. MOLECULAR STRUCTURE OF **1D** AND **1D’**

TABLE S8. SELECTED STRUCTURAL PARAMETERS OF **1D**

**Bond lengths [Å]**		**Bond angles [°]**		**Dihedrals [°]**	
P3-C35	1.901(5)	P3-C28-S2	117.7(3)	P3-C28-S2-P4	-25.8(3)
P3-C39	1.892(5)	P3-C28-N6	119.4(4)	N6-C28-S2-P4	156.1(4)
P4-N4	1.676(5)	S2-C28-N6	122.9(4)		
P4-N5	1.689(5)	P4-S2-C28	107.7(2)		
P3-C28	1.855(5)	P4-N4-C43	128.5(3)		
C28-S2	1.798(5)	P4-N4-C46	116.2(4)		
C28-N6	1.280(6)	C43-N4-C46	115.0(4)		
P4-S2	2.201(2)	P4-N5-C49	126.5(4)		
N6-C29	1.425(7)	P4-N5-C52	117.3(4)		
N4-C43	1.480(7)	C49-N5-C52	115.6(4)		
N4-C46	1.485(8)				
N5-C49	1.470(7)				
N5-C52	1.494(7)				

TABLE S9. SELECTED STRUCTURAL PARAMETERS OF **1D’**

**Bond lengths [Å]**		**Bond angles [°]**		**Dihedrals [°]**	
P1-C8	1.888(5)	P1-C1-S1	112.9(2)	P1-C1-S1-P2	-62.6(2)
P1-C12	1.896(5)	S1-C1-N3	125.0(4)	N3-C1-S1-P2	120.9(4)
P2-N1	1.689(4)	N3-C1-P1	122.0(3)		
P2-N2	1.682(4)	P2-S1-C1	97.6(2)		
P1-C1	1.866(5)	P2-N1-C16	117.3(3)		
C1-S1	1.800(5)	P2-N1-C19	124.4(3)		
C1-N3	1.276(6)	C16-N1-C19	116.3(4)		
P2-S1	2.184(2)	P2-N2-C22	115.4(3)		
N3-C2	1.422(6)	P2-N2-C25	128.1(3)		
N1-C16	1.487(7)	C22-N2-C25	116.5(4)		
N1-C19	1.481(6)				
N2-C22	1.485(6)				
N2-C25	1.471(7)				

Single crystal X-ray structure analysis of $2a_{Pt}$

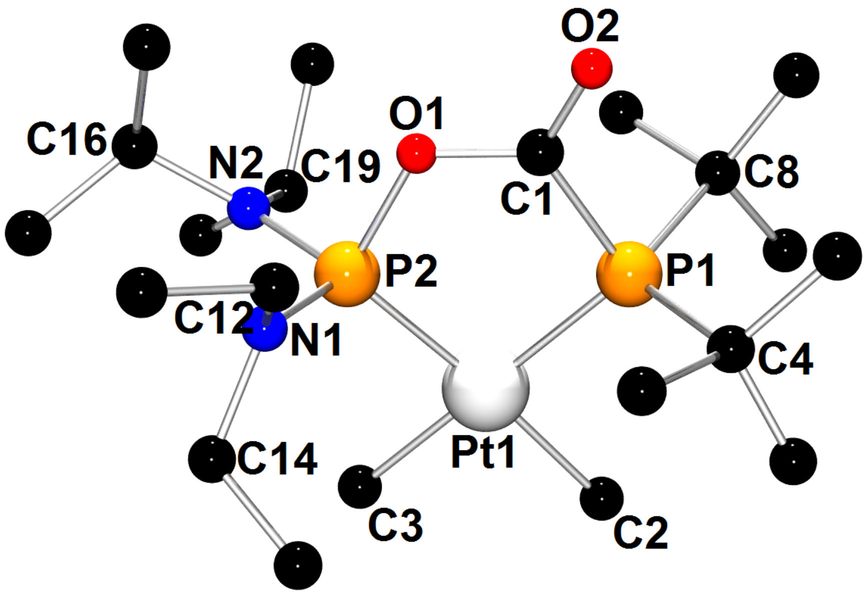

FIG. S4. MOLECULAR STRUCTURE OF **$2A_{Pt}$**

TABLE S10. SELECTED STRUCTURAL PARAMETERS OF **$2A_{Pt}$**

**Bond lengths [Å]**		**Bond angles [°]**		**Dihedrals [°]**	
P1-C1	1.90(2)	C1-P1-Pt1	104.9(5)	P1-C1-O1-P2	-172(1)
P1-C4	1.87(2)	O1-P2-Pt1	110.0(4)	O2-C1-O1-P2	120.9(4)
P1-C8	1.90(1)	P1-Pt1-P2	85.9(1)		
P1-Pt1	2.277(3)	P1-C1-O1	117(1)		
P2-N1	1.67(1)	P2-O1-C1	120.8(9)		
P2-N2	1.67(1)	P1-C1-O2	123(1)		
P2-O1	1.69(1)	O1-C1-O2	120(1)		
P2-Pt1	2.257(3)				
O1-C1	1.33(2)				
O2-C1	1.21(2)				
N1-C12	1.46(2)				
N1-C14	1.51(2)				
N2-C16	1.49(2)				
N2-C19	1.46(2)				
Pt1-C2	2.13(1)				
Pt1-C3	2.10(2)				

Single crystal X-ray structure analysis of 2d

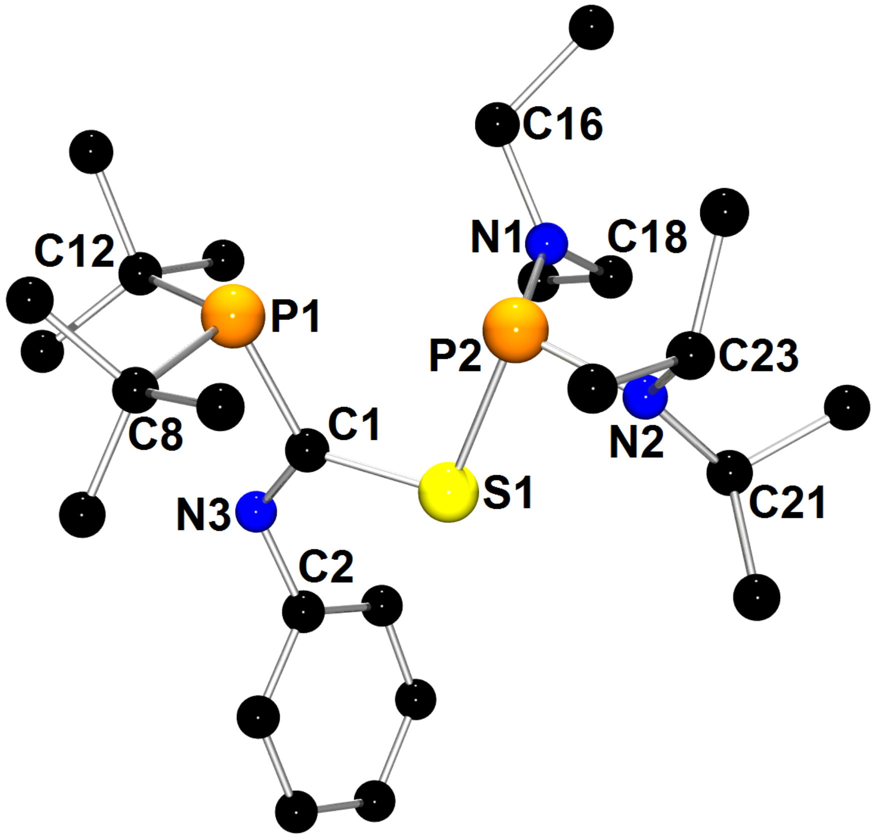

FIG. S5. MOLECULAR STRUCTURE OF **2D**

TABLE S11. SELECTED STRUCTURAL PARAMETERS OF **2D**

Bond lengths [Å]		Bond angles [°]		Dihedrals [°]	
P1-C8	1.901(2)	P1-C1-S1	118.53(9)	P1-C1-S1-P2	-16.7(1)
P1-C12	1.894(2)	P1-C1-N3	120.1(1)	S1-C1-N3-C2	-1.6(2)
P1-C1	1.861(2)	S1-C1-N3	121.3(1)		
P2-N1	1.675(2)	P2-S1-C1	103.96(6)		
P2-N2	1.683(1)	P2-N1-C16	116.0(1)		
P2-S1	2.1900(7)	P2-N1-C18	127.5(1)		
S1-C1	1.790(2)	C16-N1-C18	116.5(1)		
N3-C1	1.276(2)	P2-N2-C21	127.7(1)		
N3-C2	1.421(2)	P2-N2-C23	114.9(1)		
N1-C16	1.466(2)	C21-N2-C23	116.6(1)		
N1-C18	1.467(3)				
N2-C21	1.477(2)				
N2-C23	1.487(2)				

Single crystal X-ray structure analysis of 3c

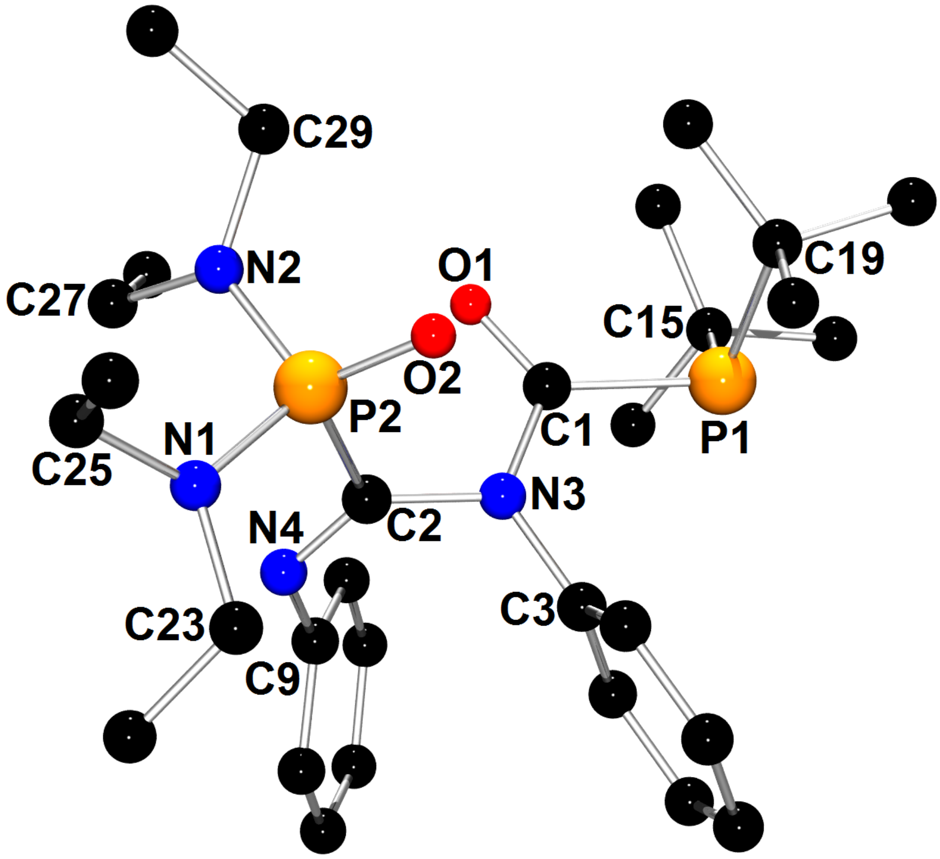

FIG. S6. MOLECULAR STRUCTURE OF **3C**

TABLE S12. SELECTED STRUCTURAL PARAMETERS OF **3D**

**Bond lengths [Å]**		**Bond angles [°]**		**Dihedrals [°]**	
P1-C15	1.887(2)	P1-C1-N3	116.1(1)	P1-C1-N3-C3	0.8(2)
P1-C19	1.887(2)	P1-C1-O1	125.3(1)	O1-C1-N3-C2	-9.8(2)
P1-C1	1.891(1)	N3-C1-O1	118.6(2)	P2-C2-N3-C1	-66.8(2)
P2-N1	1.650(2)	C1-N3-C2	117.8(1)	O2-P2-C2-N4	163.7(1)
P2-N2	1.638(2)	P2-C2-N3	117.8(1)		
P2-C2	1.854(2)	P2-C2-N4	118.7(1)		
P2-O2	1.475(1)	C1-N3-C3	125.4(1)		
O1-C1	1.224(2)	C2-N3-C3	116.3(1)		
N3-C1	1.375(3)	N3-C2-N4	123.5(2)		
N3-C3	1.441(2)	P2-N1-C23	119.2(1)		
N3-C2	1.451(2)	P2-N1-C25	120.1(1)		
N4-C2	1.273(2)	C23-N1-C25	114.9(2)		
N4-C9	1.416(2)	P2-N2-C27	121.1(1)		
N1-C23	1.463(3)	P2-N2-C29	120.4(1)		
N1-C25	1.475(2)	C27-N2-C29	117.1(2)		
N2-C27	1.467(2)	O2-P2-C2	108.84(8)		
N2-C29	1.469(3)				

Single crystal X-ray structure analysis of 3d

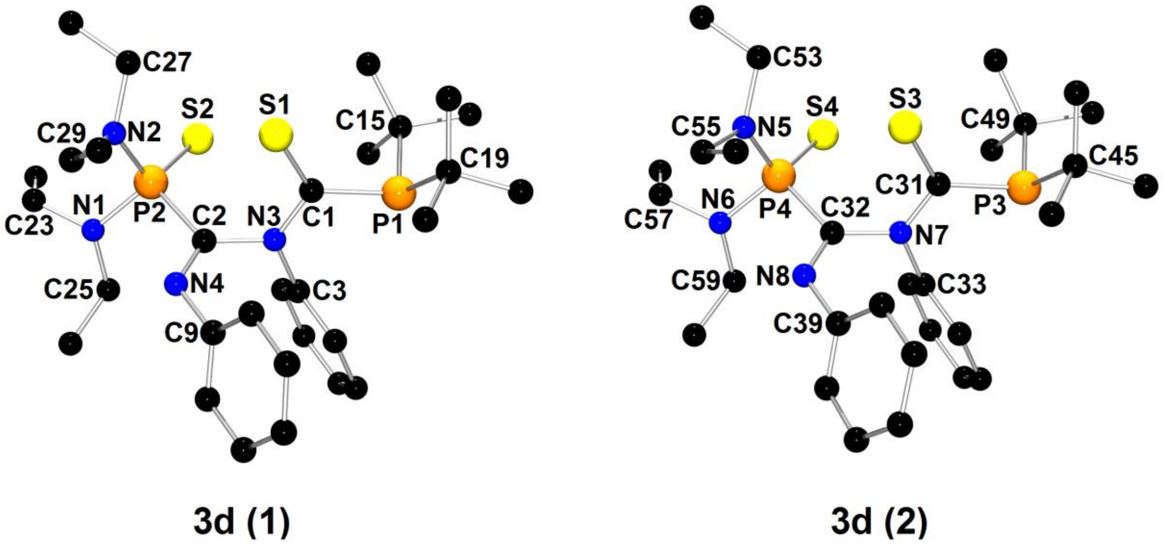

FIG. S7. MOLECULAR STRUCTURE OF **3D(1)** AND **3D(2)**

TABLE S13. SELECTED STRUCTURAL PARAMETERS OF **3D(1)**

**Bond lengths [Å]**		**Bond angles [°]**		**Dihedrals [°]**	
P1-C15	1.884(7)	P1-C1-S1	126.6(4)	P1-C1-N3-C3	1.1(8)
P1-C19	1.894(7)	P1-C1-N3	115.4(5)	S1-C1-N3-C2	5.1(8)
P1-C1	1.879(6)	S1-C1-N3	118.0(5)	N3-C2-P2-S2	9.3(5)
P2-N1	1.663(6)	P2-C2-N3	120.0(4)		
P2-N2	1.654(5)	P2-C2-N4	116.5(5)		
P2-S2	1.935(3)	N3-C2-N4	123.3(6)		
P2-C2	1.875(6)	S2-P2-C2	111.0(2)		
S1-C1	1.657(6)	P2-N2-C27	122.4(5)		
N3-C1	1.352(9)	P2-N2-C29	120.6(4)		
N3-C3	1.461(8)	C27-N2-C29	116.3(5)		
N3-C2	1.472(7)	P2-N1-C23	117.1(4)		
N4-C2	1.251(9)	P2-N1-C25	120.5(4)		
N4-C9	1.438(8)	C23-N1-C25	114.9(5)		
N1-C23	1.485(7)	C1-N3-C2	119.3(5)		
N1-C25	1.481(8)	C1-N3-C3	125.0(5)		
N2-C27	1.47(1)	C2-N3-C3	115.7(5)		
N2-C29	1.474(9)				

TABLE S14. SELECTED STRUCTURAL PARAMETERS OF **3D(2)**

Bond lengths [Å]		Bond angles [°]		Dihedrals [°]	
P3-C45	1.901(7)	P3-C31-S3	126.4(4)	P3-C31-N7-C33	2.2(8)
P3-C49	1.879(8)	P3-C31-N7	116.0(5)	S3-C31-N7-C32	4.9(8)
P3-C31	1.888(6)	S3-C31-N7	117.6(5)	S4-P4-C32-N7	9.6(6)
P4-N5	1.643(5)	P4-C32-N8	115.9(5)		
P4-N6	1.665(5)	P4-C32-N7	120.7(4)		
P4-S4	1.938(3)	N7-C32-N8	123.2(6)		
P4-C32	1.865(6)	S4-P4-C32	111.0(2)		
S3-C31	1.662(7)	P4-N5-C53	121.8(5)		
N7-C31	1.351(8)	P4-N5-C55	121.5(5)		
N7-C33	1.461(8)	C53-N5-C55	115.9(5)		
N7-C32	1.455(7)	P4-N6-C57	118.5(5)		
N8-C39	1.425(9)	P4-N6-C59	119.7(4)		
N8-C32	1.277(9)	C57-N6-C59	114.5(5)		
N5-C53	1.473(9)	C31-N7-C32	120.0(5)		
N5-C55	1.477(9)	C31-N7-C33	123.4(5)		
N6-C57	1.474(9)	C32-N7-C33	116.7(5)		
N6-C59	1.468(8)				

Single crystal X-ray structure analysis of 4

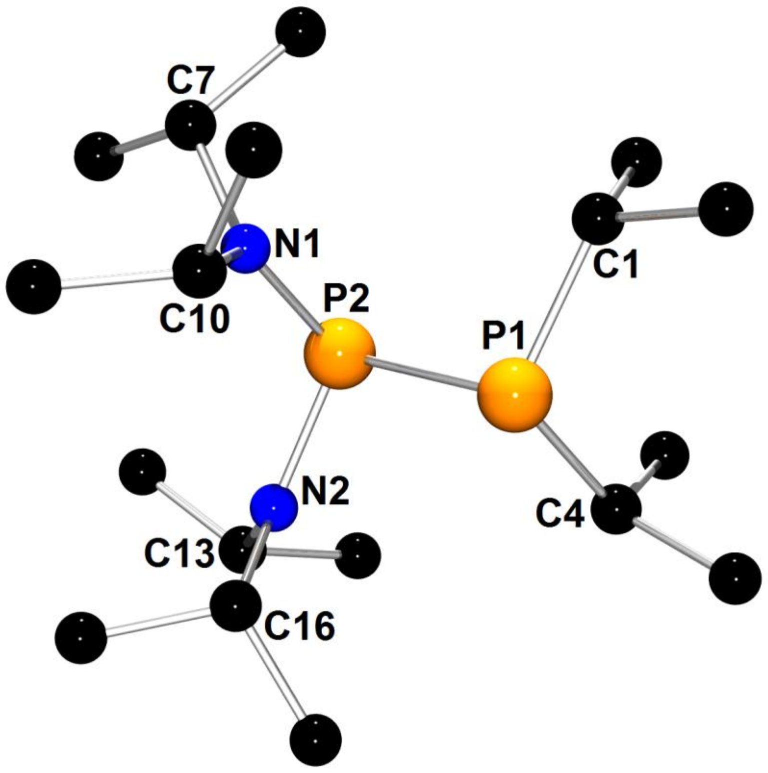

FIG. S8. MOLECULAR STRUCTURE OF **4**

TABLE S15. SELECTED STRUCTURAL PARAMETERS OF **4**

Bond lengths [Å]		Bond angles [°]		Dihedrals [°]	
P1-P2	2.2278(4)	P2-N1-C7	118.71(9)	C1-P1-P2-N1	-69.39(6)
P1-C1	1.877(1)	P2-N1-C10	126.54(9)	C4-P1-P2-N2	64.42(6)
P1-C4	1.872(2)	C7-N1-C10	114.6(1)		
P2-N1	1.703(1)	P2-N2-C13	118.47(9)		
P2-N2	1.703(1)	P2-N2-C16	126.45(9)		
N1-C7	1.481(1)	C13-N2-C16	115.0(1)		
N1-C10	1.480(2)				
N2-C13	1.486(2)				
N2-C16	1.481(1)				

Single crystal X-ray structure analysis of 4b

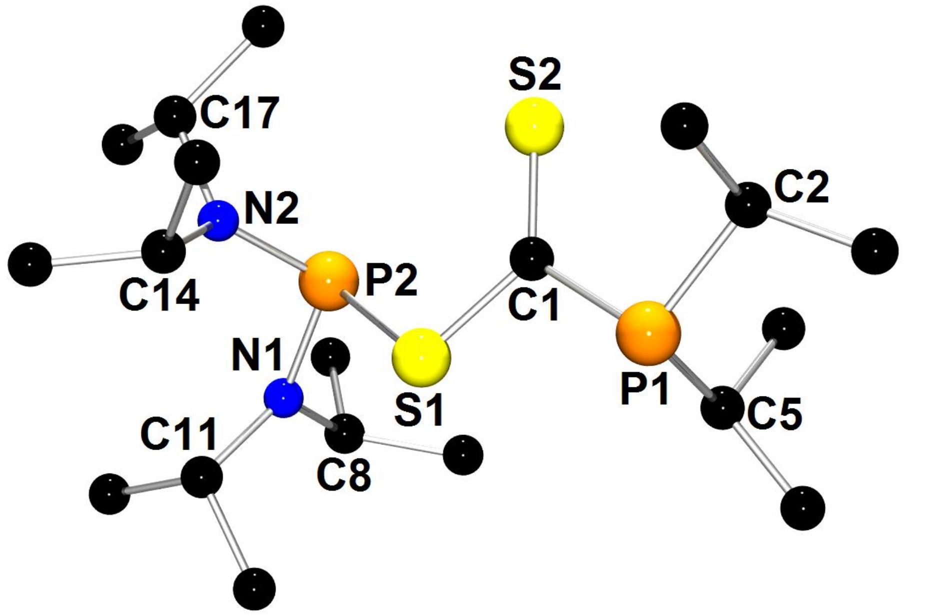

FIG. S9. MOLECULAR STRUCTURE OF **4B**

TABLE S16. SELECTED STRUCTURAL PARAMETERS OF **4B**

Bond lengths [Å]		Bond angles [°]		Dihedrals [°]	
P1-C1	1.859(2)	P1-C1-S2	126.9(1)	P1-C1-S1-P2	-164.83(8)
P1-C2	1.852(2)	P1-C1-S1	107.8(1)	S2-C1-S1-P2	15.4(2)
P1-C5	1.875(2)	S1-C1-S2	125.3(1)		
P2-N1	1.681(2)	P2-S1-C1	104.31(7)		
P2-N2	1.674(2)	P2-N1-C8	116.3(1)		
P2-S1	2.2088(7)	P2-N1-C11	125.9(1)		
S2-C1	1.634(2)	C8-N1-C11	116.8(2)		
S1-C1	1.731(2)	P2-N2-C14	127.5(1)		
N1-C8	1.486(3)	P2-N2-C17	116.4(1)		
N1-C11	1.474(3)	C14-N2-C17	115.6(2)		
N2-C14	1.481(3)				
N2-C17	1.496(3)				

Single crystal X-ray structure analysis of 4d

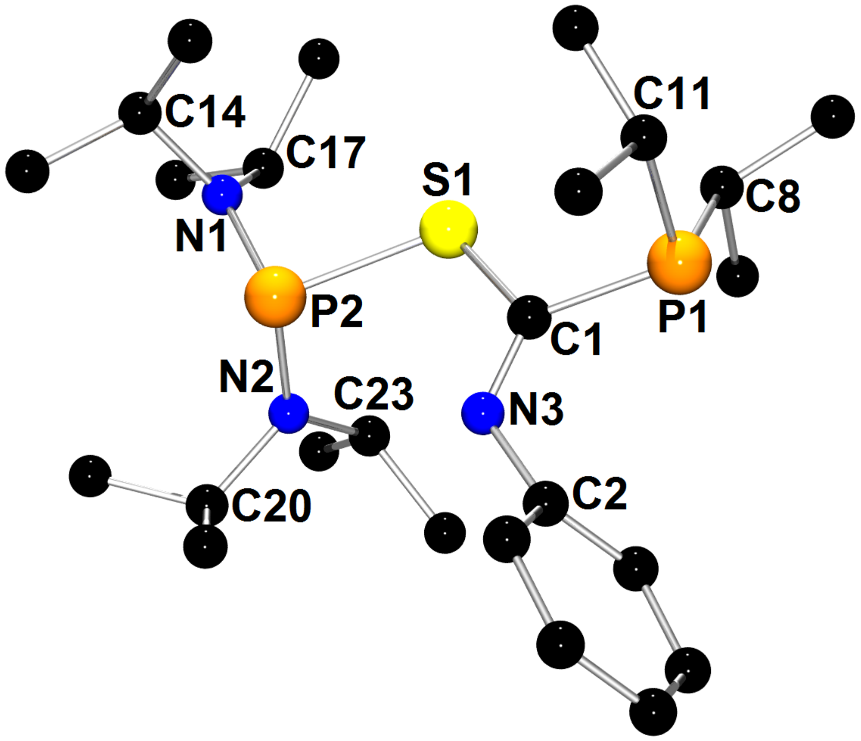

FIG. S10. MOLECULAR STRUCTURE OF **4D'**

TABLE S17. SELECTED STRUCTURAL PARAMETERS OF **4D'**

**Bond lengths [Å]**		**Bond angles [°]**		**Dihedrals [°]**	
P1-C1	1.866(4)	P1-C1-N3	124.3(3)	P1-C1-S1-P2	-165.5(2)
P1-C8	1.853(3)	P1-C1-S1	117.3(2)	P2-S1-C1-N3	12.6(3)
P1-C11	1.864(5)	N3-C1-S1	118.4(3)		
P2-N1	1.685(3)	P2-S1-C1	105.2(1)		
P2-N2	1.685(3)	P2-N1-C14	117.0(2)		
N3-C1	1.271(5)	P2-N1-C17	125.7(2)		
N3-C2	1.424(5)	C14-N1-C17	116.2(3)		
S1-C1	1.777(4)	P2-N2-C20	114.7(2)		
P2-S1	2.175(1)	P2-N2-C23	128.9(3)		
N1-C14	1.485(5)	C20-N2-C23	116.3(3)		
N1-C17	1.473(5)				
N2-C20	1.490(4)				
N2-C23	1.458(5)				

Single crystal X-ray structure analysis of 6c

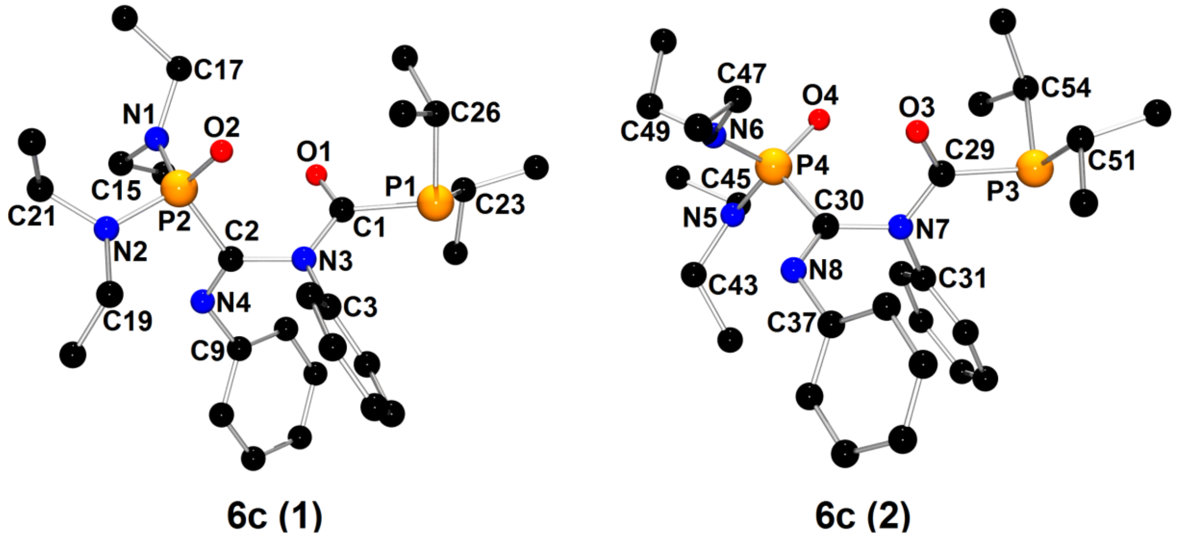

FIG. S11. MOLECULAR STRUCTURE OF **6C(1)** AND **6C(2)**

TABLE S18. SELECTED STRUCTURAL PARAMETERS OF **6C(2)**

**Bond lengths [Å]**		**Bond angles [°]**		**Dihedrals [°]**	
P1-C23	1.853(1)	P1-C1-O1	123.00(8)	P1-C1-N3-C3	2.4(1)
P1-C26	1.865(1)	P1-C1-N3	117.18(8)	O1-C1-N3-C2	-9.2(1)
P1-C1	1.891(1)	O1-C1-N3	119.8(1)	C1-N3-C2-N4	108.2(1)
P2-N1	1.637(1)	C1-N3-C2	117.52(9)	P2-C2-N3-C1	-71.7(1)
P2-N2	1.6479(9)	C1-N3-C3	125.66(9)	O2-P2-C2-N4	168.27(9)
P2-O2	1.4776(9)	C2-N3-C3	116.18(9)		
P2-C2	1.851(1)	P2-C2-N3	116.03(7)		
O1-C1	1.224(1)	P2-C2-N4	119.72(8)		
N3-C1	1.370(1)	N3-C2-N4	124.3(1)		
N3-C2	1.460(1)	P2-N1-C15	122.86(8)		
N3-C3	1.443(2)	P2-N1-C17	120.19(8)		
N4-C2	1.266(1)	C15-N1-C17	116.74(9)		
N4-C9	1.424(1)	P2-N2-C19	119.21(8)		
N1-C15	1.469(2)	P2-N2-C21	119.80(8)		
N1-C17	1.468(2)	C19-N2-C21	114.84(9)		
N2-C19	1.475(2)	O2-P2-C2	108.65(5)		
N2-C21	1.471(1)				

TABLE S19. SELECTED STRUCTURAL PARAMETERS OF **6C(2)**

**Bond lengths [Å]**		**Bond angles [°]**		**Dihedrals [°]**	
P3-C51	1.858(1)	P3-C29-O3	122.64(8)	P3-C29-N7-C31	1.7(1)
P3-C54	1.869(1)	P3-C29-N7	117.79(8)	O3-C29-N7-C30	9.7(1)
P3-C29	1.891(1)	O3-C29-N7	119.5(1)	C29-N7-C30-N8	-109.3(1)
P4-N5	1.644(1)	C29-N7-C30	117.54(9)	P4-C30-N7-C29	79.3(1)
P4-N6	1.647(1)	C29-N7-C31	125.36(9)	O4-P4-C30-N8	174.21(9)
P4-O4	1.4750(9)	C30-N7-C31	116.86(9)		
P4-C30	1.843(1)	P4-C30-N7	116.25(8)		

O3-C29	1.226(1)	P4-C30-N8	118.43(8)
N7-C29	1.371(1)	N7-C30-N8	124.8(1)
N7-C30	1.457(1)	P4-N5-C43	121.95(8)
N7-C31	1.442(2)	P4-N5-C45	121.87(8)
N8-C30	1.268(1)	C43-N5-C45	115.9(1)
N8-C37	1.420(1)	P4-N6-C47	117.46(8)
N5-C43	1.468(2)	P4-N6-C49	118.45(8)
N5-C45	1.469(2)	C47-N6-C49	115.1(1)
N6-C47	1.477(2)	O4-P4-C30	109.69(5)
N6-C49	1.472(2)		

Single crystal X-ray structure analysis of 6d

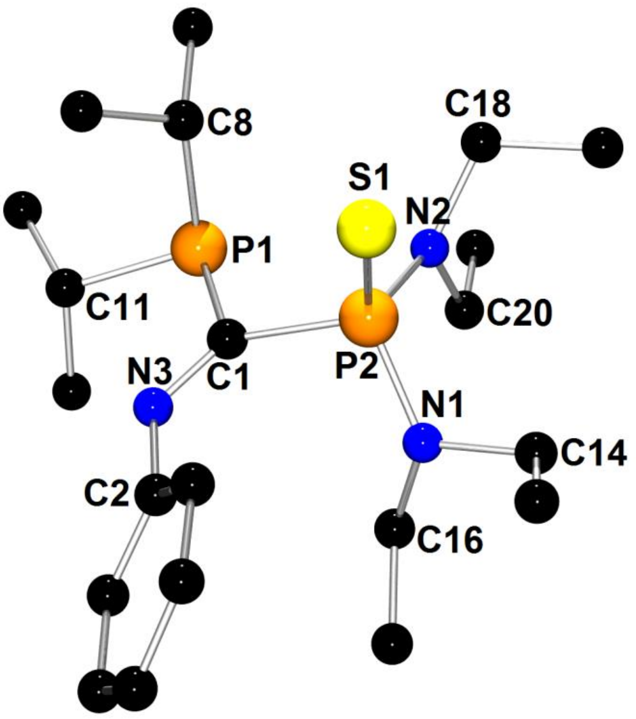

FIG. S12. MOLECULAR STRUCTURE OF **6D**

TABLE S20. SELECTED STRUCTURAL PARAMETERS OF **6D**

Bond lengths [Å]		Bond angles [°]		Dihedrals [°]	
P1-C1	1.864(2)	P1-C1-P2	112.6(1)	P1-C1-P2-N2	-17.1(1)
P1-C8	1.879(3)	P1-C1-N3	118.4(2)	P1-C1-P2-S1	101.7(1)
P1-C11	1.861(3)	P2-C1-N3	129.0(2)	N3-C1-P2-N2	163.8(2)
P2-N1	1.653(2)	P2-N1-C14	116.3(1)	P2-C1-P1-C11	-179.2(1)
P2-N2	1.668(1)	P2-N1-C16	123.8(1)		
P2-S1	1.9573(9)	C14-N1-C16	115.1(2)		
P2-C1	1.875(2)	P2-N2-C18	123.2(1)		
N3-C1	1.279(2)	P2-N2-C20	116.3(1)		
N3-C2	1.422(3)	C18-N2-C20	116.8(2)		
N1-C14	1.484(3)	S1-P2-C1	110.60(7)		
N1-C16	1.480(4)				
N2-C18	1.478(3)				
N2-C20	1.478(3)				

Single crystal X-ray structure analysis of 7

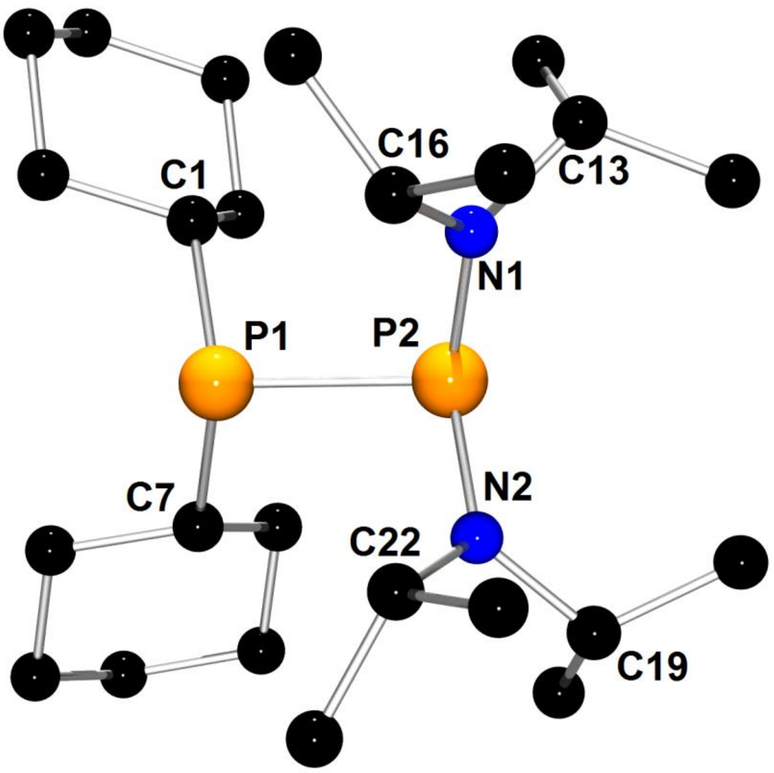

FIG. S13. MOLECULAR STRUCTURE OF **7**

TABLE S21. SELECTED STRUCTURAL PARAMETERS OF **7**

**Bond lengths [Å]**		**Bond angles [°]**		**Dihedrals [°]**	
P1-P2	2.2317(9)	P2-N1-C13	119.0(2)	C1-P1-P2-N1	65.3(1)
P1-C1	1.871(2)	P2-N1-C16	126.7(2)	C7-P1-P2-N2	-68.3(1)
P1-C7	1.871(3)	C13-N1-C16	114.2(2)		
P2-N1	1.703(2)	P2-N2-C19	120.6(2)		
P2-N2	1.697(2)	P2-N2-C22	125.0(2)		
N1-C13	1.491(4)	C19-N2-C22	114.1(2)		
N1-C16	1.484(3)				
N2-C19	1.475(4)				
N2-C22	1.491(3)				

Single crystal X-ray structure analysis of 7b

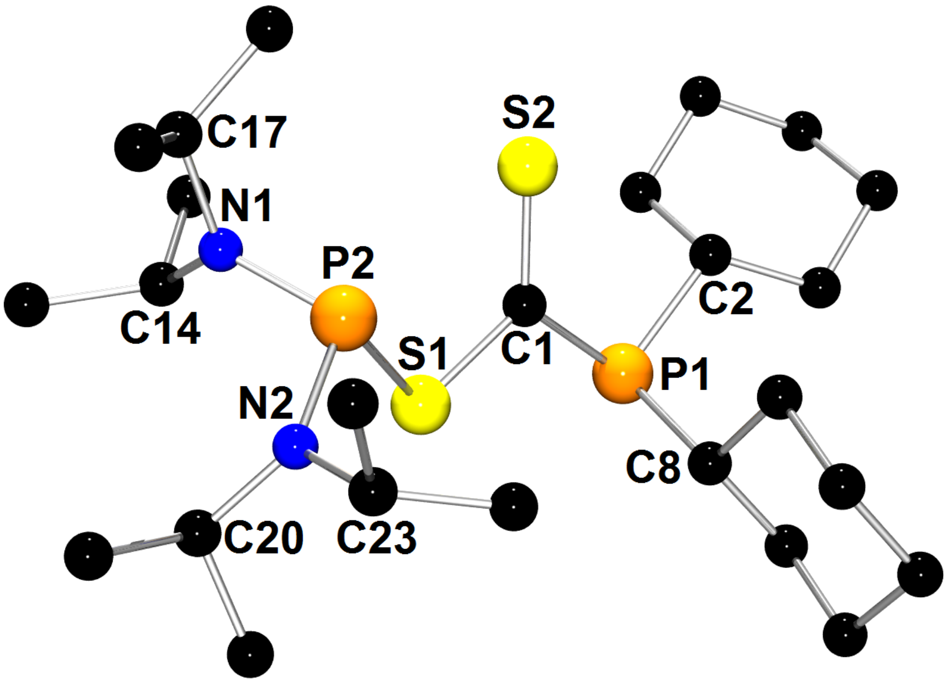

FIG. S14. MOLECULAR STRUCTURE OF **7B**

TABLE S22. SELECTED STRUCTURAL PARAMETERS OF **7B**

**Bond lengths [Å]**		**Bond angles [°]**		**Dihedrals [°]**	
P1-C1	1.849(6)	P1-C1-S2	126.0(3)	P1-C1-S1-P2	162.0(2)
P1-C2	1.852(5)	P1-C1-S1	110.5(3)	P2-S1-C1-S2	-18.5(4)
P1-C8	1.871(5)	S1-C1-S2	123.5(3)		
P2-S1	2.203(2)	P2-S1-C1	102.5(2)		
P2-N1	1.680(5)	P2-N1-C17	116.0(3)		
P2-N2	1.667(4)	P2-N1-C14	127.1(4)		
S1-C1	1.734(5)	C14-N1-C17	116.6(4)		
S2-C1	1.647(6)	P2-N2-C20	126.7(4)		
N1-C14	1.502(6)	P2-N2-C23	116.7(4)		
N1-C17	1.462(8)	C20-N2-C23	116.0(4)		
N2-C20	1.477(7)				
N2-C23	1.485(7)				

Single crystal X-ray structure analysis of 7d

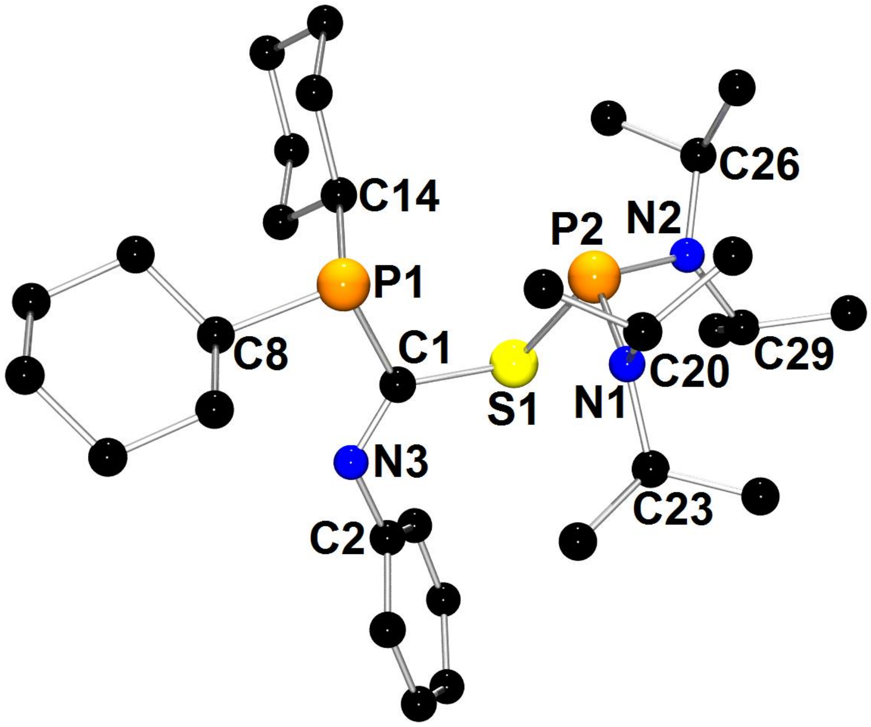

FIG. S15. MOLECULAR STRUCTURE OF **7D**

TABLE S23. SELECTED STRUCTURAL PARAMETERS OF **7D**

**Bond lengths [Å]**		**Bond angles [°]**		**Dihedrals [°]**	
P1-C1	1.850(2)	P1-C1-S1	116.7(1)	P1-C1-S1-P2	35.4(1)
P1-C8	1.861(2)	P1-C1-N3	120.6(1)	P2-S1-C1-N3	-145.4(2)
P1-C14	1.870(2)	S1-C1-N3	122.7(1)		
P2-N1	1.681(2)	P2-S1-C1	105.02(7)		
P2-N2	1.693(2)	P2-N1-C20	117.2(1)		
P2-S1	2.1876(7)	P2-N1-C23	127.3(1)		
S1-C1	1.797(2)	C20-N1-C23	115.3(2)		
N3-C1	1.279(3)	P2-N2-C26	116.3(1)		
N3-C2	1.414(3)	P2-N2-C29	126.1(1)		
N1-C20	1.489(3)	C26-N2-C29	116.2(1)		
N1-C23	1.473(3)				
N2-C26	1.488(3)				
N2-C29	1.479(2)				

Single crystal X-ray structure analysis of 9c

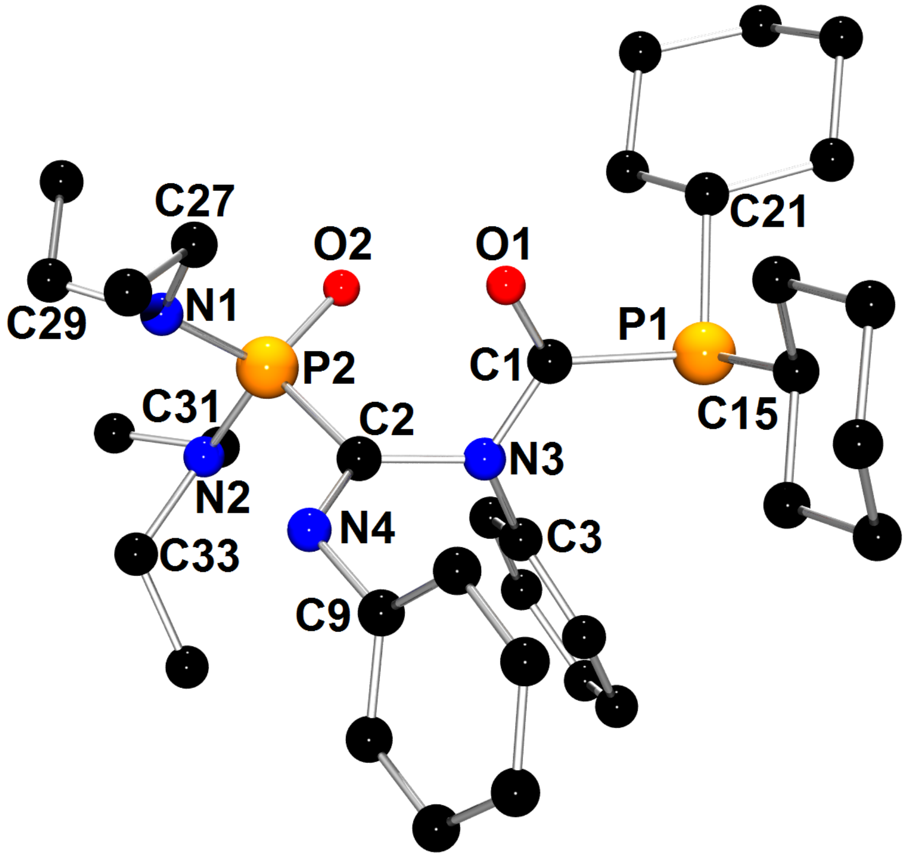

FIG. S16. MOLECULAR STRUCTURE OF **9C**

TABLE S24. SELECTED STRUCTURAL PARAMETERS OF **9C**

**Bond lengths [Å]**		**Bond angles [°]**		**Dihedrals [°]**	
P1-C1	1.894(3)	P1-C1-O1	123.6(2)	P1-C1-N3-C2	-171.8(2)
P1-C15	1.863(3)	P1-C1-N3	116.4(2)	O1-C1-N3-C3	171.8(2)
P1-C21	1.859(3)	O1-C1-N3	119.9(2)	C1-N3-C2-N4	-110.5(3)
P2-N1	1.642(2)	P2-C2-N3	116.5(2)	P2-C2-N3-C1	77.6(2)
P2-N2	1.642(2)	P2-C2-N4	119.8(2)	O2-P2-C2-N4	171.2(2)
P2-O2	1.478(2)	N3-C2-N4	123.2(2)		
P2-C2	1.853(3)	C1-N3-C2	116.4(2)		
O1-C1	1.224(3)	C1-N3-C3	124.5(2)		
N3-C1	1.381(3)	C2-N3-C3	116.7(2)		
N3-C3	1.453(3)	P2-N1-C27	118.3(2)		
N3-C2	1.453(3)	P2-N1-C29	119.6(2)		
N4-C2	1.280(3)	C27-N1-C29	115.5(2)		
N4-C9	1.431(4)	P2-N2-C31	121.6(2)		
N1-C27	1.474(4)	P2-N2-C33	120.6(2)		
N1-C29	1.477(4)	C31-N2-C33	117.7(2)		
N2-C31	1.470(4)	O2-P2-C2	109.3(1)		
N2-C33	1.474(3)				

Single crystal X-ray structure analysis of 9d

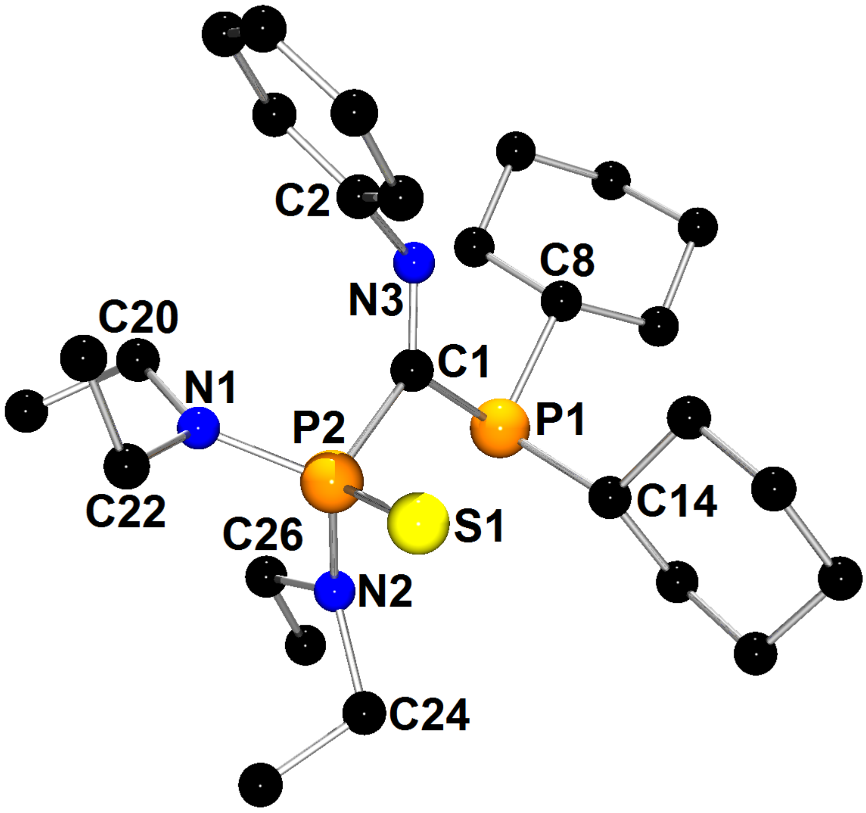

FIG. S17. MOLECULAR STRUCTURE OF **9D**

TABLE S25. SELECTED STRUCTURAL PARAMETERS OF **9D**

**Bond lengths [Å]**		**Bond angles [°]**		**Dihedrals [°]**	
P1-C1	1.868(3)	P1-C1-P2	114.8(1)	P1-C1-P2-N2	-20.5(1)
P1-C8	1.854(2)	P1-C1-N3	118.9(2)	P1-C1-P2-S1	96.8(1)
P1-C14	1.871(2)	P2-C1-N3	126.1(2)	N3-C1-P2-N2	164.2(2)
P2-N1	1.649(3)	P2-N1-C20	124.0(2)	P2-C1-P1-C8	-176.9(1)
P2-N2	1.679(2)	P2-N1-C22	118.7(2)		
P2-S1	1.9521(8)	C20-N1-C22	116.6(2)		
P2-C1	1.866(2)	P2-N2-C24	122.1(2)		
N3-C1	1.272(3)	P2-N2-C26	116.8(2)		
N3-C2	1.416(3)	C24-N2-C26	116.8(2)		
N1-C20	1.473(3)	S1-P2-C1	110.50(8)		
N1-C22	1.462(4)				
N2-C24	1.505(4)				
N2-C26	1.491(3)				

Single crystal X-ray structure analysis of 10b

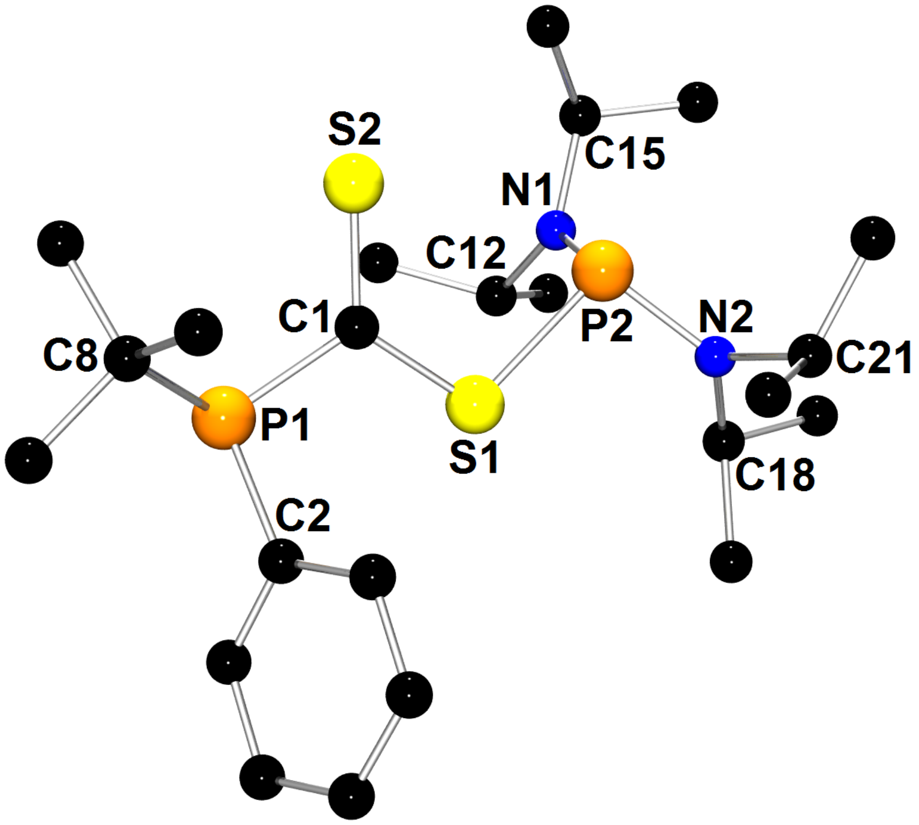

FIG. S18. MOLECULAR STRUCTURE OF **10B**

TABLE S26. SELECTED STRUCTURAL PARAMETERS OF **10B**

**Bond lengths [Å]**		**Bond angles [°]**		**Dihedrals [°]**	
P1-C1	1.831(2)	P1-C1-S1	113.18(9)	P1-C1-S1-P2	172.93(7)
P1-C2	1.834(2)	P1-C1-S2	122.0(1)	P2-S1-C1-S2	2.7(1)
P1-C8	1.896(2)	S1-C1-S2	124.1(1)		
P2-N1	1.676(1)	P2-S1-C1	103.27(6)		
P2-N2	1.684(1)	P2-N1-C12	127.7(1)		
P2-S1	2.1955(6)	P2-N1-C15	116.5(1)		
S1-C1	1.749(2)	C12-N1-C15	115.8(1)		
S2-C1	1.640(2)	P2-N2-C18	126.4(1)		
N1-C12	1.485(2)	P2-N2-C21	116.6(1)		
N1-C15	1.490(2)	C18-N2-C21	116.4(1)		
N2-C18	1.474(2)				
N2-C21	1.483(2)				

Single crystal X-ray structure analysis of 10d

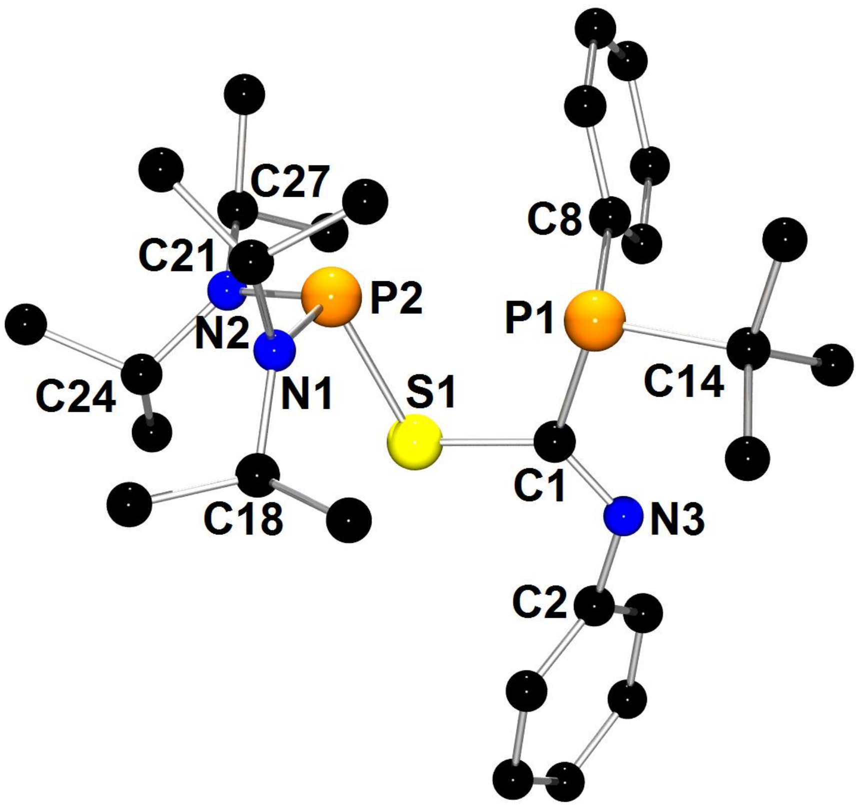

FIG. S19. MOLECULAR STRUCTURE OF **10D**

TABLE S27. SELECTED STRUCTURAL PARAMETERS OF **10D**

Bond lengths [Å]		Bond angles [°]		Dihedrals [°]	
P1-C1	1.852(2)	P1-C1-S1	115.14(9)	P1-C1-S1-P2	-2.9(1)
P1-C8	1.834(2)	P1-C1-N3	122.3(1)	P2-S1-C1-N3	177.4(1)
P1-C14	1.886(2)	S1-C1-N3	122.6(1)		
P2-N1	1.692(1)	P2-S1-C1	105.91(6)		
P2-N2	1.681(2)	P2-N1-C21	115.3(1)		
P2-S1	2.2065(7)	P2-N1-C18	127.9(1)		
S1-C1	1.786(2)	C18-N1-C21	116.2(1)		
N3-C1	1.274(2)	P2-N2-C24	124.6(1)		
N3-C2	1.418(2)	P2-N2-C27	117.2(1)		
N1-C18	1.477(2)	C24-N2-C27	116.9(1)		
N1-C21	1.488(2)				
N2-C24	1.474(2)				
N2-C27	1.485(2)				

Spectroscopic data

NMR spectra of isolated compounds

NMR spectra of 4

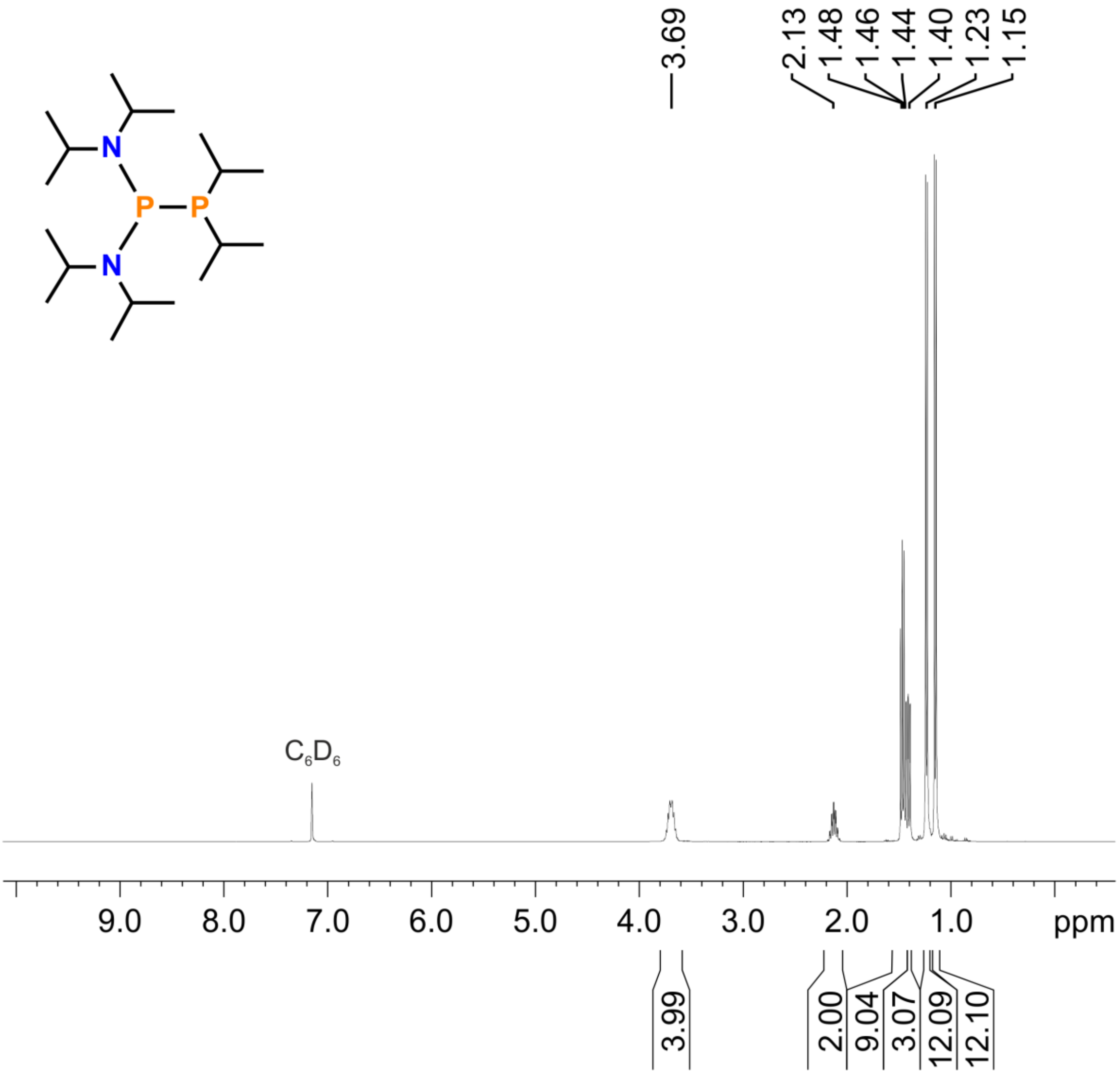

FIG. S20. ^{1}H NMR (C_6D_6) SPECTRUM OF **4**

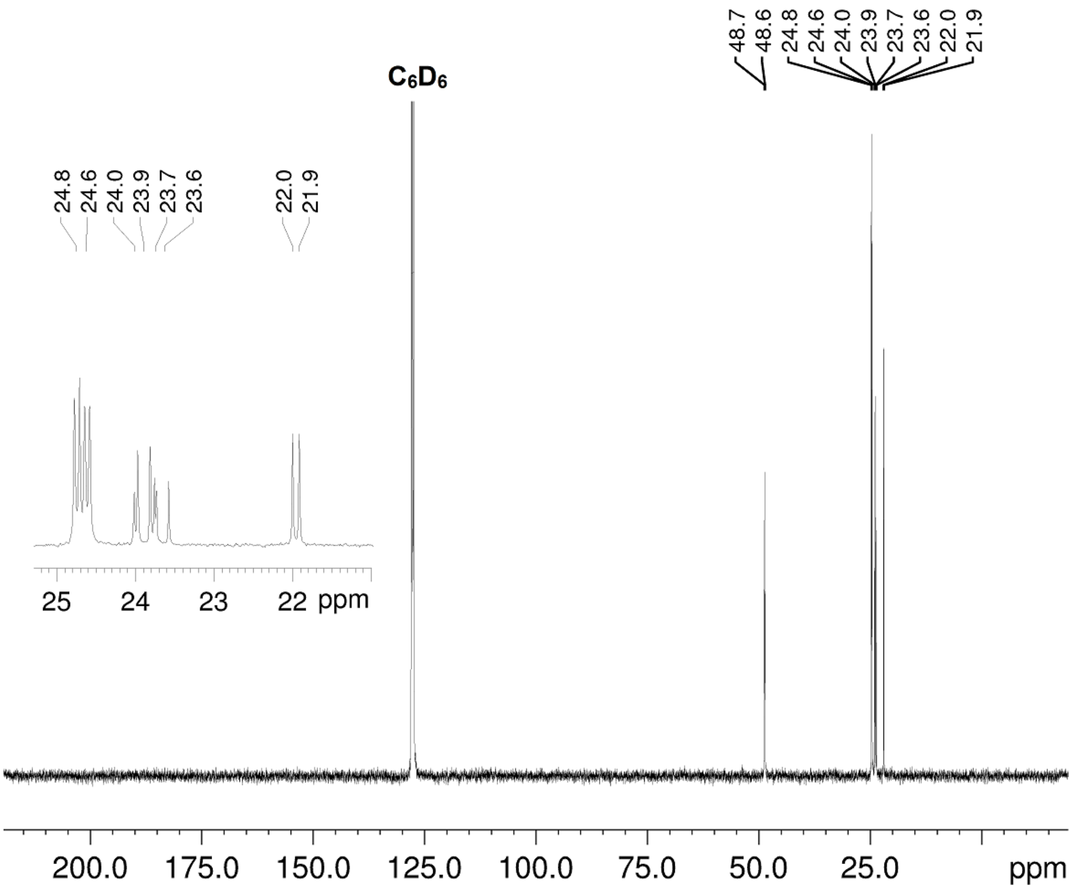

FIG. S21. $^{13}C\{^1H\}$ NMR (C_6D_6) SPECTRUM OF **4**

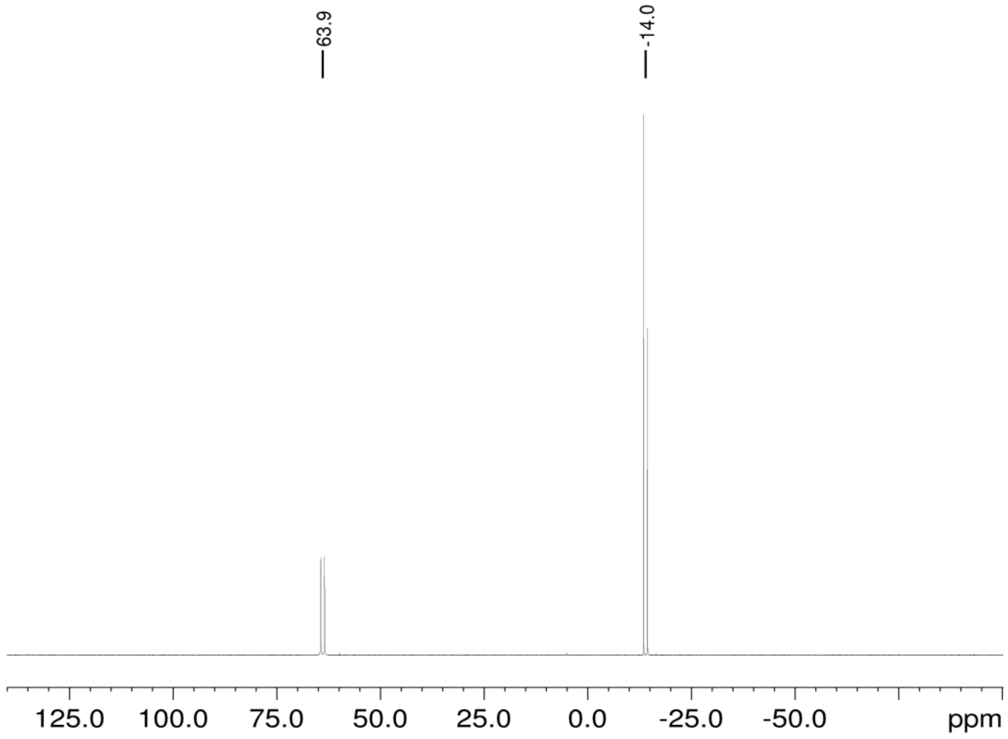

FIG. S22. $^{31}P\{^1H\}$ NMR (C_6D_6) SPECTRUM OF **4**

NMR spectra of 5

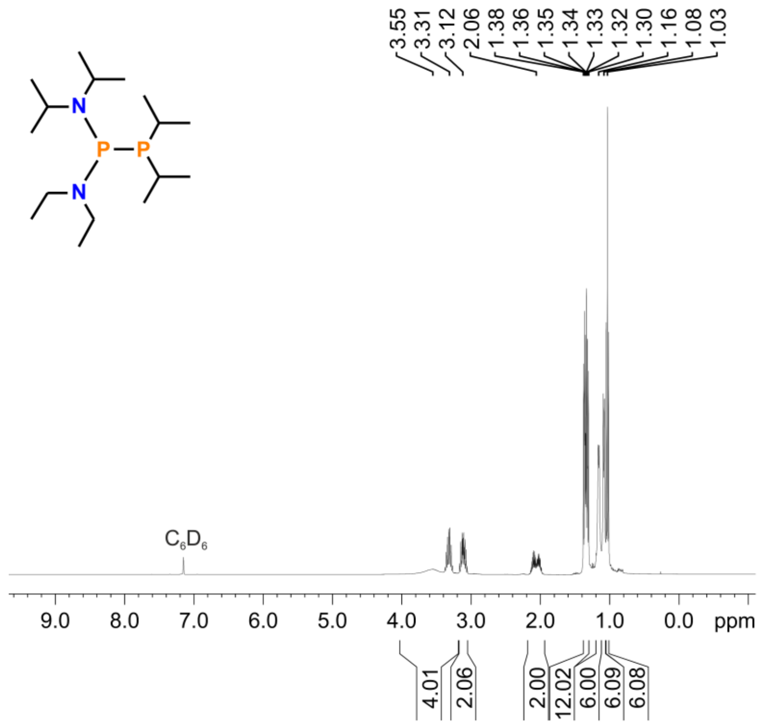

FIG. S23. ^{1}H NMR (C_6D_6) SPECTRUM OF **5**

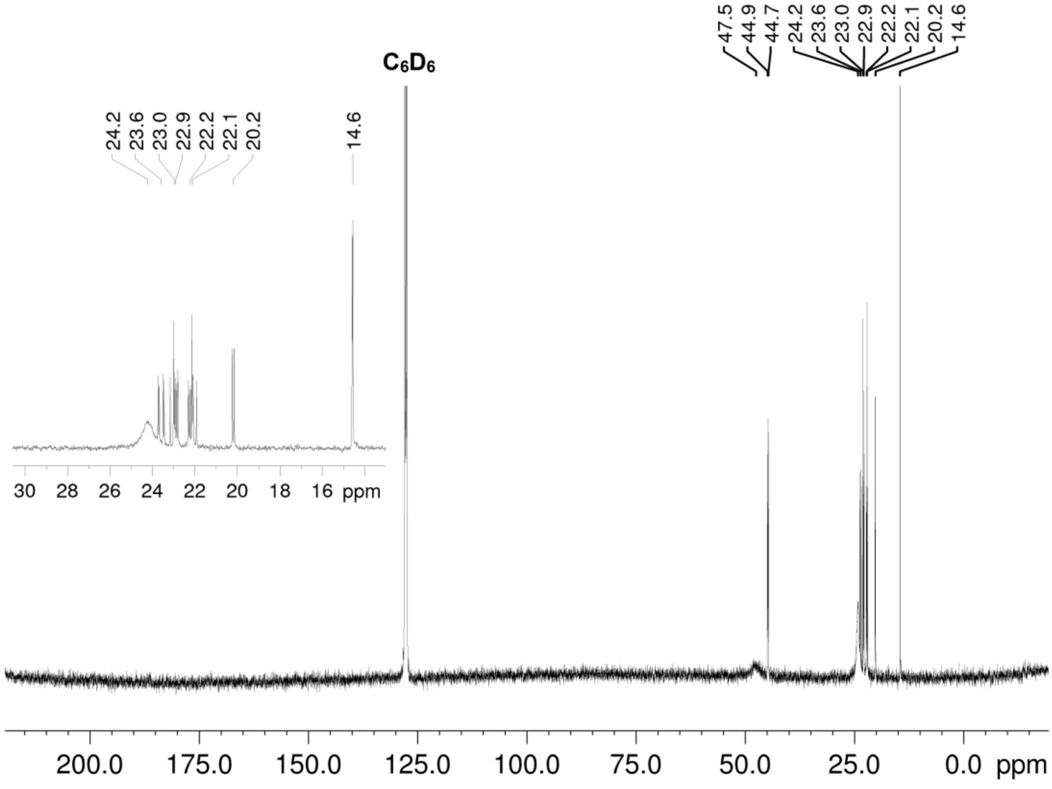

FIG. S24. $^{13}C\{^{1}H\}$ NMR (C_6D_6) SPECTRUM OF **5**

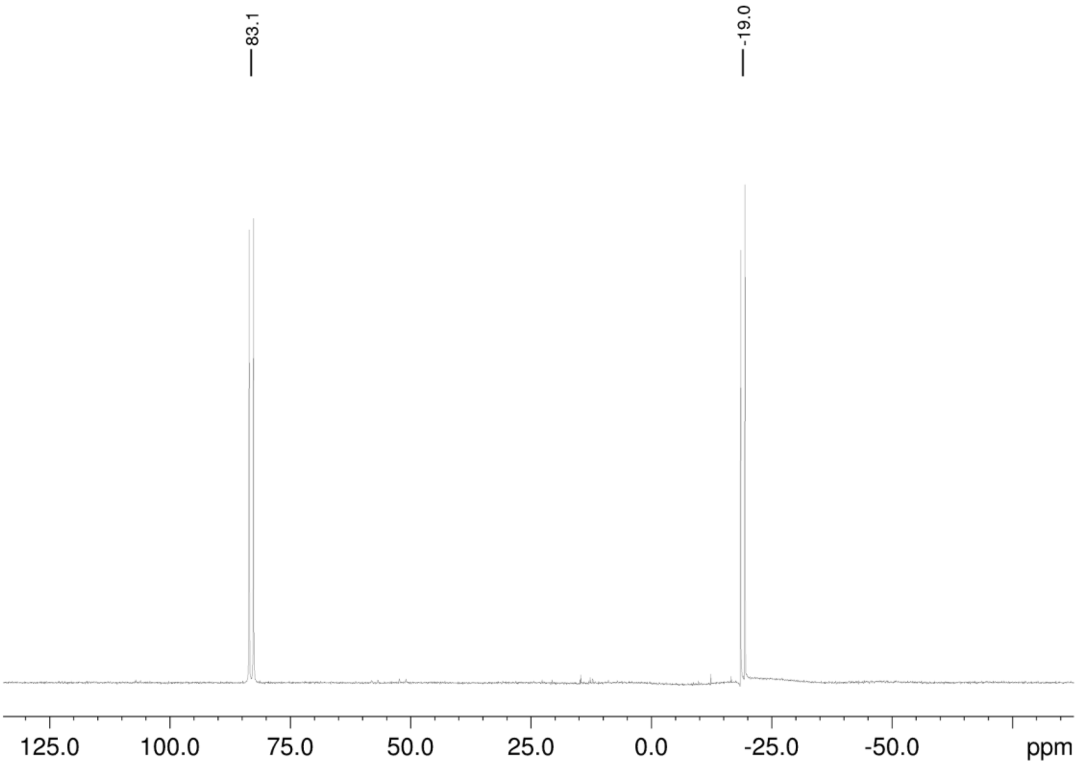

FIG. S25. $^{31}P\{^{1}H\}$ NMR (C_6D_6) SPECTRUM OF **5**

NMR spectra of 6

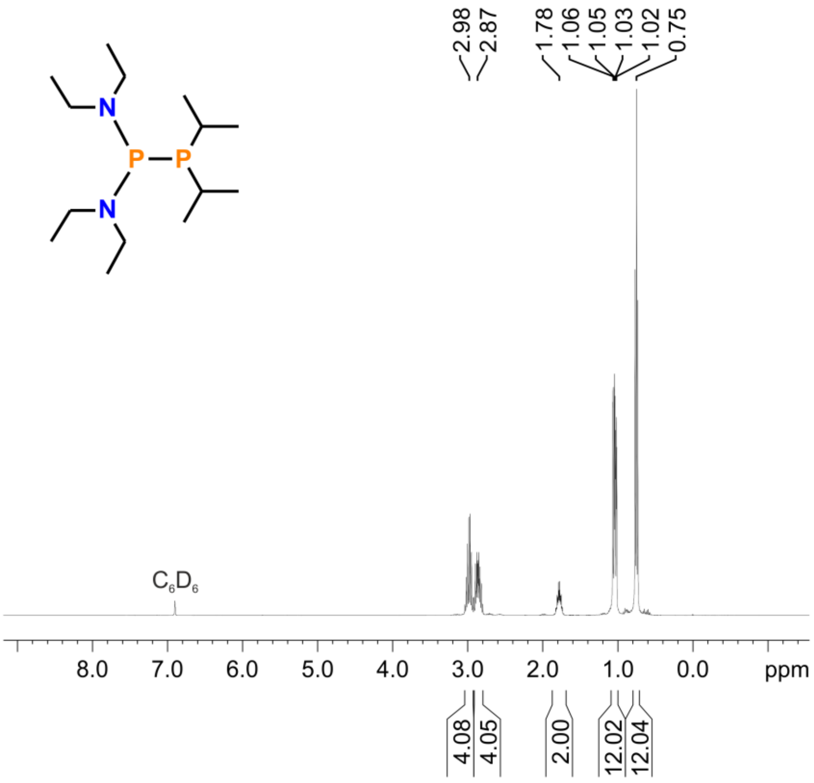

FIG. S26. ^{1}H NMR (C_6D_6) SPECTRUM OF **6**

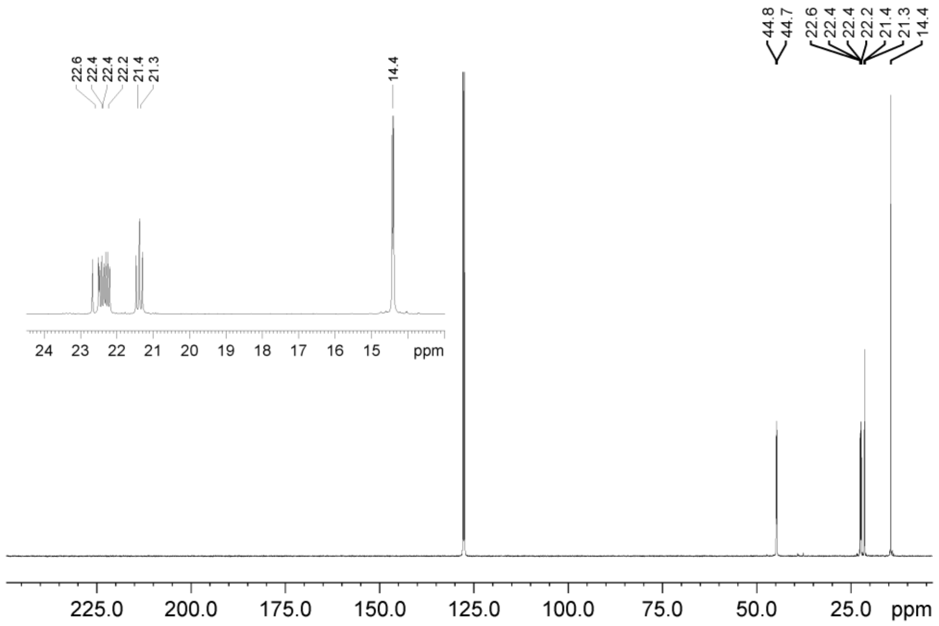

FIG. S27. $^{13}C\{^{1}H\}$ NMR (C_6D_6) SPECTRUM OF **6**

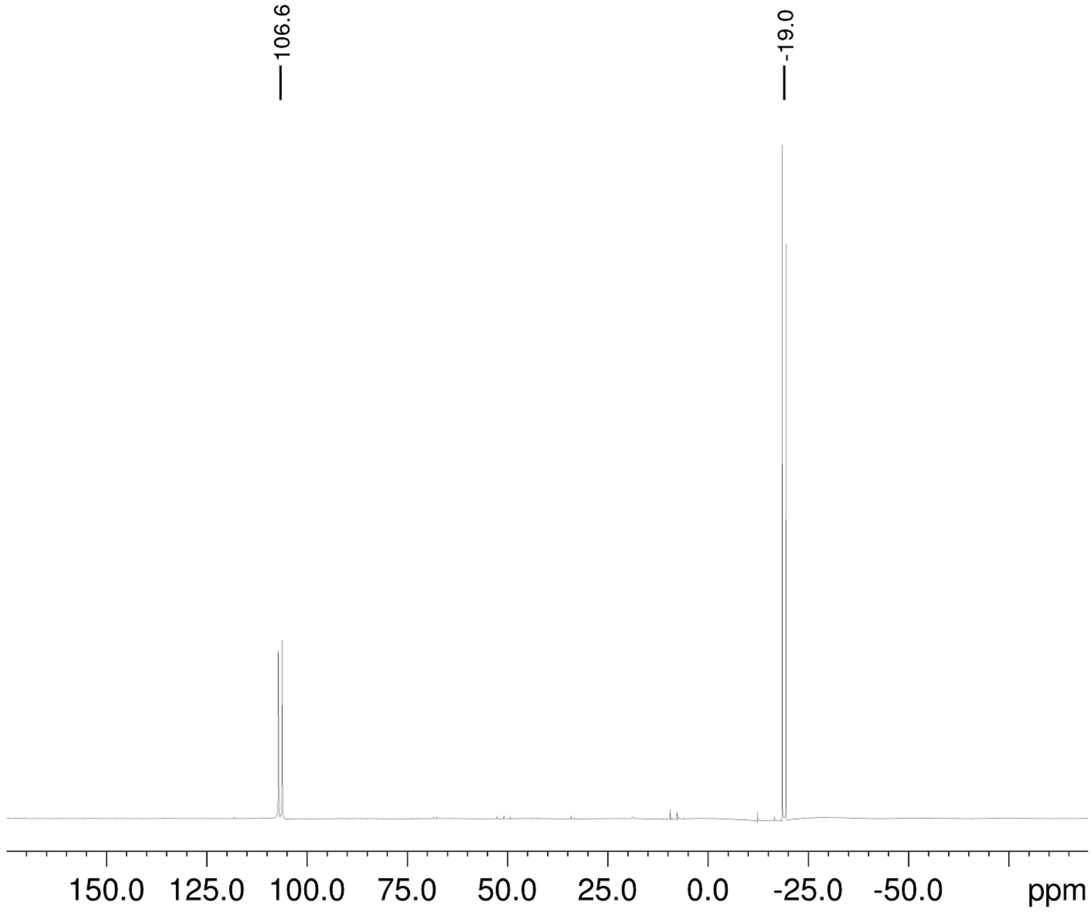

FIG. S28. $^{31}P\{^{1}H\}$ NMR (C_6D_6) SPECTRUM OF **6**

NMR spectra of 7

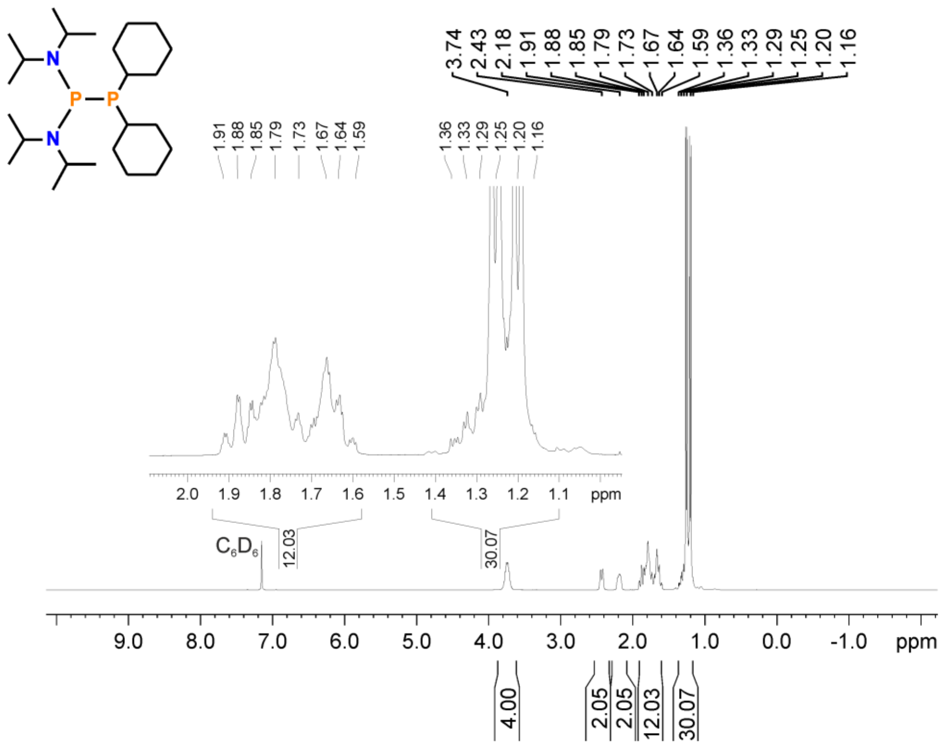

FIG. S29. ^{1}H NMR (C_6D_6) SPECTRUM OF **7**

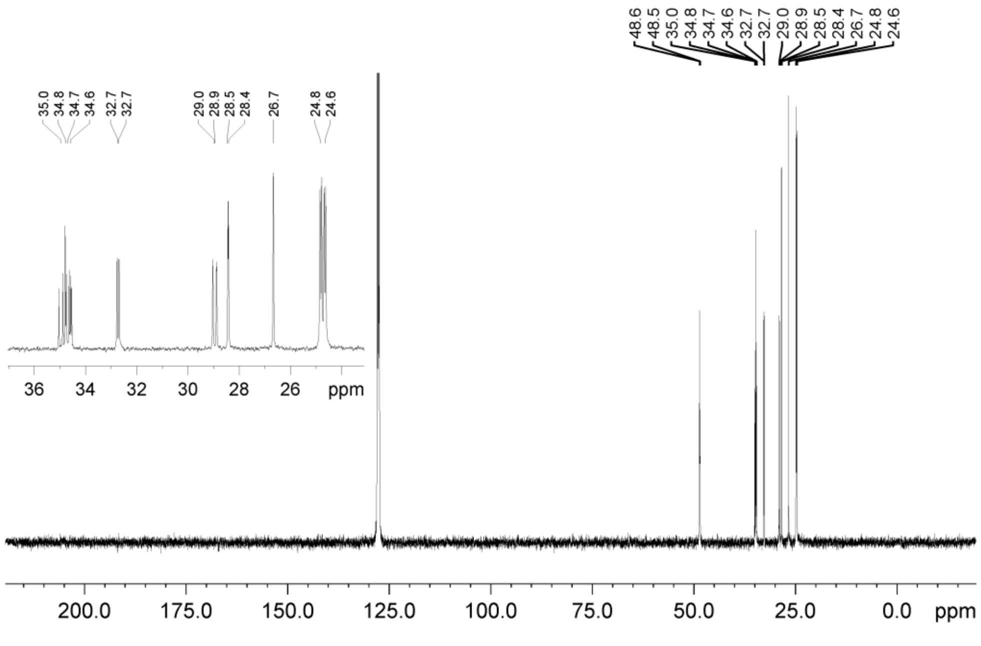

FIG. S30. $^{13}C\{^{1}H\}$ NMR (C_6D_6) SPECTRUM OF **7**

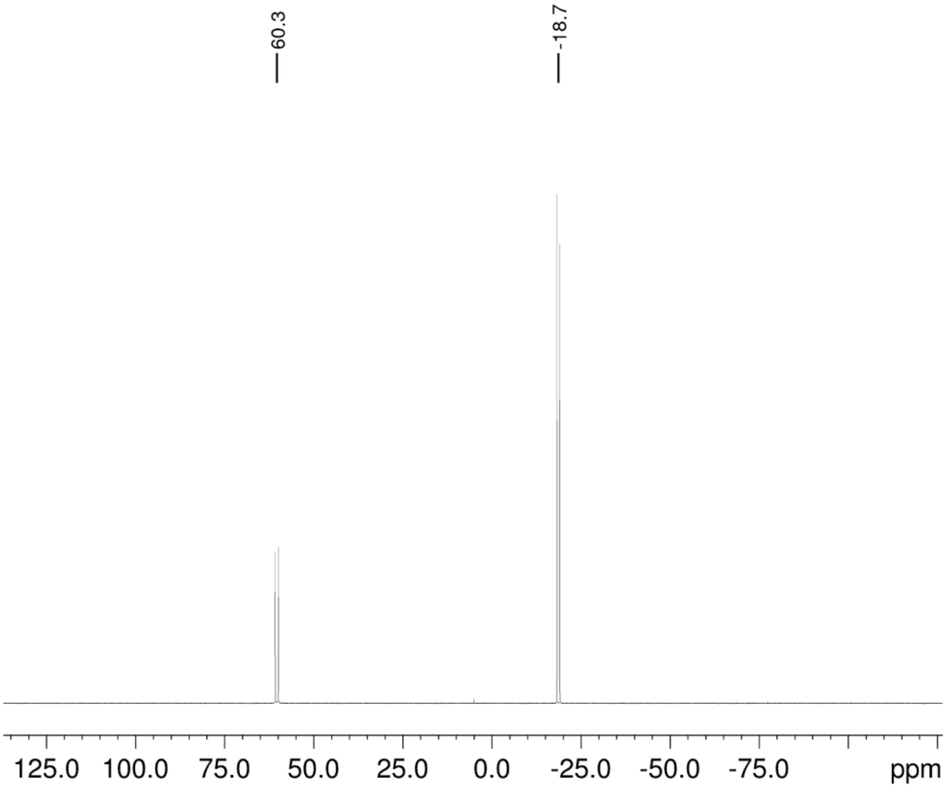

FIG. S31. $^{31}P\{^{1}H\}$ NMR (C_6D_6) SPECTRUM OF **7**

NMR spectra of 8

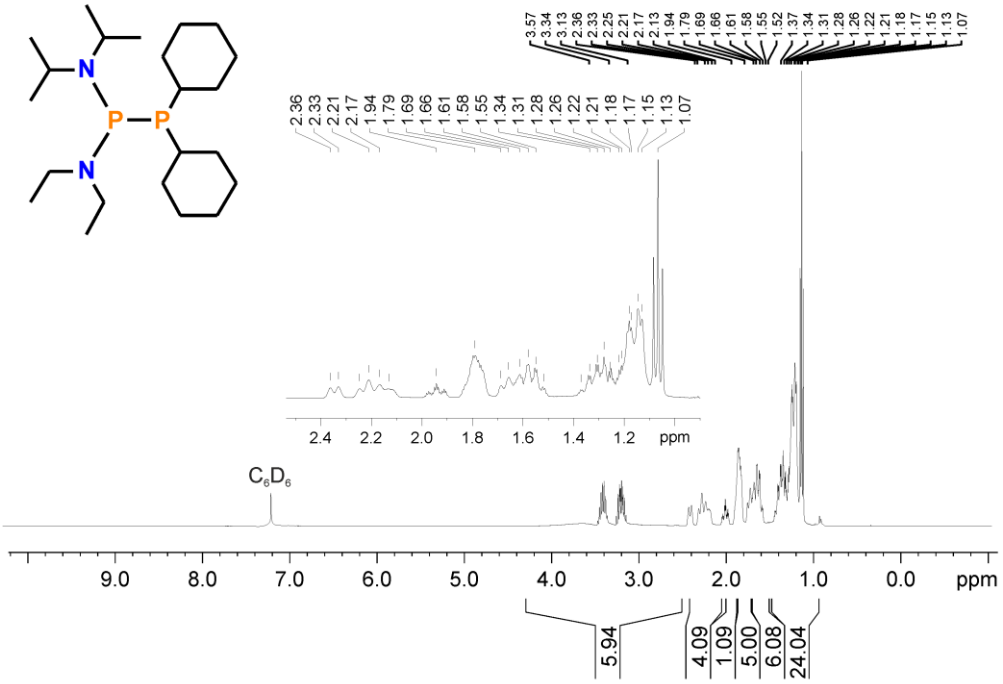

FIG. S32. ^{1}H NMR (C_6D_6) SPECTRUM OF **8**

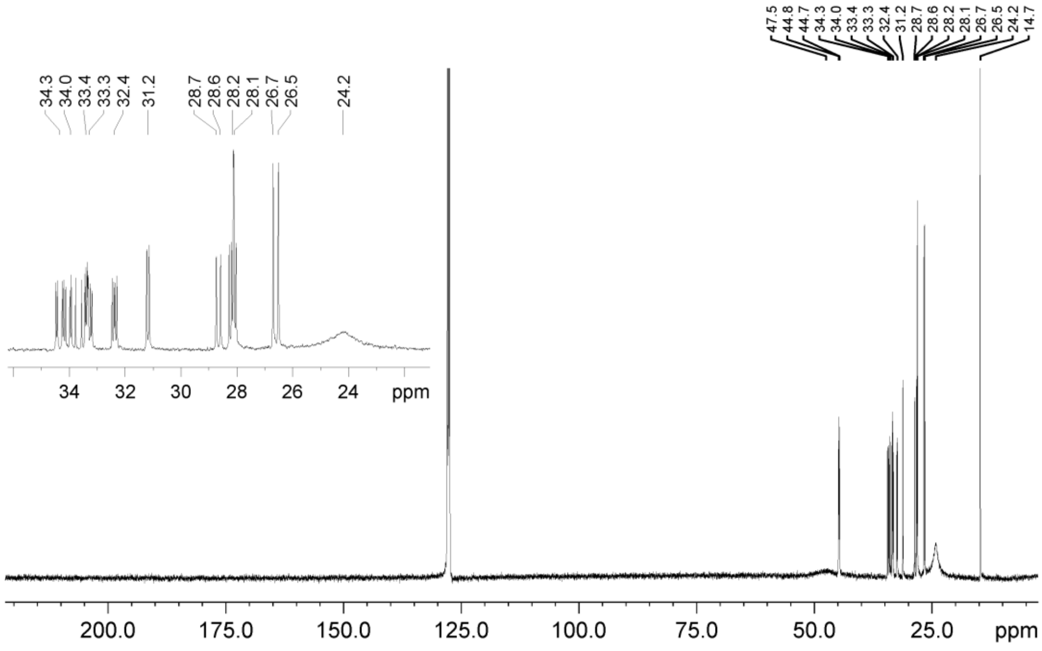

FIG. S33. $^{13}C\{^1H\}$ NMR (C_6D_6) SPECTRUM OF **8**

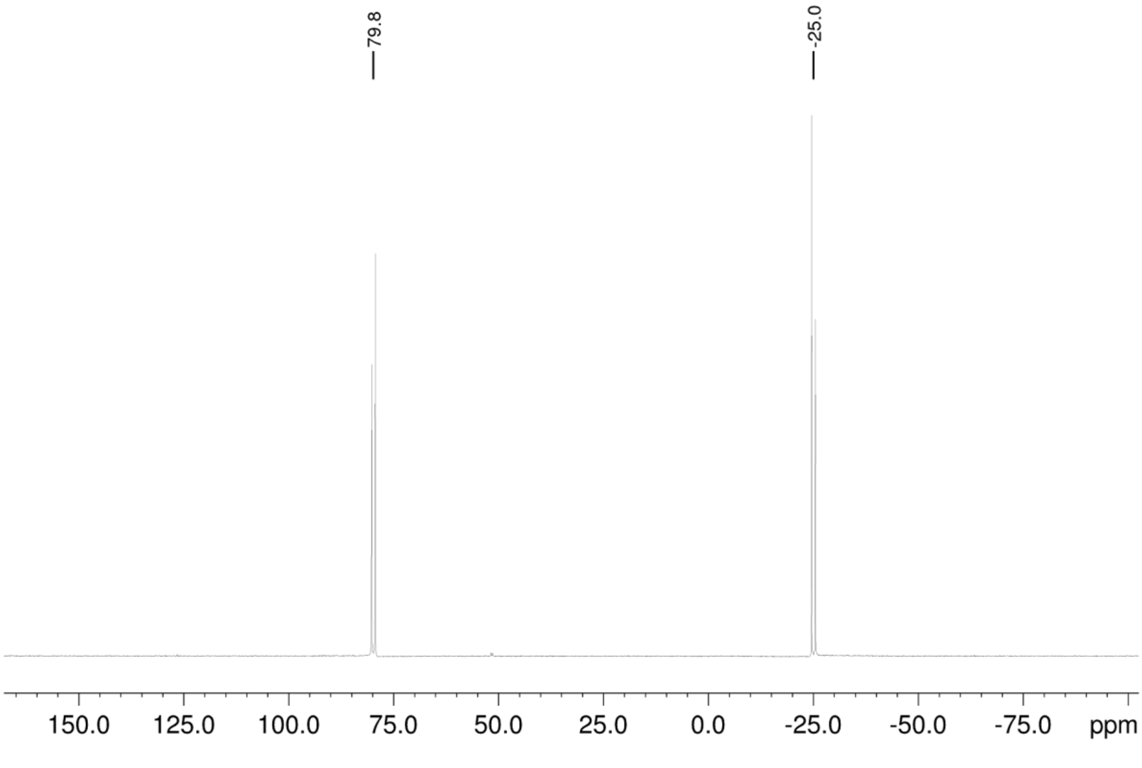

FIG. S34. $^{31}P\{^1H\}$ NMR (C_6D_6) SPECTRUM OF **8**

NMR spectra of 9

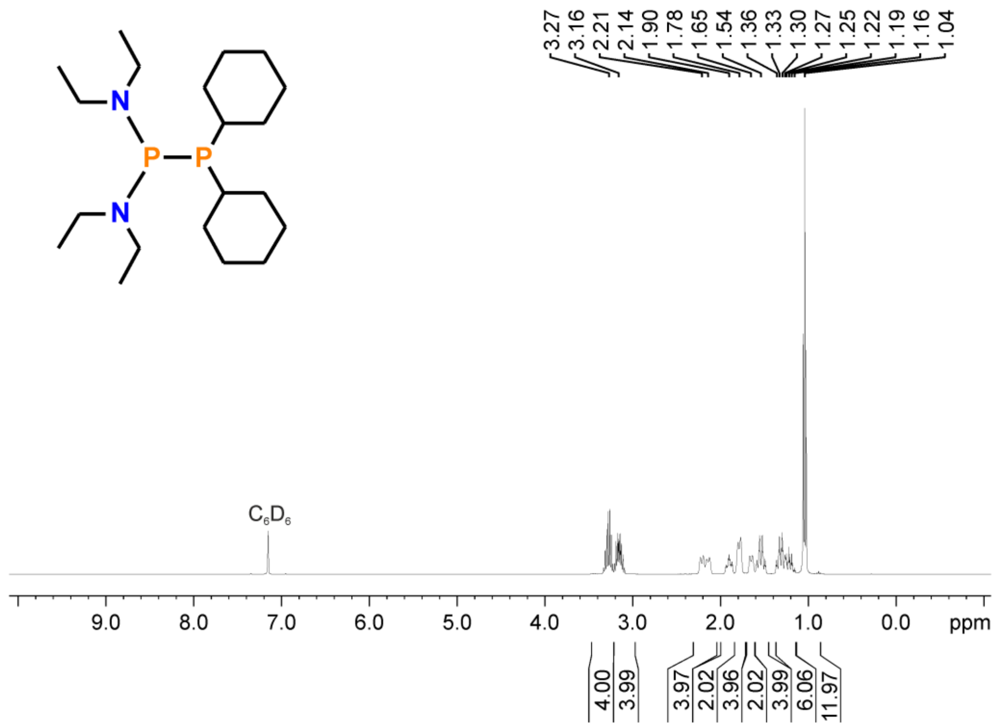

FIG. S35. ^{1}H NMR (C_6D_6) SPECTRUM OF **9**

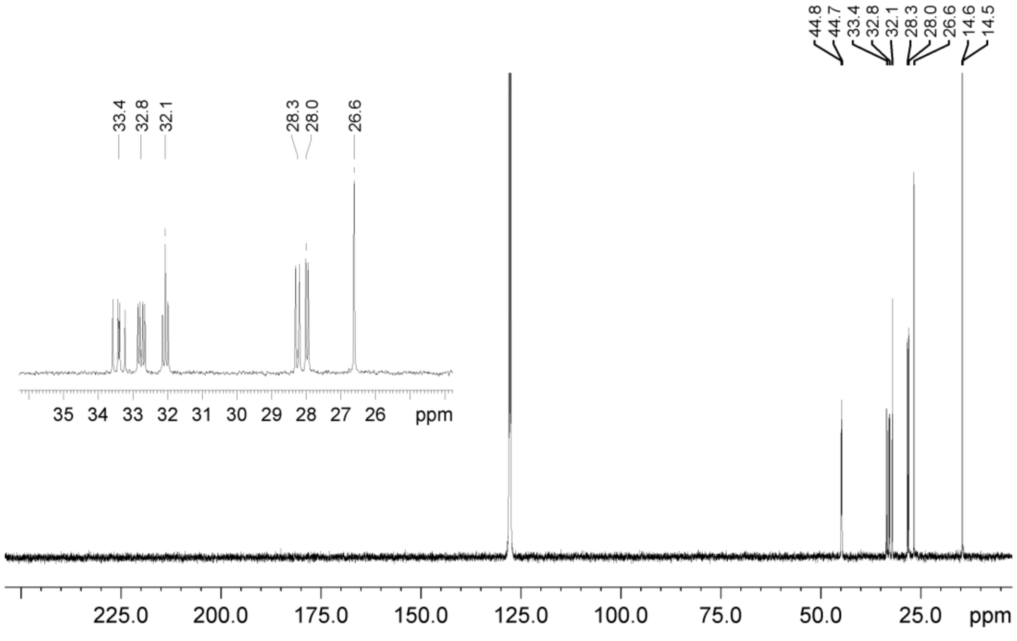

FIG. S36. $^{13}C\{^{1}H\}$ NMR (C_6D_6) SPECTRUM OF **9**

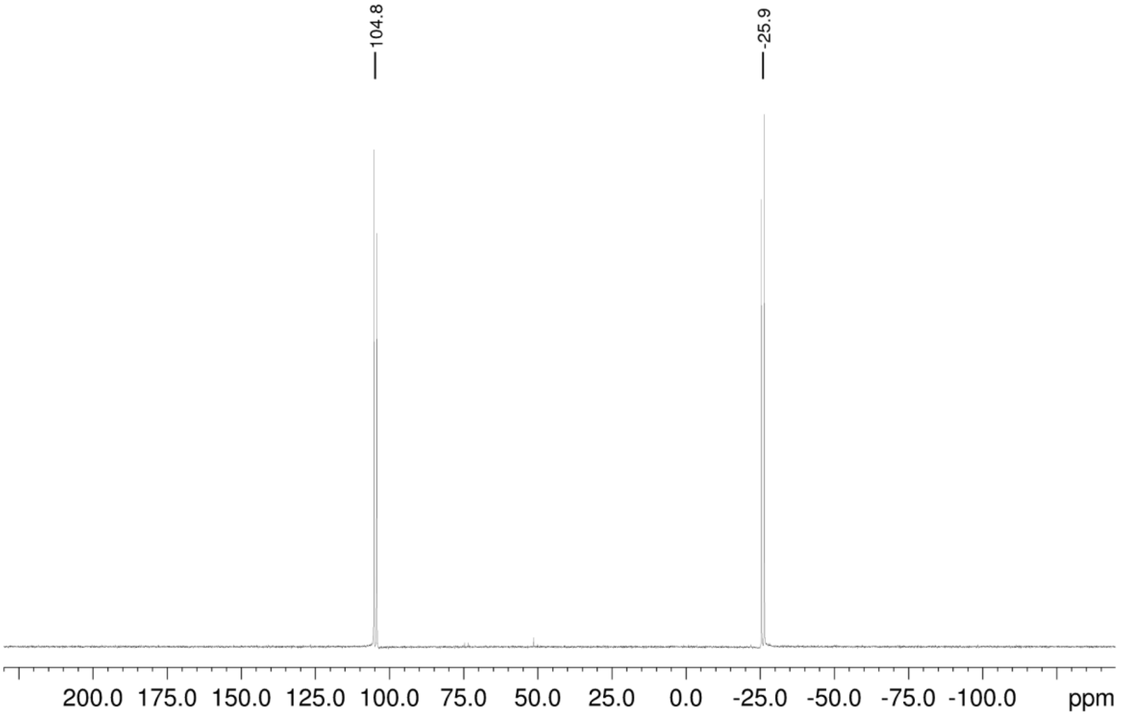

FIG. S37. $^{31}P\{^{1}H\}$ NMR (C_6D_6) SPECTRUM OF **9**

NMR spectra of 14

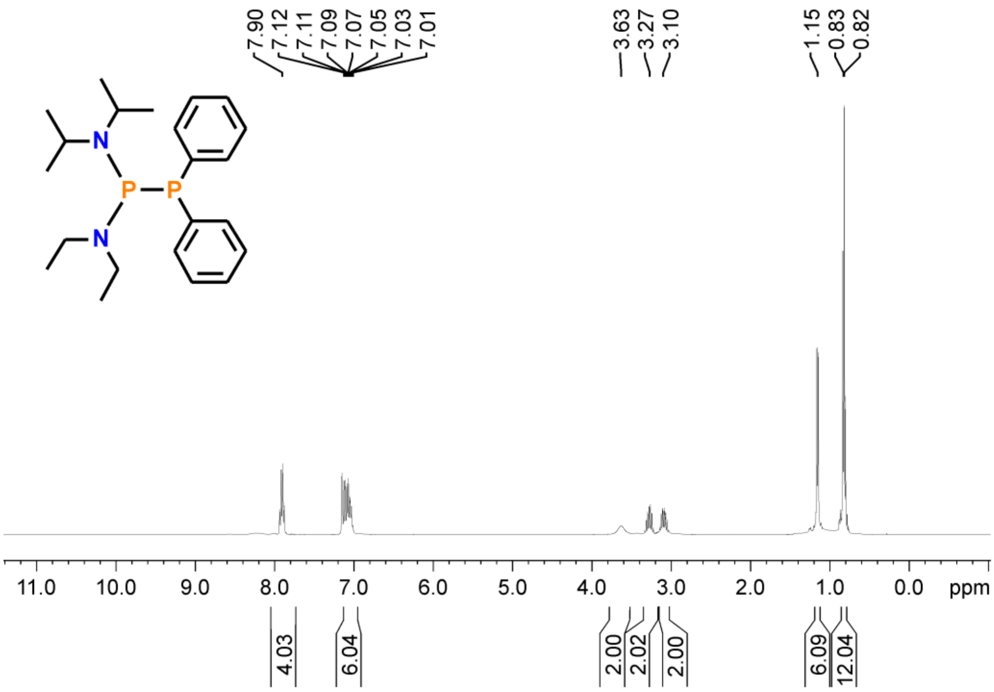

FIG. S38. ^{1}H NMR (C_6D_6) SPECTRUM OF **14**

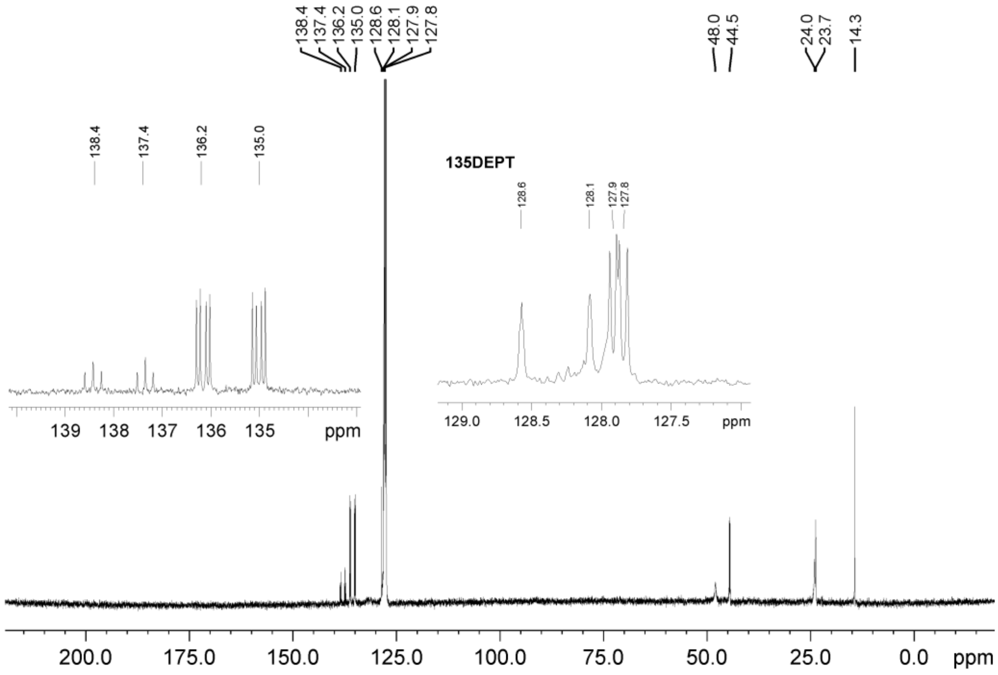

FIG. S39. $^{13}C\{^{1}H\}$ NMR (C_6D_6) SPECTRUM OF **14**

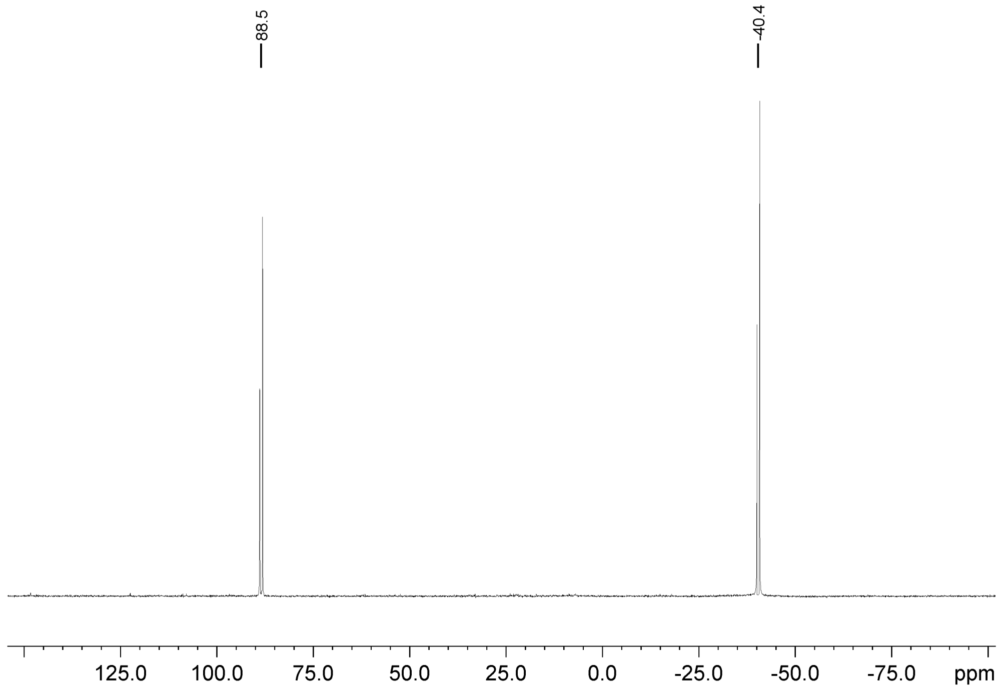

FIG. S40. $^{31}P\{^{1}H\}$ NMR (C_6D_6) SPECTRUM OF **14**

NMR spectra of 2a$_{Pt}$

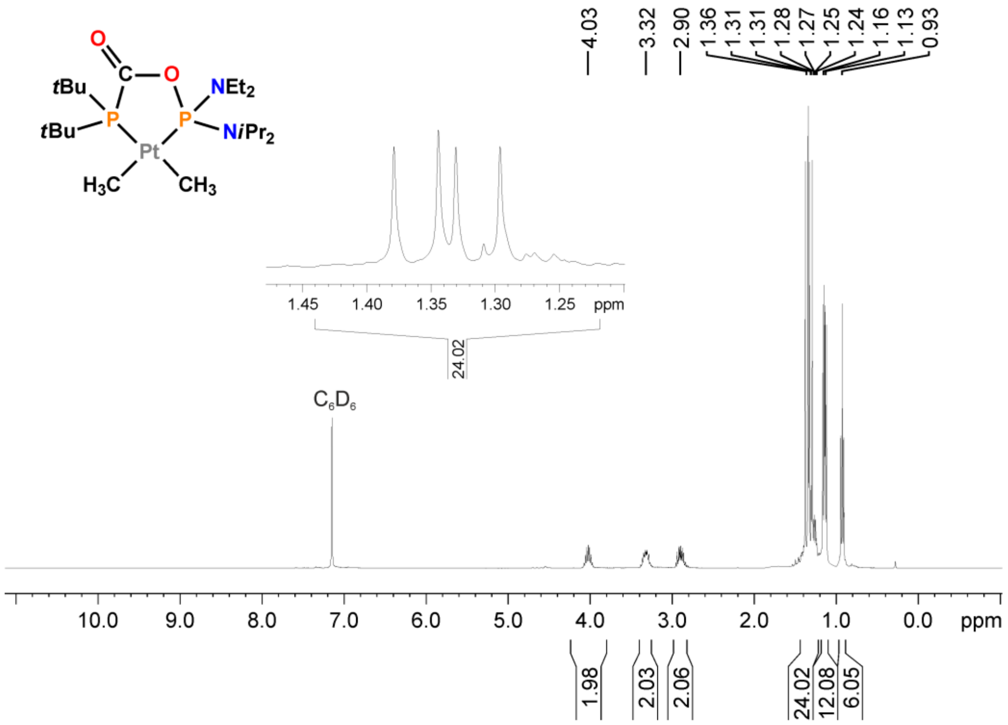

FIG. S41. ^{1}H NMR (C_6D_6) SPECTRUM OF **2A$_{Pt}$**

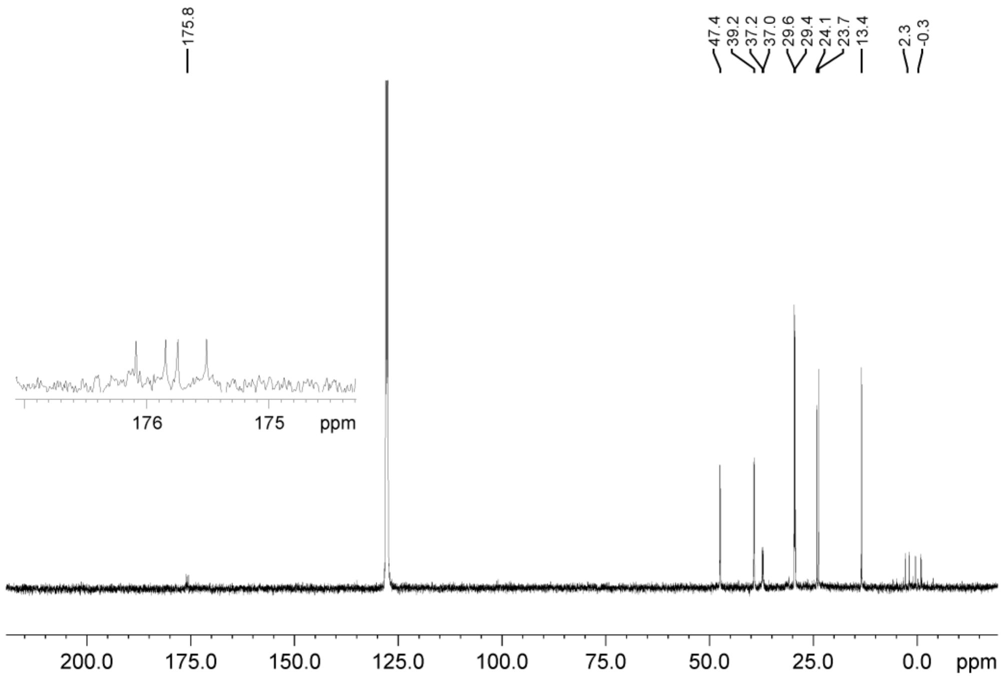

FIG. S42. $^{13}C\{^1H\}$ NMR (C_6D_6) SPECTRUM OF **2A$_{Pt}$**

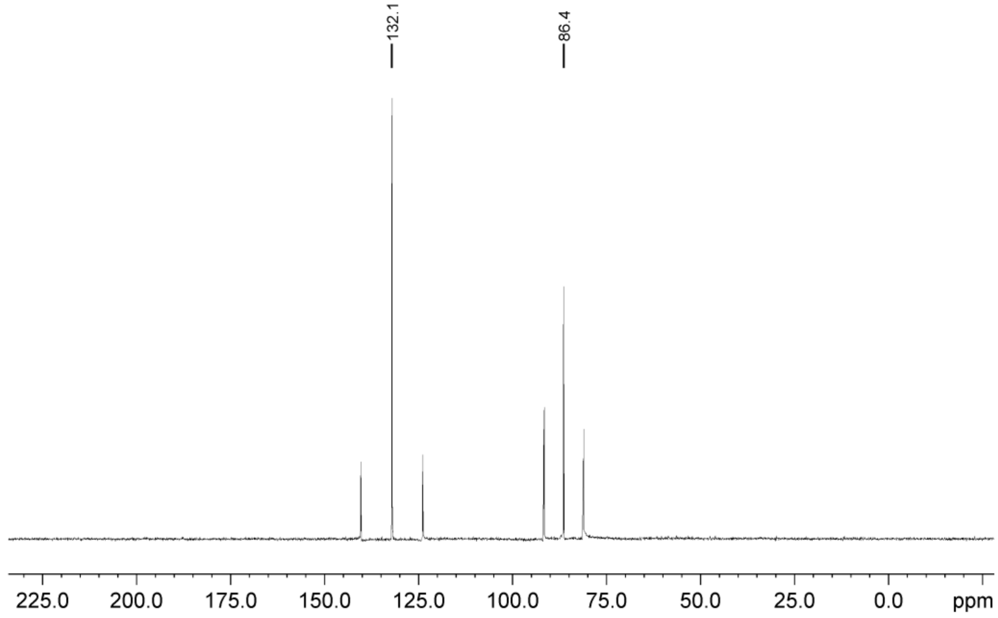

FIG. S43. $^{31}P\{^{1}H\}$ NMR (C_6D_6) SPECTRUM OF **$2A_{Pt}$**

NMR spectra of 4b

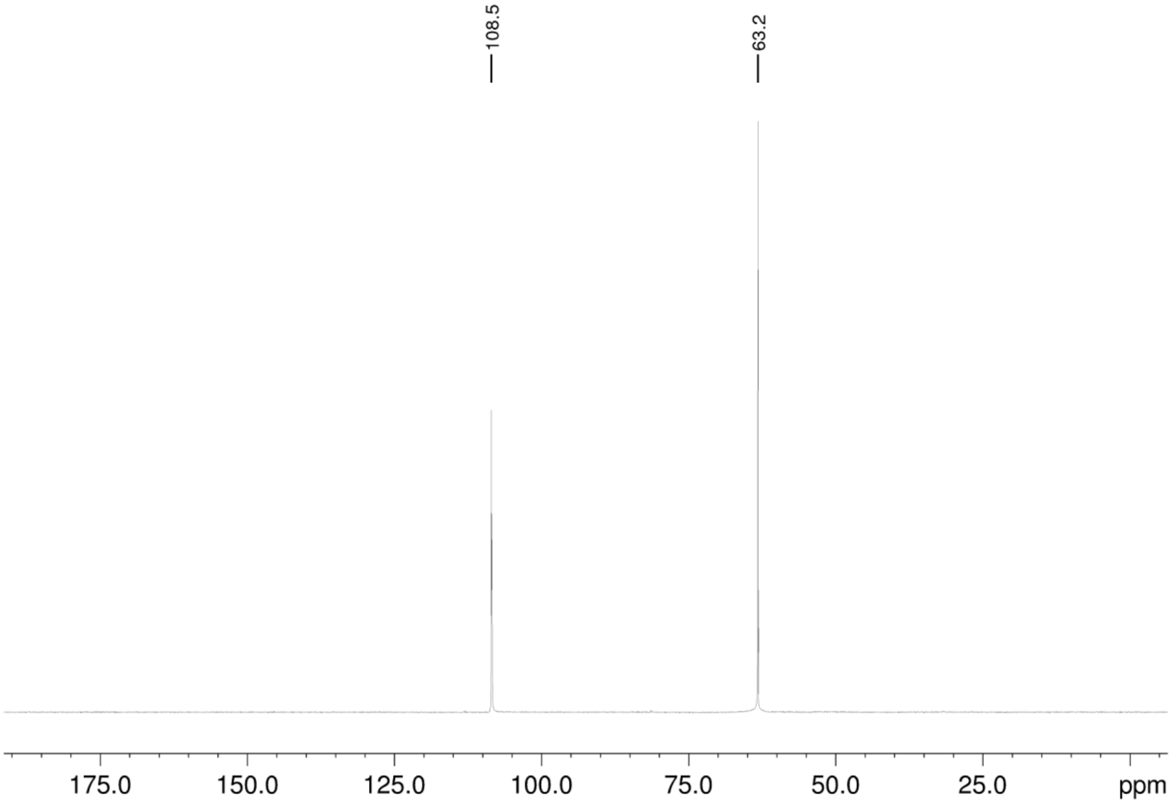

FIG. S44. ^{1}H NMR (C_6D_6) SPECTRUM OF **4B**

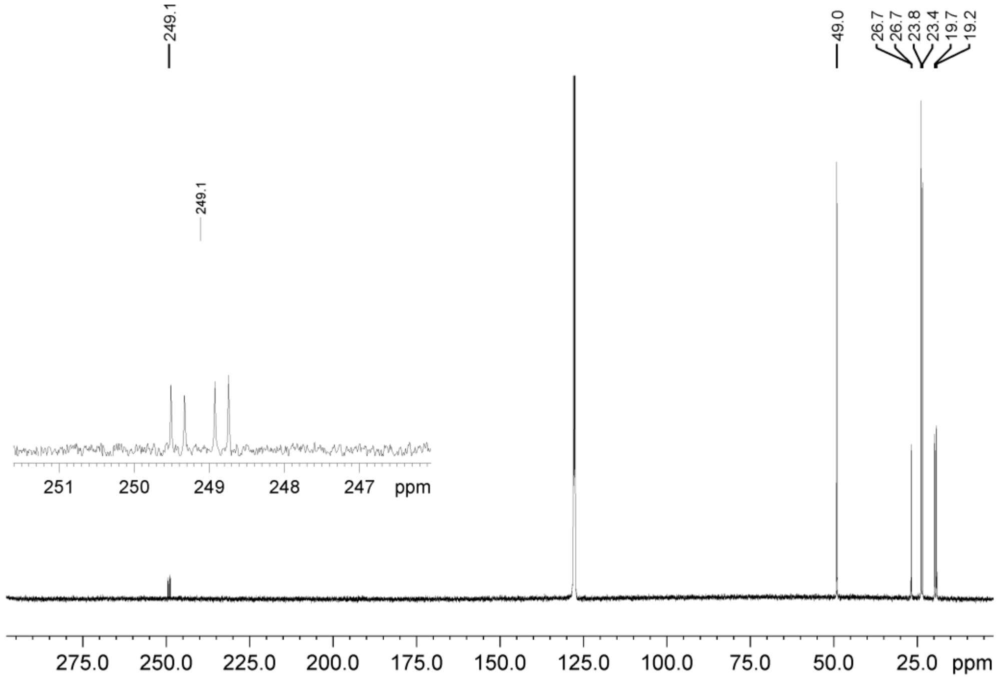

FIG. S45. $^{13}C\{^{1}H\}$ NMR (C_6D_6) SPECTRUM OF **4B**

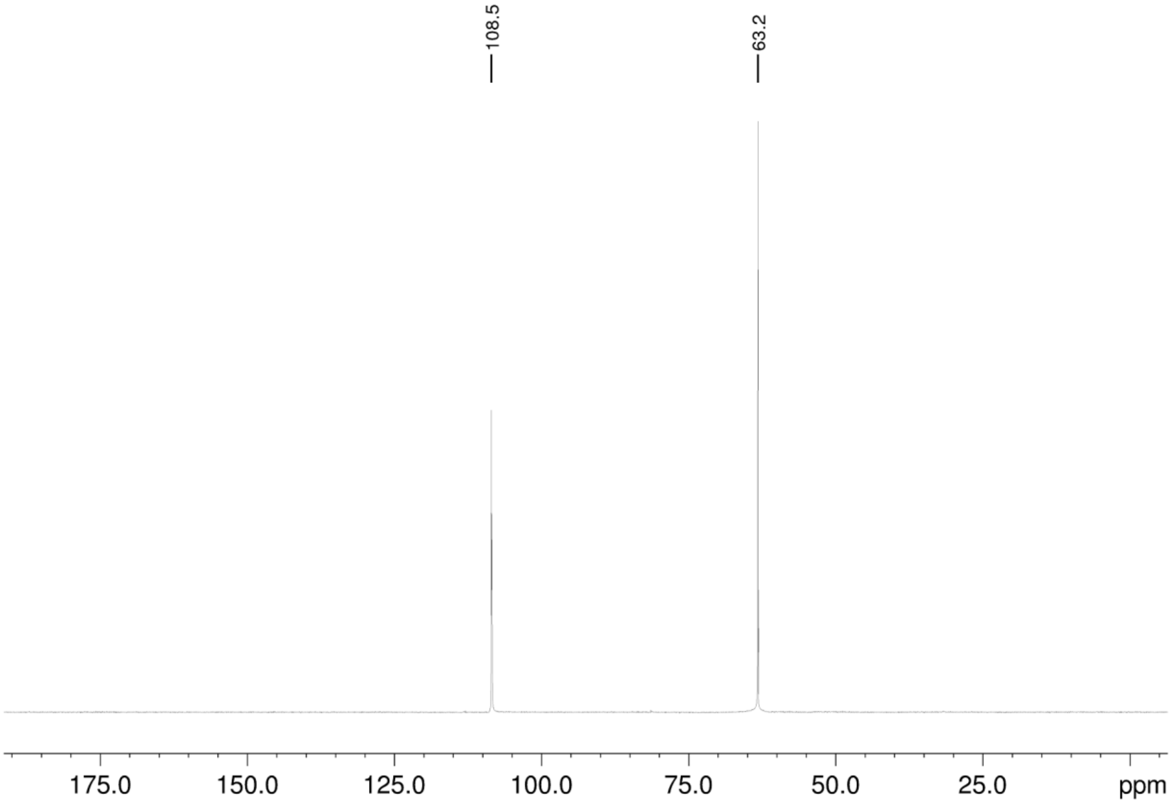

FIG. S46. $^{31}P\{^{1}H\}$ NMR (C_6D_6) SPECTRUM OF **4B**

NMR spectra of 7b

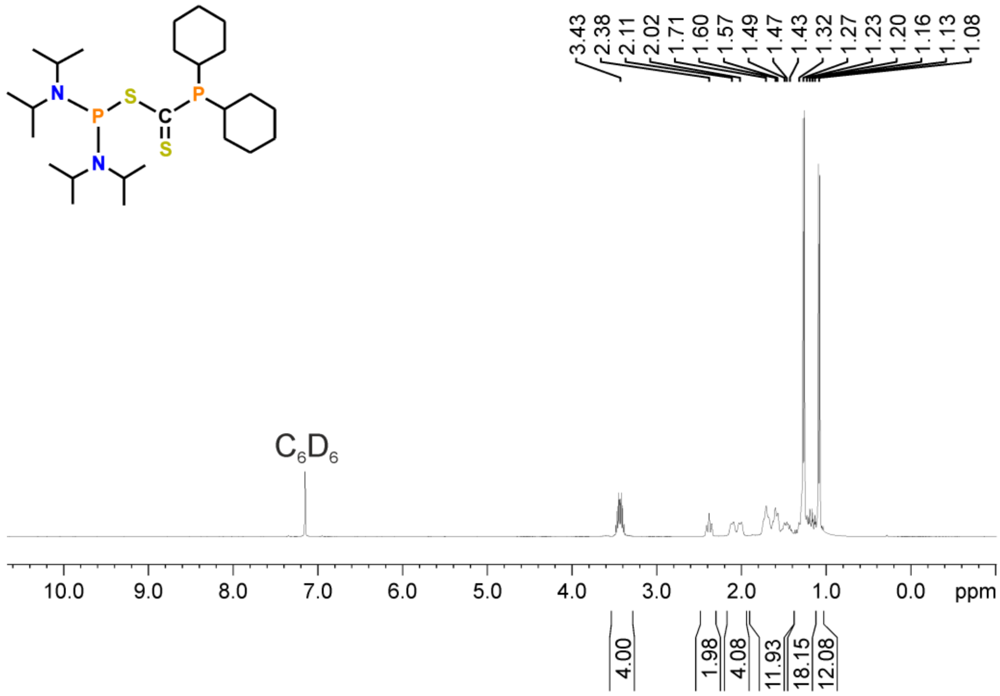

FIG. S47. ^{1}H NMR (C_6D_6) SPECTRUM OF **7B**

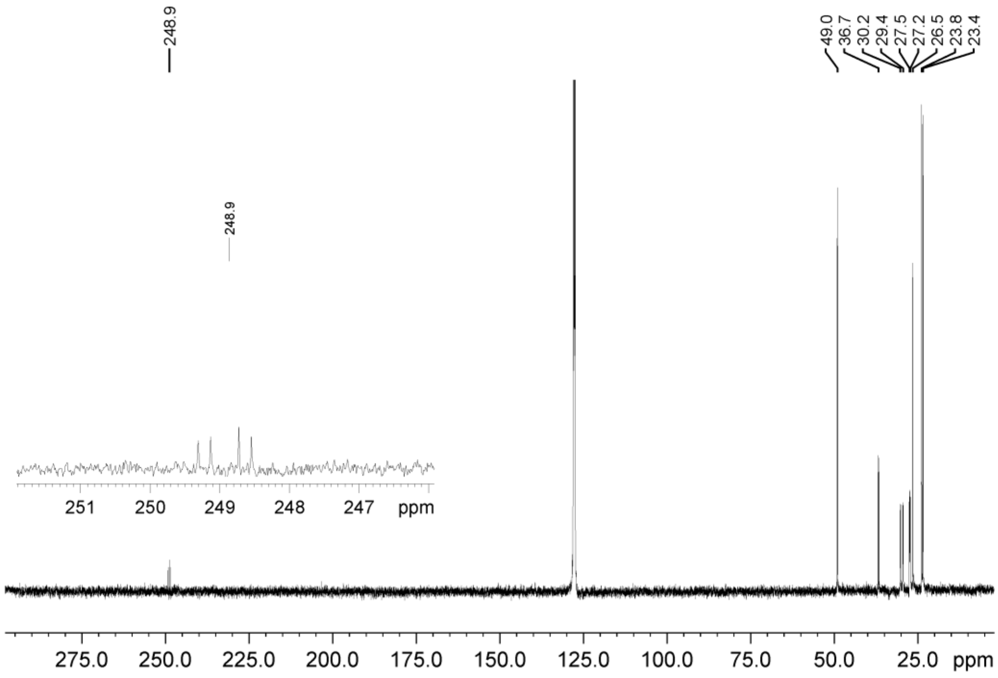

FIG. S48. $^{13}C\{^{1}H\}$ NMR (C_6D_6) SPECTRUM OF **7B**

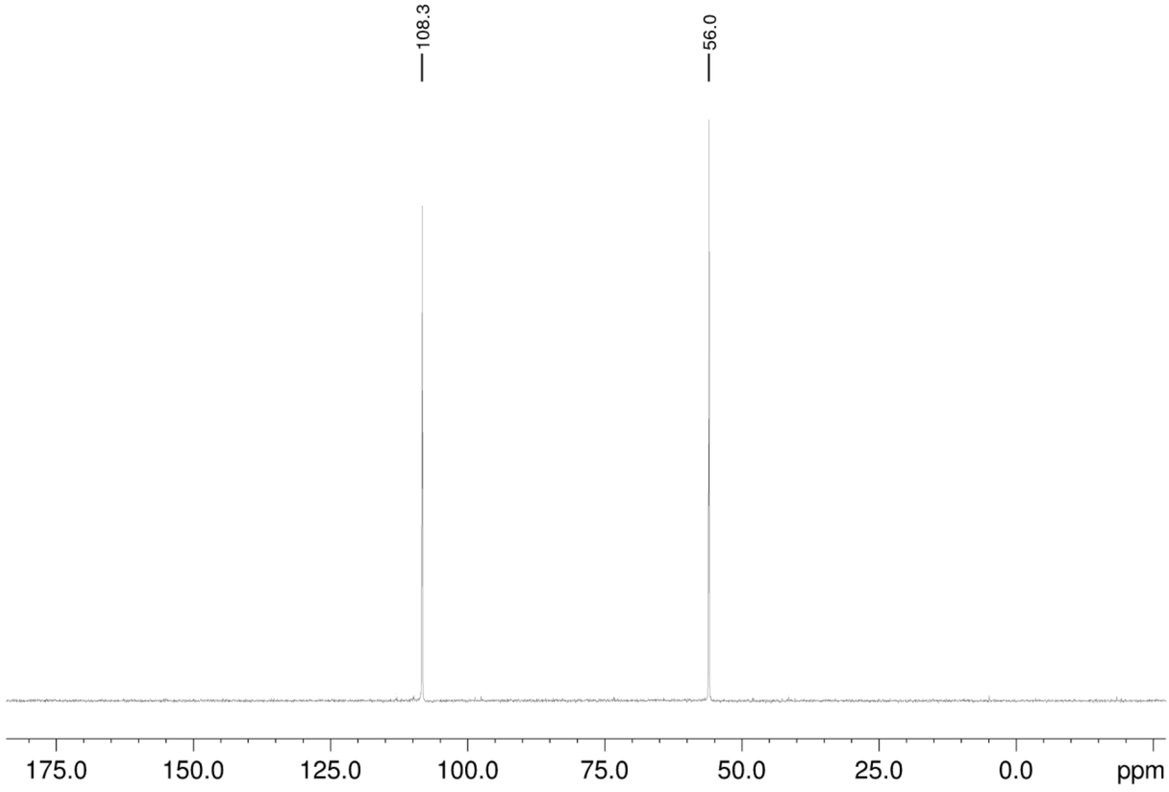

FIG. S49. $^{31}P\{^{1}H\}$ NMR (C_6D_6) SPECTRUM OF **7B**

NMR spectra of 10b

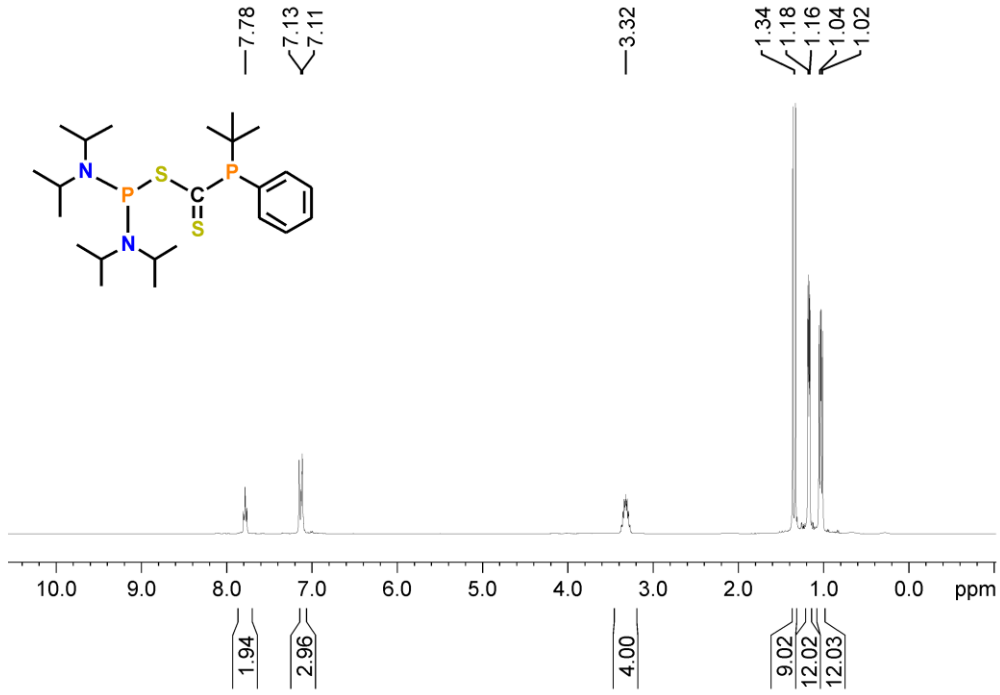

FIG. S50. ^{1}H NMR (C_6D_6) SPECTRUM OF **10B**

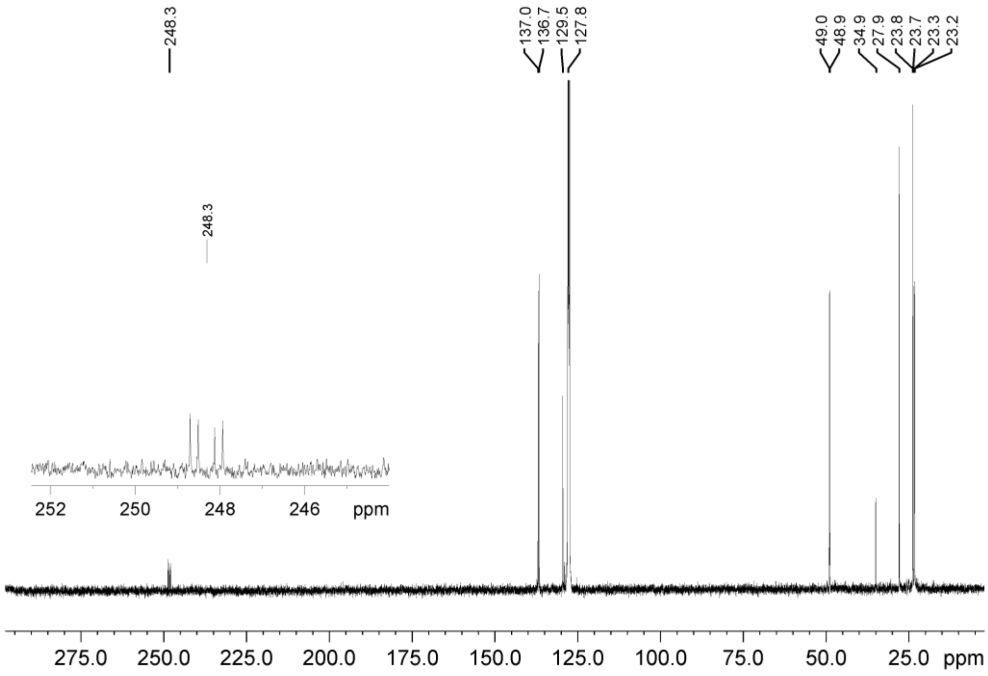

FIG. S51. $^{13}C\{^{1}H\}$ NMR (C_6D_6) SPECTRUM OF **10B**

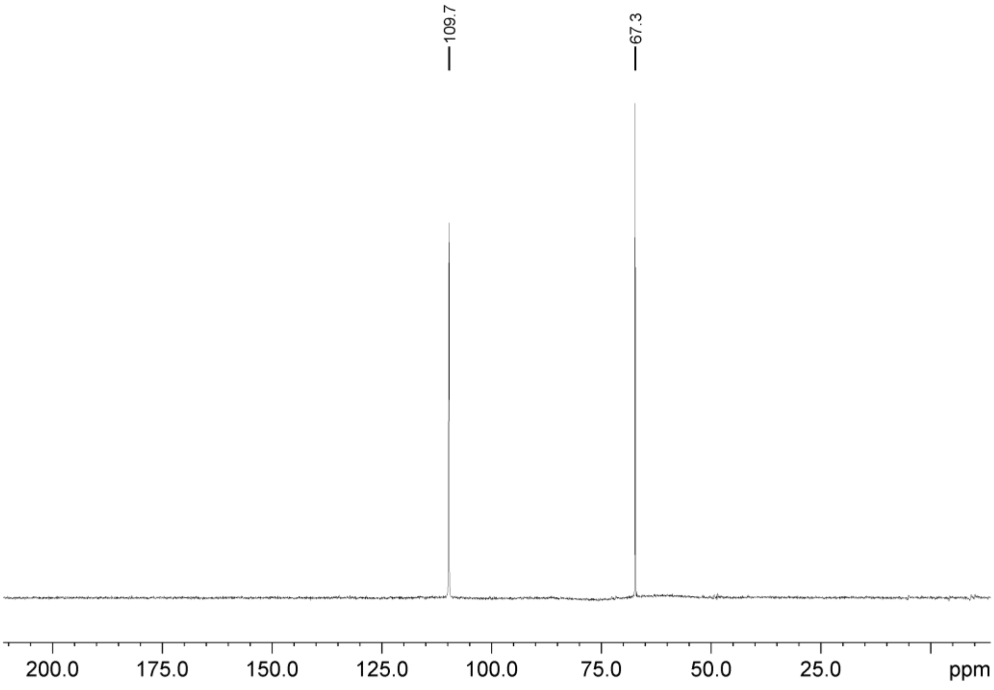

FIG. S52. $^{31}P\{^{1}H\}$ NMR (C_6D_6) SPECTRUM OF **10B**

NMR spectra of 1c and 1c’

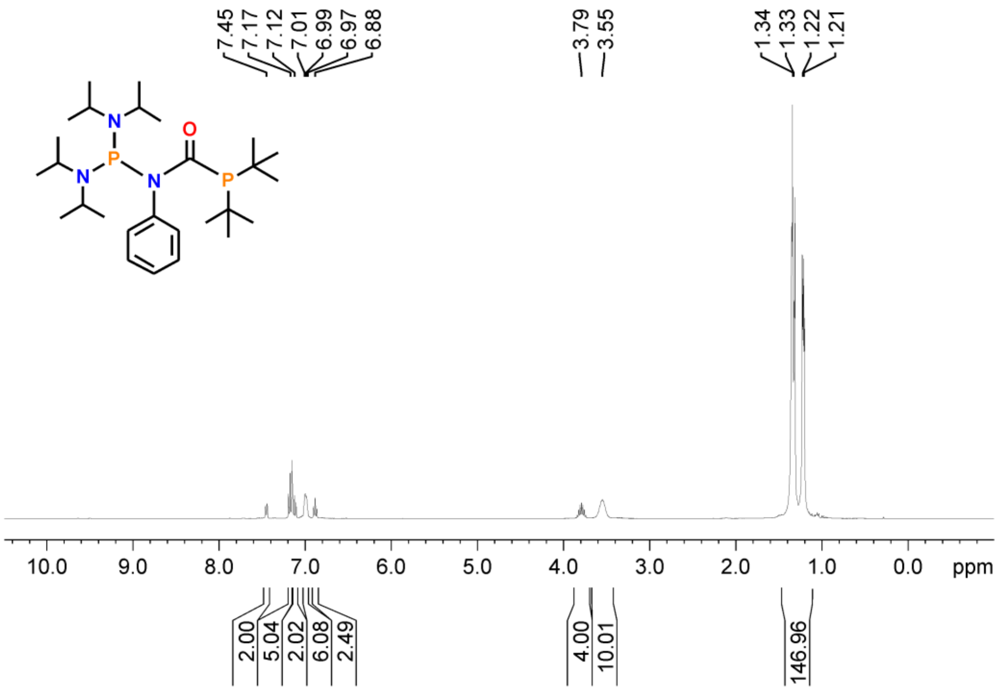

FIG. S53. ^{1}H NMR (C_6D_6) SPECTRUM OF **1C** AND **1C’**

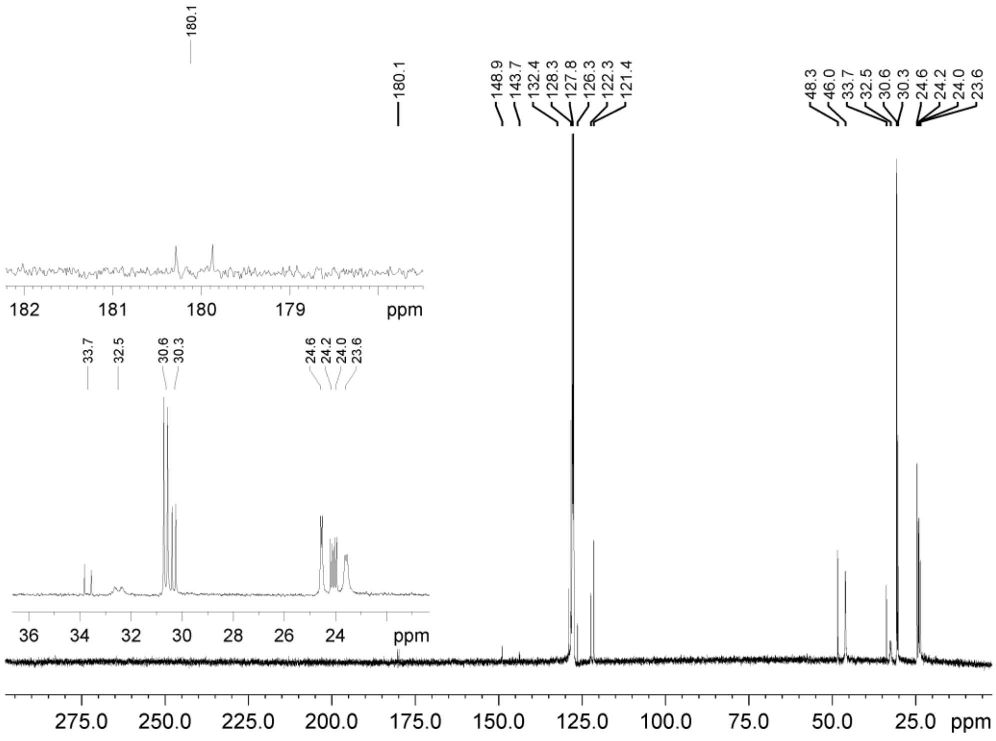

FIG. S54. ^{13}C{^{1}H} NMR (C_6D_6) SPECTRUM OF **1C** AND **1C’**

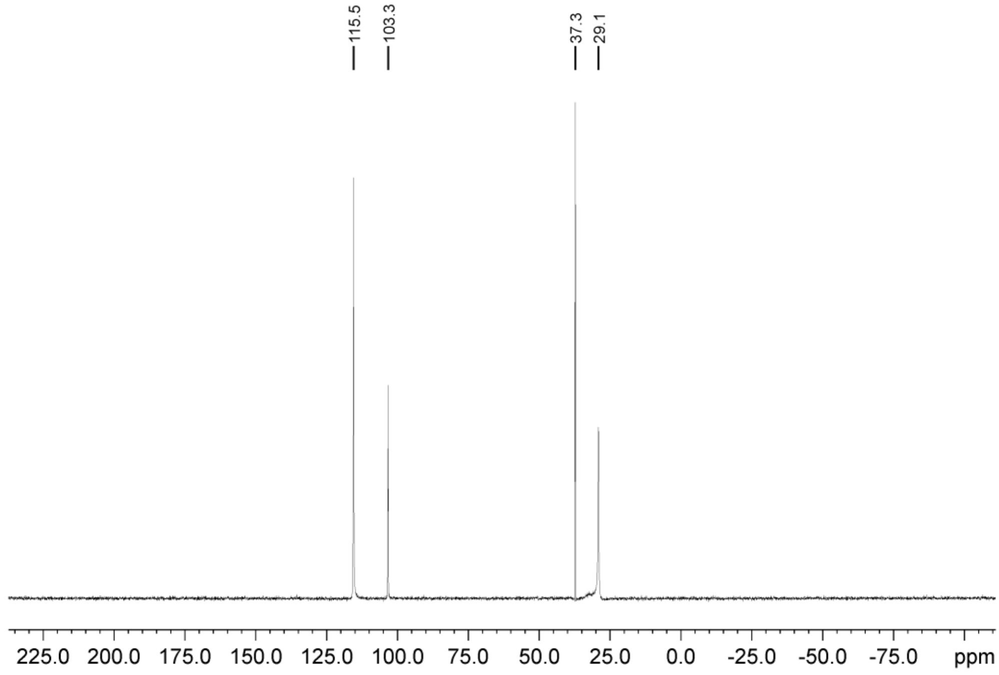

FIG. S55. $^{31}P\{^{1}H\}$ NMR (C_6D_6) SPECTRUM OF **1C** AND **1C'**

NMR spectra of 2c

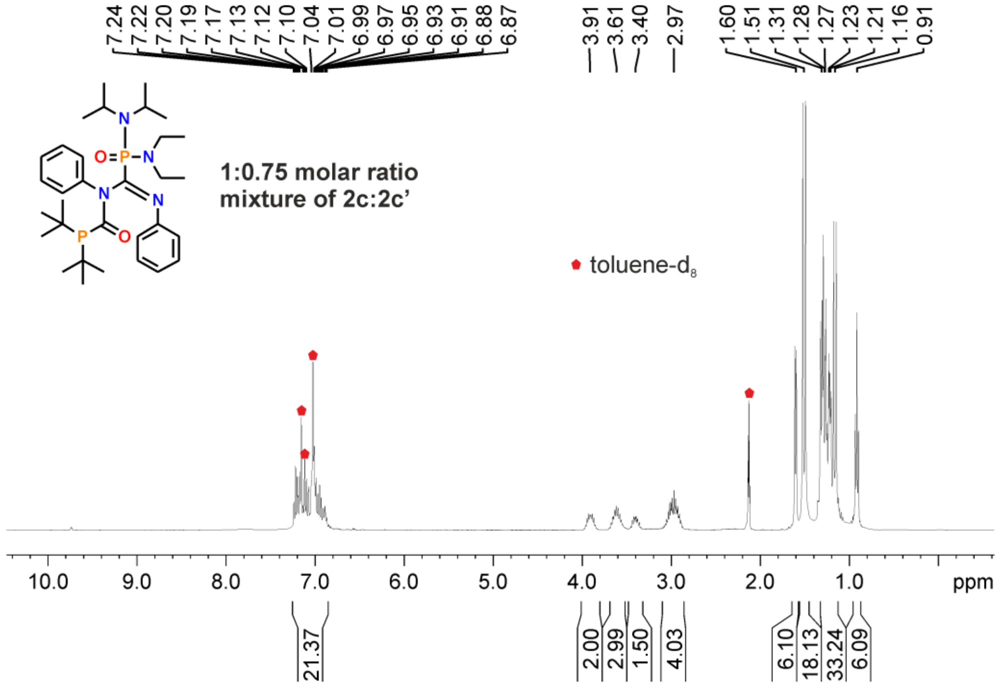

FIG. S56. ^{1}H NMR (TOLUENE-D_8, 273K) SPECTRUM OF **2C**

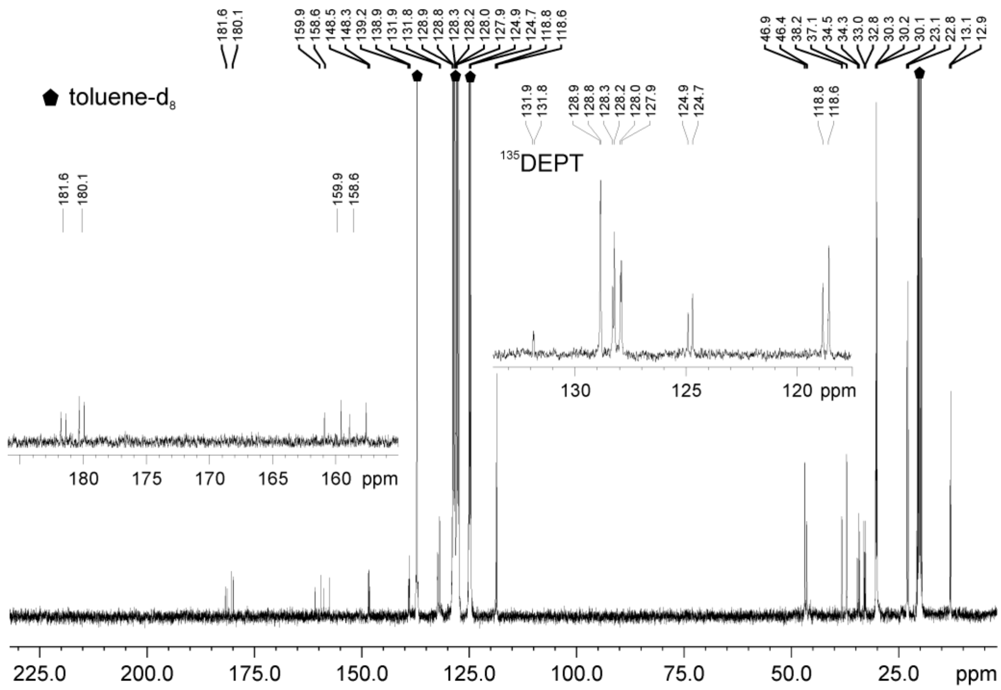

FIG. S57. $^{13}C\{^{1}H\}$ NMR (TOLUENE-D$_8$, 273K) SPECTRUM OF **2C**

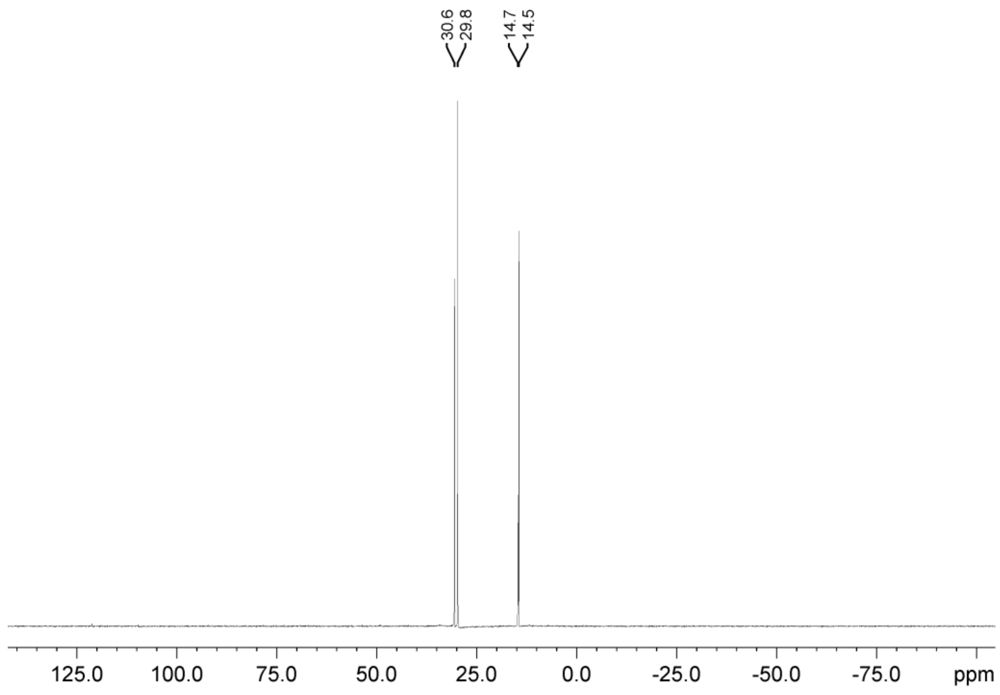

FIG. S58. $^{31}P\{^{1}H\}$ NMR (TOLUENE-D$_8$, 273K) SPECTRUM OF **2C**

NMR spectra of 3c

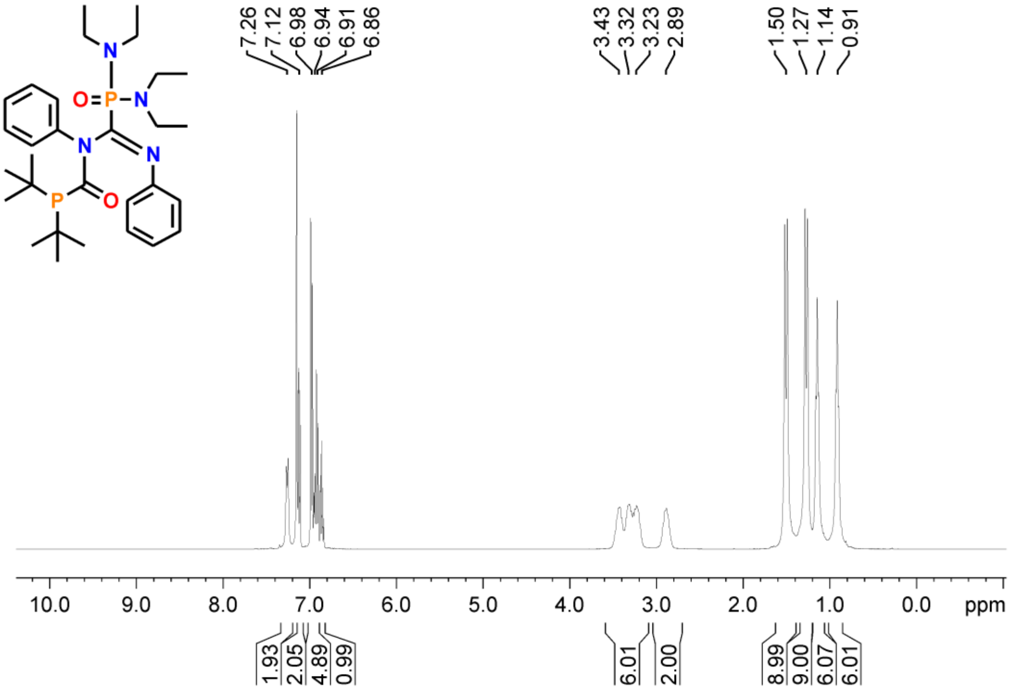

FIG. S59. ^{1}H NMR (C_6D_6) SPECTRUM OF **3C**

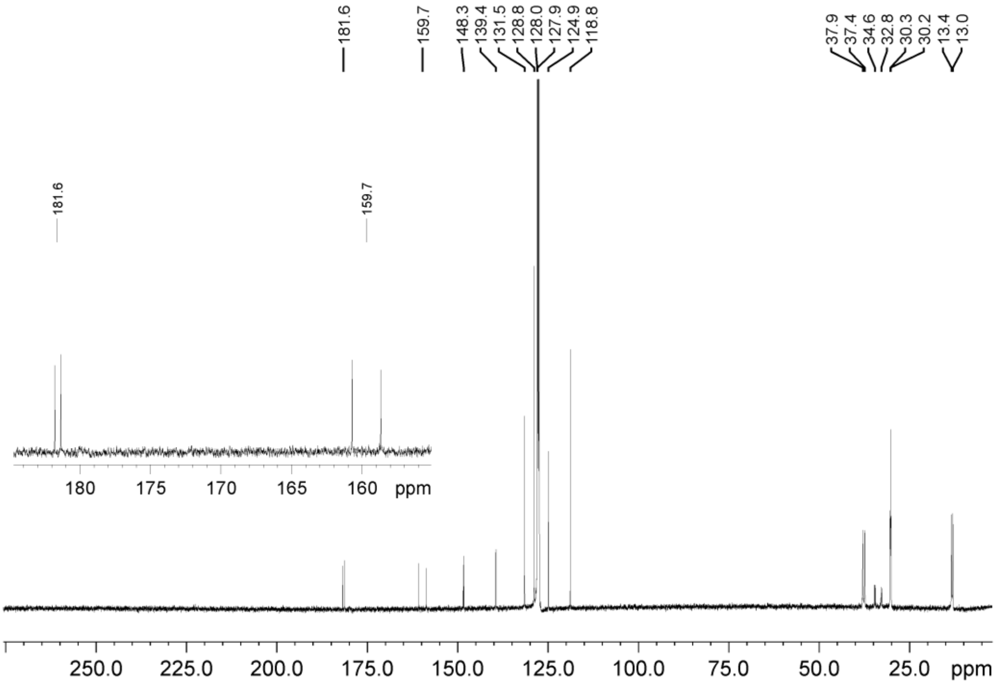

FIG. S60. ^{13}C{^{1}H} NMR (C_6D_6) SPECTRUM OF **3C**

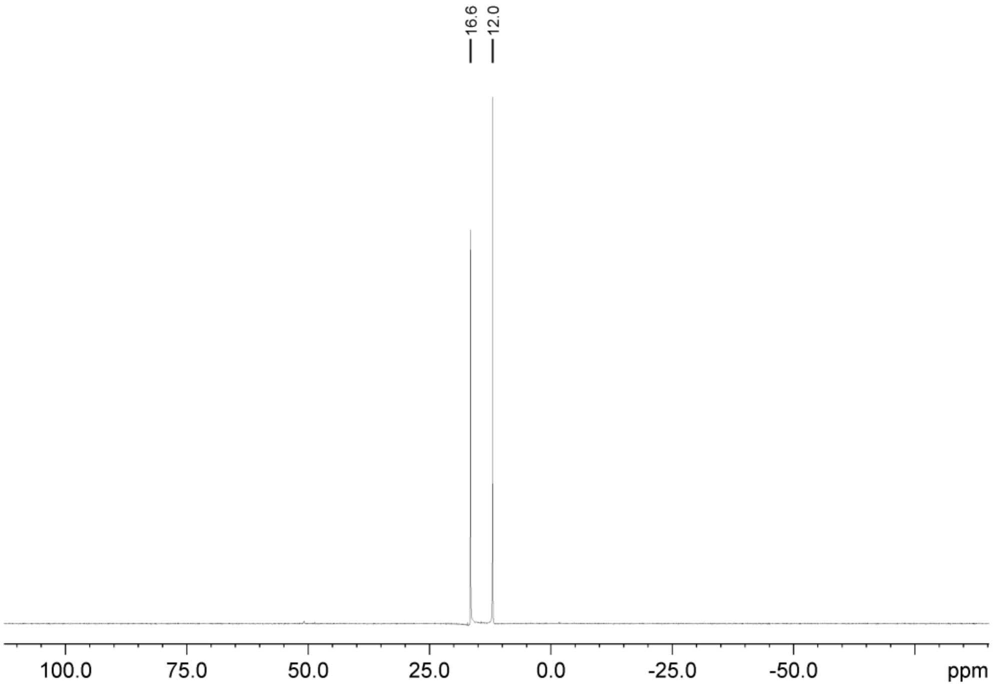

FIG. S61. $^{31}P\{^1H\}$ NMR (C_6D_6) SPECTRUM OF **2C**

NMR spectra of 6c

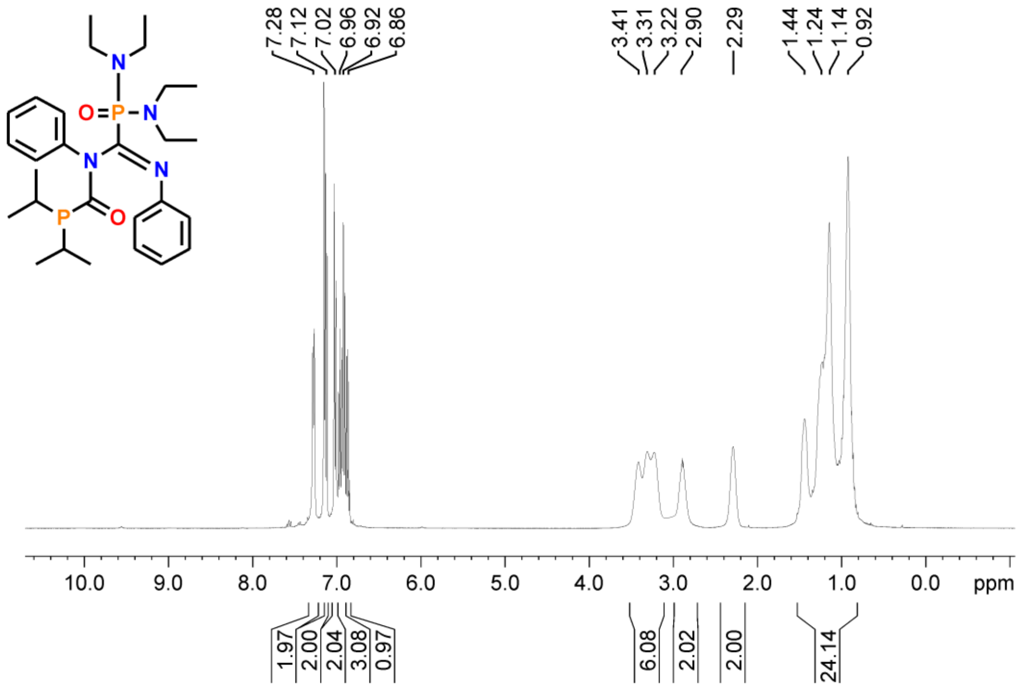

FIG. S62. ^1H NMR (C_6D_6) SPECTRUM OF **6C**

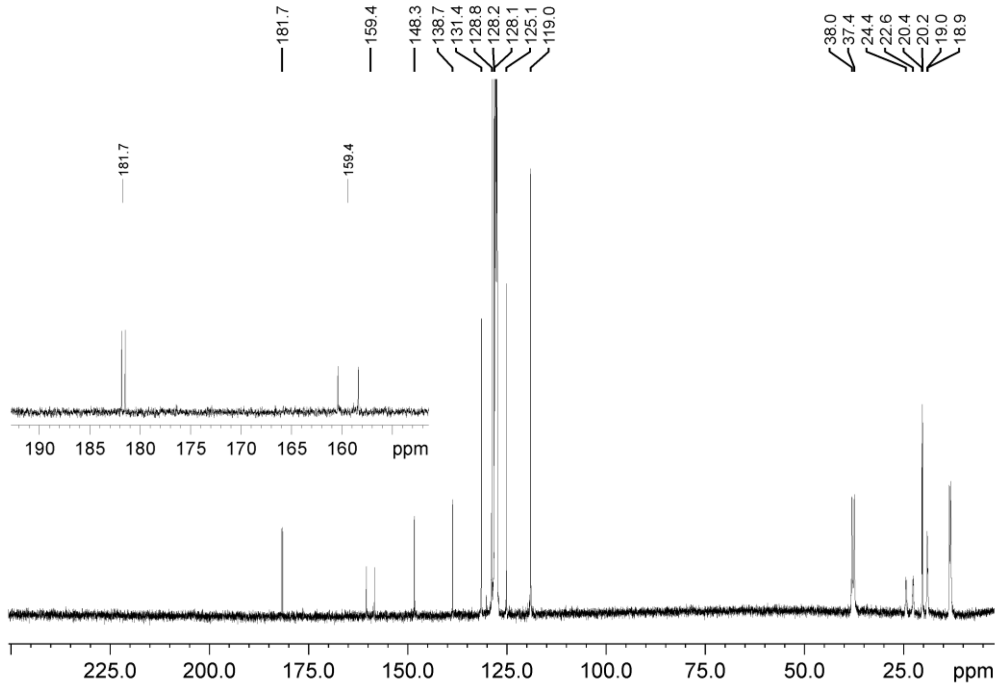

FIG. S63. $^{13}C\{^{1}H\}$ NMR (C_6D_6) SPECTRUM OF **6C**

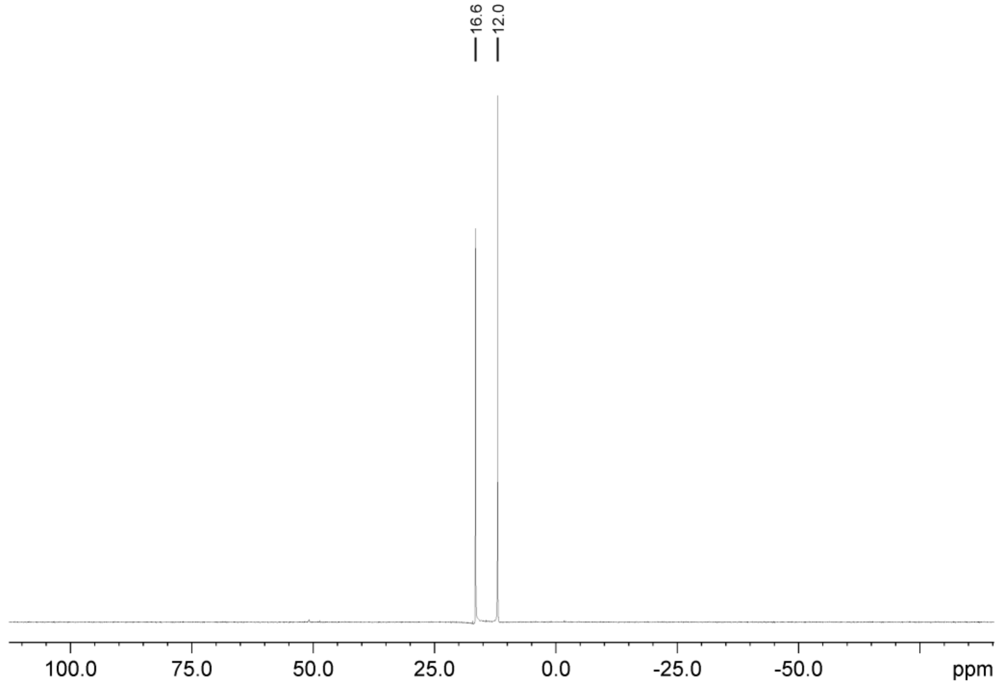

FIG. S64. $^{31}P\{^{1}H\}$ NMR (C_6D_6) SPECTRUM OF **6C**

NMR spectra of 9c

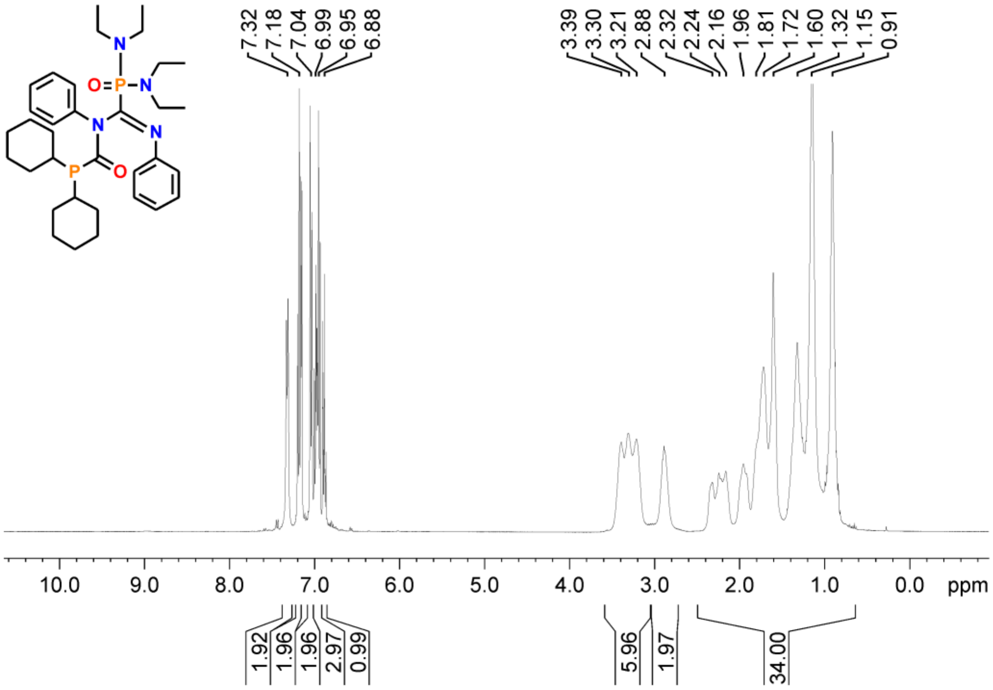

FIG. S65. ^{1}H NMR (C_6D_6) SPECTRUM OF **9C**

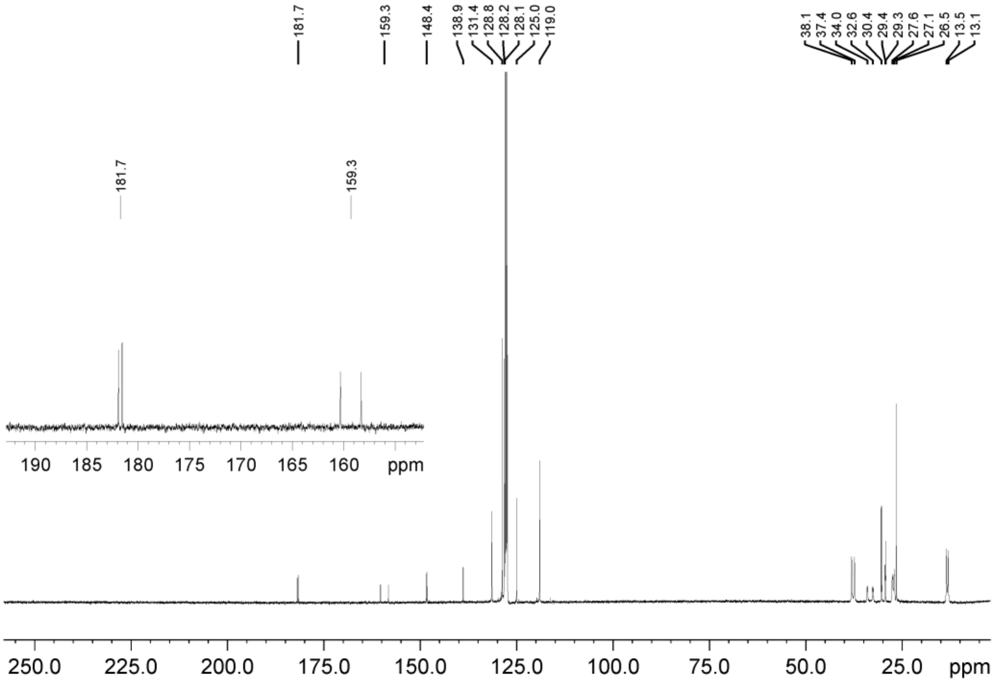

FIG. S66. $^{13}C\{^{1}H\}$ NMR (C_6D_6) SPECTRUM OF **9C**

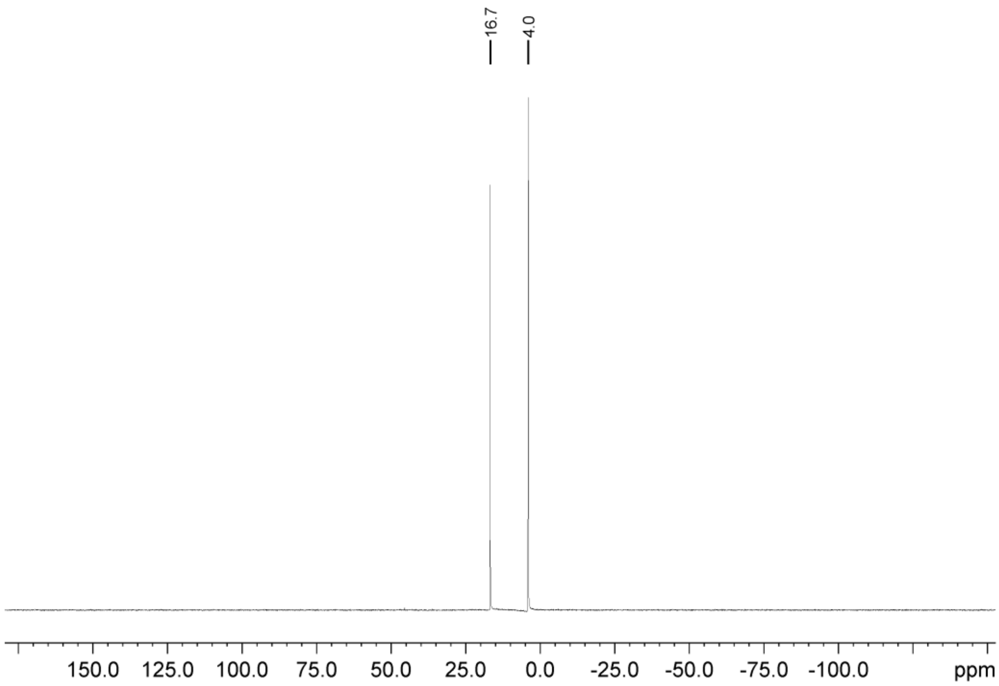

FIG. S67. $^{31}P\{^{1}H\}$ NMR (C_6D_6) SPECTRUM OF **9C**

NMR spectra of 1d and 1d’

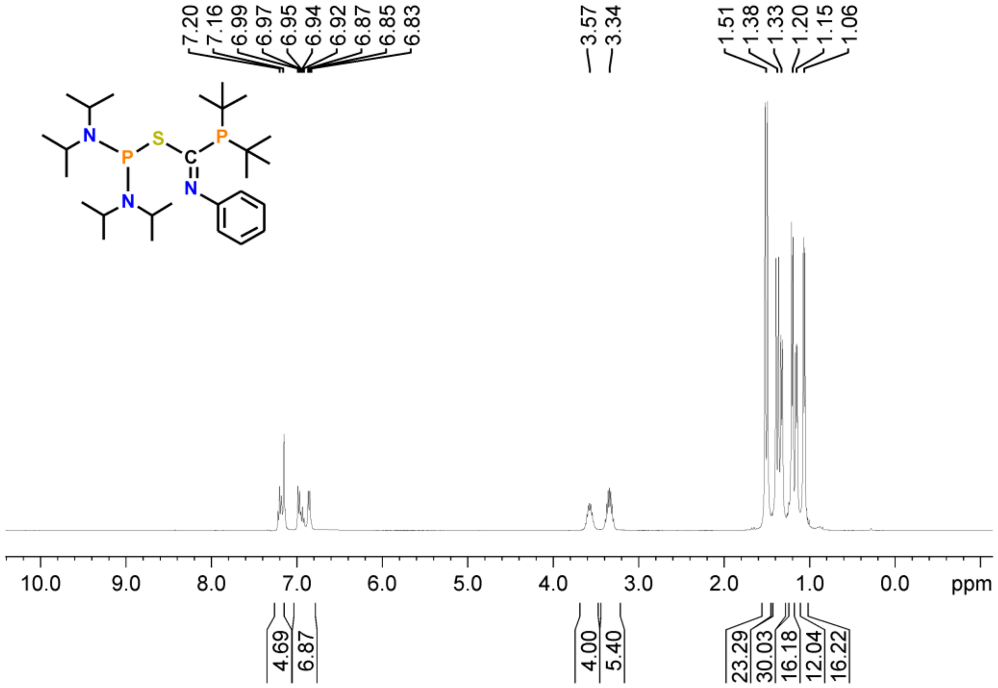

FIG. S68. ^{1}H NMR (C_6D_6) SPECTRUM OF **1D** AND **1D’**

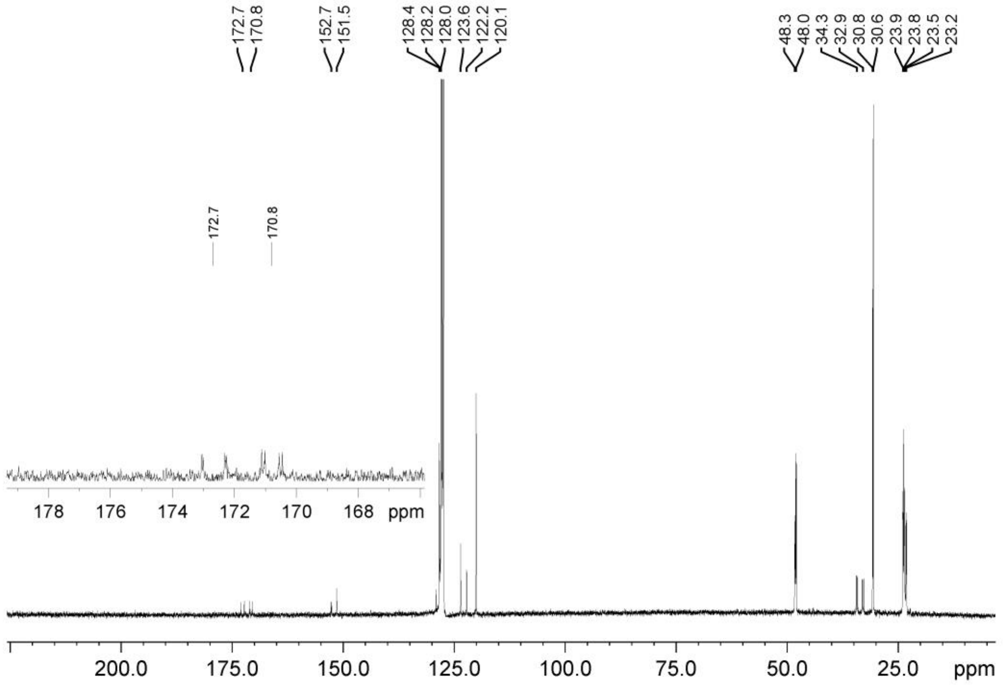

FIG. S69. $^{13}C\{^{1}H\}$ NMR (C_6D_6) SPECTRUM OF **1D** AND **1D'**

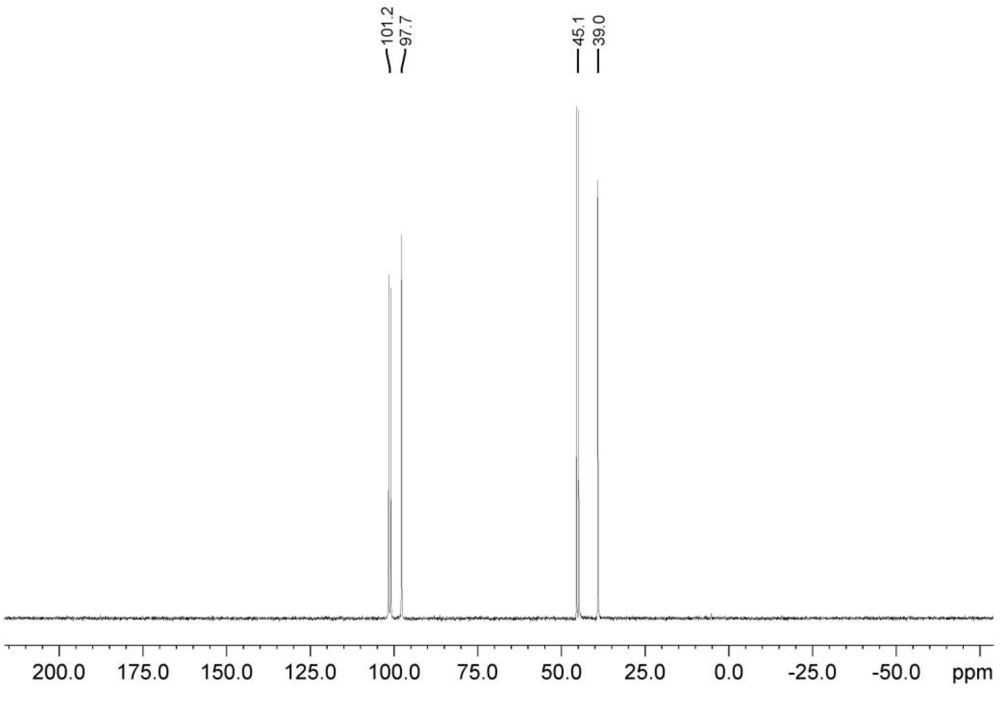

FIG. S70. $^{31}P\{^{1}H\}$ NMR (C_6D_6) SPECTRUM OF **1D** AND **1D'**

NMR spectra of 2d

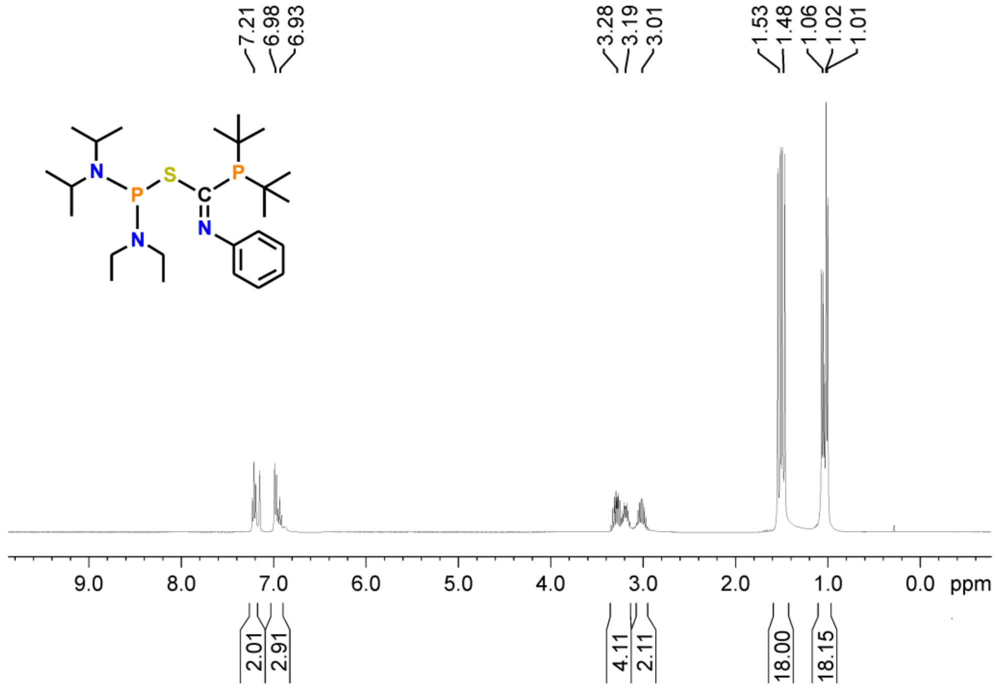

FIG. S71. ^{1}H NMR (C_6D_6) SPECTRUM OF **2D**

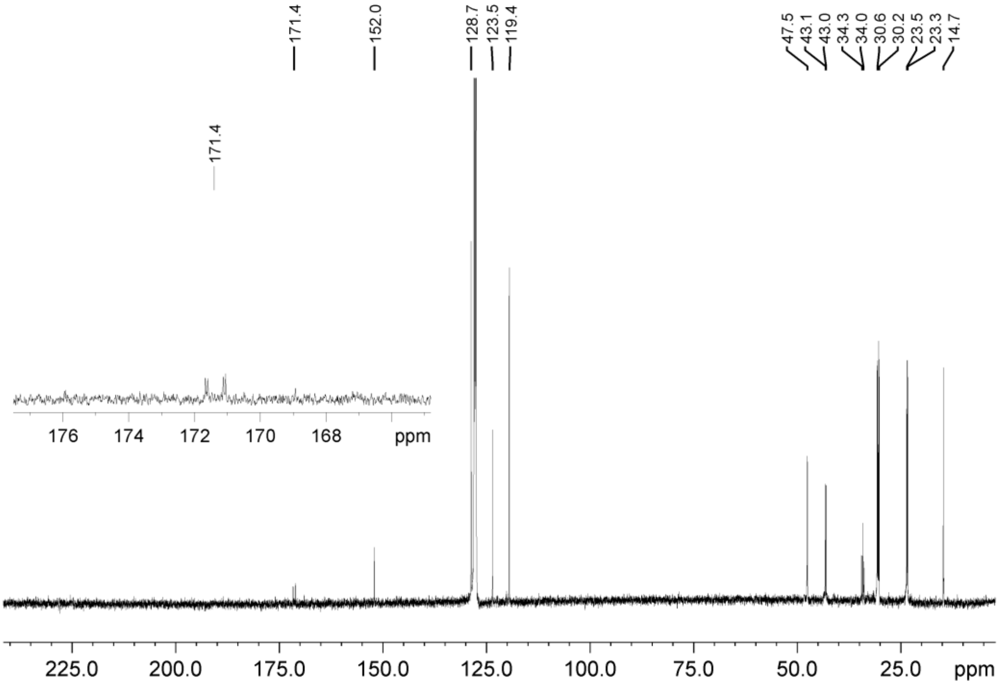

FIG. S72. ^{13}C{^{1}H} NMR (C_6D_6) SPECTRUM OF **2D**

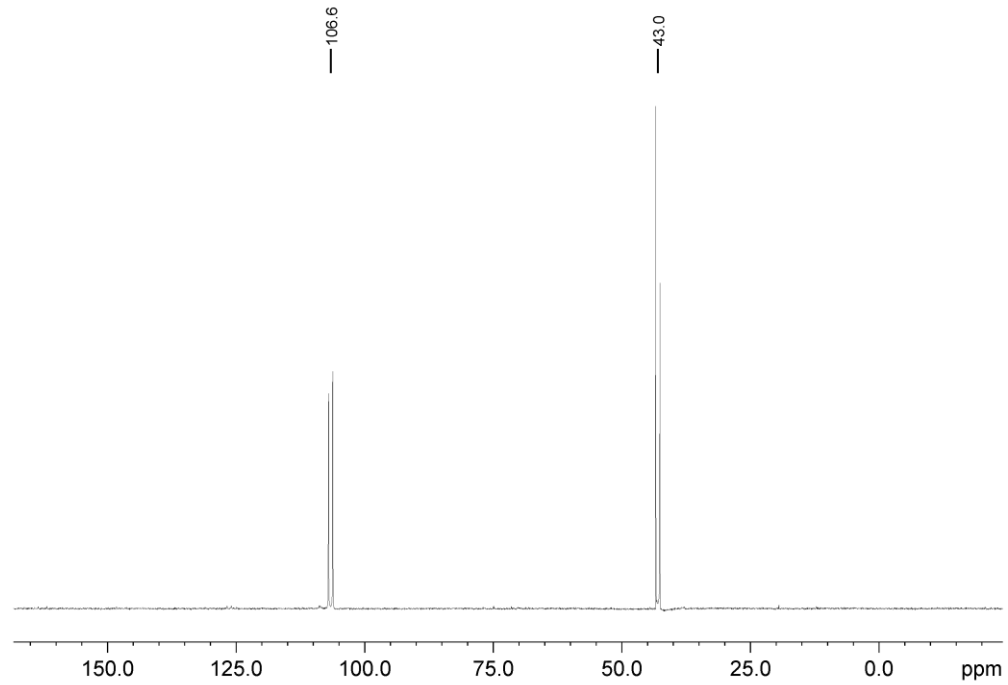

FIG. S73. $^{31}P\{^{1}H\}$ NMR (C_6D_6) SPECTRUM OF **2D**

NMR spectra of 3d and 3d’

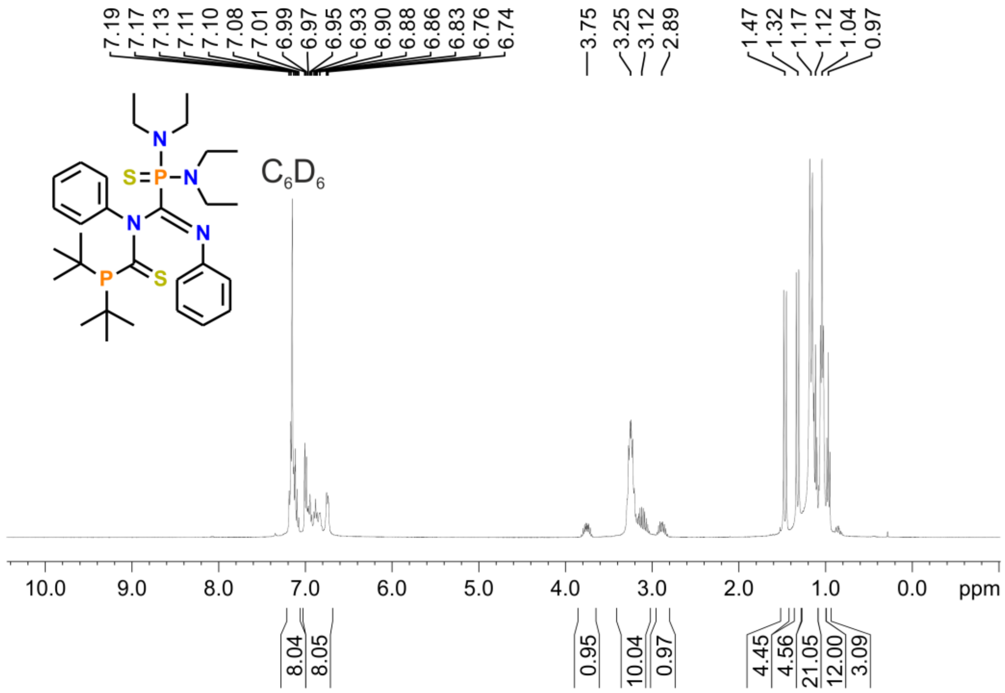

FIG. S74. ^{1}H NMR (C_6D_6) SPECTRUM OF **3D** AND **3D’**

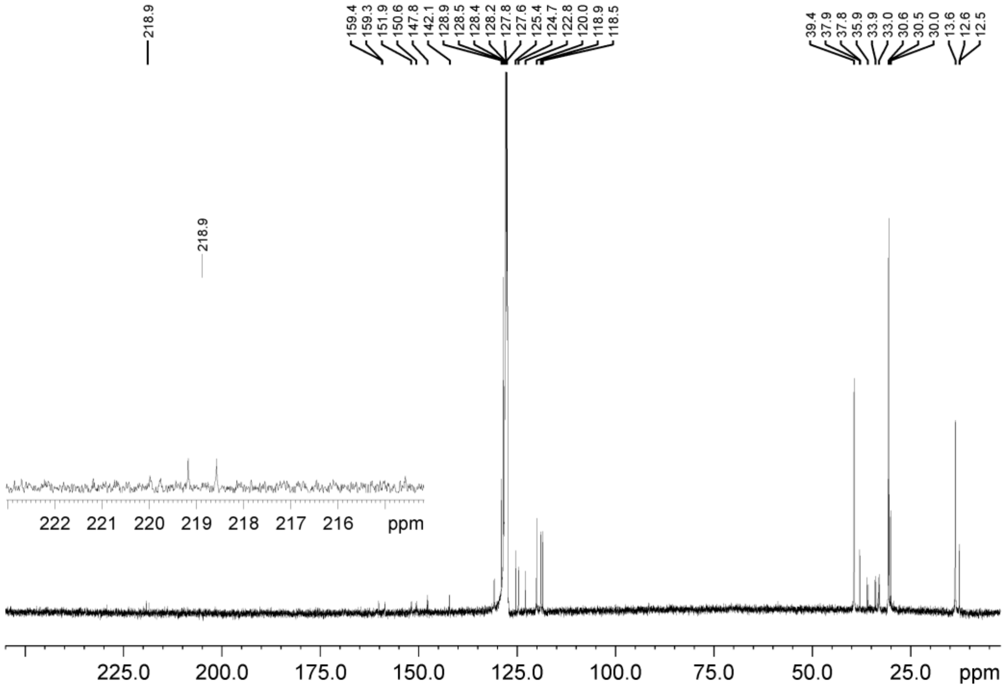

FIG. S75. $^{13}C\{^{1}H\}$ NMR (C_6D_6) SPECTRUM OF **3D** AND **3D'**

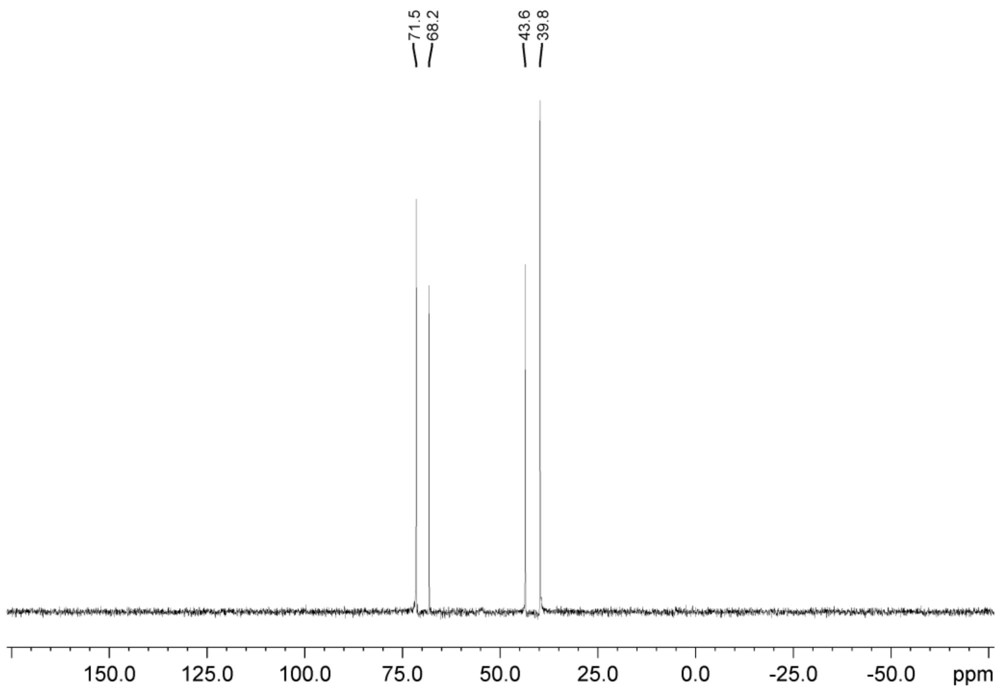

FIG. S76. $^{31}P\{^{1}H\}$ NMR (C_6D_6) SPECTRUM OF **3D** AND **3D'**

NMR spectra of 3d*

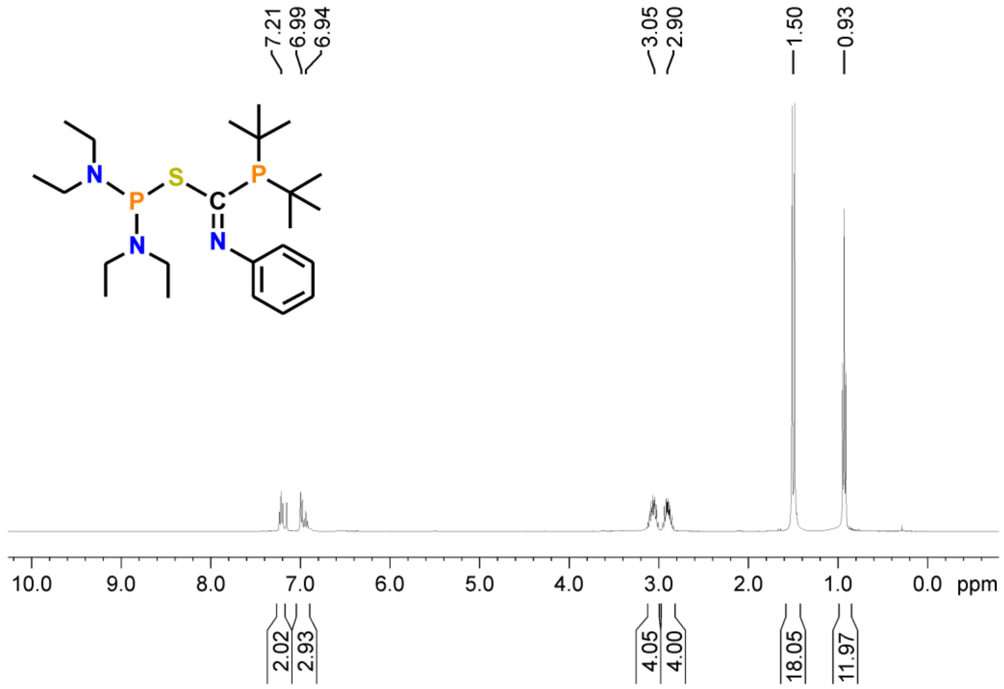

FIG. S77. ^{1}H NMR (C_6D_6) SPECTRUM OF **3D***

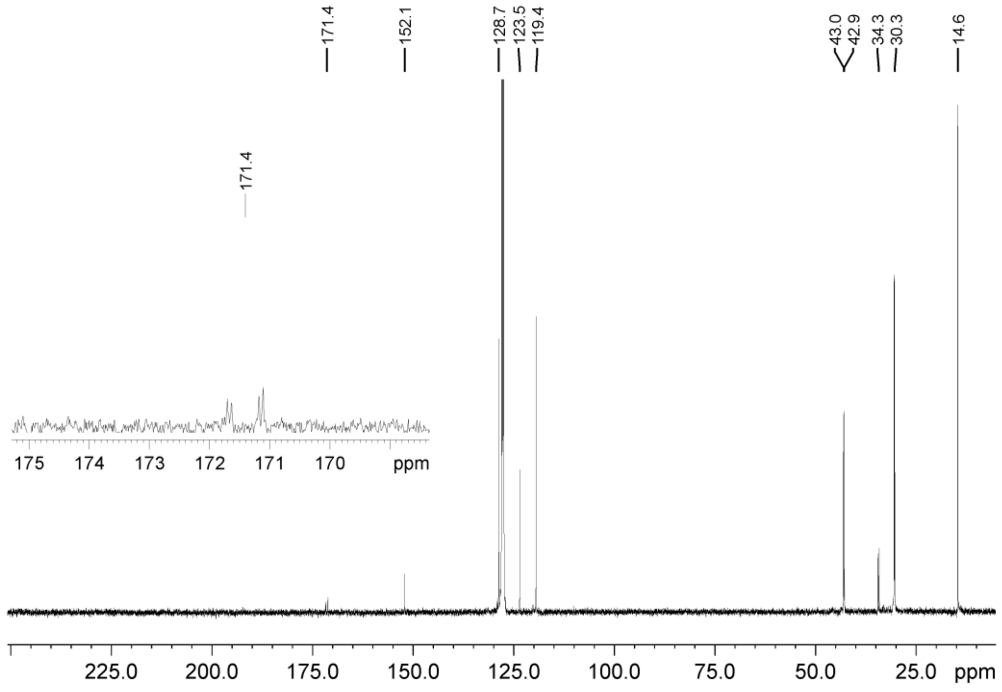

FIG. S78. $^{13}C\{^{1}H\}$ NMR (C_6D_6) SPECTRUM OF **3D***

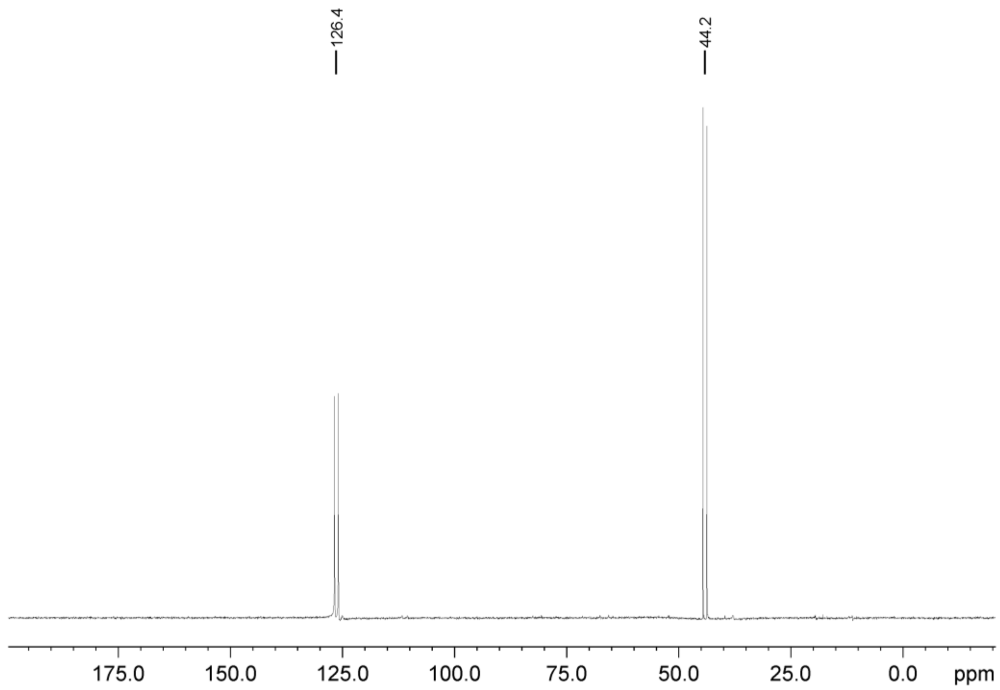

FIG. S79. $^{31}P\{^{1}H\}$ NMR (C_6D_6) SPECTRUM OF **3D***

NMR spectra of 4d and 4d’

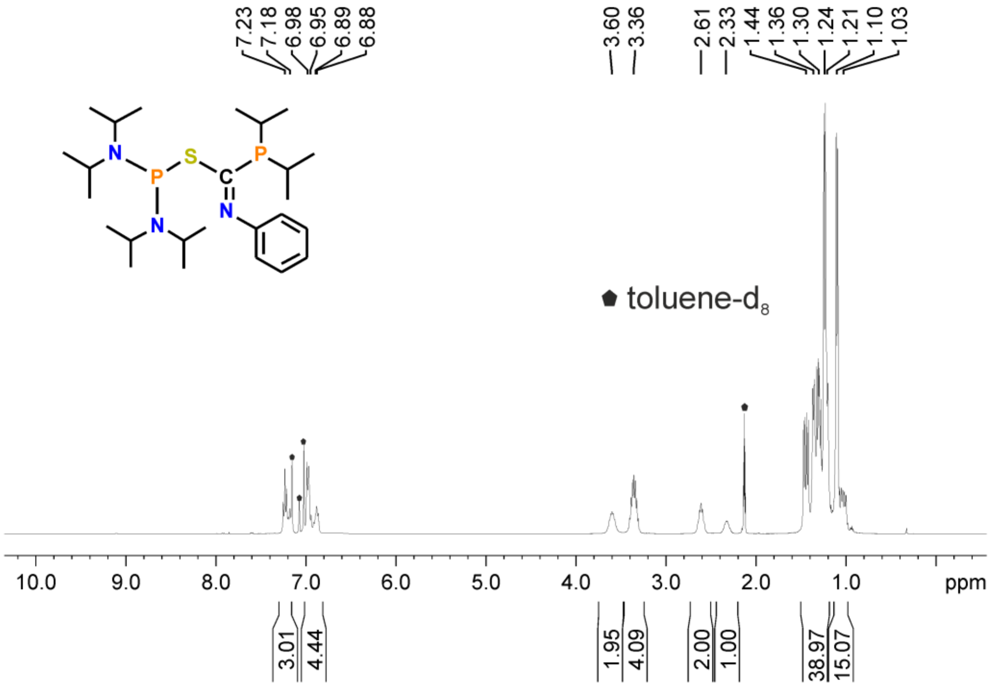

FIG. S80. ^{1}H NMR (TOLUENE-D_8, 273K) SPECTRUM OF **4D** AND **4D’**

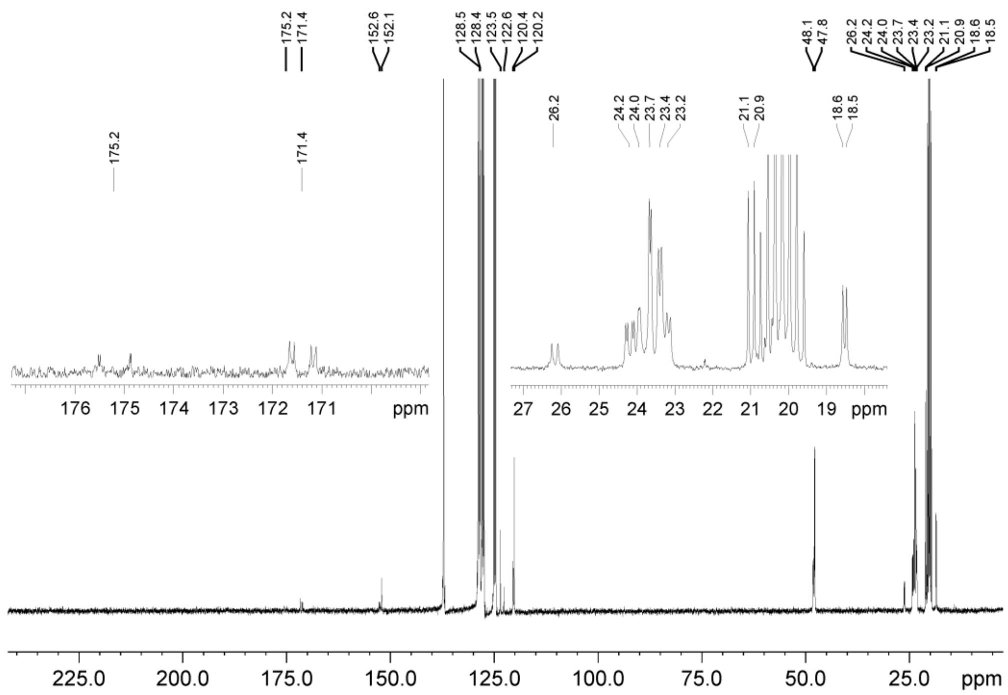

FIG. S81. $^{13}C\{^{1}H\}$ NMR (TOLUENE-D$_8$, 273K) SPECTRUM OF **4D** AND **4D'**

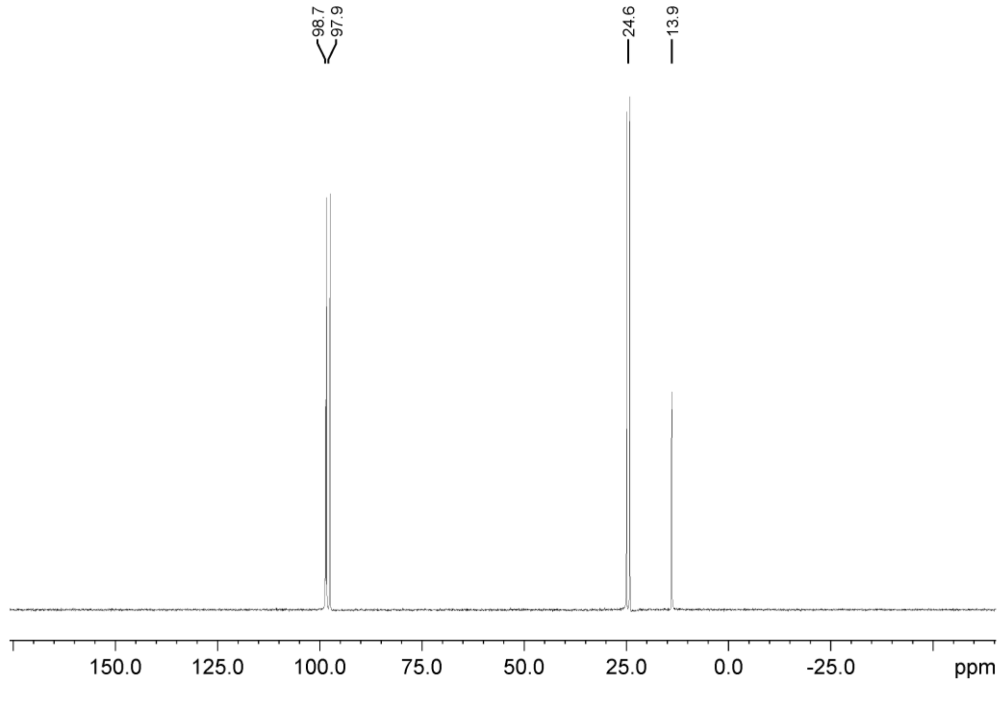

FIG. S82. $^{31}P\{^{1}H\}$ NMR (TOLUENE-D$_8$, 273K) SPECTRUM OF **4D** AND **4D'**

NMR spectra of 5d

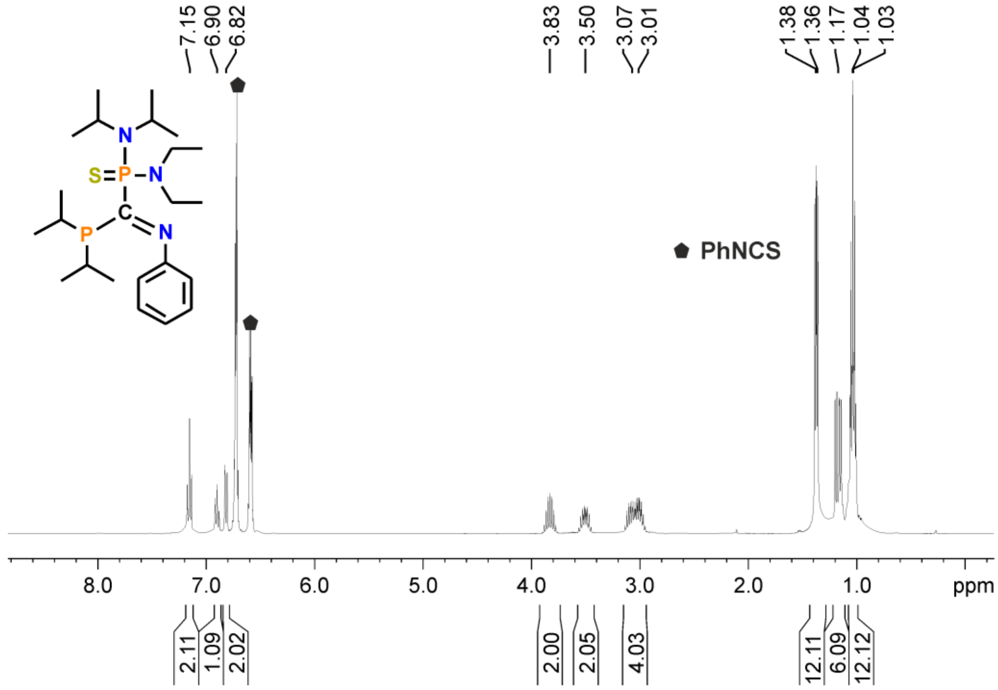

FIG. S83. ^{1}H NMR (C_6D_6) SPECTRUM OF **5D**

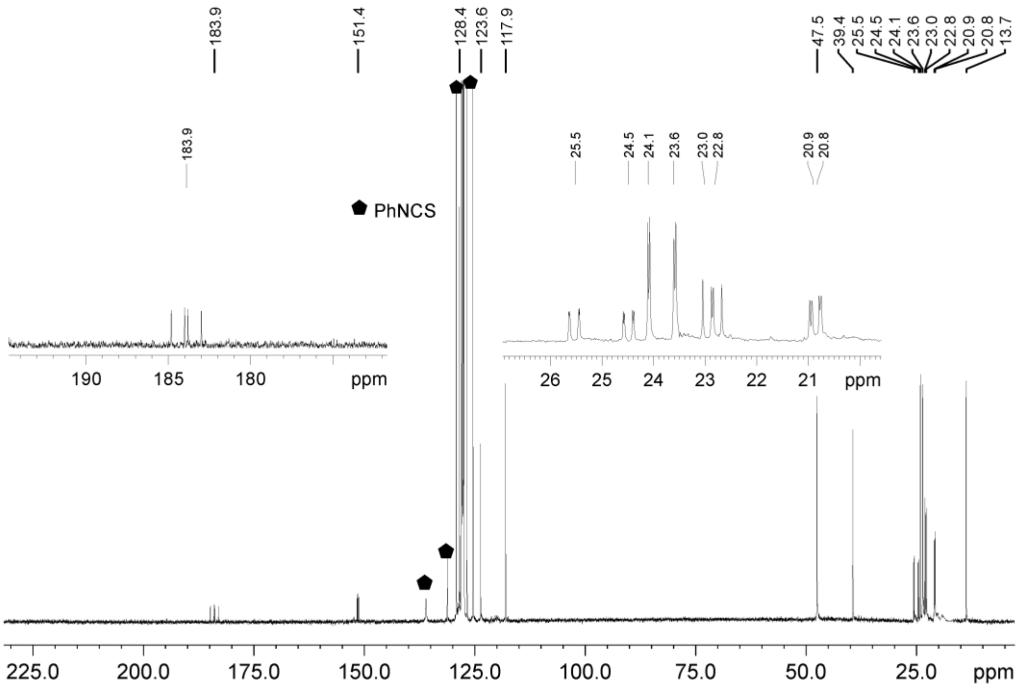

FIG. S84. ^{13}C{^{1}H} NMR (C_6D_6) SPECTRUM OF **5D**

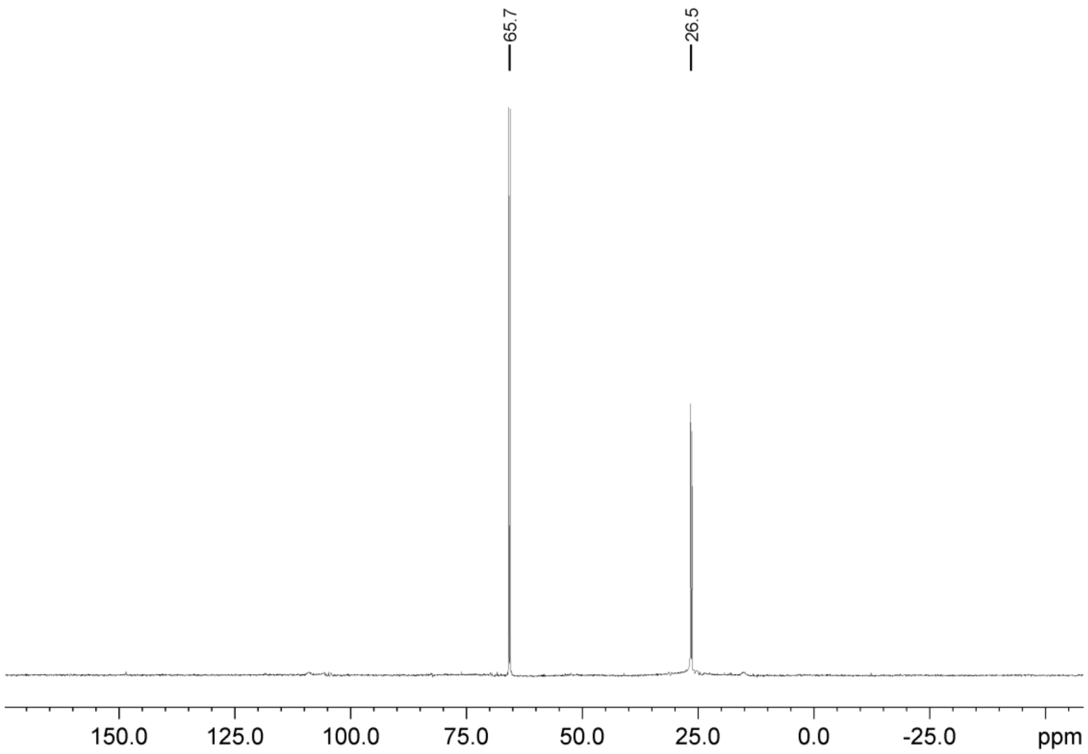

FIG. S85. $^{31}P\{^{1}H\}$ NMR (C_6D_6) SPECTRUM OF **5D**

NMR spectra of 6d

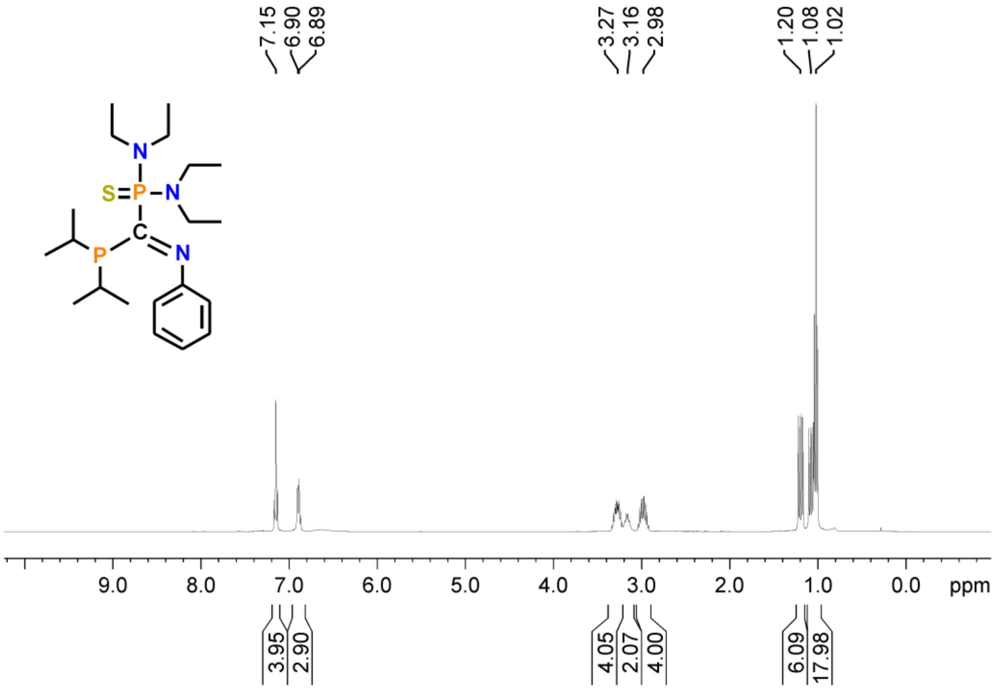

FIG. S86. ^{1}H NMR (C_6D_6) SPECTRUM OF **6D**

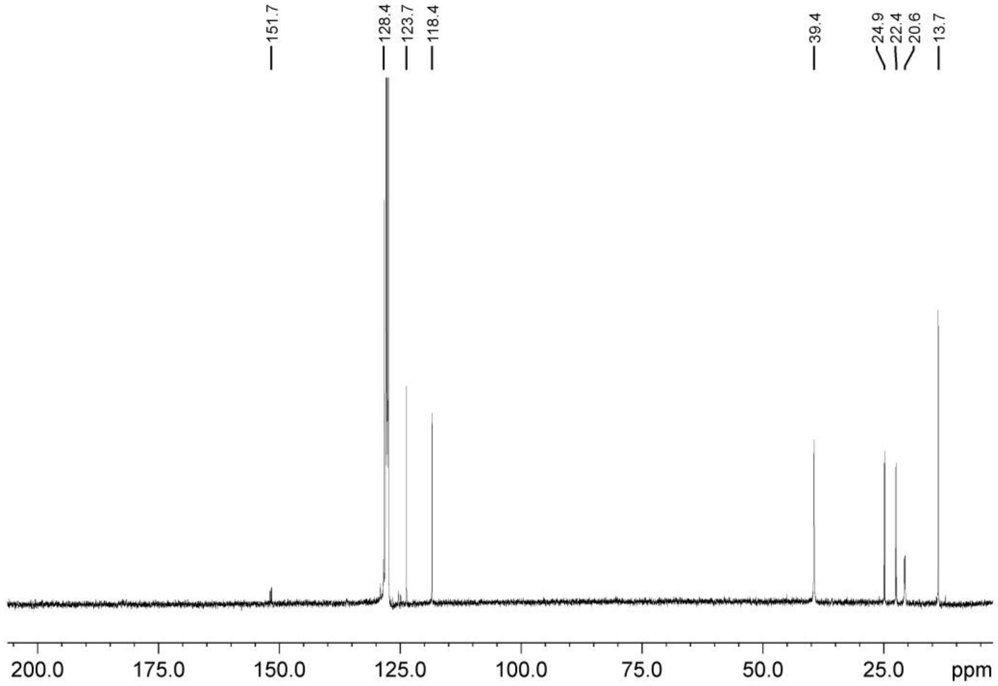

FIG. S87. $^{13}C\{^{1}H\}$ NMR (C_6D_6) SPECTRUM OF **6D**

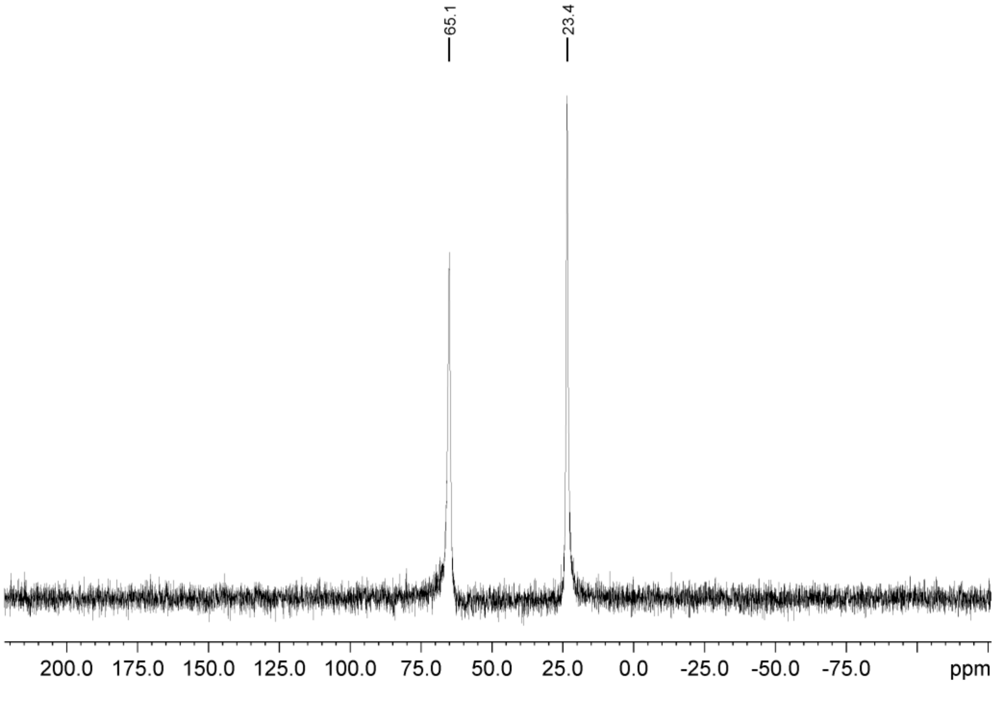

FIG. S88. $^{31}P\{^{1}H\}$ NMR (C_6D_6) SPECTRUM OF **6D**

NMR spectra of 7d and **7d’**

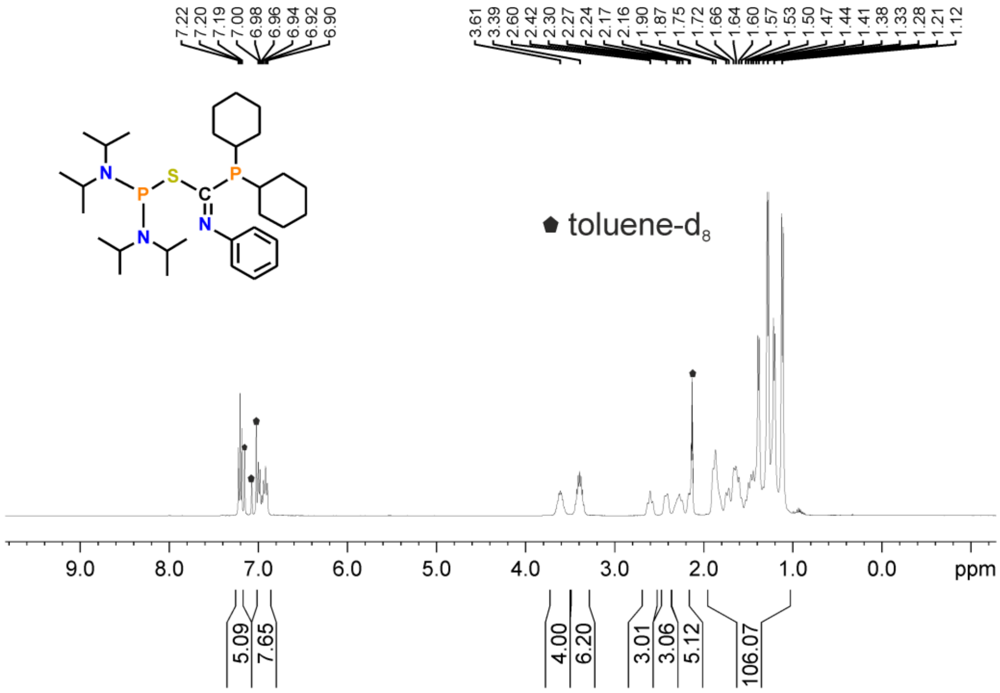

FIG. S89. ^{1}H NMR (TOLUENE-D$_8$, 273K) SPECTRUM OF **7D** AND **7D’**

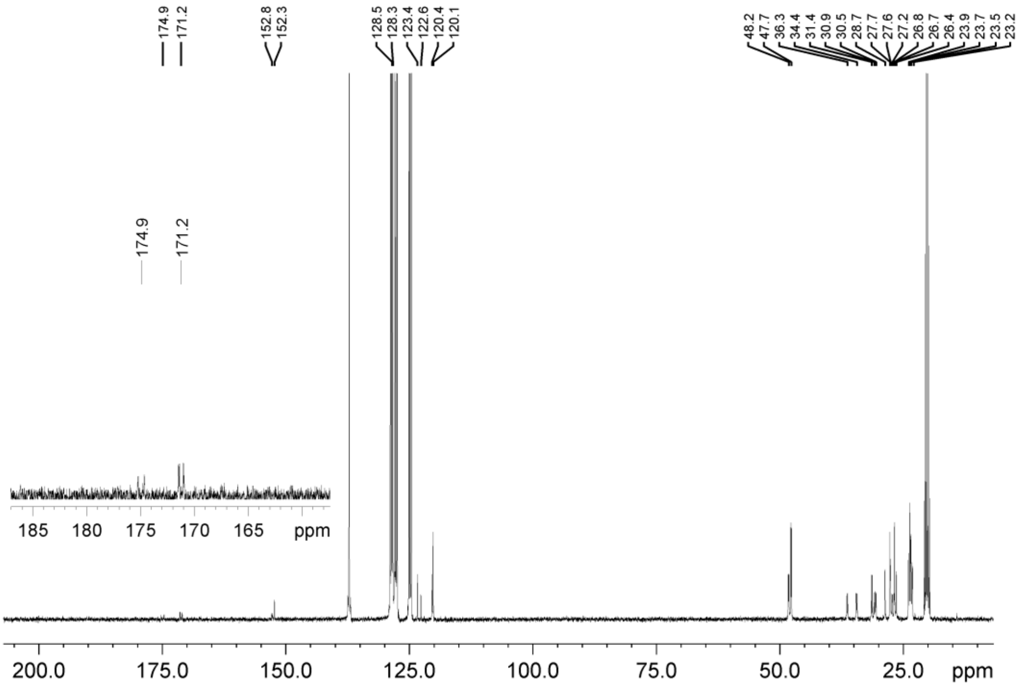

FIG. S90. ^{13}C{^{1}H} NMR (TOLUENE-D$_8$, 273K) SPECTRUM OF **7D** AND **7D’**

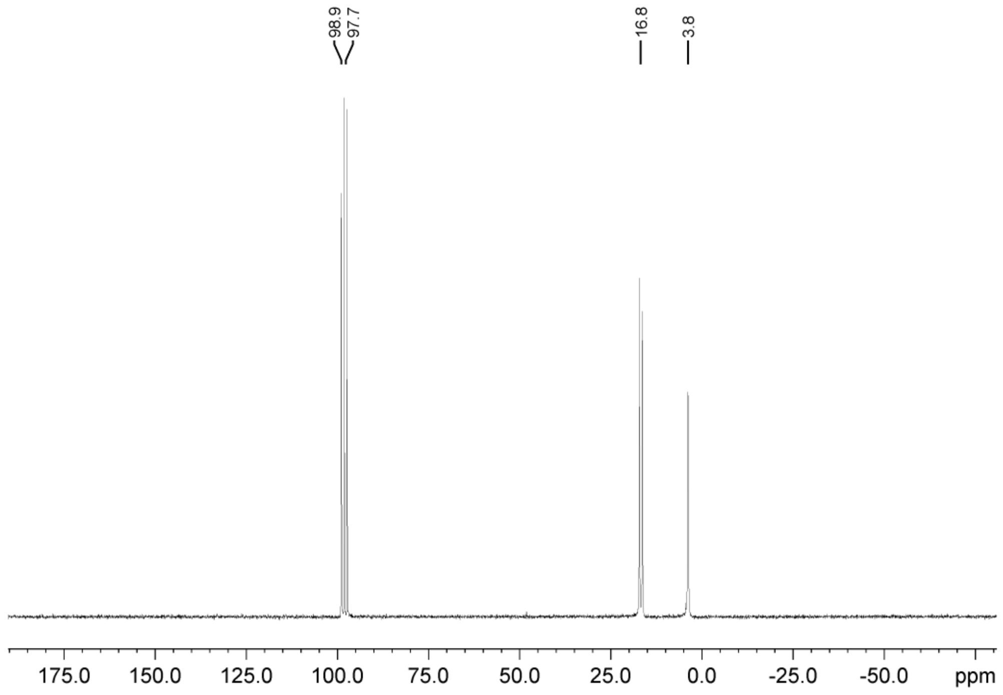

FIG. S91. $^{31}P\{^{1}H\}$ NMR (TOLUENE-D_8, 273K) SPECTRUM OF **7D** AND **7D'**

NMR spectra of 8d

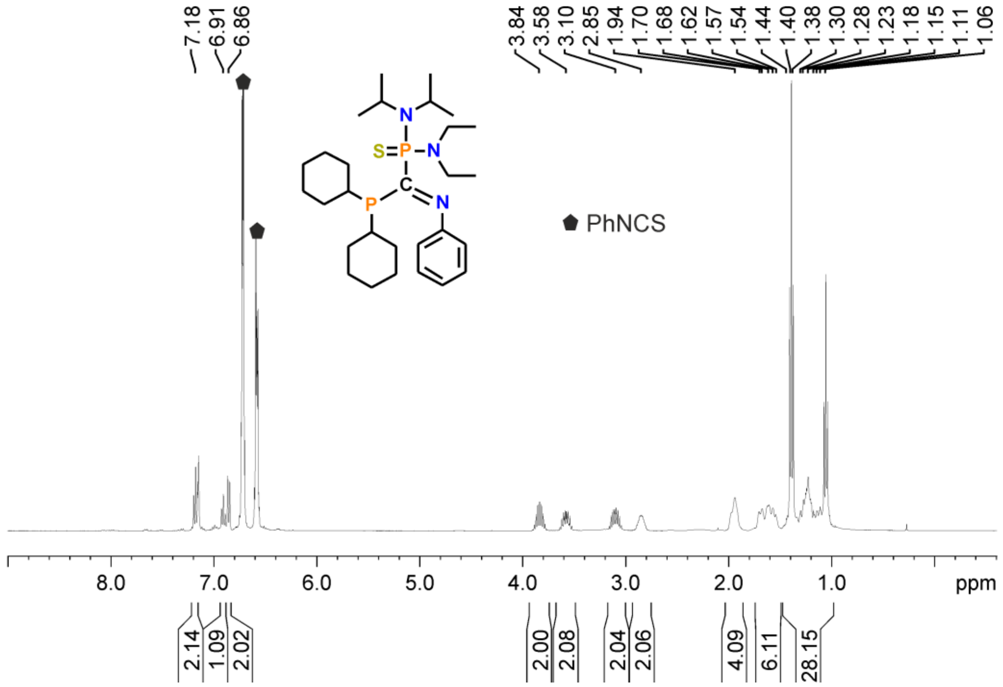

FIG. S92. ^{1}H NMR (C_6D_6) SPECTRUM OF **8D**

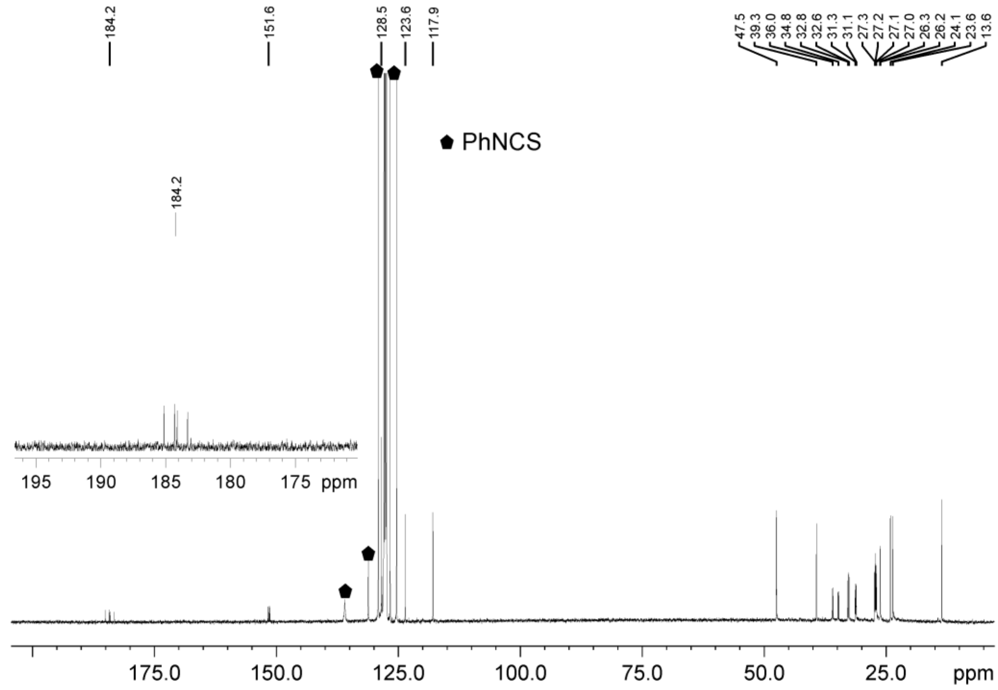

FIG. S93. $^{13}C\{^{1}H\}$ NMR (C_6D_6) SPECTRUM OF **8D**

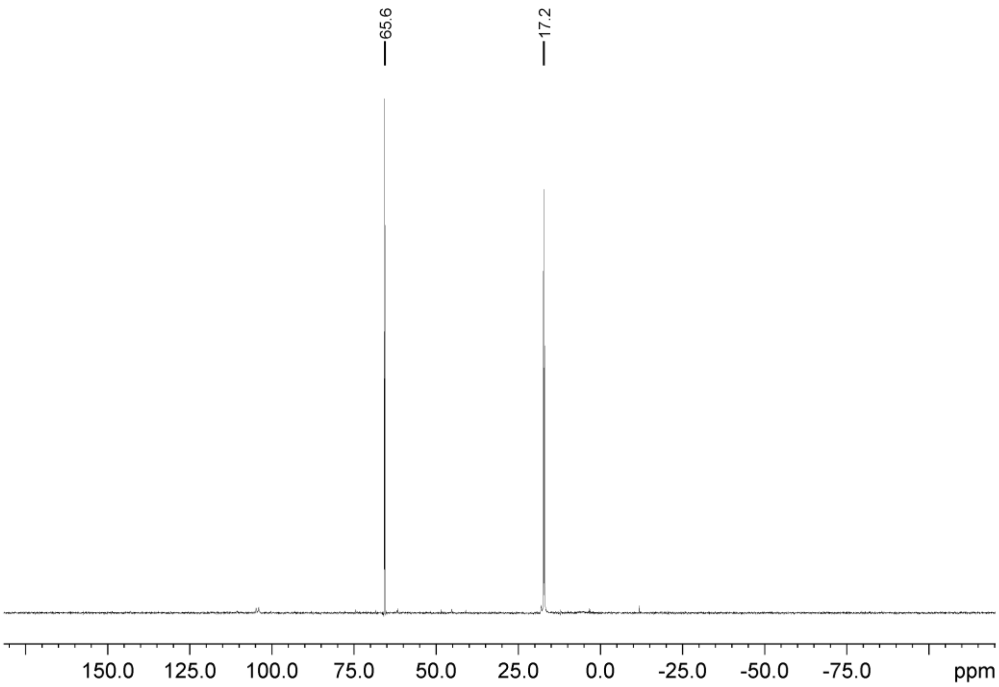

FIG. S94. $^{31}P\{^{1}H\}$ NMR (C_6D_6) SPECTRUM OF **8D**

NMR spectra of 9d

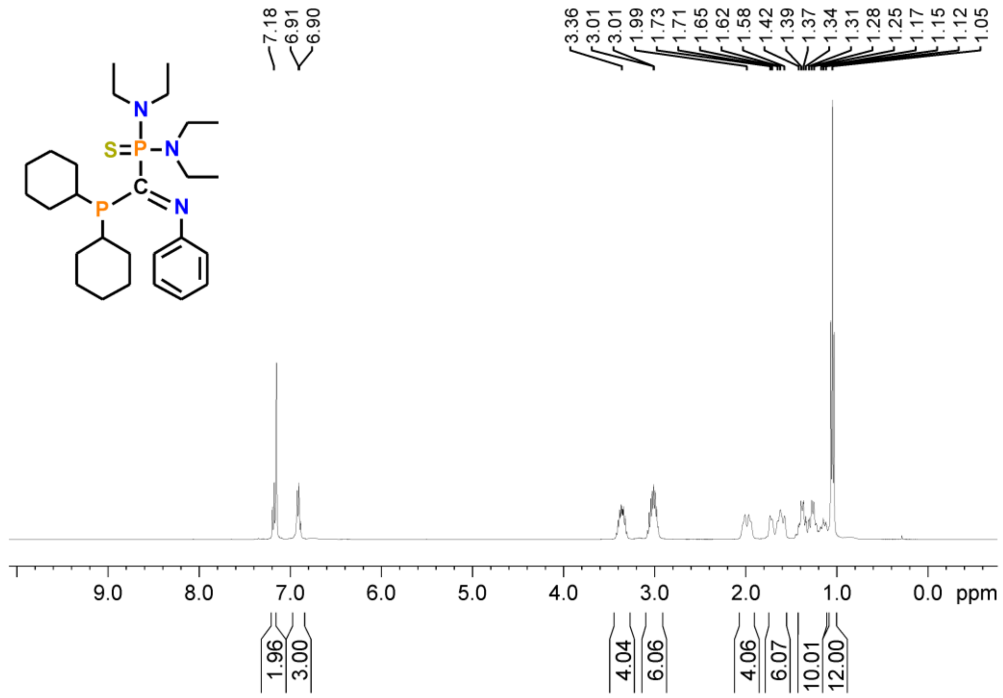

FIG. S95. ^{1}H NMR (C_6D_6) SPECTRUM OF **9D**

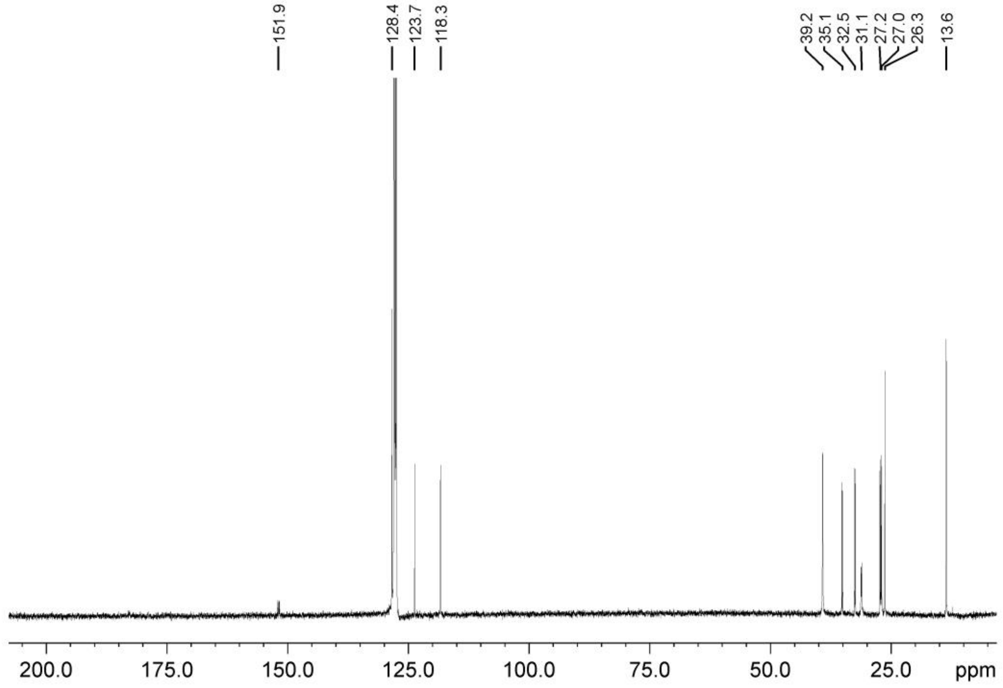

FIG. S96. $^{13}C\{^{1}H\}$ NMR (C_6D_6) SPECTRUM OF **9D**

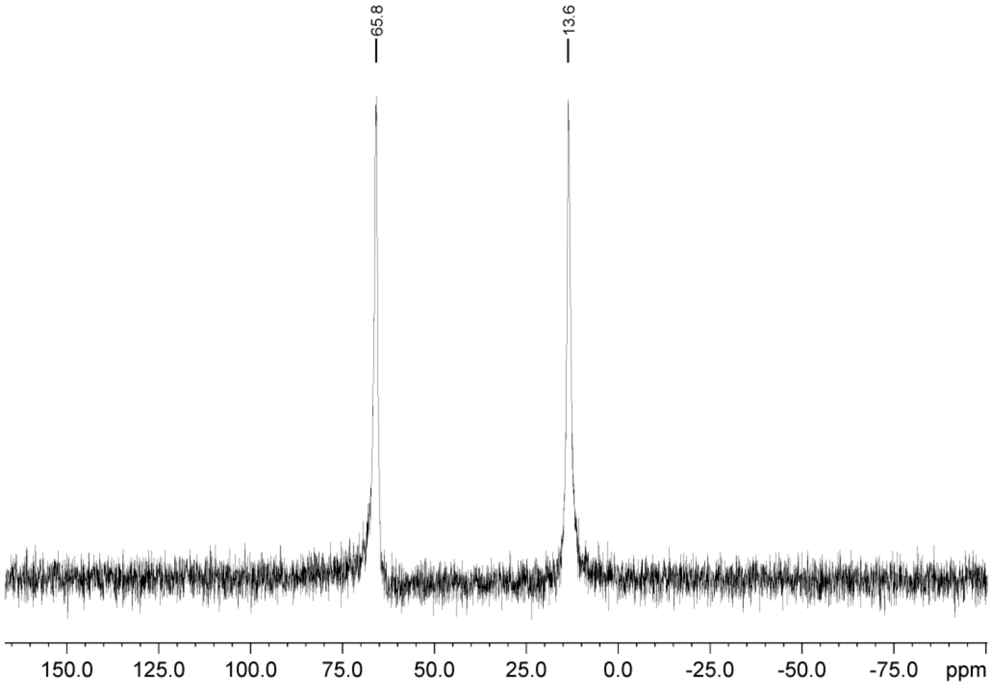

FIG. S97. $^{31}P\{^{1}H\}$ NMR (C_6D_6) SPECTRUM OF **9D**

NMR spectra of 10d

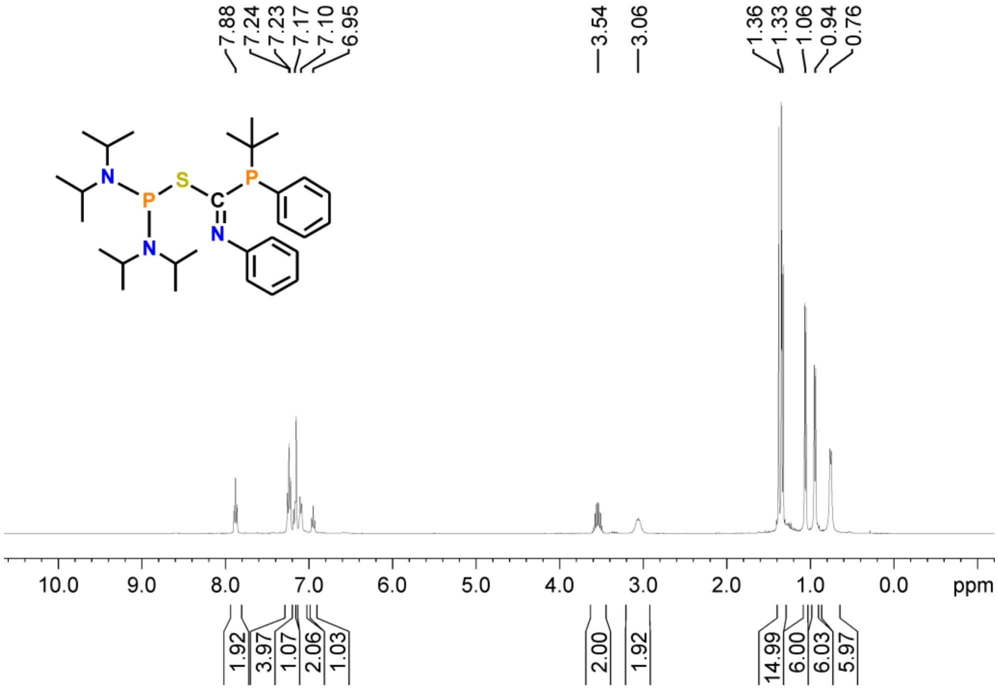

FIG. S98. ^{1}H NMR (C_6D_6) SPECTRUM OF **10D**

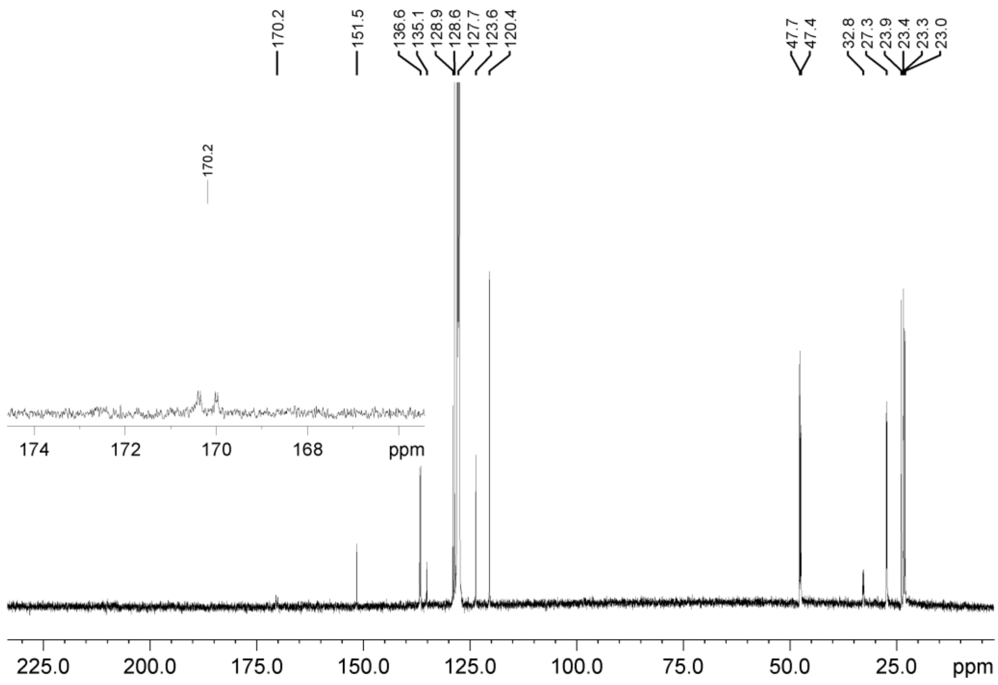

FIG. S99. $^{13}C\{^1H\}$ NMR (C_6D_6) SPECTRUM OF **10D**

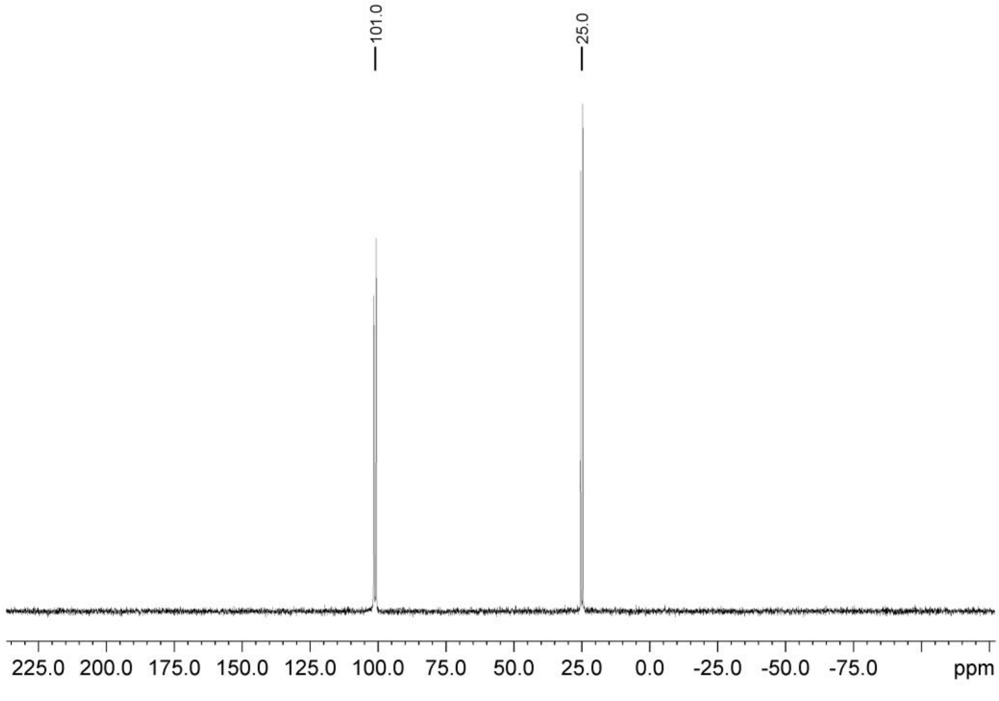

FIG. S100. $^{31}P\{^1H\}$ NMR (C_6D_6) SPECTRUM OF **10D**

IR spectra of isolated compounds

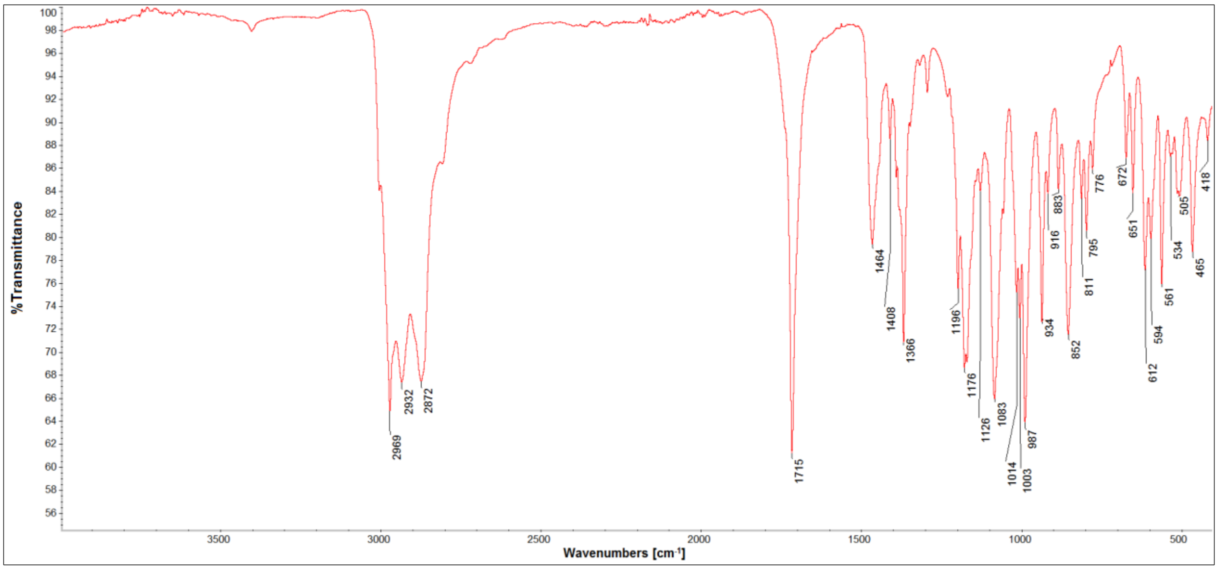

FIG. S101. IR SPECTRUM OF SOLID $\mathbf{2A}_{\mathbf{Pt}}$

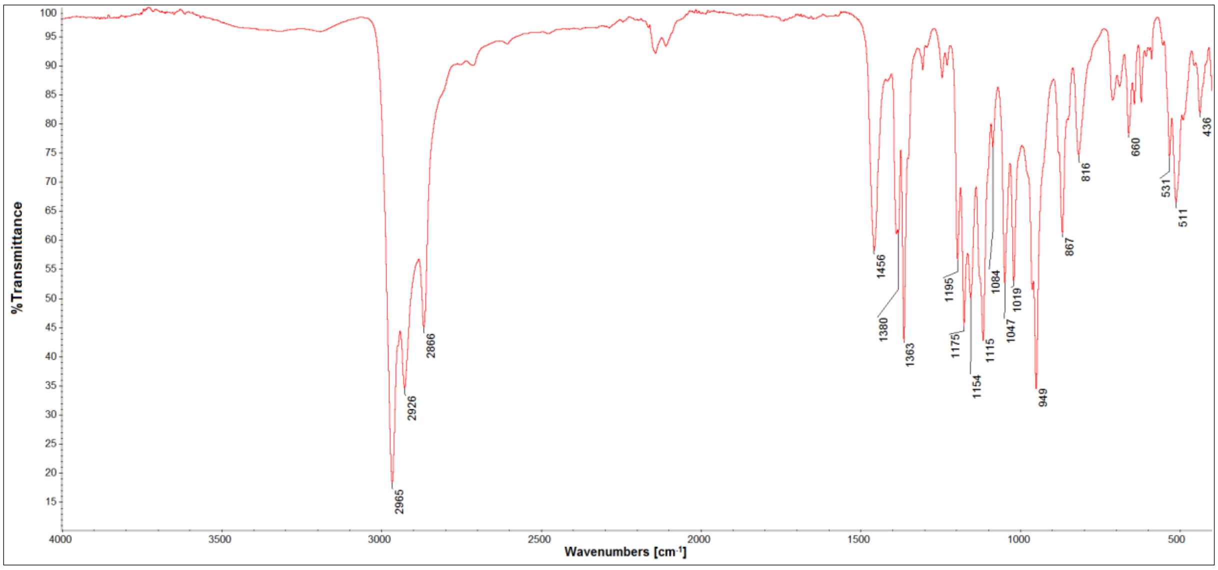

FIG. S102. IR SPECTRUM OF SOLID **4B**

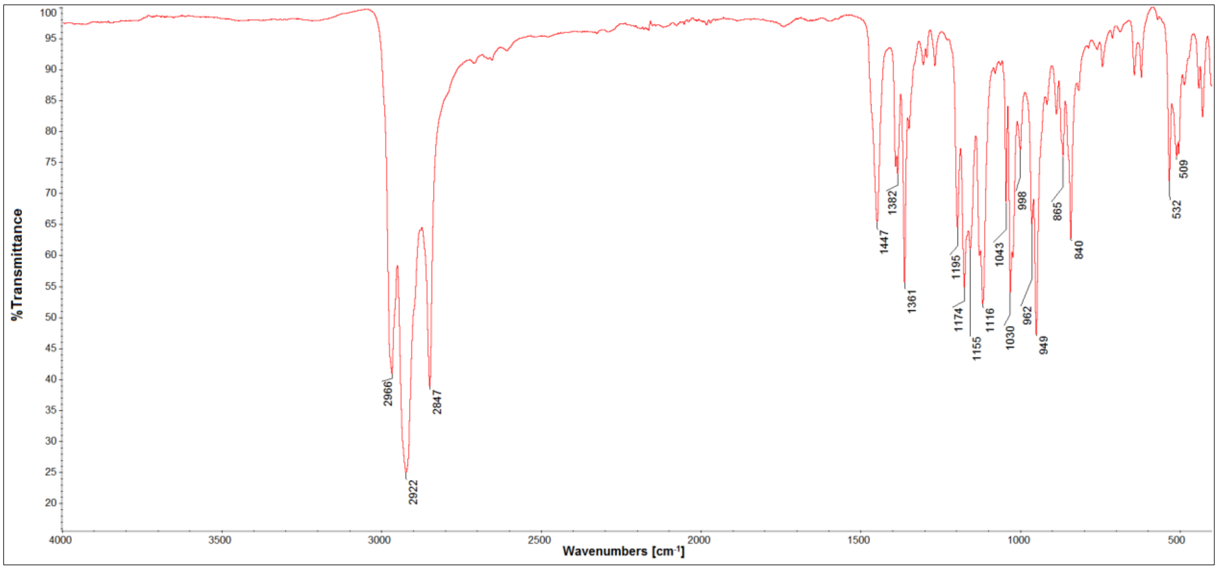

FIG. S103. IR SPECTRUM OF OIL **7B**

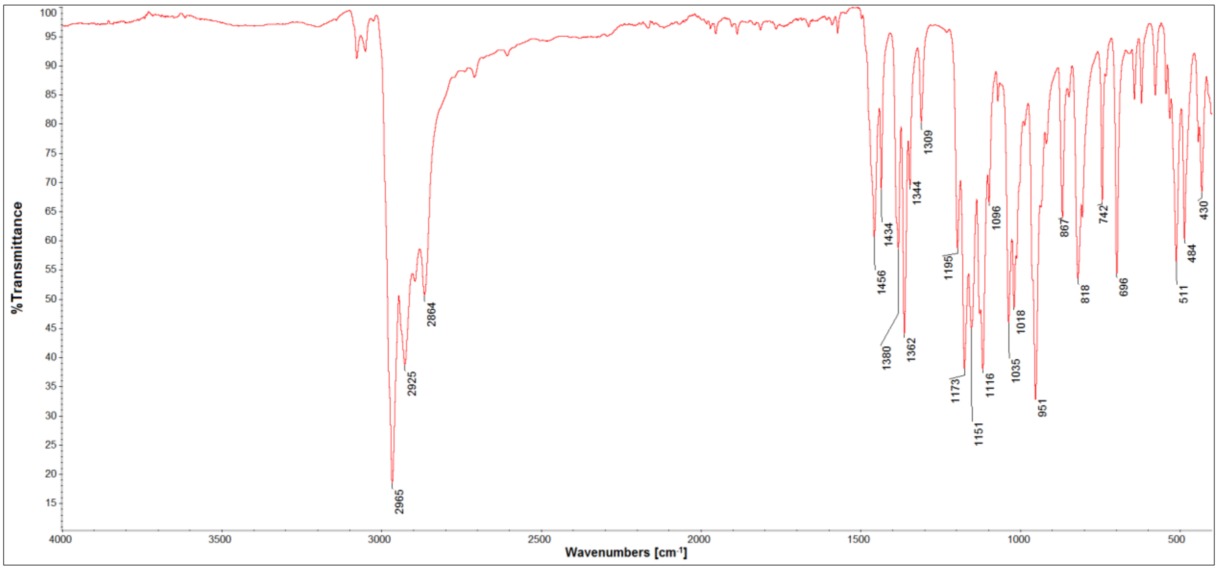

FIG. S104. IR SPECTRUM OF SOLID **10B**

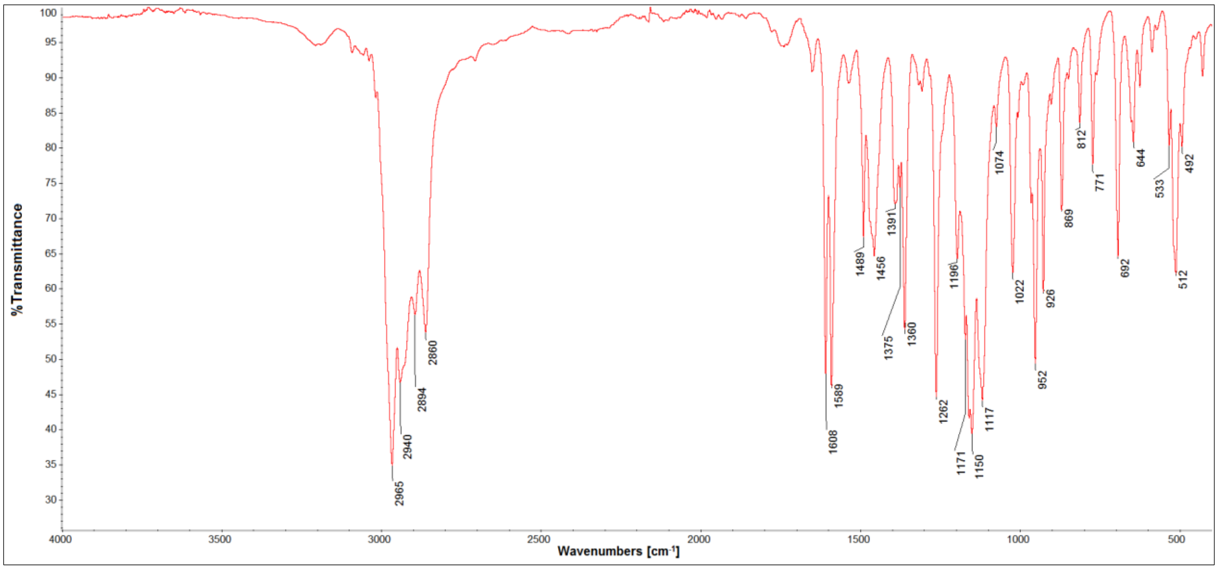

FIG. S105. IR SPECTRUM OF SOLID **1C/1C'**

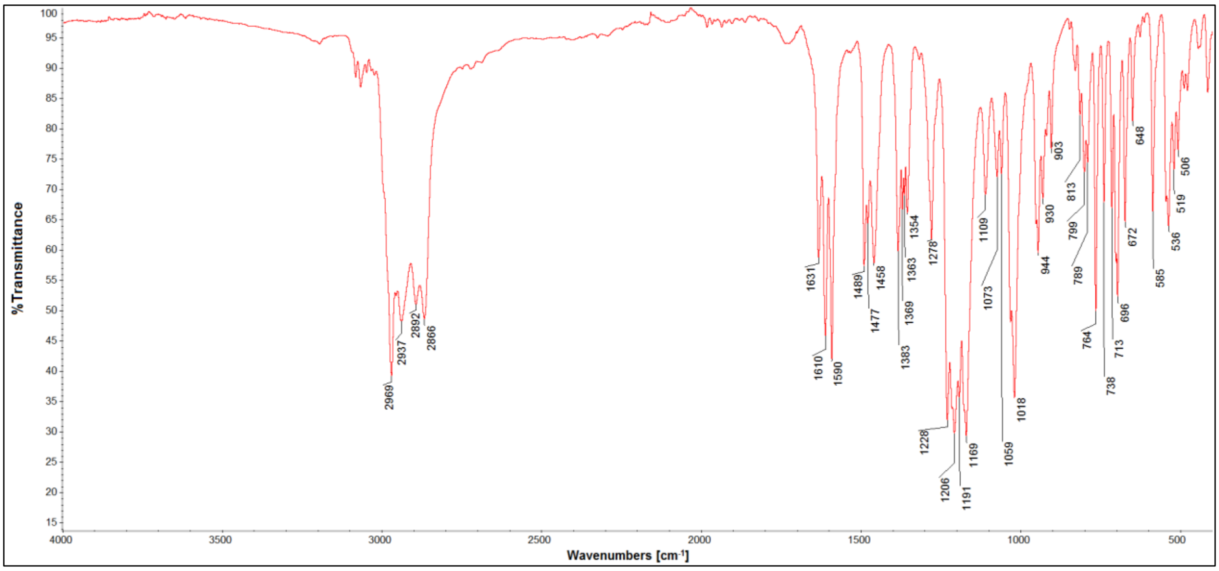

FIG. S106. IR SPECTRUM OF SOLID **3C**

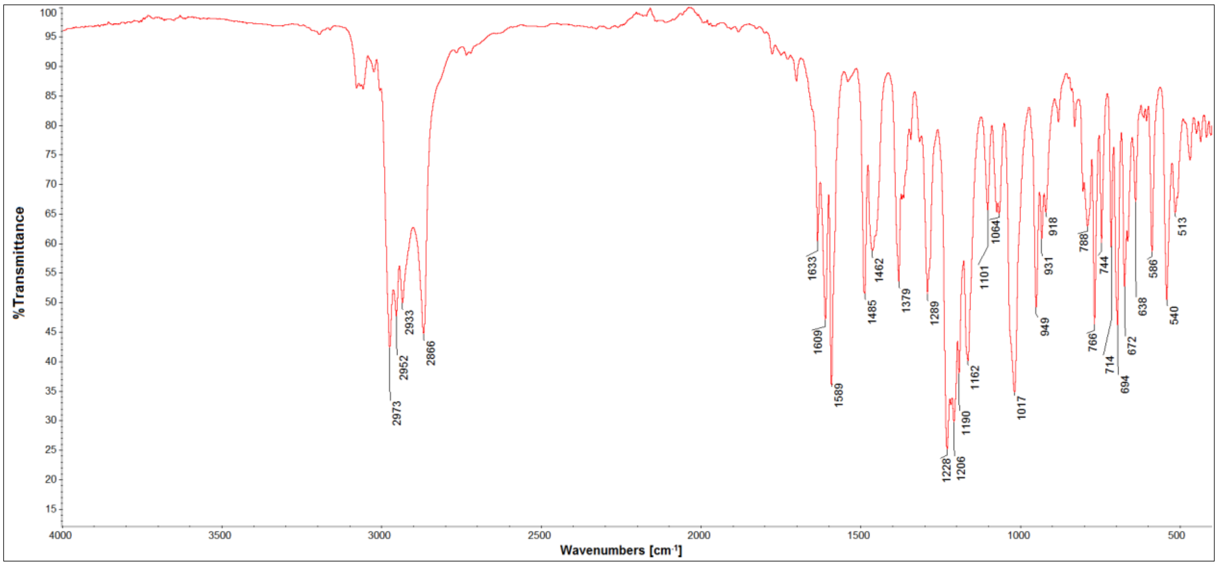

FIG. S107. IR SPECTRUM OF SOLID **6C**

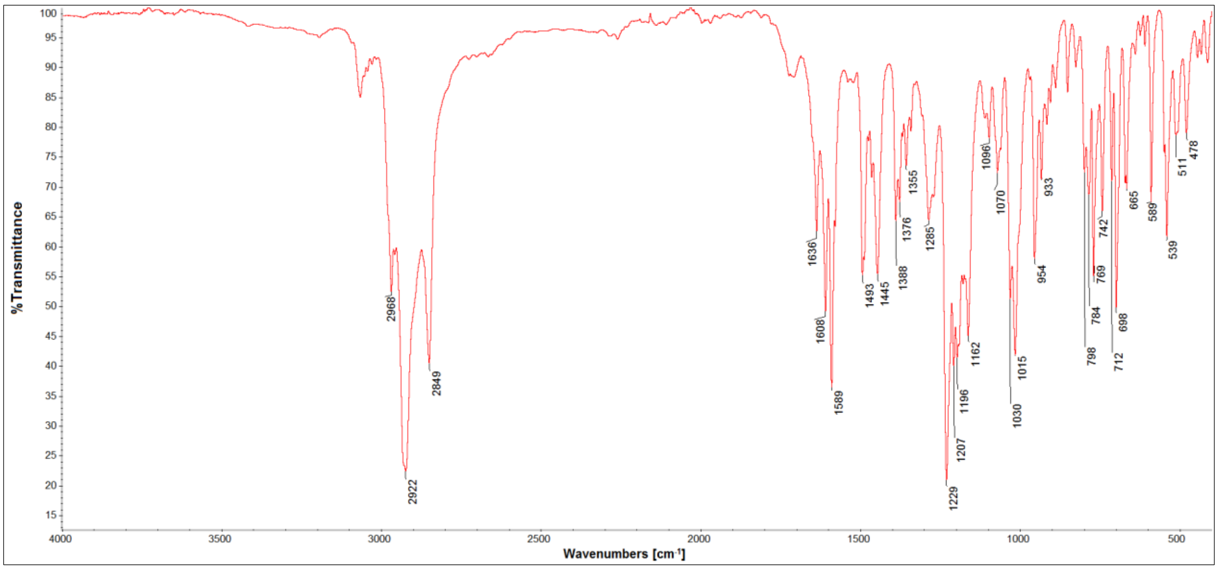

FIG. S108. IR SPECTRUM OF SOLID **9C**

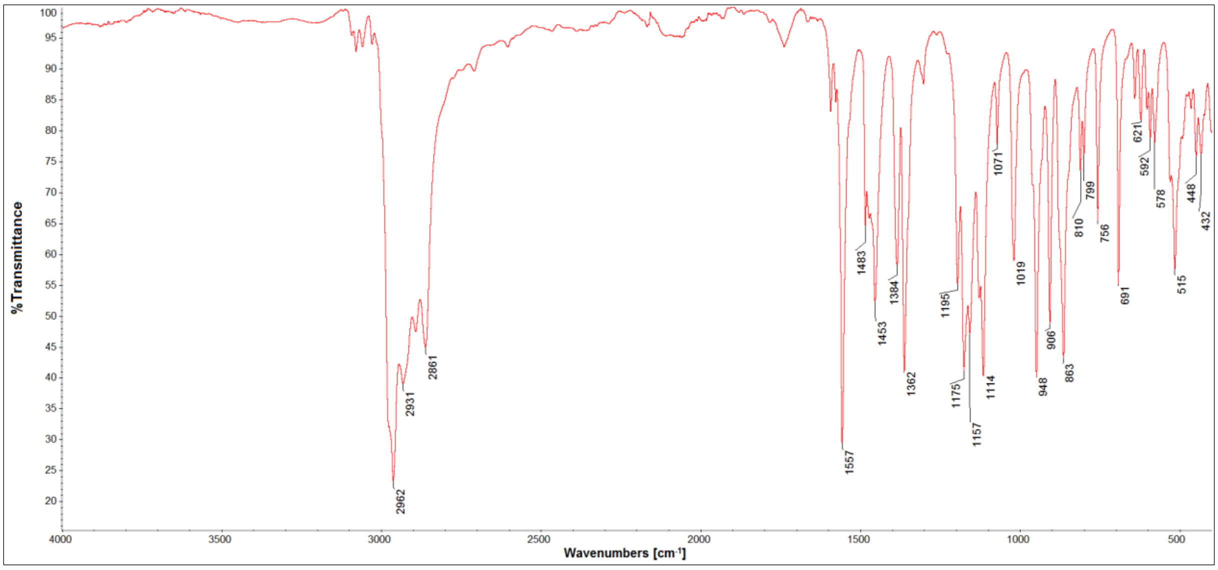

FIG. S109. IR SPECTRUM OF SOLID **1D** AND **1D'**

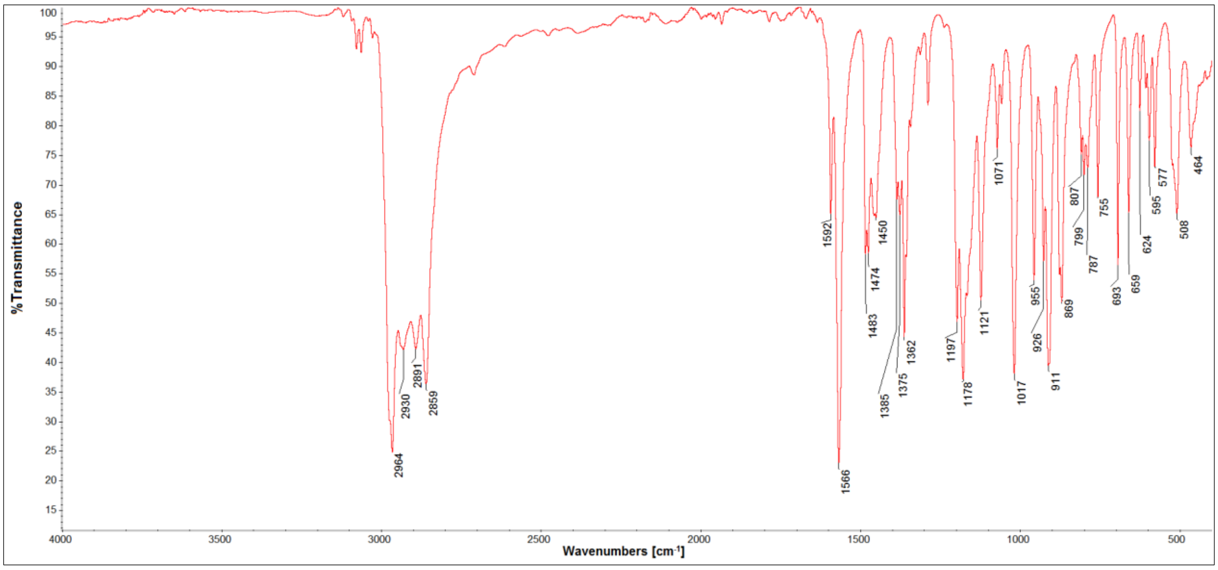

FIG. S110. IR SPECTRUM OF SOLID **2D**

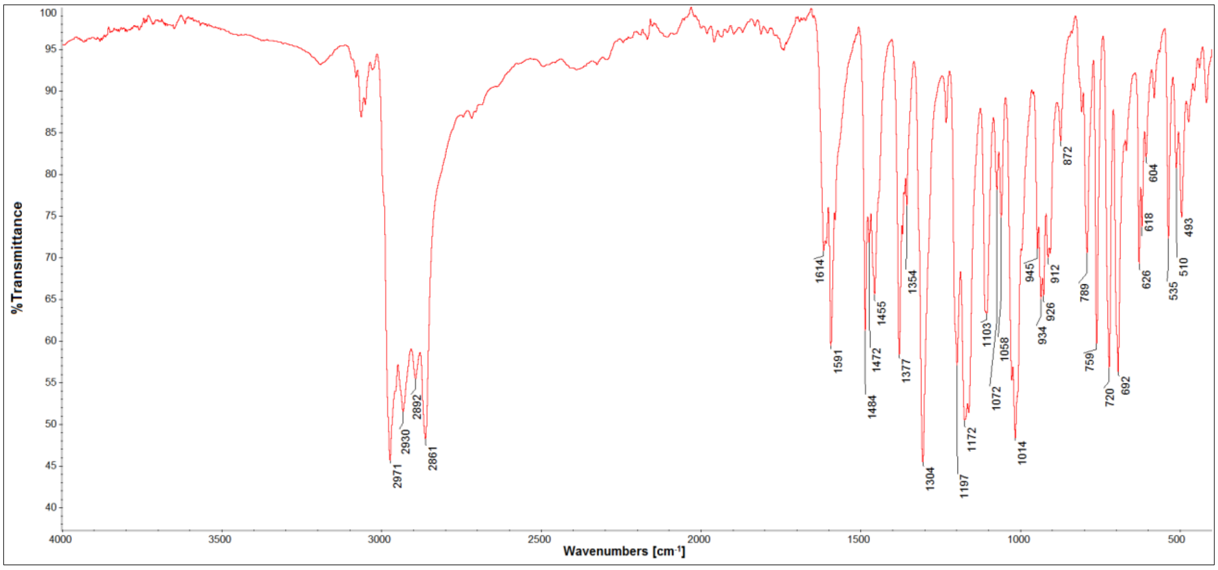

FIG. S111. IR SPECTRUM OF SOLID **3D** AND **3D'**

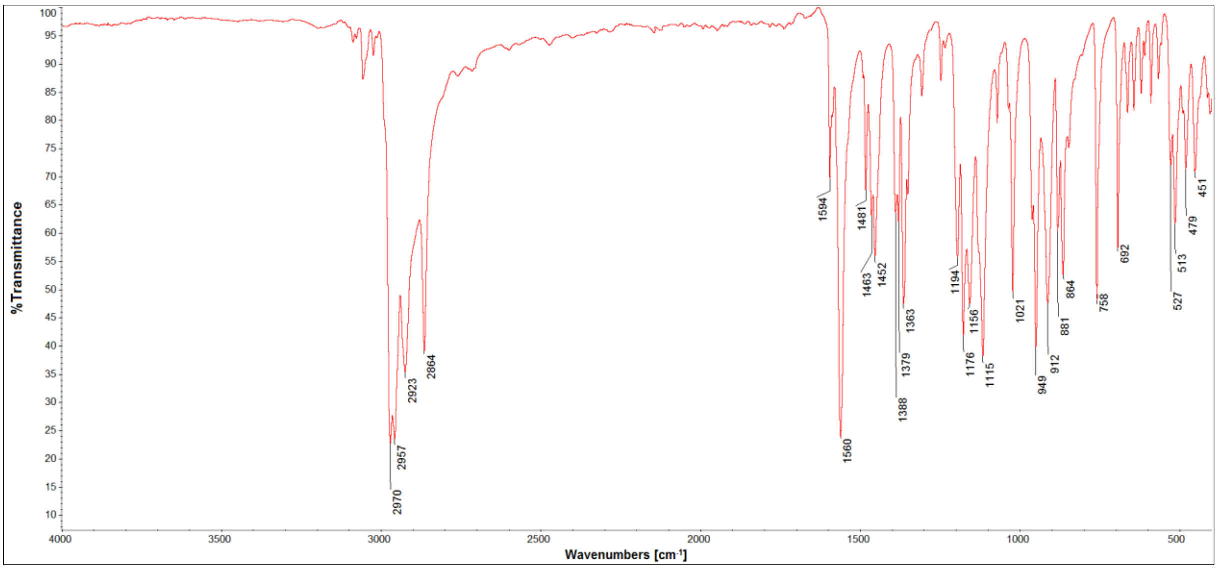

FIG. S112. IR SPECTRUM OF SOLID **4D** AND **4D'**

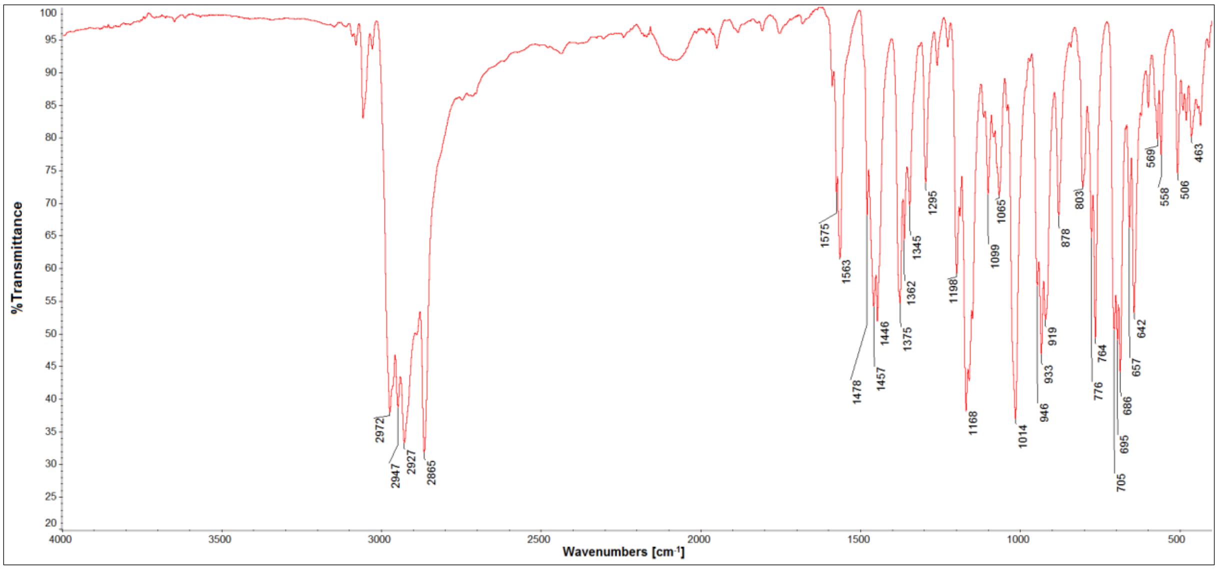

FIG. S113. IR SPECTRUM OF SOLID **6D**

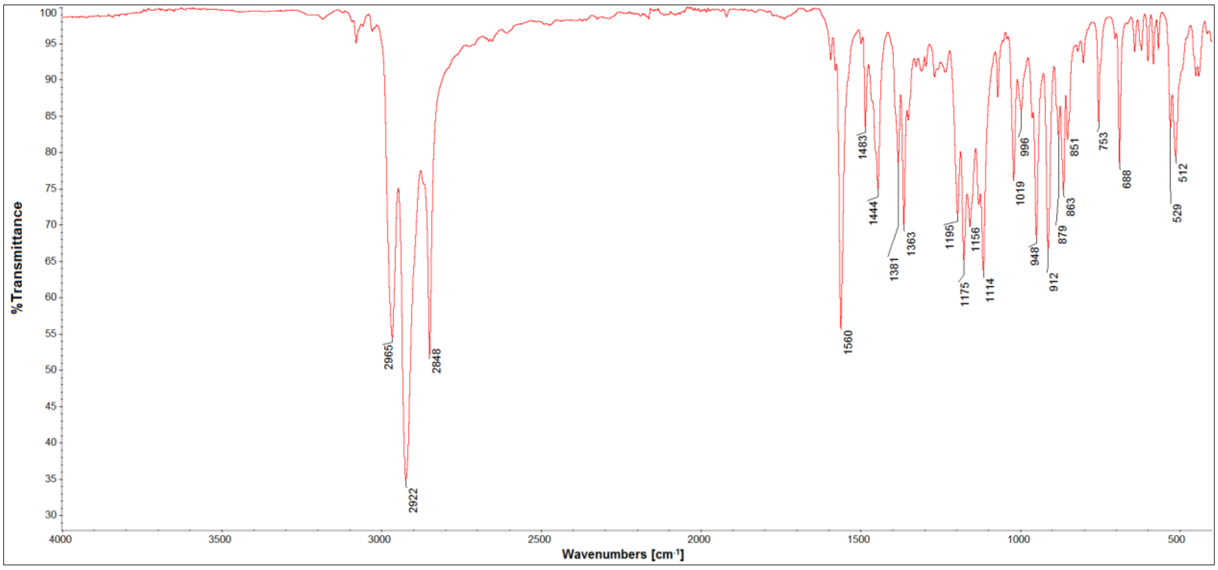

FIG. S114. IR SPECTRUM OF SOLID **7D** AND **7D’**

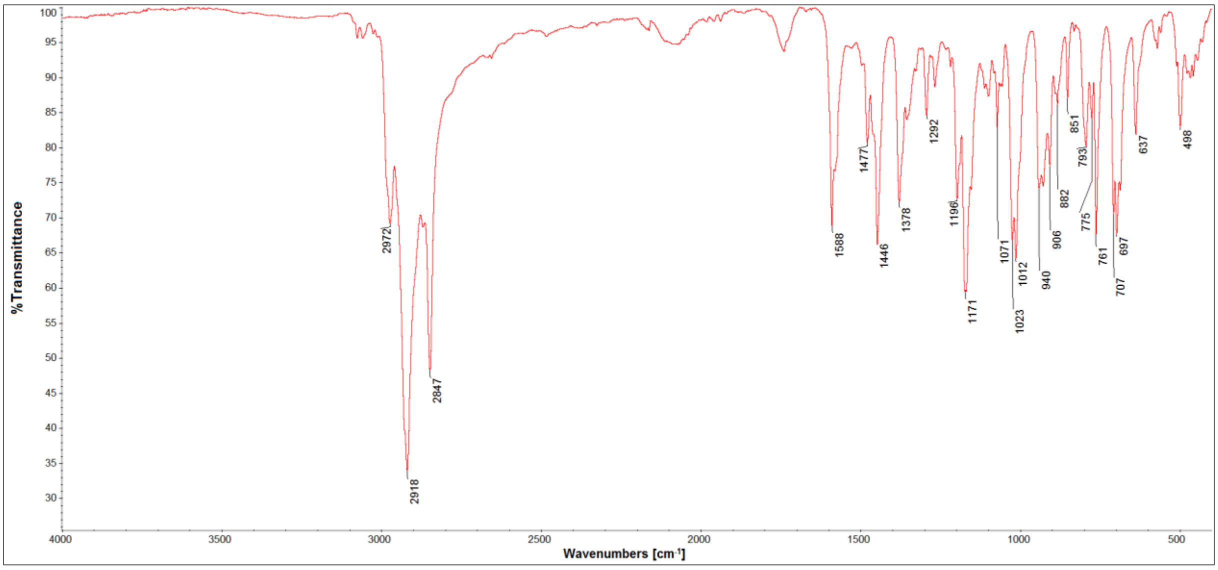

FIG. S115. IR SPECTRUM OF SOLID **9D**

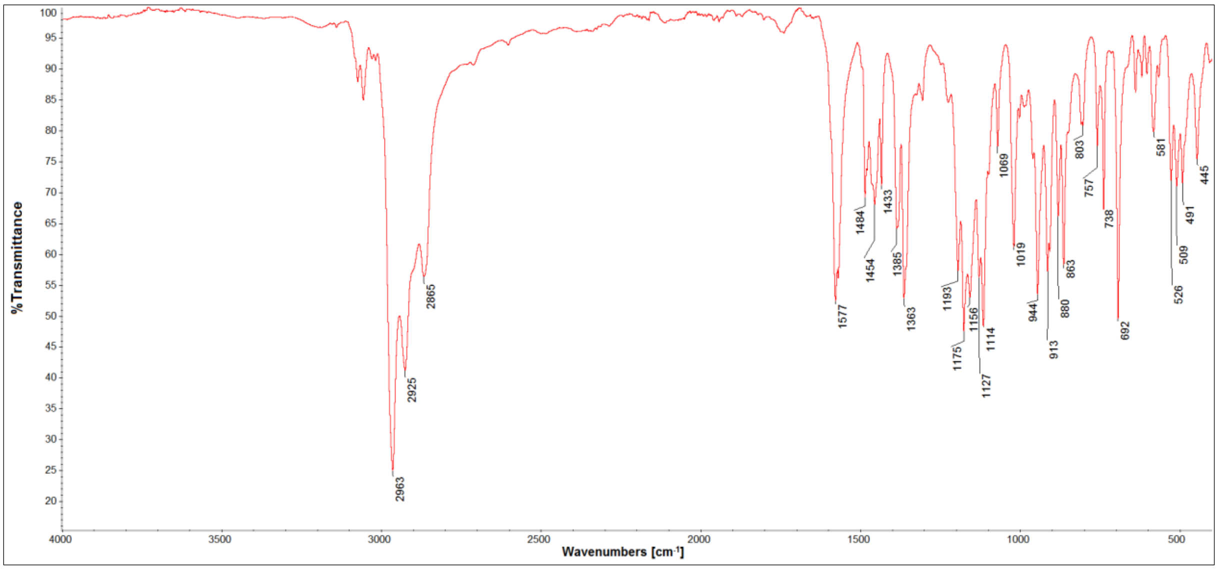

FIG. S116. IR SPECTRUM OF SOLID **10D**

NMR studies of selected reactions

Variable temperature NMR study of selected compounds

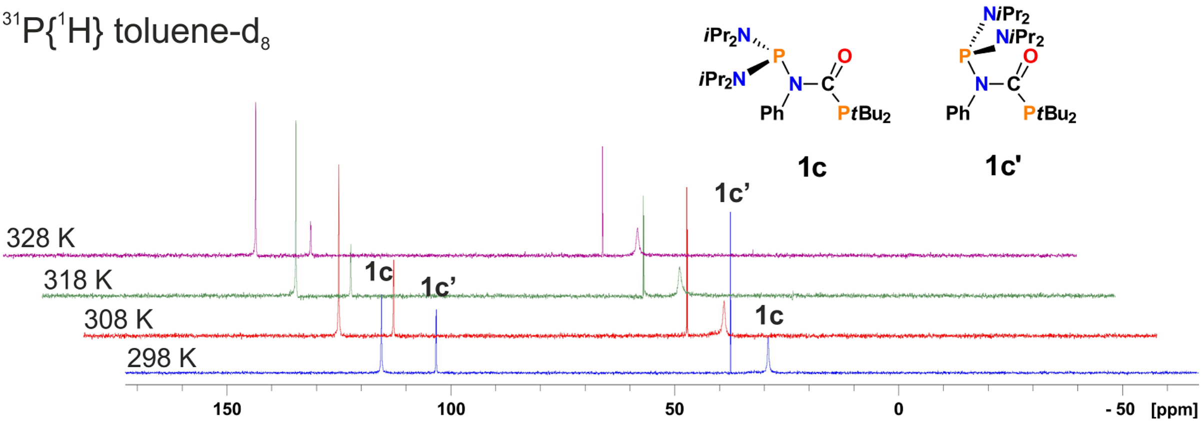

FIG. S117. $^{31}P\{^{1}H\}$ VT-NMR OF ISOLATED MIXTURE OF ISOMERS **1C** AND **1C'**

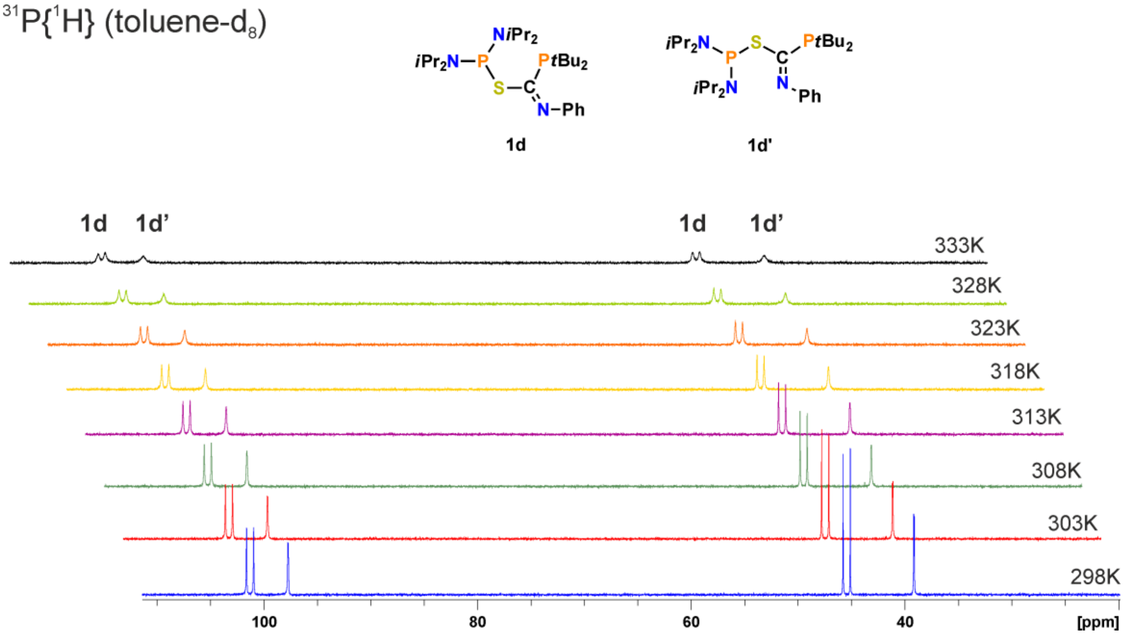

FIG. S118. $^{31}P\{^{1}H\}$ VT-NMR OF ISOLATED MIXTURE OF ISOMERS **1D** AND **1D'**

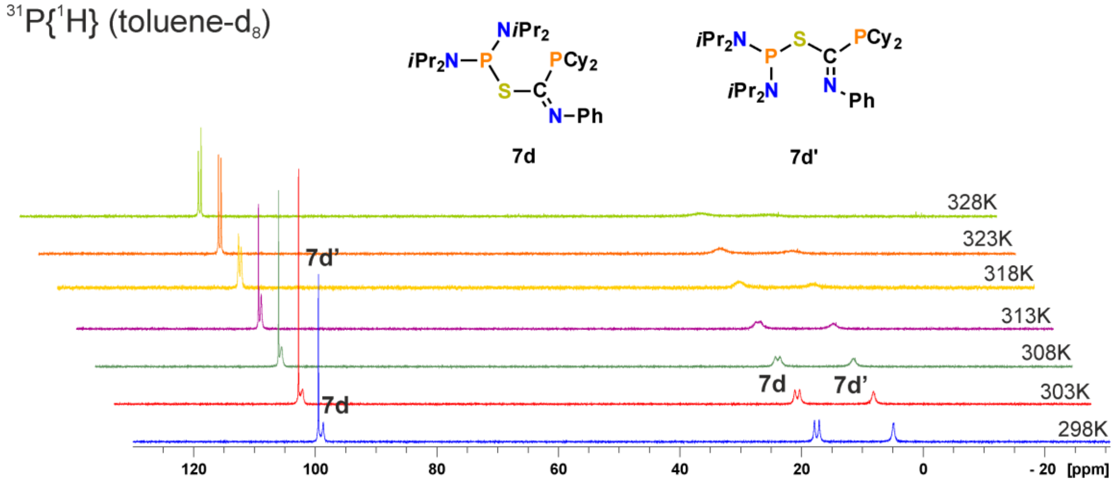

FIG. S119. $^{31}P\{^{1}H\}$ VT-NMR OF ISOLATED MIXTURE OF ISOMERS **7D** AND **7D'**

Monitoring of reaction progress by $^{31}P\{^1H\}$ NMR of selected reactions

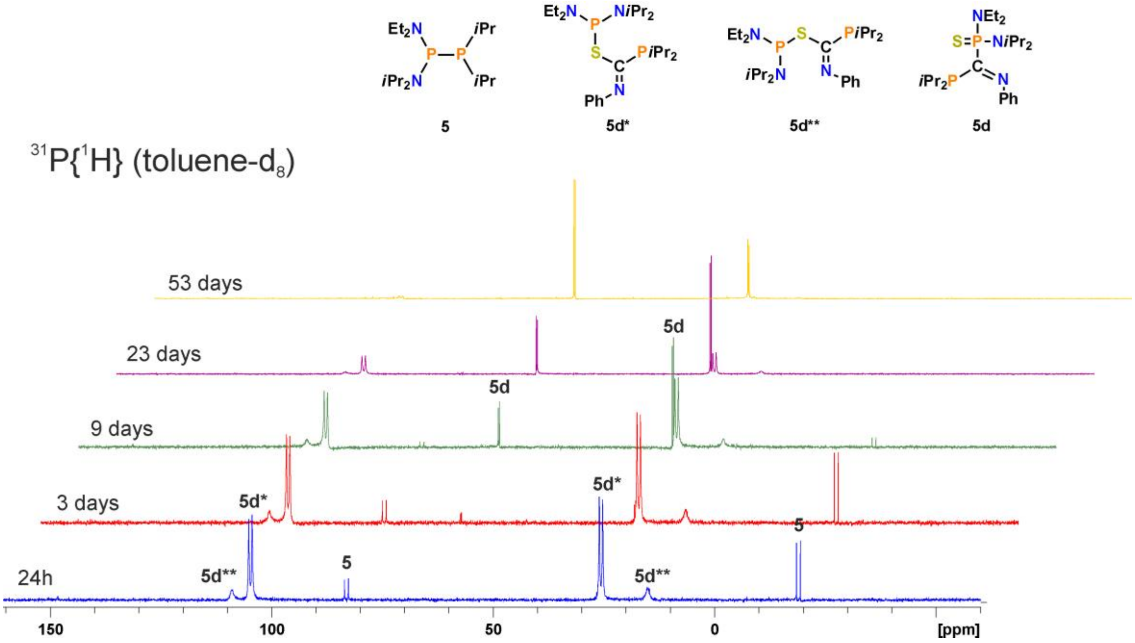

FIG. S120. MONITORING OF REACTION PROGRESS BY $^{31}P\{^1H\}$ NMR. REACTION OF **5** WITH PhNCS

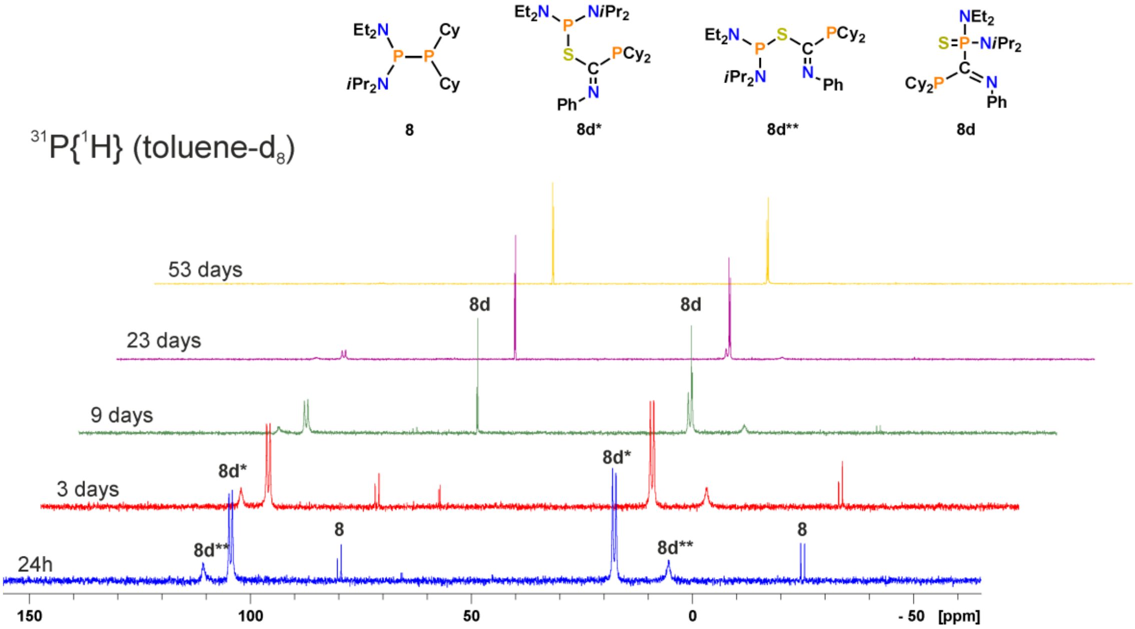

FIG. S121. MONITORING OF REACTION PROGRESS BY $^{31}P\{^1H\}$ NMR. REACTION OF **8** WITH PhNCS

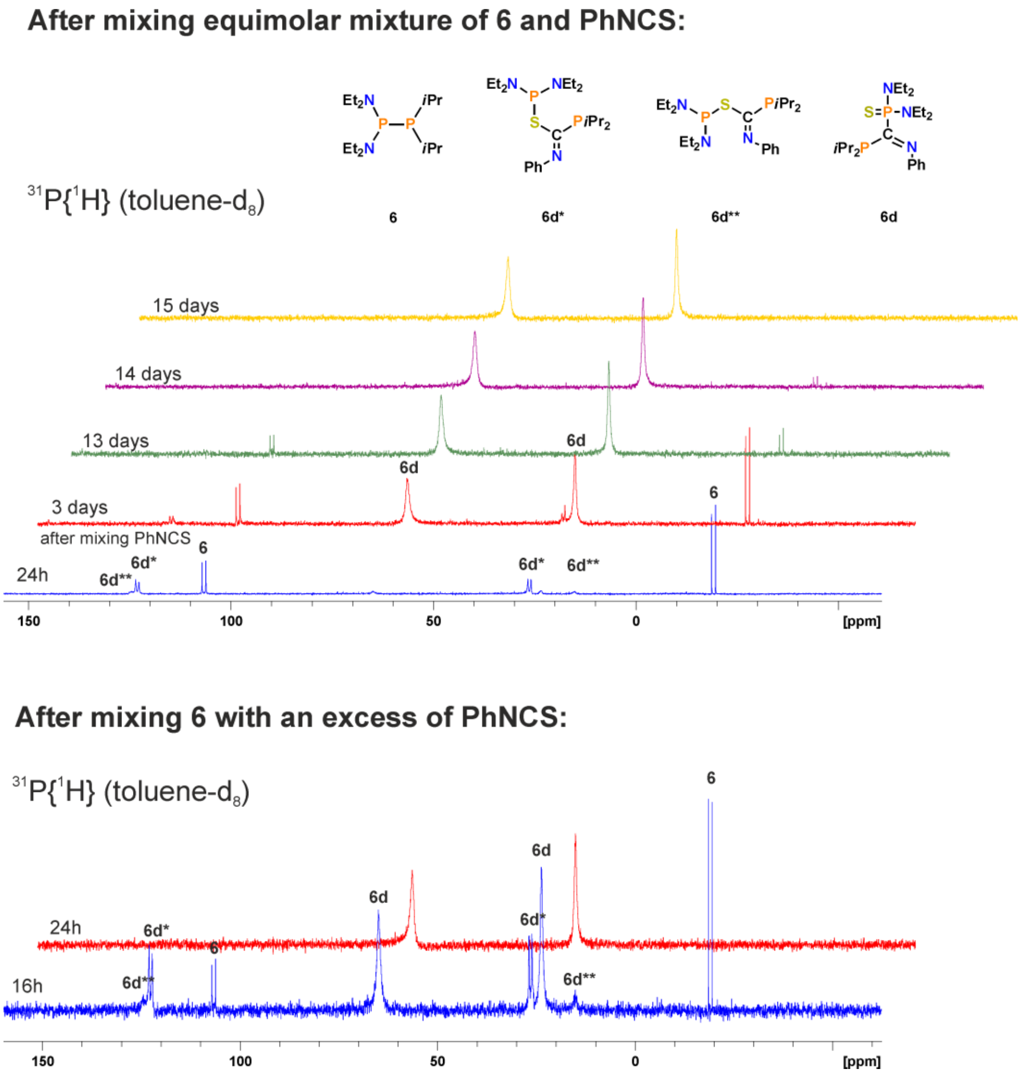

FIG. S122. MONITORING OF REACTION PROGRESS BY ^{31}P{^{1}H} NMR. REACTION OF **6** WITH PhNCS

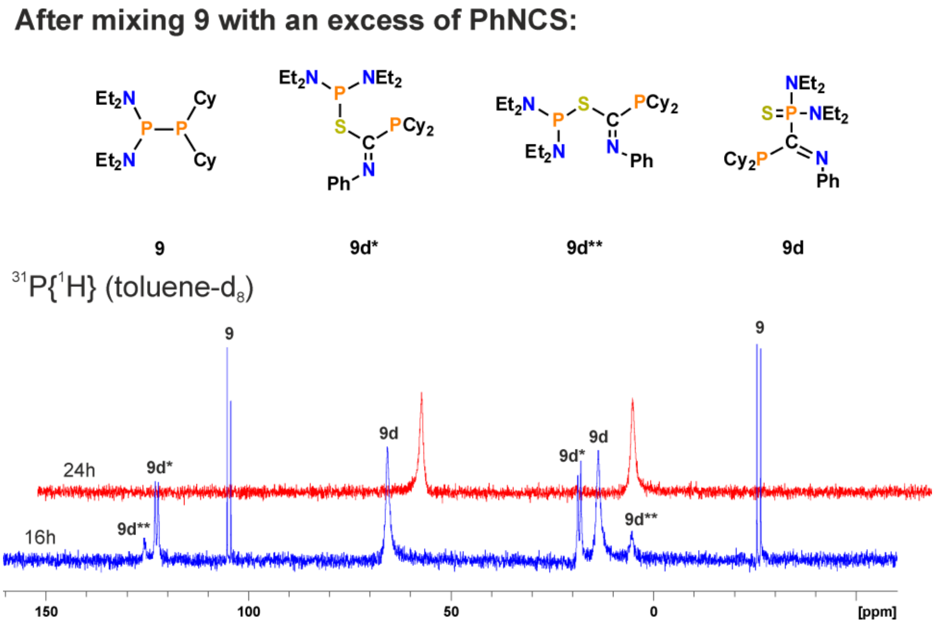

FIG. S123. MONITORING OF REACTION PROGRESS BY ^{31}P{^{1}H} NMR. REACTION OF **9** WITH PhNCS

TABLE S28. $^{31}P\{^{1}H\}$ NMR DATA OF PARENT DIPHOSPHANES **1-15**.

No.	Diphosphane	δP¹ [ppm]	δP² [ppm]	$^{1}J_{P-P}$ [Hz]
**1**[1]	tBu$_2$P^1-P^2(NiPr$_2$)$_2$	62.6	88.2	358.2
**2**[2]	tBu$_2$P^1-P^2(NEt$_2$)(NiPr$_2$)	35.3	98.9	247.0
**3**[1]	tBu$_2$P^1-P^2(NEt$_2$)$_2$	11.8	111.1	193.7
**4**	iPr$_2$P^1-P^2(NiPr$_2$)$_2$	-14.0	63.9	146.7
**5**	iPr$_2$P^1-P^2(NEt$_2$)(NiPr$_2$)	-19.0	83.1	145.3
**6**	iPr$_2$P^1-P^2(NEt$_2$)$_2$	-19.0	106.6	152.6
**7**	Cy$_2$P^1-P^2(NiPr$_2$)$_2$	-18.7	60.3	138.9
**8**	Cy$_2$P^1-P^2(NEt$_2$)(NiPr$_2$)	-25.0	79.8	138.1
**9**	Cy$_2$P^1-P^2(NEt$_2$)$_2$	-25.9	104.8	152.6
**10**[1]	tBuPhP^1-P^2(NiPr$_2$)$_2$	-9.5	72.2	155.3
**11**[1]	tBuPhP^1-P^2(NEt$_2$)(NiPr$_2$)	-13.7[a]<br>-14.9[b]	79.5[a]<br>79.3[b]	143.1[a]<br>140.4[b]
**12**[1]	tBuPhP^1-P^2(NEt$_2$)$_2$	-15.7	99.5	145.3
**13**[1]	Ph$_2$P^1-P^2(NiPr$_2$)$_2$	-38.0	71.8	119.3
**14**	Ph$_2$P^1-P^2(NEt$_2$)(NiPr$_2$)	-40.4	88.5	123.5
**15**[1]	Ph$_2$P^1-P^2(NEt$_2$)$_2$	-38.3	108.4	135.0

[a] p-*meso* isomers; [b] p-*rac* isomers

TABLE S29. $^{31}P\{^1H\}$ NMR DATA OF DIPHOSPHINATION PRODUCTS (A: CO2; B: CS2; C: PHNCO, D: PHNCS).

No.	δP[1] [ppm]	δP[2] [ppm]	J_{P-P} [Hz]
**1a**[2]	47.4	113.4	14.5
**2a**[2]	46.1	119.4	14.5
**4a**	20.9	114.4	14.5
**7a**	12.6	114.3	14.5
**1b**[2]	75.0	106.6	14.5
**2b**[2]	74.9	112.6	10.5
**3b**[2]	75.9	128.9	14.5
**4b**	63.2	108.5	14.5
**5b**	63.1	114.0	14.5
**6b**	64.9	132.0	21.8
**7b**	56.0	108.3	14.5
**8b**	56.5	114.1	14.5
**9b**	58.5	131.7	21.8
**10b**	67.3	109.7	14.5
**13b**	42.4	111.8	14.5
**1c/1c'**	37.3/29.1	103.3/115.5	14.5/14.5[a]
**2c/2c'**	29.8/30.6	14.5/14.7	-/-
**3c**	16.8	31.4	-
**6c**	12.0	16.6	-
**9c**	4.0	16.7	-
**1d/1d'**	45.1/39.0	101.2/97.7	109.0/8.0
**2d**	43.0	106.6	130.8
**3d/3d'**	39.8/43.6	71.5/68.2	-/-
**3d***	44.2	126.4	138.1
**4d/4d'**	24.6/13.9	97.9/98.7	130.8/14.5
**5d**	26.5	65.7	43.6
**5d*/5d****	25.3/15.1	104.9/109.2	130.8/[b]
**6d**	23.4	65.1	[b]
**7d/7d'**	16.8/3.8	97.7/98.9	130.8/[b]
**8d**	17.2	65.6	43.6
**8d*/8d****	17.5/5.3	104.4/110.6	130.8/[b]
**9d**	13.6	65.8	[b]
**10d**	25.0	101.0	130.8

[a] value of coupling for spectra recorded at 273 K; for spectra recorded at 298 K, coupling is not visible due to the broadness of the signals; [b] coupling is not visible due to the broadness of the signals

DFT calculations

General methods

All calculations presented in the paper were performed using the Gaussian 09[8] program package. Molecular geometries of all compounds were optimized using density functional theory at the TPSSTPSS functional by Tao *et al*[9] with 6-31+G(d,p) basis set. The TPSSTPSS exchange-correlation functional has been chosen, as it has good overall performance for the description of main-group element compounds. Adding the GD3BJ keyword that includes the D3 version of Grimme dispersion with Becke-Johnson[10] damping into calculations also accounts for long-range and dispersion interactions. Molecular geometries were energy-optimized, and the most stable (the lowest energy) conformer was identified during the potential energy surface scanning. Nature of the final gas-phase geometries as local minima (no imaginary frequencies) or transition states (one imaginary frequency) on the potential energy surface was then validated by harmonic frequency calculations at the same level of theory. Values of calculated energies, enthalpies and Gibbs free-energies derived from thermochemical calculations were corrected for the zero-point energy (ZPE). Scans of potential energy surface along the P-C, P-N, P-O, P-S or C-N bonds were performed to establish local minima corresponding to transition products and confirm proposed mechanism of the reaction. Local maxima related to transition states were also established and validated by IRC calculations (to confirm that a located saddle points lie on the minimum energy path between assumed minima) and used to determine energy barriers between respective transformations. Values of energy barriers $\Delta G^{\ddagger}$ and $\Delta H^{\ddagger}$ of reactions **1c, 3c** and **3d** were determined as the difference between energy of rate-determining transition state and rate-determining intermediate as described in [[11]]. Harmonic frequency calculations were also used to identify frequencies (stretching vibrations) corresponding with fragments of incorporated molecules (C=N, C=O or C=S bonds) and correctly assign them to the bands on experimental IR spectra.

Condensed Fukui functions[12] and dual descriptors[12,13] were determined using optimized structures to single point calculations on diphosphanes **1-15** for *N*, *N-1* and *N+1* electron states at the TPSSTPSS//6-31+G(d,p) level of theory. Condensed to atom parameters were calculated using partial charges derived *via* Hirshfeld population analysis. NBO analysis was performed for non-optimized structures derived from X-ray analysis at TPSSTPSS//6-31+G(d,p) level of theory by applying the NBO 3.1[14] module built-in Gaussian 09.

TABLE S30. SELECTED COMPUTATIONAL PARAMETERS OBTAINED FOR CONSIDERED SYSTEMS (IN ATOMIC UNITS A.U.): $\mathbf{E_0}$ - ELECTRONIC ENERGY; $\mathbf{E_{0+...}}$ - SUM OF ELECTRONIC AND: $\mathbf{E_{ZPE}}$ - ZERO-POINT ENERGIES, $\mathbf{E_{THERM}}$ - THERMAL ENERGIES, **H –** THERMAL ENTHALPIES, **G -** THERMAL FREE ENERGIES CALCULATED AT TPSSTPSS//6-31+G(d,p) LEVEL OF THEORY.

Substrates							
Compound	E_{electr} [A.U.]	$\varepsilon_0 + E_{ZPE}$ [A.U.]	$\varepsilon_0 + E_{therm}$ [A.U.]	ε_0 + H [A.U.]	ε_0 + G [A.U.]	ΔH^o_{298K} [kcal mol^{-1}]	ΔG^o_{298K} [kcal mol^{-1}]
1	-1582.393660	-1581.761670	-1581.727375	-1581.726431	-1581.822142	-	-
2	-1503.744201	-1503.167782	-1503.136211	-1503.135267	-1503.225826	-	-
3	-1425.094200	-1424.572467	-1424.543796	-1424.542852	-1424.627845	-	-
4	-1503.754931	-1503.178710	-1503.147515	-1503.146571	-1503.237297	-	-
5	-1425.104134	-1424.583497	-1424.554093	-1424.553148	-1424.641556	-	-
6	-1346.447892	-1345.982438	-1345.955783	-1345.954839	-1346.038566	-	-
7	-1737.311051	-1736.604269	-1736.568679	-1736.567735	-1736.670125	-	-
8	-1658.659961	-1658.008783	-1657.975901	-1657.974957	-1658.071938	-	-
9	-1580.001681	-1579.406081	-1579.375652	-1579.374708	-1579.468659	-	-

10	-1656.231222	-1655.630596	-1655.597001	-1655.596057	-1655.692924	-	-
11	-1577.582162	-1577.037440	-1577.006422	-1577.005478	-1577.097473	-	-
11'	-1577.581375	-1577.036672	-1577.005549	-1577.004605	-1577.097493	-	-
12	-1498.924225	-1498.435316	-1498.406686	-1498.405742	-1498.495518	-	-
13	-1730.054144	-1729.485468	-1729.452384	-1729.451440	-1729.549141	-	-
14	-1651.399618	-1650.886775	-1650.856157	-1650.855213	-1650.949952	-	-
15	-1572.743792	-1572.285972	-1572.258226	-1572.257282	-1572.345450	-	-
CO_2	-188.617512	-188.606279	-188.603617	-188.602673	-188.627020	-	-
CS_2	-834.521371	-834.514556	-834.511428	-834.510484	-834.537516	-	-
PhNCO	-399.841622	-399.739475	-399.732237	-399.731293	-399.771924	-	-
PhNCS	-722.799981	-722.700113	-722.692507	-722.691563	-722.733681	-	-
Formation of **1c** and **1c'**							
Compound	E_{electr} [A.U.]	$\varepsilon_0 + E_{ZPE}$ [A.U.]	$\varepsilon_0 + E_{therm}$ [A.U.]	ε_0 + H [A.U.]	ε_0 + G [A.U.]	ΔH^o_{298K} [kcal mol^{-1}]	ΔG^o_{298K} [kcal mol^{-1}]
$TS1_A$	-1982.233612	-1981.498959	-1981.456225	-1981.455281	-1981.573769	1.6	13.0
$I1_A$	-1982.246860	-1981.509292	-1981.467414	-1981.466470	-1981.578786	-5.6	9.8
$TS2_A$	-1982.221357	-1981.486503	-1981.444196	-1981.443252	-1981.559390	9.3	22.2
$TS1_B$	-1982.232042	-1981.497137	-1981.454506	-1981.453561	-1981.572923	2.7	13.5
$I1_B$	-1982.246406	-1981.509406	-1981.46721	-1981.466266	-1981.579375	-5.5	9.4
$TS2_B$	-1982.222855	-1981.487353	-1981.44541	-1981.444465	-1981.557731	8.5	23.3
1c	-1982.269598	-1981.533964	-1981.491222	-1981.490277	-1981.606682	-20.8	-8.1
1c'	-1982.271012	-1981.534865	-1981.492395	-1981.491451	-1981.607029	-21.6	-8.3
1c"	-1982.264805	-1981.529708	-1981.487263	-1981.486319	-1981.60214	-18.3	-5.2
Formation of **3c**							
Compound	E_{electr} [A.U.]	$\varepsilon_0 + E_{ZPE}$ [A.U.]	$\varepsilon_0 + E_{therm}$ [A.U.]	ε_0 + H [A.U.]	ε_0 + G [A.U.]	ΔH^o_{298K} [kcal mol^{-1}]	ΔG^o_{298K} [kcal mol^{-1}]
$TS1_A$	-1824.925050	-1824.301013	-1824.264745	-1824.263801	-1824.367401	6.6	20.7
$I1_A$	-1824.932554	-1824.306880	-1824.269724	-1824.268780	-1824.375818	3.4	15.3
$TS2_A$	-1824.921522	-1824.295837	-1824.259516	-1824.258571	-1824.362136	10.0	24.1
$I2_A$ (3c**)	-1824.950428	-1824.325812	-1824.288307	-1824.287363	-1824.398741	-8.5	0.7
TS3	-1824.910045	-1824.287005	-1824.249751	-1824.248807	-1824.358938	16.2	26.1
I3	-1824.959083	-1824.333212	-1824.296326	-1824.295381	-1824.401241	-13.6	-0.9
TS4	-2224.799058	-2224.069113	-2224.024658	-2224.023714	-2224.145413	-11.7	16.8
I4	-2224.814945	-2224.082166	-2224.038297	-2224.037353	-2224.155566	-20.4	10.3
TS5	-2224.781179	-2224.050895	-2224.006801	-2224.005857	-2224.128466	-0.3	27.7
3c	-2224.837453	-2224.106065	-2224.061131	-2224.060187	-2224.184826	-35.1	-8.4
$TS1_B$	-1824.922927	-1824.299341	-1824.261922	-1824.260977	-1824.370043	8.4	19.0
$I1_B$	-1824.936490	-1824.310965	-1824.273741	-1824.272797	-1824.379456	0.9	13.0
$TS2_B$	-1824.926558	-1824.301538	-1824.265085	-1824.264141	-1824.368391	6.4	20.1
3c*	-1824.959299	-1824.334076	-1824.296868	-1824.295924	-1824.403650	-13.9	-2.5
Formation of **2c**/**2c'**, **6c** and **9c**							
Compound	E_{electr} [A.U.]	$\varepsilon_0 + E_{ZPE}$ [A.U.]	$\varepsilon_0 + E_{therm}$ [A.U.]	ε_0 + H [A.U.]	ε_0 + G [A.U.]	ΔH^o_{298K} [kcal mol^{-1}]	ΔG^o_{298K} [kcal mol^{-1}]
2c	-2303.492154	-2302.704743	-2302.657481	-2302.656537	-2302.783726	-37.6	-9.0
2c'	-2303.488633	-2302.701089	-2302.653903	-2302.652959	-2302.780042	-35.3	-6.6
6c	-2146.189277	-2145.513256	-2145.470906	-2145.469962	-2145.590312	-33.6	-5.1
9c	-2379.743229	-2378.936613	-2378.891639	-2378.890695	-2379.016683	-34.2	-2.7

Formation of **1d** and **1d'**							
Compound	E_{electr} [A.U.]	$\varepsilon_0 + E_{ZPE}$ [A.U.]	$\varepsilon_0 + E_{therm}$ [A.U.]	$\varepsilon_0 + H$ [A.U.]	$\varepsilon_0 + G$ [A.U.]	ΔH^o_{298K} [kcal mol^{-1}]	ΔG^o_{298K} [kcal mol^{-1}]
1d	-2305.229795	-2304.496805	-2304.453408	-2304.452464	-2304.57084	-22.1	-9.6
1d'	-2305.229255	-2304.496523	-2304.453055	-2304.452111	-2304.571272	-21.8	-9.9
Formation of **2d**							
Compound	E_{electr} [A.U.]	$\varepsilon_0 + E_{ZPE}$ [A.U.]	$\varepsilon_0 + E_{therm}$ [A.U.]	$\varepsilon_0 + H$ [A.U.]	$\varepsilon_0 + G$ [A.U.]	ΔH^o_{298K} [kcal mol^{-1}]	ΔG^o_{298K} [kcal mol^{-1}]
2d	-2226.575203	-2225.897504	-2225.856873	-2225.855928	-2225.969895	-18.6	-6.7
2d'	-2226.570739	-2225.893527	-2225.852606	-2225.851662	-2225.967417	-15.9	-5.1
2d*	-2949.396129	-2225.898272	-2225.85882	-2225.857876	-2225.965353	-19.9	-3.7
2d**	-2949.402075	-2948.618667	-2948.570907	-2948.569963	-2948.697643	-33.0	-2.9
Formation of **3d** and **3d'**							
Compound	E_{electr} [A.U.]	$\varepsilon_0 + E_{ZPE}$ [A.U.]	$\varepsilon_0 + E_{therm}$ [A.U.]	$\varepsilon_0 + H$ [A.U.]	$\varepsilon_0 + G$ [A.U.]	ΔH^o_{298K} [kcal mol^{-1}]	ΔG^o_{298K} [kcal mol^{-1}]
TS1	-2147.876509	-2147.254736	-2147.217097	-2147.216152	-2147.325089	11.7	23.3
I1	-2147.896665	-2147.273019	-2147.235589	-2147.234645	-2147.341554	-0.1	12.8
TS2	-2147.886615	-2147.263855	-2147.227032	-2147.226088	-2147.331107	5.3	19.5
I2 (3d*)	-2147.917384	-2147.295064	-2147.257107	-2147.256163	-2147.365225	-13.9	-2.4
3d**	-2147.915089	-2147.292775	-2147.254799	-2147.253854	-2147.363274	7.4	19.0
TS3	-2147.882768	-2147.261626	-2147.223853	-2147.222909	-2147.331911	-15.9	-2.0
I3	-2147.921677	-2147.297392	-2147.260166	-2147.259221	-2147.364678	-9.7	20.4
TS4	-2870.713343	-2869.987092	-2869.942121	-2869.941176	-2870.063428	-20.6	11.3
I4	-2870.732538	-2870.003620	-2869.959131	-2869.958187	-2870.077595	-3.4	28.4
TS5	-2870.703738	-2869.976767	-2869.932209	-2869.931265	-2870.050839	-35.5	-6.3
3d	-2870.755163	-2870.027696	-2869.982327	-2869.981382	-2870.105098	11.7	23.3
Formation of **4d** and **4d'**							
Compound	E_{electr} [A.U.]	$\varepsilon_0 + E_{ZPE}$ [A.U.]	$\varepsilon_0 + E_{therm}$ [A.U.]	$\varepsilon_0 + H$ [A.U.]	$\varepsilon_0 + G$ [A.U.]	ΔH^o_{298K} [kcal mol^{-1}]	ΔG^o_{298K} [kcal mol^{-1}]
4d	-2226.582655	-2225.90576	-2225.864461	-2225.863517	-2225.981835	-16.3	-7.0
4d'	-2226.580991	-2225.903778	-2225.862771	-2225.861827	-2225.977567	-15.2	-4.2
Formation of **5d**							
Compound	E_{electr} [A.U.]	$\varepsilon_0 + E_{ZPE}$ [A.U.]	$\varepsilon_0 + E_{therm}$ [A.U.]	$\varepsilon_0 + H$ [A.U.]	$\varepsilon_0 + G$ [A.U.]	ΔH^o_{298K} [kcal mol^{-1}]	ΔG^o_{298K} [kcal mol^{-1}]
5d*	-2147.92749	-2147.305372	-2147.26708	-2147.266136	-2147.377336	-13.7	-1.3
5d**	-2147.923841	-2147.301821	-2147.263565	-2147.262621	-2147.37375	-11.5	1.0
5d	-2147.933715	-2147.308502	-2147.271621	-2147.270677	-2147.373546	-16.6	1.1
Formation of **6d**							
Compound	E_{electr} [A.U.]	$\varepsilon_0 + E_{ZPE}$ [A.U.]	$\varepsilon_0 + E_{therm}$ [A.U.]	$\varepsilon_0 + H$ [A.U.]	$\varepsilon_0 + G$ [A.U.]	ΔH^o_{298K} [kcal mol^{-1}]	ΔG^o_{298K} [kcal mol^{-1}]
6d*	-2069.266054	-2068.697859	-2068.662306	-2068.661362	-2068.769592	-9.6	1.7
6d**	-2069.265626	-2068.698455	-2068.663094	-2068.66215	-2068.76824	-10.1	2.6
6d	-2069.284022	-2068.714623	-2068.680318	-2068.679374	-2068.778109	-21.1	-3.8
6d***	-2792.108739	-2791.436467	-2791.393634	-2791.39269	-2791.512398	-35.0	-4.1
Formation of **7d** and **7d'**							
Compound	E_{electr} [A.U.]	$\varepsilon_0 + E_{ZPE}$ [A.U.]	$\varepsilon_0 + E_{therm}$ [A.U.]	$\varepsilon_0 + H$ [A.U.]	$\varepsilon_0 + G$ [A.U.]	ΔH^o_{298K} [kcal mol^{-1}]	ΔG^o_{298K} [kcal mol^{-1}]

7d	-2460.138256	-2459.330553	-2459.285902	-2459.284958	-2459.410585	-16.4	-4.3
7d'	-2460.136198	-2459.328643	-2459.283962	-2459.283018	-2459.408395	-15.2	-2.9
Formation of **8d**							
Compound	E_{electr} [A.U.]	$\varepsilon_0 + E_{ZPE}$ [A.U.]	$\varepsilon_0 + E_{therm}$ [A.U.]	ε_0 + H [A.U.]	ε_0 + G [A.U.]	ΔH^o_{298K} [kcal mol^{-1}]	ΔG^o_{298K} [kcal mol^{-1}]
8d*	-2381.479677	-2380.727557	-2380.68554	-2380.684596	-2380.805847	-11.6	-0.1
8d**	-2381.47663	-2380.723981	-2380.682044	-2380.6811	-2380.802729	-9.3	1.9
8d	-2381.48974	-2380.734004	-2380.693602	-2380.692658	-2380.804479	-16.7	0.7
Formation of **9d**							
Compound	E_{electr} [A.U.]	$\varepsilon_0 + E_{ZPE}$ [A.U.]	$\varepsilon_0 + E_{therm}$ [A.U.]	ε_0 + H [A.U.]	ε_0 + G [A.U.]	ΔH^o_{298K} [kcal mol^{-1}]	ΔG^o_{298K} [kcal mol^{-1}]
9d*	-2302.824035	-2302.12668	-2302.087485	-2302.08654	-2302.202532	-13.0	-0.1
9d**	-2302.820498	-2302.122924	-2302.083847	-2302.082903	-2302.19869	-10.7	2.3
9d	-2302.839388	-2302.139783	-2302.101596	-2302.100652	-2302.210812	-22.0	-5.4
9d***	-3025.665743	-3024.863076	-3024.816601	-3024.815656	-3024.944813	-37.0	-5.6
Formation of **10d** and **10d'**							
Compound	E_{electr} [A.U.]	$\varepsilon_0 + E_{ZPE}$ [A.U.]	$\varepsilon_0 + E_{therm}$ [A.U.]	ε_0 + H [A.U.]	ε_0 + G [A.U.]	ΔH^o_{298K} [kcal mol^{-1}]	ΔG^o_{298K} [kcal mol^{-1}]
10d	-2379.058019	-2378.357222	-2378.314286	-2378.313342	-2378.434307	-16.5	-4.9
10d'	-2379.055687	-2378.354525	-2378.311756	-2378.310812	-2378.43053	-14.9	-2.5

Philicity of reactive centres

TABLE S31. VALUES OF NUCLEOPHILIC (***f***$_N$), ELECTROPHILIC (***f***$_E$) FUKUI FUNCTIONS AND DUAL DESCRIPTOR (**Δ*f***) CALCULATED FOR DIPHOSPHANES **1-15**, AND C=E MOLECULES USING PARTIAL CHARGES DERIVED VIA HIRSHFELD POPULATION ANALYSIS AT TPSSTPSS//6-31+G(D,P) LEVEL OF THEORY.

Diphosphanes **1-15**						
Compound	$(R_2N)(R'_2N)$**P**			RR'**P**		
	f_N	f_E	Δ*f*	f_N	f_E	Δ*f*
1	0.091	0.004	-0.087	0.116	0.003	-0.113
2	0.088	0.006	-0.082	0.123	0.008	-0.114
3	0.099	0.010	-0.089	0.121	0.018	-0.103
4	0.096	0.009	-0.087	0.115	0.019	-0.096
5	0.091	0.011	-0.080	0.116	0.024	-0.092
6	0.086	0.041	-0.045	0.115	0.048	-0.067
7	0.097	0.005	-0.091	0.115	0.011	-0.103
8	0.092	0.007	-0.085	0.121	0.014	-0.107
9	0.092	0.009	-0.083	0.134	0.015	-0.118
10	0.093	0.017	-0.076	0.069	0.036	-0.033
11	0.087	0.016	-0.071	0.071	0.039	-0.033
12	0.049	0.026	-0.022	0.076	0.046	-0.030
13	0.087	0.034	-0.053	0.072	0.054	-0.017
14	0.069	0.033	-0.036	0.060	0.056	-0.004
15	0.040	0.029	-0.011	0.079	0.062	-0.017
Compound	**C**=E			C=**E**		
	f_N	f_E	Δ*f*	f_N	f_E	Δ*f*
O=**C=O**	0.228	0.571	0.344	0.386	0.214	-0.172
S=**C=S**	0.088	0.163	0.075	0.456	0.419	-0.037
PhN**C=O**	0.090	0.106	0.017	0.144	0.113	-0.031
PhN**C=S**	0.060	0.097	0.037	0.320	0.258	-0.062

TABEL S32. POLARISATION OF THE C=O AND C=S BOND IN THE ANALYSED HETEROCUMULENES. PARTIAL CHARGES WERE DERIVED *VIA* HIRSHFELD POPULATION ANALYSIS AT THE TPSSTPSS// 6-31+G(D,P) LEVEL OF THEORY

**Compound**	**Hirshfeld atomic charges**		**Δq (q_C - $q_{O/S}$)**
	**q_C**	**$q_{O/S}$**	
O=**C=O**	0.305	-0.152	0.457
S=**C=S**	0.007	-0.004	0.011
PhN**C=O**	0.218	-0.180	0.399
PhN**C=S**	0.073	-0.103	0.176

Values of free energy of formation of considered products

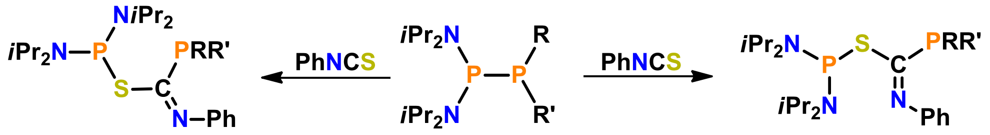

1d: ΔG^0_{1d} = -9.6 kcal mol^{-1}
4d: ΔG^0_{4d} = -7.0 kcal mol^{-1}
7d: ΔG^0_{7d} = -4.3 kcal mol^{-1}
10d: ΔG^0_{10d} = -4.9 kcal mol^{-1}

1: R, R' = *t*Bu
4: R, R' = *i*Pr
7: R, R' = Cy
10: R = *t*Bu, R' = Ph

1d': $\Delta G^0_{1d'}$ = -9.9 kcal mol^{-1}
4d': $\Delta G^0_{4d'}$ = -4.2 kcal mol^{-1}
7d': $\Delta G^0_{7d'}$ = -2.9 kcal mol^{-1}
10d': $\Delta G^0_{10d'}$ = -2.5 kcal mol^{-1}

SCHEME. S2. FREE ENERGY VALUES OF FORMATION OF PRODUCTS RESULTING FROM THE REACTION OF **1**, **4**, **7** AND **10** WITH PhNCS.

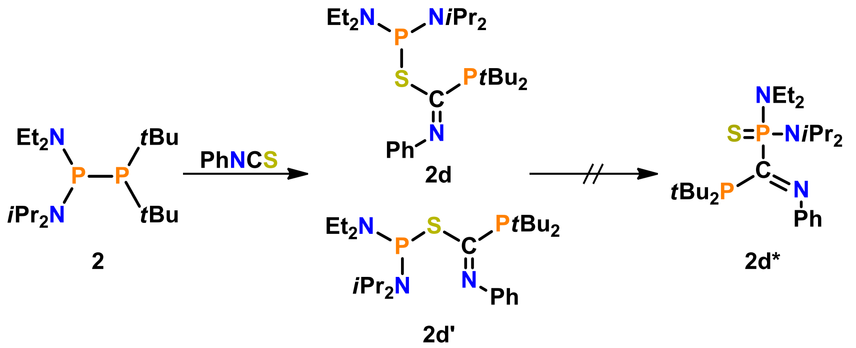

ΔG^0_{2d} = -6.7 kcal mol^{-1}
$\Delta G^0_{2d'}$ = -5.1 kcal mol^{-1} ΔG^0_{2d*} = -3.7 kcal mol^{-1}

SCHEME. S3. FREE ENERGY VALUES OF FORMATION OF PRODUCTS RESULTING FROM THE REACTION OF **2** WITH PhNCS.

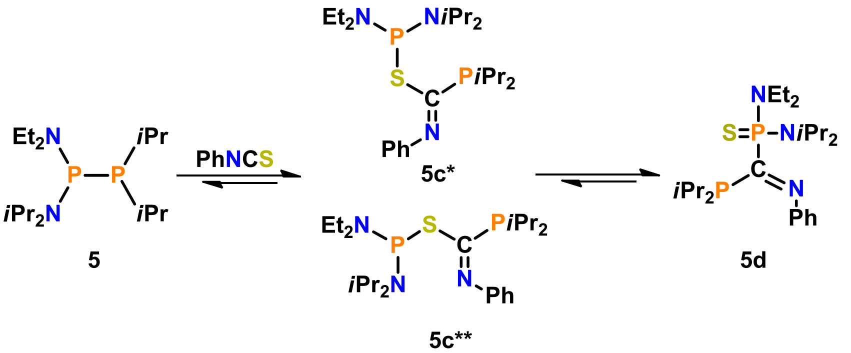

ΔG^0_{5d*} = -1.3 kcal mol^{-1}
ΔG^0_{5d**} = 1.0 kcal mol^{-1} ΔG^0_{5d} = 1.1 kcal mol^{-1}

SCHEME. S4. FREE ENERGY VALUES OF FORMATION OF PRODUCTS RESULTING FROM THE REACTION OF **5** WITH PhNCS.

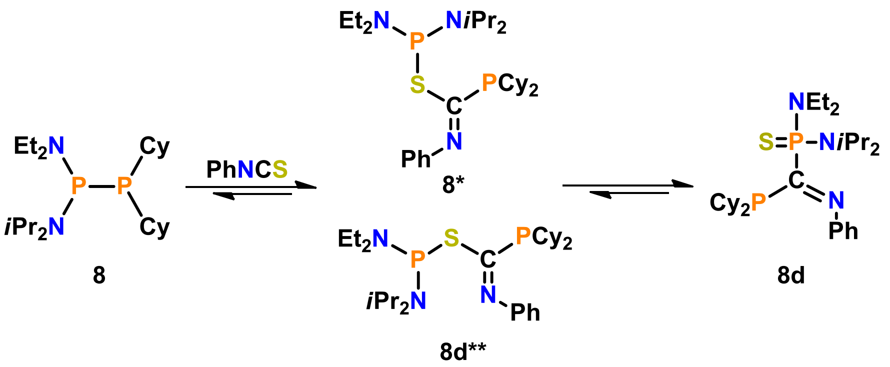

ΔG^0_{8d*} = -0.1 kcal mol^{-1}

ΔG^0_{8d**} = 1.9 kcal mol^{-1} ΔG^0_{8d} = 0.7 kcal mol^{-1}

SCHEME. S5. FREE ENERGY VALUES OF FORMATION OF PRODUCTS RESULTING FROM THE REACTION OF **8** WITH PhNCS.

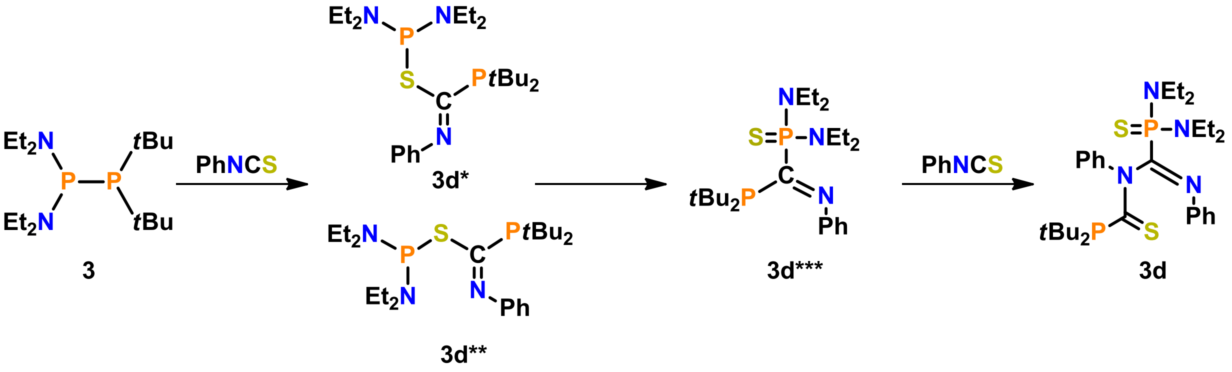

ΔG^0_{3d*} = -2.4 kcal mol^{-1}

ΔG^0_{3d**} = -1.1 kcal mol^{-1} ΔG^0_{3d***} = -2.0 kcal mol^{-1} ΔG^0_{3d} = -6.3 kcal mol^{-1}

SCHEME. S6. FREE ENERGY VALUES OF FORMATION OF PRODUCTS RESULTING FROM THE REACTION OF **3** WITH PhNCS.

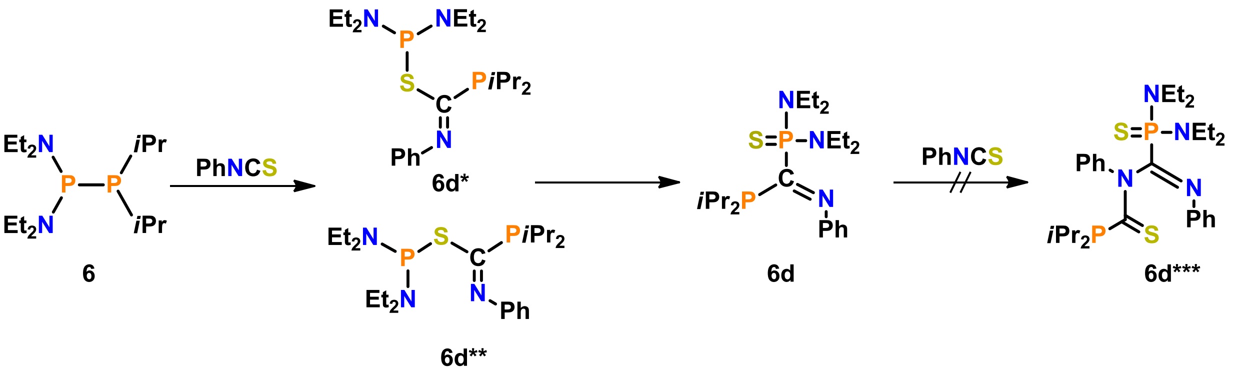

ΔG^0_{6d*} = 1.7 kcal mol^{-1}

ΔG^0_{6d**} = 3.0 kcal mol^{-1} ΔG^0_{6d} = -3.8 kcal mol^{-1} ΔG^0_{6d***} = -4.1 kcal mol^{-1}

SCHEME. S7. FREE ENERGY VALUES OF FORMATION OF PRODUCTS RESULTING FROM THE REACTION OF **6** WITH PhNCS.

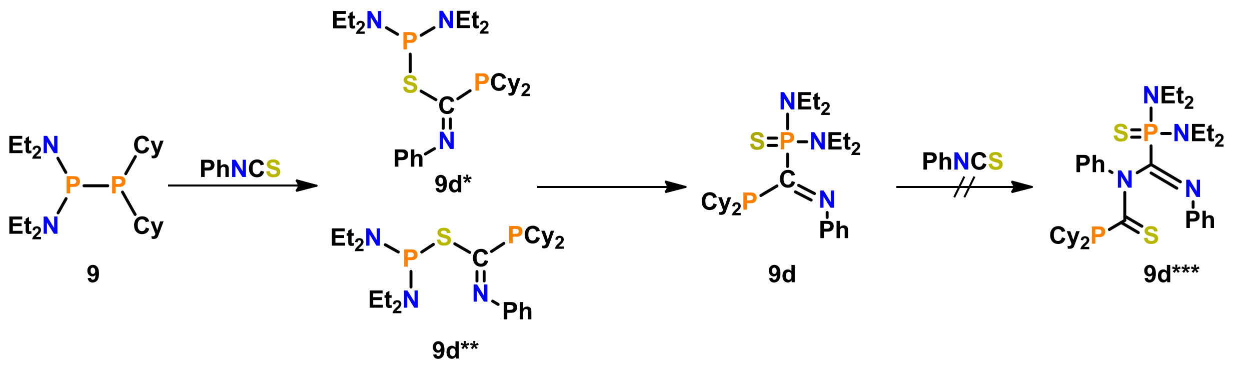

$\Delta G^0_{9d^*}$ = -0.1 kcal mol^{-1}

$\Delta G^0_{9d^{**}}$ = 2.3 kcal mol^{-1} ΔG^0_{9d} = -5.4 kcal mol^{-1} $\Delta G^0_{9d^{***}}$ = -5.6 kcal mol^{-1}

SCHEME. S8. FREE ENERGY VALUES OF FORMATION OF PRODUCTS RESULTING FROM THE REACTION OF **9** WITH PhNCS.

NBO analysis

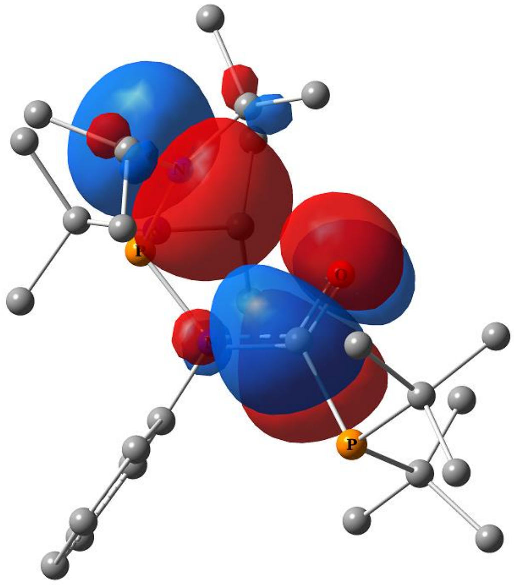

n_{N} - $\pi^{*}_{C=O}$

FIG. S124. NATURAL BOND ORBITALS REPRESENTING SELECTED INTERACTIONS WITHIN THE STRUCTURE OF **1C.**

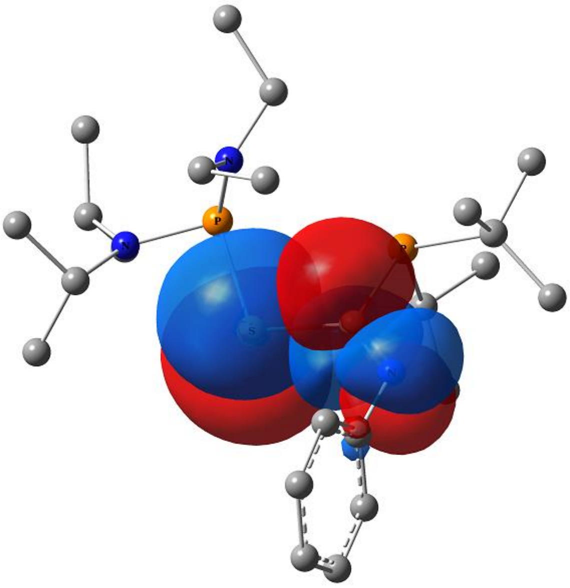

n_{S} - $\pi^{*}_{C=N}$

FIG. S125. NATURAL BOND ORBITALS REPRESENTING SELECTED INTERACTIONS WITHIN THE STRUCTURE OF **2D.**

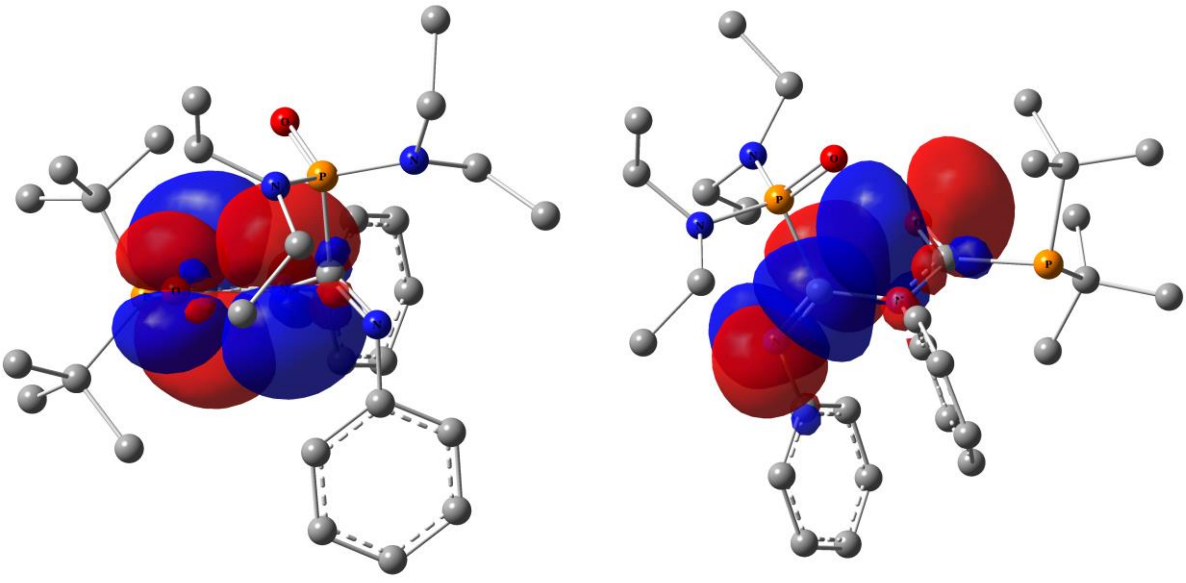

$n_{\mathbf{N}}$ - $\pi^*_{\mathbf{C=O}}$ $n_{\mathbf{O}}$ - $\pi^*_{\mathbf{N=C}}$

FIG. S126. NATURAL BOND ORBITALS REPRESENTING SELECTED INTERACTIONS WITHIN THE STRUCTURE OF **3C.**

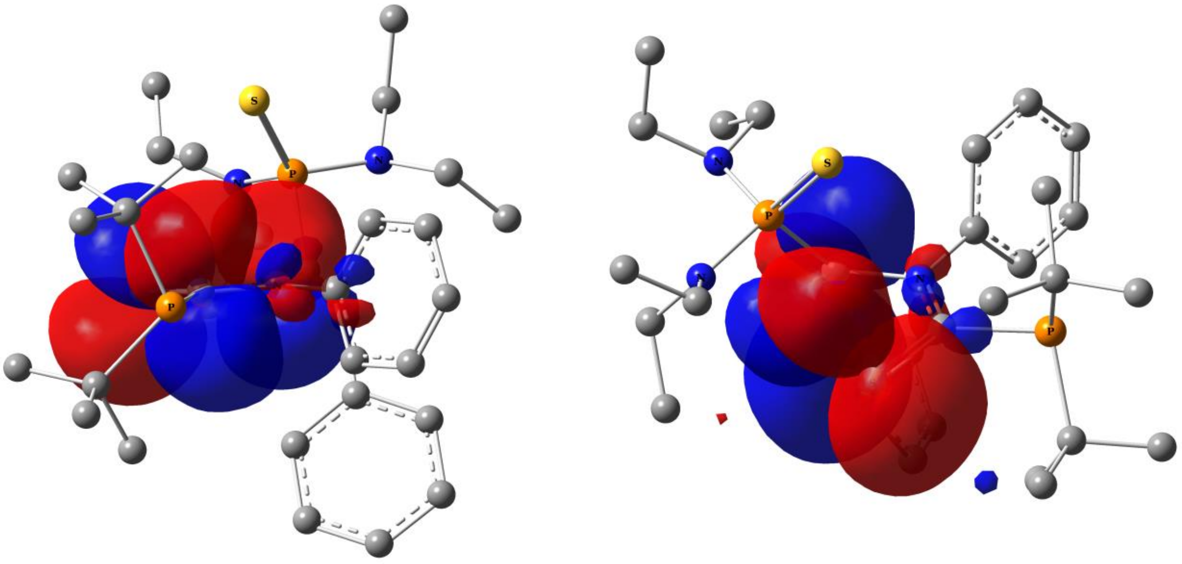

$n_{\mathbf{N}}$ - $\pi^*_{\mathbf{C=S}}$ n_{S} - $\pi^*_{C=N}$

FIG. S127. NATURAL BOND ORBITALS REPRESENTING SELECTED INTERACTIONS WITHIN THE STRUCTURE OF **3D.**

Optimized structures and Cartesian coordinates

To ease following the reaction mechanisms and provide quick access to the structural properties of all substrates, intermediates, transition states and products, their Cartesian coordinates were collected in one cif file. It is added to this ESI pdf file as the attachment, which contains a list of DFT-optimized molecular structures from which the xyz file may be extracted if needed.

Optimized molecular structures.cif

References

(1) Szynkiewicz, N.; Ponikiewski, Ł.; Grubba, R. Symmetrical and Unsymmetrical Diphosphanes with Diversified Alkyl, Aryl, and Amino Substituents. *Dalt. Trans.* **2018**, *47* (47), 16885–16894. https://doi.org/10.1039/C8DT03775B.

(2) Szynkiewicz, N.; Ponikiewski, Ł.; Grubba, R. Diphosphination of CO_2 and CS_2 Mediated by Frustrated Lewis Pairs-Catalytic Route to Phosphanyl Derivatives of Formic and Dithioformic Acid. *Chem. Commun.* **2019**, *55* (20), 2928–2931. https://doi.org/10.1039/c9cc00621d..

(3) STOE &Cie GmbH, X-Area 1.75, STOE &Cie GmbH. Darmstadt, Germany 2015.

(4) Sheldrick, G. M. SHELXT - Integrated Space-Group and Crystal-Structure Determination. *Acta Crystallogr. Sect. A Found. Crystallogr.* **2015**, *A71*, 3–8.

(5) Sheldrick, G. M. Crystal Structure Refinement with SHELXL. *Acta Cryst. C* **2015**, *71*, 3–8. https://doi.org/10.1107/S2053229614024218.

(6) Westrip, S. P. PublCIF: Software for Editing, Validating and Formatting Crystallographic Information Files. *J. Appl. Crystallogr.* **2010**, *43*, 920–925.

(7) Dolomanov, O. V; Bourhis, L. J.; Gildea, R. J.; Howard, J. A. K.; Puschmann, H. OLEX2: A Complete Structure Solution, Refinement and Analysis Program. *J. Appl. Crystallogr.* **2009**, *42*, 339–341. https://doi.org/10.1107/S0021889808042726.

(8) Frisch, M. J.; Trucks, G. W.; Schlegel, H. B.; Scuseria, G. E.; Robb, M. A.; Cheeseman, J. R.; Scalmani, G.; Barone, V.; Petersson, G. A.; Nakatsuji, H.; Li, X.; Caricato, M.; Marenich, A.; Bloino, J.; Janesko, B. G.; Gomperts, R.; Mennucci, B.; Hratchian, H. P.; Ortritz, J. V.; Izmaylov, A. F.; Sonnenberg, J. L.; Williams-Young, D.; Ding, F.; Lipparini, F.; Egidi, F.; Goings, J.; Peng, B.; Petrone, A.; Henderson, T.; Ranasinghe, D.; Zakrzewski, V. G.; Gao, J.; Rega, N.; Zheng, G.; Liang, W.; Hada, M.; Ehara, M.; Toyota, K.; Fukuda, R.; Hasegawa, J.; Ishida, M.; Nakajima, T.; Honda, Y.; Kitao, O.; Nakai, H.; Vreven, T.; Throsse, K.; Foresman, J. B.; Fox, D. J. Gaussian09 Revision D.01. Gaussian, Inc.: Wallingford CT 2016.

(9) Tao, J.; Perdew, J. P.; Staroverov, V. N.; Scuseria, G. E. Climbing the Density Functional Ladder: Nonempirical Meta–Generalized Gradient Approximation Designed for Molecules and Solids. *Phys. Rev. Lett.* **2003**, *91* (14), 146401. https://doi.org/10.1103/PhysRevLett.91.146401.

(10) Grimme, S.; Ehrlich, S.; Goerigk, L. Effect of the Damping Function in Dispersion Corrected Density Functional Theory. *J. Comput. Chem.* **2011**, *32* (7), 1456–1465. https://doi.org/10.1002/jcc.21759.

(11) Kozuch, S.; Shaik, S. How to Conceptualize Catalytic Cycles? The Energetic Span Model. *Acc. Chem. Res.* **2011**, *44* (2), 101–110. https://doi.org/10.1021/ar1000956.

(12) Parr, R. G.; Yang, W. Density Functional Approach to the Frontier-Electron Theory of Chemical Reactivity. *J. Am. Chem. Soc.* **1984**, *106* (14), 4049–4050. https://doi.org/10.1021/ja00326a036.

(13) Morell, C.; Grand, A.; Toro-Labbé, A. New Dual Descriptor for Chemical Reactivity. *J. Phys. Chem. A* **2005**, *109* (1), 205–212. https://doi.org/10.1021/jp046577a.

(14) Glendening, E. D.; Reed, A. E.; Carpenter, J. E.; Weinhold, F. NBO Version 3.1.
